# Supplementary material for: Immune resilience despite inflammatory stress promotes longevity and favorable health outcomes including resistance to infection
Source: Nat Commun. 2023 Jun 13;14:3286. doi: 10.1038/s41467-023-38238-6 (PMC10264401; doi:10.1038/s41467-023-38238-6)
Supplement: Supplementary file 1 — Supplementary Information [file 41467_2023_38238_MOESM1_ESM.pdf]

Supplementary information for

Immune resilience despite inflammatory stress promotes longevity and favorable health outcomes including resistance to infection

Abbreviations

AIC: Akaike information criteria
AIDS: Acquired immunodeficiency syndrome
ANOVA: Analysis of variance
ART: Antiretroviral therapy
BAS: Behavioral Activity Score
CC-RIX: Collaborative cross recombinant inbred intercrossed mice
CDC: Centers for Disease Control and Prevention
CI: Confidence interval
CMV: Cytomegalovirus
CoV: Coronavirus
CSCC: Cutaneous squamous cell cancer
CVD: Cardiovascular disease
dbGaP: Database of Genotypes and Phenotypes
DE: Differentially expressed
DILGOM: Dietary, Lifestyle, and Genetic determinants of Obesity and Metabolic syndrome
DNA: Deoxyribonucleic acid
ECMO: Extracorporeal membrane oxygenation
EDI: Estimated date of infection
EIC: Early HIV infection cohort
EMBL: European Molecular Biology Laboratory
FDR: False discovery rate
FHS: Framingham Heart Study
FSW: Female sex worker
GEE: Generalized estimating equations
GEO: Gene expression omnibus
GLM: Generalized linear model
GO-BP: Gene ontology biological process
GoDF: Gain of detrimental function
H-S: Hospitalized survivor
HAART: Highly active antiretroviral therapy
HIV: Human immunodeficiency virus
HR: Hazards ratio
HUGO: Human Genome Organization
IHG: Immunologic health grades
IC: Immunocompetence
IF: Inflammation
IQR: Interquartile range

IR: Immunologic resilience
IS: Immunosenescence
LoBF: Loss of beneficial function
LRT: Likelihood ratio test
LTB: Latent tuberculosis
MAS: Mortality-Associated Signatures
MOCS: The Kenya Majengo Observational Cohort Study
MSM: Men who have sex with men
MSW: Men who have sex with women
MTF: Military treatment facility
MV: Mechanical ventilation
NCBI: National Center for Biotechnology Information
NH-S: Non-hospitalized survivor
NHLBI: National Heart, Lung, and Blood Institute
NK: Natural killer cells
NS: Non-survivor
OR: Odds ratio
PBMC: Peripheral blood mononuclear cells
PCR: Polymerase chain reaction
PIC: Primary HIV infection cohort
PTB: Active pulmonary tuberculosis
qRT-PCR: Quantitative reverse transcription polymerase chain reaction
r: Correlation coefficient
RIN: RNA-seq integrity
RNA: Ribonucleic acid
RTR: Renal transplant recipient
SAFHS: San Antonio Family Heart Study
SARS: Severe acute respiratory syndrome
SAS: Survival-Associated Signatures
SD: Standard deviation
SIV: Simian immunodeficiency virus
SLE: Systemic lupus erythematosus
SNP: Single nucleotide polymorphism
START: Strategic timing of antiretroviral treatment
STI: Sexually transmitted infections
STROBE: Strengthening the reporting of observational studies in epidemiology
STVHCS: South Texas Veterans Health Care System
SVC: Spontaneous virologic controllers
TB: Tuberculosis
UCSD: University of California, San Diego
VA: Department of Veterans Affairs
VL: Viral load
WHO: World Health Organization

Table of Contents

A. Disclaimer 6

B. Acknowledgments 6

C. Supplementary materials and methods 8

Section 1. Cohorts studied 8
- 1.1. HIV-seronegative (HIV−) study groups 8
- 1.2. HIV-seropositive (HIV+) cohorts 12
- 1.3. Non-human primates 14
- 1.4. Collaborative Cross mice (CC-RIX mice – Ebola Infection) 15

Section 2: Study definitions, hypotheses, and statistical approach 16
- 2.1. Definitions. 16
- 2.2. Overarching hypothesis, cohorts, and comparisons 17
- 2.3. Rationale for studying distinct cohorts and the comparisons made 17
- 2.4. Biological plausibility and statistical approach 18

Section 3. Rationale for CD4+ T-cell count and CD4:CD8 ratio cutoffs used to define IHG grades 20
- 3.1. Pre-hoc selection of CD4+ cutoffs of 800 cells/mm^3 for IHGs 20
- 3.2. Pre-hoc selection of cutoffs for the CD4:CD8 ratio of 1.0 for IHGs 20
- 3.3. Additional considerations 20
- 3.4. Cardinal features of IHG-III or IHG-IV (ratio <1.0) 21

Section 4. Methods for associations in HIV− FSWs and COVID-19 21
- 4.1. Behavioral activity score (BAS) 21
- 4.2. Sexually transmitted infections (STI) scores 22
- 4.3. COVID-19 WHO ordinal scale 22

Section 5. Immunophenotyping methods used for assessment of immune correlates that associated with IHG status vs. age in the SardiNIA cohort 23

Section 6. Assessment of the correlation between indicators of T-cell responsiveness, T-cell dysfunction, and systemic inflammation and expression levels of SAS-1 and MAS-1 24
- 6.1. Indicators of T-cell responsiveness, T-cell dysfunction, and systemic inflammation 24

Section 7. RNA-seq in HIV+ persons and health controls 25
- 7.1. Methods 25
- 7.2. Study groups 25
- 7.3. RNA isolation 26
- 7.4. Library preparation and sequencing 26
- 7.5. Normalization and quality control for RNA-seq data 26

Section 8. Transcriptomic signature scores 27
- 8.1. Methods for IMM-AGE transcriptomic signature score 27
- 8.2. Survival-associated Score 1 (SAS-1) and Mortality-associated Score 1 (MAS-1) 28
- 8.3. Publicly available expression datasets 30

Section 9. Analysis of datasets of GEO and ArrayExpress 37
- 9.1. Overall 37
- 9.2. Data download and normalization 37
- 9.3. Probe-Gene information 38
- 9.4. Data analysis 38
- 9.5. Quality control of the dataset and interpretation 39
- 9.6. Survival analysis 39

Section 10. Predictors and outcomes **40**
10.1. Predictors: grades of antigenic stimulation and IR metrics 40
10.2. Key predictor-outcome dyads 40

Section 11. Statistical analysis and analytical approach **40**
11.1. Overview 40
11.2. Statistical analysis for levels of immune traits analyzed in the SardiNIA cohort 41
11.3. Details of statistical methods and analytical strategies per main figure panel 43
11.3.1. Figure 1 43
11.3.2. Figure 2 43
11.3.3. Figure 3 44
11.3.4. Figure 4 46
11.3.5. Figure 5 49
11.3.6. Figure 6 50
11.3.7. Figure 7 50
11.3.8. Figure 8 53
11.3.9. Figure 9 56
11.3.10. Figure 10 57
11.4. Details of statistical methods and analytical strategy per supplementary figure panel 58
11.4.1. Supplementary Fig. 1 58
11.4.2. Supplementary Fig. 2 58
11.4.3. Supplementary Fig. 3 58
11.4.4. Supplementary Fig. 4 59
11.4.5. Supplementary Fig. 5 60
11.4.6. Supplementary Fig. 6 61
11.4.7. Supplementary Fig. 7 61
11.4.8. Supplementary Fig. 8 61
11.4.9. Supplementary Fig. 9 61
11.4.10. Supplementary Fig. 10 62
11.4.11. Supplementary Fig. 11 62
11.4.12. Supplementary Fig. 12 63
11.4.13. Supplementary Fig. 13 64
11.4.14. Supplementary Fig. 14 67
11.4.15. Supplementary Fig. 15 67
11.4.16. Supplementary Fig. 16 67
11.4.17. Supplementary Fig. 17 67
11.4.18. Supplementary Fig. 18 67
11.4.19. Supplementary Fig. 19 68
11.4.20. Supplementary Fig. 20 68
11.4.21. Supplementary Fig. 21 69
11.4.22. Supplementary Fig. 22 69
11.4.23. Supplementary Fig. 23 70
11.4.24. Supplementary Fig. 24 71
11.4.25. Supplementary Tables 1 to 2 71
11.4.26. Supplementary Data 1 to 14 71

D. Supplementary Notes **72**

Supplementary Note 1. Immunologic health grades (IHGs): rationale and mitigation of confounding **72**

Supplementary Note 2. Study design: strategies to mitigate confounding **75**

Supplementary Note 3. CD8-CD4 equilibrium vs. disequilibrium and CMV serostatus **78**

Supplementary Note 4. IHG status in Ebola virus outcomes in Collaborative Cross-RIX mice **81**

Supplementary Note 5. Host genetic features that may associate with susceptibility vs. resistance to develop CD8-CD4 disequilibrium ____ **82**

Supplementary Note 6. Age-appropriate evaluations of CD4+ and CD8+ counts and the CD4:CD8 ratio: accounting for IHGs mitigates confounding ____ **83**

Supplementary Note 7. Survival rates in the Framingham Heart Study and the Vitality 90+ study ____ **85**

Supplementary Note 8. Immunologic traits that associated with IR status vs. age vs. both ____ **86**

Supplementary Note 9. Supplementary discussion - immunologic resilience program **88**

Supplementary Note 10. Strengths and limitations ____ **93**

Supplementary Note 11. Bradford Hill criteria – framework for causal inference ____ **94**

E. Supplementary Figures ____ **99**

Supplementary Fig. 1. ____ 99
Supplementary Fig. 2. ____ 100
Supplementary Fig. 3. ____ 102
Supplementary Fig. 4. ____ 103
Supplementary Fig. 5. ____ 105
Supplementary Fig. 6. ____ 106
Supplementary Fig. 7. ____ 107
Supplementary Fig. 8. ____ 108
Supplementary Fig. 9. ____ 109
Supplementary Fig. 10. ____ 110
Supplementary Fig. 11. ____ 111
Supplementary Fig. 12. ____ 112
Supplementary Fig. 13. ____ 114
Supplementary Fig. 14. ____ 116
Supplementary Fig. 15. ____ 117
Supplementary Fig. 16. ____ 118
Supplementary Fig. 17. ____ 119
Supplementary Fig. 18. ____ 120
Supplementary Fig. 19. ____ 121
Supplementary Fig. 20. ____ 122
Supplementary Fig. 21. ____ 124
Supplementary Fig. 22. ____ 125
Supplementary Fig. 23. ____ 126
Supplementary Fig. 24. ____ 128

F. Supplementary Tables ____ **130**

Supplementary Table 1. ____ 131
Supplementary Table 2. ____ 136

G. References ____ **141**

A. Disclaimer

The authors have no conflicts of interest to disclose. The views expressed are those of the authors and do not necessarily reflect the official views of the Uniformed Services University of the Health Sciences; the National Institutes of Health; the Department of Health and Human Services; Walter Reed National Military Medical Center; Brooke Army Medical Center; Madigan Army Medical Center; Naval Medical Center San Diego; Naval Medical Center Portsmouth; the Department of Defense; the Departments of the Army, Navy, or Air Force; or the Henry M. Jackson Foundation for the Advancement of Military Medicine. Mention of trade names, commercial products, or organizations does not imply endorsement by the U.S. government. The investigators have adhered to the policies for protection of human subjects as prescribed in 45 CFR 46. The U.S. government is authorized to reproduce and distribute reprints for governmental purposes notwithstanding any copyright notation thereon.

B. Acknowledgments

The two main sources of funding for the data presented herein are those awarded to SKA and JFO. SKA was supported by grants from the Veterans Affairs (VA) [VA Research Center for AIDS and HIV Infection, VA Center for Personalized Medicine (IP1 CX000875-01A1), and a VA MERIT award]; the National Institutes of Health (NIH) MERIT award (R37AI046326); the Doris Duke Distinguished Clinical Scientist Award; the Elizabeth Glaser Pediatric AIDS Foundation; the Burroughs Wellcome Clinical Scientist Award in Translational Research; and the Senior Scholar Award from the Max and Minnie Tomerlin Voelcker Fund. The work was also supported, in part, by an award jointly funded by NIAID/NIH (#AAI20042-001) and the Veterans Affairs (COVID19-8100-01) awarded to SKA and MIR. A portion of the material presented is based on research sponsored by the U.S. Air Force under agreement number FA8650-17-2-6816 (United States Air Force 59th Medical Wing Intramural Award to JFO). This study was also supported by the Infectious Disease Clinical Research Program (IDCRP), a Department of Defense program executed by the Uniformed Services University of the Health Sciences through a cooperative agreement with The Henry M. Jackson Foundation for the Advancement of Military Medicine, Inc. (HJF). The IDCRP has been funded in whole, or in part, with federal funds from the National Institute of Allergy and Infectious Diseases, NIH, under Inter-Agency Agreement (Y1-AI-5072). The SardiNIA study was supported in part by the Intramural Research Program of the NIH, National Institute on Aging, with contracts N01-AG-1-2109 and HHSN271201100005C; by Italian grants FISM 2011/R/13, FaReBio2011, Funds MIUR/CNR for Rare Diseases and Molecular Screening, CNR/DSB flagship INTEROMICS, PNR/CNR Aging Program 2012-2014; European Union's Horizon 2020 Research and Innovation Programme under grant agreement 633964; Giovani Ricercatori 2007 (D.lgs 502/92); and Legge Regionale 30 giugno 2011 n.12, articolo 3, comma 3 (to FC). The Kenya Majengo Observational (female sex worker) Cohort Study was supported by grants from the NIH (R01 AI56980), the Canadian Institutes of Health Research (HOP-43135), the Bill and Melinda Gates Foundation (39673), and the CIHR through the Grand Challenges in Global Health Initiative (to FAP). LRM was supported by a CIHR Biomedical/Clinical HIV/AIDS Research Fellowship and the International

Infectious Diseases and Global Health Training Program. The HIV- UCSD cohort was supported by National Institute of Mental Health (NIMH) P30 grant (PI: R. Heaton, MH62512), MARC from National Institute on Drug Abuse P50 grant (PI: I. Grant DA26306), and ProM from NIMH R01 grant (PI: S. Woods, MH73419). SL was supported by K24 MH097673 from the National Institute of Mental Health. The renal transplant recipient cohort and MJB were supported by grants from the Wellcome Trust (Clinical Training Fellowship) and Oxford Hospitals Research Services Committee. MJB acknowledges the support of the UK National Institute for Health Research through the Local Clinical Research Network. The HIV- Kenyan *Schistosoma haematobium* children cohort was supported by NIH grant AI064687 (CLK). The primary HIV infection cohort and DMS, SJL, and DDR were supported by NIH grants AI43638, AI074621, AI106039, and MH100974; Inter-Agency Agreement Y1-AI-5072; and the California HIV Research Program RN07-SD-702. The Sooty mangabey cohort and GS were supported by NIH grant A1 R3766998. The Chinese rhesus macaque study was supported by the National Basic Research Program of China (2012CBA01305), the Knowledge Innovation Program of CAS (KSCX2-EW-R-13), the National Natural Science Foundation of China (81172876, 81273251, U1202228), and the Key Scientific and Technological Program of China (2013ZX10001-002, 2012ZX10001-007). The CC mice study and JML were supported by NIH grants AI100625, AI096968, and AI087657. AMS was supported by the NIH T32DE014318 COSTAR institutional research training grant. KRC was supported by NIH grant T32GM113896/STXMSTP. This work was also supported by NIH grant 1UL1 TR002645 (Clinical and Translational Science Award to RAC). GCL was supported by the NIH K23-AG066933. We thank participants of the cohorts, other members of the Ahuja lab that contributed to the study, Dr. Kimberly Summers for help with study approvals, and Donna Thordsen for critical reading of the manuscript.

Framingham Heart Study dbGaP Acknowledgement Statement: The Framingham Heart Study is conducted and supported by the National Heart, Lung, and Blood Institute (NHLBI) in collaboration with Boston University (Contract No. N01-HC-25195 and HHSN268201500001I). This manuscript was not prepared in collaboration with investigators of the Framingham Heart Study and does not necessarily reflect the opinions or views of the Framingham Heart Study, Boston University, or NHLBI. Additional funding for SABRe was provided by Division of Intramural Research, NHLBI, and Center for Population Studies, NHLBI.

C. Supplementary materials and methods

Section 1. Cohorts studied

All studies were approved by the Institutional Review Boards at the University of Texas Health San Antonio and institutions participating in this study. The cohorts and study groups (**Fig. 1a**) were assembled to evaluate the hypothesis and outcomes of this study noted in **Fig. 1a** of the main text. In total, *n*=48,936 human subjects/samples, 279 non-human primates, and 378 mice were studied. The source of the cohorts/participants are summarized in **Supplementary Data 1**.

1.1. HIV-seronegative (HIV−) study groups

Baseline characteristics of all adult HIV− cohorts are listed in **Supplementary Data 2**.

1.1.1. HIV− participants from a large community sample

Cohort name: SardiNIA

Cohort description: The SardiNIA study investigates genotypic and phenotypic aging-related traits in a longitudinal manner. The main features of this project have been described in detail previously[1-3]. All residents from 4 towns (Lanusei, Arzana, Ilbono, and Elini) in a valley in Sardinia (Italy) were invited to participate. Since November 2001, a total of 6,921 participants were recruited and phenotyped, male and female, age 14 years and older. This corresponds to approximately >60% of the population eligible for recruitment in the area.

In the present study, two groups of the SardiNIA cohort were studied.

The first group (Group 1 – **Supplementary Fig. 19a**) comprised 3893 SardiNIA participants for whom we could derive the full repertoire of IHGs shown in **Fig. 2f-g**, **5c**. Age ranges were from 15 to 103 years, and 57% were females. The median CD8+ and CD4+ T-cell counts and median CD4:CD8 ratio of these individuals are reported in **Supplementary Data 3**.

The second group (Group 2 – **Supplementary Fig. 19b**) is aligned to the study by Orru et al.[2] who evaluated the genetic and immunologic traits in these participants. Results of these studies are presented in **Fig. 10**, **Supplementary Fig. 15-18**, and **Supplementary Data 12**. Thus, the SardiNIA participants studied by Orru et al. comprised 3,896 participants (age 18-102 years) in whom immune marker sets were available were included in the analyses, as reported previously[2]. 57% were females. The median (interquartile range [IQR]) CD4+ T-cell counts, CD8+ T-cell counts, and CD4:CD8 ratios in this study sample were 1,033 (806-1,291) cells/mm^3, 489 (358-670) cells/mm^3, and 2.11 (1.58-2.78) respectively. The immune markers studied are described below.

There were no differences in the IHG distributions between these two groups of SardiNIA participants, as shown in **Supplementary Fig. 19c**.

1.1.2. Female Sex Workers (FSWs) (outlined in **Supplementary Fig. 1c**)

Cohort name: The Kenya Majengo Observational Cohort Study cohort (MOCS)

Cohort description: The Majengo sex worker cohort[4] is an open cohort dedicated to better understanding the natural history of HIV infection, including defining immunologic correlates of HIV acquisition and disease progression. The MOCS cohort was established in 1985 in the Majengo area of Nairobi, Kenya. At a dedicated sex worker clinic, FSWs have access to treatment and prevention of HIV and sexually transmitted infections as well as basic health problems. Free education and condoms are provided as part of a comprehensive risk reduction program. Biannually (3- to 6-month intervals), all FSWs participate in a resurvey where repeat HIV/STI testing and CD4+ and CD8+ T-cell counts were assessed. MOCS also provides HIV-1 prevention services and ART through funding from the President's Emergency Plan for AIDS Relief. The cohort currently has more than 3,800 FSWs (pre-menopausal) and enrolls approximately 250 new members per year. These FSWs have a high risk of HIV-1 infection, and 50% of the women are seropositive at enrollment. Despite effective intervention programs, the annual incidence of HIV-1 infection among initially seronegative women is approximately 10%.

The present study comprised 1,050 initially HIV-negative FSWs with data available for analysis and were evaluated from the time they were enrolled (see criteria in **Supplementary Fig. 1c**). Of these 127 subsequently seroconverted. 762 HIV-negative FSWs had available both CD4+ count and CD4:CD8 ratio values as well as baseline risk behavior data. The characteristics of these 762 FSWs are listed in **Supplementary Data 4a**. The association of risk behavior (e.g., duration of sex work, frequency of condom use, clients per week) with prevalence of CD8-CD4 disequilibrium grades IHG-III and IHG-IV, as well as future HIV seroconversion were evaluated in these 762 FSWs. To mitigate confounding, in the main text, we show data for the 449 women who met the following criteria: had concurrent CD4+ T-cell count and CD4:CD8 ratio measurements, as well as risk behavior data and at least 2 HIV seronegative follow-up visits 3 months apart. Among these, 53 subsequently seroconverted. Prior to seroconversion, the 53 FSWs were followed for 309.31 person-years; 396 FSWs who remained HIV- were followed for 1,664.81 person-years. The characteristics of these 449 FSWs are listed in **Supplementary Data 4b**. Of the 53 FSWs who seroconverted, 43 of them had at least one CD4+ and CD8+ T-cell count measurement within 1 year of seroconversion date and the data from these participants are presented in **Fig. 4c** in the main text.

To investigate the reconstitution of IHG-I after reduction of risk behavior-associated antigenic stimulation (herein reflected by reductions in risk behavior), 101 FSWs who remained HIV− during prospective follow-up were selected who met the following criteria: (i) available at baseline both an IHG measurement as well as risk behavior activity score (BAS) computed as described below and (ii) at least 4 years of follow-up with available both IHG and BAS. The changes in IHG distributions, BAS, and other T-cell markers in

these 101 FSWs are shown in **Fig. 4b** and **Supplementary Fig. 4b-c. Supplementary Fig. 4d** shows the IHG distributions of 73 who remained HIV-free for at least 6 years. A subset of these FSWs (*n*=27) remained HIV seronegative for 10 years and reversibility in this subset is shown in **Fig. 4a** and **Supplementary Fig. 4a**.

1.1.3. Renal transplant recipient (RTR) cohort

Cohort description: To investigate the associations of IHG status with cancer development, we assessed the hazard of developing Cutaneous Squamous Cell Carcinoma (CSCC) within a predominantly Caucasian cohort of long-term renal transplant recipients (RTRs). A total of 114 RTRs with available clinical and immunological phenotype were evaluated. The characteristics of the RTRs are as described previously[5] and summarized in the **Supplementary Data 5**. Briefly, sixty-five eligible RTRs with a history of post-transplant CSCC were identified, of which 63 were approached and 59 participated. Seventy-two matched eligible RTRs without a previous history of CSCC were approached and 58 were recruited. Fifteen percent of participants received induction therapy at time of transplant, and four-fifths had received a period of dialysis prior to transplantation.

Forty of these participants developed CSCC during the study period or preceding year (as detailed previously[5] and summarized in the **Supplementary Data 5**). This subgroup was re-analyzed for CSCC-free survival (i.e., time to next CSCC during study follow-up), stratified by IHG status at enrollment (baseline).

1.1.4. Cohort of Kenyan HIV− children with *Schistosoma haematobium* (**Fig. 3a; Supplementary Fig. 2c**).

Cohort description: The Kenyan HIV− children with *S. haematobium* urinary tract infection were from a previous study[6]. Briefly, all participants were examined by ultrasound for *S. haematobium* infection and associated morbidity in the Msambweni Division of the Kwale district, southern Coast Province, Kenya, an area where *S. haematobium* is endemic. No community-based treatment for schistosomiasis had been conducted during the preceding 8 years of enrollment in this population. From this initial survey, we selected all children 5–18 years old residing in 2 villages, Vidungeni and Marigiza, who had detectable bladder pathology and *S. haematobium* infection. All participants identified with schistosomiasis were treated with praziquantel (40 mg/kg) immediately after we collected peripheral blood for immunological studies. IHG was defined by cutoff of the median CD4 percentage (36%) and CD4:CD8 cutoff of 1.0. We chose the cutoff of CD4 percentage instead of the absolute CD4 count as the latter varies significantly during maturity[7]. In the current study, 169 children prior to treatment were included. The baseline characteristics of the participants are in the **table below**.

Number of participants studied	169
Male sex, *n* (%)	69 (46.7)
Age*	12 (9 – 15)

Hematuria, *n* (%)	
0	29 (17.2)
1	28 (16.6)
2	41 (24.3)
3	71 (42.0)
Eggs, (counts/mL)*	29 (6 – 123)
CD4%*	36 (28 – 44)
CD8%*	29 (22 – 38)
IHG-III or IHG-IV, *n* (%)	64 (37.9)
* Data are median (IQR)	

1.1.5. HIV− cohort from the University of California San Diego (termed HIV− UCSD cohort)

<u>Cohort description:</u> The HIV-seronegative UCSD cohort was derived from the following three resources: (a) Those who enrolled as a normative population for ongoing studies funded by the National Institute of Mental Health; (b) Those who enrolled as a normative population for studies funded by the National Institute on Drug Abuse; (c) Those who enrolled as HIV– users of recreational drugs for studies funded by the National Institute on Drug Abuse.

In the present study, we evaluated 759 participants, pooled from the 3 abovementioned sources. The median (IQR) age of these 759 participants was 39 (30-48) years. Sixty-six percent (*n*=504) were males. The proportion of European-, African-, Hispanic-, Asian-American and others was 61% (*n*=461), 15% (*n*=112), 18% (*n*=136), 6% (*n*=45), and 1% (*n*=5), respectively. Overall, 57% (*n*=434) were CMV seropositive. In the overall cohort, median (IQR) CD4+ counts, CD8+ counts, and ratios were 917 (741-1,128) cells/mm^3, 515 (377-674) cells/mm^3 and 1.78 (1.41-2.35), respectively. The corresponding values according to IHG grades are in **Supplementary Data 3**.

Eighty-nine percent (*n*=677) of the participants had urine drug screen data available. A positive urine toxicology drug test was positive at the baseline visit for any one of the following drugs: marijuana (mj), cocaine (coc), opiates (opi), methamphetamines (meth), amphetamines (amp), barbiturates (barb), benzodiazepines (benz), and phencyclidine (pcp) (Table below). Of the 8 drugs evaluated, a total of 99 participants had at least 1 positive test. Marijuana was the most frequently used drug (Table below).

Urine drug screen test results in HIV– UCSD participants

	Any drug+	One drug+	Two drug+	Three drug+	mj+	coc+	opi+	meth+	amp+	barb+	benz+	pcp+
n	99	74	23	2	46	8	15	17	15	1	22	2
% of all	14.62	10.93	3.40	0.30	6.79	1.18	2.22	2.51	2.22	0.15	3.25	0.30
% drug+	100.00	74.75	23.23	2.02	46.46	8.08	15.15	17.17	15.15	1.01	22.22	2.02

*Of the 759 participants studied, 677 had urine toxicology drug test data available. +, positive urine drug test

1.1.6. HIV− University of Texas Health Science Center, San Antonio (UTHSCSA)

Cohort description: Three HIV− adults accrued at the UTHSCSA to serve as controls for transcriptomic (genomic) trait analyses.

1.1.7. South Texas Veterans Health Care System COVID-19 cohort

Cohort description: This was a prospective observational cohort study of patients testing positive for SARS-CoV-2 evaluated at the Audie L. Murphy VA Medical Center, South Texas Veterans Health Care System (STVHCS), San Antonio, Texas from March 20, 2020 through November 15, 2020. Patients were followed during hospitalization and/or a minimum of 30 days from inclusion. The overall COVID-19 cohort comprised of 541 HIV− patients. The cohort characteristics and samples procedures are described in **Supplementary Data 2 and Supplementary Data 7**. The cohort features of a smaller subset of patients studied herein and samples procedures have been previously described[8]. COVID-19 progression along the severity continuum was characterized by hospitalization and death. Standard laboratory methods in the Flow Cytometry Core of the Central Pathology Laboratory at the Audie L. Murphy VA Medical Center were used to determine peripheral blood CD4+ and CD8+ T-cell levels. The overview of this cohort is shown in **Supplementary Fig. 1d**. All measurements evaluated in the present study were prior to the availability of COVID-19 vaccinations.

1.1.7.a. COVID-19 study groups: Hospitalized vs. non-hospitalized

To define the patient characteristics of those who manifest COVID-19 progression vs. non-progression, both hospitalized and non-hospitalized COVID-19 patients were evaluated. Hospitalized (H) patients were those who were hospitalized for at least 1 day; non-hospitalized (NH) patients were those testing positive for SARS-CoV-2 in outpatient clinics or the emergency department who were enrolled in the STVHCS home COVID-19 telehealth program. Hospitalized patients who were discharged home were enrolled in a Hospital-in-Home (HIH) program for a minimum of 48 hours from inpatient discharge.

1.1.7.b. Convalescence subset of the COVID-19 cohort

A subset of patients discharged from the hospital and outpatient program (home telehealth program during acute disease) who subsequently enrolled in the STVHCS COVID-19 convalescence monitoring program were evaluated. The convalescence program comprised of monthly immune profile assessments (e.g., CD4+ and CD8+ T-cells) and visits from multi-specialties.

1.2. HIV-seropositive (HIV+) cohorts

Participant characteristics at entry in all HIV+ cohorts are listed in **Supplementary Data 2 and Supplementary Data 6**.

1.2.1 Primary HIV infection cohort

Cohort: Primary Infection cohort (PIC) from University of California, San Diego (designated as PIC in the main text; outlined in **Supplementary Fig. 1a**).

Cohort description: The PIC cohort comprised 723 HIV+ participants[9]. These participants were recruited between June 1996 and June 2010 and then followed prospectively. Details of the cohort were as described previously[9]. We evaluated only those participants in whom an estimated date of infection could be calculated through a series of well-defined stepwise rules that characterize stages of infection based on our previously described serologic and virologic criteria[9]. Of the 723 participants, 685 were evaluated in the present study while they were therapy-naïve (see criteria in **Supplementary Fig. 1a; Supplementary Data 6**). 194 who commenced ART between 4/26/1997 and 4/26/2013 (with clinical data collected until 10/13/2014) and met other inclusion criteria were also evaluated (**Supplementary Data 6**). The inclusion criteria are outlined in **Supplementary Fig. 1a**. Participants in the cohort self-selected as to whether or not to initiate ART, and those who chose not to start therapy were followed in a manner identical to those who chose to start ART. Rules of computing time to estimated date of infection are as reported by us previously[9]. 75 of the 194 were on ART for at least four ears and had at least one IHG measurement in each of those four years.

1.2.2. Early infection cohort (EIC)

Cohort: US Military HIV Natural History Study designated as the early infection cohort (EIC) in the main text (outlined in **Supplementary Fig. 1b**)

Cohort description: The US Military HIV Natural History Study is an ongoing, continuous-enrollment, prospective, multicenter, observational cohort study conducted through the Uniformed Services University of the Health Sciences Infectious Disease Clinical Research Program. The EIC has enrolled approximately 5,723 active-duty military service members and beneficiaries since 1986 at 7 military treatment facilities (MTFs) throughout the United States. The US military medical system provides comprehensive HIV education, care, and treatment, including the provision of ART and regular visits with clinicians with expertise in HIV medicine at MTFs, at no cost to the patient. Mandatory periodic HIV screening according to Department of Defense policy allowed treatment initiation to be considered at an early stage of infection before it was recommended practice. Eighty-eight percent of the participants since 1995 have documented seroconversion (i.e., a documented negative HIV test preceding a positive HIV test), with a median seroconversion window of approximately 15 months. The median CD4+ T-cell count at diagnosis was approximately 500 cells/mm^3.

Active-duty personnel are required to visit an MTF at least twice yearly for formal medical evaluation. Following retirement or separation from active duty, all participants retain health benefits and may continue participation in the cohort study while receiving their primary HIV care either within or outside of the military health care system. Aside from the advantages afforded by the medical system, there are aspects of this cohort that allow

for a unique perspective on HIV treatment response. The military population from which these patients are derived consists of highly motivated and disciplined participants who possess either a minimum of a high school equivalent education (enlisted) or an undergraduate college degree (officers) and maintain rigorous physical standards. Through periodic random drug screening, the reported rate of injection drug use in this population is less than 1%. Thus, many factors that typically hinder the clinical response to ART in most North American cohorts are minimized or eliminated in the military setting. Additionally, the cohort is racially balanced and geographically diverse, reflecting the distribution of participants with HIV in the United States.

In the present study, 4,883 of 5,723 EIC participants were available for evaluation (**Supplementary Fig. 1b; Supplementary Data 6**).

Additional details of the SardiNIA[1-3], FSW-MOCS[4], PIC-UCSD[9], RTR cohort[5], *S. haematobium* infected children cohort[6], and EIC[10-14] have been described previously. Some features of the entire or subsets of the SardiNIA, COVID-19, SLE (Supplementary Information Section 8.3.1), Framingham Heart Study (Supplementary Information Section 8.3.2), San Antonio Family Heart Study (Supplementary Information Section 8.3.3), and HIV cohorts studied herein have been described previously by us in a recent study[8].

1.3. Non-human primates

1.3.1 Sooty mangabeys

One hundred sixty sooty mangabeys were evaluated in the current study. Of these, 50 were SIV seronegative (SIV–) and 110 were naturally infected with SIV (**Fig. 4c,d; Fig. 9d**). Data from a subset of these sooty mangabeys has been reported previously by Sumpter et al.[15]. All sooty mangabeys were housed at the Yerkes National Primate Research Center and maintained in accordance with National Institutes of Health guidelines. In uninfected animals, negative SIV determined by PCR in plasma confirmed the absence of SIV infection. Based on longitudinal serologic surveys, the majority of SIV+ sooty mangabeys are known to have acquired their infection by 3 to 4 years of age. IL-7R (CD127) levels of CD8+ T-cells as well as CD8+ effector T-cells were assessed as the proportion of CD127+CD8+ T-cells and CD28–CD95+CD8+ T-cells, respectively (**Fig. 9d**). Other immune traits studied are reported in **Supplementary Data 11**. Flow cytometry was performed as described previously[15]. The baseline characteristics of sooty mangabeys are listed in the table below. Note: Of the 50 SIV– sooty mangabeys, age and sex data are only available in 47 animals. The immune traits of the SIV– and SIV+ sooty mangabeys were measured on two separate experimental days. We used same CD4+ and CD4:CD8 T-cell ratio cutoffs as in humans to derive IHGs (**Fig. 2b**, main text; Ref.[8]).

Characteristics	*n*#	SIV–	*n*#	SIV+	*P* values*
No. of animals studied		50		110	
Male sex, *n* (%)	47	20 (43.5)	110	52 (47.3)	0.712
Age at experiment, years	47	10 (6-13)	110	13 (10–16)	0.001

CD4+ T-cell counts at experiment, cells/mm^3	50	883 (565-1268)	110	987 (669–1296)	0.174
CD8+ T-cell counts at experiment, cells/mm^3	50	784 (640–1214)	110	1478 (948–2064)	<0.001
CD4:CD8 ratio at experiment	50	1.10 (0.67–1.48)	110	0.71 (0.48–1.09)	0.006
SIV VL, log_{10} copies/mL	50	-	110	5.05 (4.64-5.30)	-
Unless otherwise specified all data are median (interquartile range). VL, plasma SIV RNA viral load.<br># no. of animals on which data are available.<br>* *P* values determined by χ^2 test for sex and Wilcoxon rank-sum test for all other variables. Two-sided tests were used. *P* values are in Supplementary Data 14.					

1.3.2. Chinese rhesus macaques

Forty-seven male and 40 female SIV– Chinese rhesus macaques from a previous study were evaluated (**Fig. 4e**)[16]. All animals were colony-bred rhesus macaques (*M. mulatta*) of Chinese origin. All animals were without overt symptoms of disease (tumors, trauma, acute infection, or wasting disease); estrous, pregnant, and lactational macaques were excluded. A marker of T-cell dysfunction was evaluated, which was expression levels of PD-1 on CD8+ T-cells (**Fig. 9e; Supplementary Fig. 14**), using methods described previously[16]. The characteristics of these animals are shown in the table below. We used same CD4+ and CD4:CD8 T-cell ratio cutoffs as in humans to derive IHGs (**Fig. 2b**, main text; Ref.[8]).

Age groups	Males		Females	
	n	Age (yrs)*	n	Age (yrs)*
Juvenile (2-3 yrs)	9	2.3 ± 0.5	8	2.5 ± 0.5
Adult (5-12 yrs)	17	7.7 ± 2.1	17	9.2 ± 2.2
Aged (15-24 yrs)	21	19.3 ± 3.3	15	19.5 ± 3.0
Total	47	11.9 ± 7.5	40	11.8 ± 7.0
*The data are mean ± SD.				

1.4. Collaborative Cross mice (CC-RIX mice – Ebola Infection)

		Median values		
IHG	n	CD4+ cells/mm^3	CD8+ cells/mm^3	Ratio
I	162	5807742	3244964	1.77
II	128	3018513	1580441	1.71
III	5	4994856	5915567	0.85
IV	39	1870888	2928236	0.79

In a previous study by Rasmussen et al.[17], the role of mice genetics in Ebola virus disease was studied using the Collaborative Cross (CC) resource[17]. CC is a genetically diverse panel of recombinant inbred (CC-RI) mice obtained through a systematic cross of eight inbred founder mouse strains, five of which are classic laboratory strains (C57BL/6J, A/J, 129S1/SvImJ, NOD/ShiLtJ, and NZO/H1LtJ) and three of which are wild-derived inbred strains (CAST/EiJ, PWK/PhJ, and WSB/EiJ). Different strains were crossed with one another to generate CC-RI intercrossed (CC-RIX) F1 progeny. The authors reported that the CX-RIX mice exhibited distinct disease phenotypes after mouse-

adapted Ebola virus infection and the phenotypes ranged from complete resistance (0% mortality) to lethal disease (>50% mortality).

Our collaborators at CC resource collected total counts of CD4+ T-cells and CD8+ T-cells from the spleen of mock CC-RIX mice at two different timepoints (12 and 28 days). The median CD4+ and CD8+ T-cell counts in CC-RIX mice (*n*=334) were 4,025,822 and 2,510,971 cells/mm^3, respectively. A CD4:CD8 ratio >1.0 and total CD4+ counts > median CD4+ (4,025,822 cells/mm^3) counts were used as cutoffs to compute the IHG groups similar to groupings in **Fig. 2b** of the main text. The corresponding median CD4+ and CD8+ T-cell counts and CD4:CD8 ratio in the IHG groups from CC-RIX mice (*n*=334) are listed in the accompanying table and the overall distribution of IHGs is shown in the **adjacent figure**.

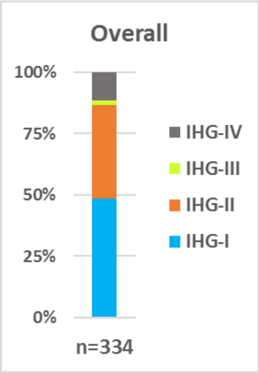

We screened mock CC-RIX mice with IHG data for CC-RIX strains with reported[17] Ebola-disease phenotype data and identified 99 mice with both data. (Note: IHGs were not derived from the Ebola-infection study). We overlaid the Ebola-disease phenotype data of CC-RIX strains to IHGs from mock CC-RIX mice to generate the distribution of IHGs by Ebola-disease phenotype using percentage stacked barplots reported in **Fig. 4f** and **Supplementary Note 4**.

Section 2: Study definitions, hypotheses, and statistical approach

2.1. Definitions.

(i) Study entry (baseline) was defined as the date when the first CD4+ and CD8+ T-cell counts were available after HIV diagnosis.

(ii) The IHG grade achieved during ART was based on the highest CD4+ T-cell count and the concomitantly assessed CD4:CD8 ratio during the duration of ART follow up.

(iii) Viral load suppression was defined previously[9,10] and was 2 or more consecutive viral load measurements of less than 50 copies/mL (in the PIC) at least 14 days apart.

(iv) The estimated date of infection (EDI) in PIC participants was computed as described previously.[9]

(v) Time of progression to AIDS was computed in participants from the EIC as described previously[10]. The 1993 CDC criteria for AIDS were used[18]. For these analyses, patients were right-censored if they initiated virally suppressive ART, died, or were lost to follow-up. Participants with an HIV diagnosis date on or prior to entry were excluded.

(vi) In a previous study[9], we had conducted a review of studies reported in the PubMed in which CD4+ T-cell counts had been surveyed in confirmed or presumed HIV− individuals of European descent and in African Americans. We updated this survey and included the survey of the CD4:CD8 T-cell ratio. The

results of this updated survey are reported in **Supplementary Table 1.** Because of concerns that differences in nutritional standards and parasitic infections may impact lymphocyte levels, in this survey we had excluded studies that had surveyed other populations.

(vii) ART initiation date was defined as the first documented antiretroviral therapy (ART) initiation date in the PIC-UCSD cohort.

2.2. Overarching hypothesis, cohorts, and comparisons

Our hypothesis is based on the concept that in response to environmental stressors, including infections, individuals may manifest resilience (ability to recover after deviation from an optimal level of immunity) and/or robustness (ability to resist such deviation). Without intensive prospective monitoring, it is challenging to distinguish between resilience vs. robustness. We therefore combined these two concepts under the umbrella of the definition of immunologic resilience (**IR**). We previously defined optimal IR as the capacity to preserve and/or restore immunocompetence and suppress inflammation during acute, chronic, or repetitive antigenic exposures experienced across the lifespan, including SARS-CoV-2 infection. Using laboratory (immune health grades; IHGs) and transcriptomic metrics (SAS-1, MAS-1), we established a benchmark of optimal IR. We tested the overarching hypothesis that deviations from optimal IR predicted inferior immunity-dependent health outcomes in settings of acute (SARS-CoV-2) and chronic (HIV) infections, as well as settings of repetitive antigenic stimulation, such as sex work and aging.

2.3. Rationale for studying distinct cohorts and the comparisons made

1. While we articulated an omnibus or overarching hypothesis, this hypothesis has distinct facets depending on the clinical or biological context.
2. We tested specific hypotheses relevant to IR within distinct clinical or biological contexts. Each of these contexts has no association with any context other than environmental/antigenic stress.
3. It was not possible to study a single cohort or group of individuals to test whether deviations from optimal IR influence each of the outcomes (e.g., HIV acquisition, COVID-19 severity, lifespan, AIDS).
4. For these reasons, we examined IR metrics in distinct cohorts that permitted us to address specific IR-related questions that are aligned with the overarching hypothesis.
5. The impetus to evaluate multiple cohorts depicting the same issue (e.g., erosion of IR during aging; resistance to IR erosion associates with influenza resistance) was for purposes of replication and to define inter-cohort variability.
6. Although there may be conceptual overlaps, each cohort provided a unique biological context. While the broad principles of IR may apply to sex workers, HIV, influenza, and COVID-19, the biological underpinnings by which deviations from optimal IR may associate with inferior immunity-dependent outcomes are distinct. For example:

- Erosion of IR attributable to HIV risk factor-associated antigenic stimulation results in an increase in the number of sex workers with IHG-III and IHG-IV. This may be attributable to the high rate of CMV seropositivity in the cohort.
- Erosion of IR during COVID-19 results an increase in the proportion of patients with IHG-II vs. IHG-IV. IHG-III is infrequent in COVID-19 or aging.
- During COVID-19, IHG-II emerges more frequently in patients with CMV seronegativity, whereas IHG-IV emerges more frequently in patients with CMV seropositivity.
- For these reasons, to mitigate the confounding effects of CMV serostatus, where appropriate we performed comparisons in CMV seropositive vs. seronegative persons.

7. We performed comparisons to account for the effects of age and sex, as antigenic stimulation during aging is associated with erosion of IR, and females preserve optimal IR to a greater extent than men. In our analyses we (i) adjusted for age and/or sex, or (ii) performed overall comparisons, followed by stratification by age strata and sex.
8. Additionally, we could not apply the same IR metric or strata of a metric across each of the cohorts. For some analyses, we used IHGs and in others we used transcriptomic metrics of IR (SAS-1/MAS-1 profiles). An important aspect of this study was to validate SAS-1/MAS-1 profiles as transcriptomic proxies for IHGs. This was important, as while gene expression studies are commonplace, assessments of CD4+ and CD8+ T-cell counts required for derivations of IHGs are uncommon in most cohort-based studies.
9. Additionally, we define mechanisms by which IR status influences immunity-dependent outcomes. Each mechanistic cohort lends itself to distinct questions and analyses.
10. SUMMARY: For the above-noted reasons, it is not possible to apply an omnibus statistical test across these varied cohorts. Each cohort was distinct, and each comparison addressed a specific question using the best statistical practices and accounted for multiple comparisons and relevant variables that may influence results (e.g., age, sex, CMV serostatus).

2.4. Biological plausibility and statistical approach

A key goal of our study was to examine the biological plausibility of our overarching hypothesis wherein the general principles of IR are applicable to distinct biological/clinical contexts studied in distinct cohorts. Thus, it was to be anticipated that IR metrics will show statistically significant differences in varied cohorts.

Statistical approach:

1. The focus of our statistical design was to determine whether the directions and magnitudes of differences (perhaps including some trending with $P>0.05$ and <0.10) fit a biologically coherent pattern vs. chance findings.
2. If only one or very few measures reached statistical significance and their directions and/or magnitudes did not coherently fit with our hypothesis that IR was

associated with superior immunity-dependent outcomes, our plan was to note that result(s) significant at $P<0.05$ lacked biological plausibility and could be due to chance despite meeting the conventional cutoff for statistical significance.

3. With recognition of points 1 and 2, our approach to multiple comparisons adheres to best practices. Depending on the question asked and nature of the dataset, we provide (i) an overall omnibus test (e.g., ANOVA), (ii) FDR or Bonferroni corrections, and (iii) nominal *P* values, without adjustment for multiple testing. Such adjustment would be focused on avoidance of one or more results with $P<0.05$ in the case where all differences are truly zero[19-21], which is an extremely unrealistic possibility about the association between IR status and immunity-dependent outcomes. In addition, adjustment would require that each result detract from the others, but there are clear biological relationships among many of the issues that we examine, and these permit coherent sets of findings to reinforce each other rather than detract from one another. Thus, multiple comparison adjustment would be an incorrect approach in such cases[22]. Hence, when reporting nominal *P* values, we relied on scientific judgment regarding the biological plausibility of our findings in the context of our overall findings, rather than formal adjustment methods to indicate where caution is warranted despite findings with significance at $P<0.05$. Most importantly, in all cases where we reported nominal *P* values, it is self-evident from the smaller *P* values ($P<0.001$) that formal adjustments for multiple comparisons would not change the interpretation of our findings.
4. Thus, we individualized the analysis of each dataset, adhering to these best practices:
   a) We based interpretations on a synthesis of statistic results with scientific considerations.
   b) We relied on scientific considerations to guard against overinterpretation of findings with $P<0.05$.
   c) We acknowledged the desirability of independent replications, particularly for unexpected findings (e.g., IR response was associated with symptom responsiveness to viral infections).
   d) Where appropriate we determined group level differences followed by post-hoc testing.
   e) The assumptions of each statistical test were evaluated to ensure that associated proposed analyses were appropriate (e.g., normality, homogeneity, linearity, and independence).
   f) We provide estimates with confidence intervals
   g) We chose accuracy, scientific judgement, and biological plausibility balanced by appropriate statistical testing for multiple comparisons. We are mindful that conventions for statistical analysis and interpretation have emerged from a formal statistical hypothesis testing paradigm, guarding against chance false-positive results by application of multiple comparisons adjustments. However, these paradigms are inherently focused only on *P* values, promoting use of the *P* value fallacy. These adjustments also have the unfortunate property that the results of each analysis are automatically assumed to detract from all the others, with no consideration of how well the different results fit together conceptually or scientifically[21,22]. This general approach has been criticized as

unreliable and contrary to the original statistical theories that supposedly support it[23-27], but it remains engrained in research culture[28,29].

h) Taken together, our overall statistical approach balances the considerations in points a) to g).

Section 3. Rationale for CD4+ T-cell count and CD4:CD8 ratio cutoffs used to define IHG grades

We used preselected cutoffs for the CD4+ T-cell count and CD4:CD8 T-cell ratio to co-index these measures to derive the IHGs (**Fig. 2b**, main text**;** Ref.[8]).

3.1. Pre-hoc selection of CD4+ cutoffs of 800 cells/mm^3 for IHGs

As a point of reference, we had used this cut-off in 2 previous studies[9,10]. We previously reported that CD4+ T-cell count of approximately 800 cells/mm^3 approximated the lower bounds of the median CD4+ T-cell count in >12,000 HIV uninfected participants[9,10]; this survey was conducted by a Medline search. On this basis, we had previously used CD4+ 800 cells/mm^3 as an outcome during antiretroviral therapy (ART) of HIV+ persons[9]. Additionally, we found that attainment of CD4+ counts equal to or above 800 cells/mm^3 associated with restoration of markers of T-cell health to levels observed in HIV-seronegative persons[10].

3.2. Pre-hoc selection of cutoffs for the CD4:CD8 ratio of 1.0 for IHGs

Most HIV-seronegative individuals maintain a CD4:CD8 T-cell ratio of ≥1.0[30,31], sometimes even in conditions associated with CD4+ lymphocytopenia (e.g., infections other than HIV-1, malignancy)[32]. In sharp contrast, a universal feature of untreated chronic HIV infection is the inversion of the CD4:CD8 T-cell ratio (<1.0)[33-35], and an inverted ratio in HIV-seronegative participants correlates with adverse events[33,36-43].

3.3. Additional considerations

We co-indexed CD4:CD8 ratio and CD4+ T-cell counts as we considered them to be controlled through independent mechanisms and hence only partly related. Two observations substantiated these viewpoints. First, a genome-wide association study revealed that, while the gene variants that influenced CD4+ T-cell counts and the ratio mapped to the MHC locus, the loci to which they mapped were distinct[30]. Moreover, the variant that influenced CD8+ T-cell counts overlapped with the variant that influenced the ratio. Second, we found that the explained variability (r^2) of the CD4:CD8 ratio by the concomitantly measured CD4+ T-cell count was low as noted in the table below; values ranged between 7% and 9% for the HIV-seronegative participants and between 10% and 33% in ART-naïve HIV-seropositive participants and higher explained variability in HIV+ persons receiving ART during primary infection.

Explained variability (r^2) of the ratio by the concomitantly measured CD4+ T-cell counts					
HIV status	Cohort	*n*	Entry	Pre-ART	During-ART
HIV−	SardiNIA	3,896	0.09		
	MOCS (Female sex workers)	1,050	0.07		
HIV+	EIC: Therapy-naïve	4,833	0.33		
	PIC-UCSD: Therapy-naïve	685	0.16		
	PIC-UCSD: Received ART	194	0.10*	0.24*	0.15#
*The explained variability computed during therapy naïve status at entry and at pre-ART in HIV+ participants.<br>#The explained variability computed with the best CD4+ T-cell counts during ART and the accompanying CD4:CD8 ratio.					

3.4. Cardinal features of IHG-III or IHG-IV (ratio <1.0)

To identify inducers and clinical outcomes associated with CD8-CD4 disequilibrium grades IHG-III or IHG-IV, we surveyed IHGs in our HIV− cohorts and performed an extensive literature review (detailed in **Fig. 5c**, **Supplementary Table 2**). We used an inverted CD4:CD8 ratio as a proxy for the presence of IHG-III and IHG-IV in the review as in many literatures the stratification of IHGs is not available.

Section 4. Methods for associations in HIV− FSWs and COVID-19

4.1. Behavioral activity score (BAS)

To provide a summated metric of the risk behaviors in FSWs, a BAS was derived using four behavioral risk factors.

(i) Duration of sex work categorized as 1-5, 6-10, and ≥11 years

(ii) Condom use frequency was recorded as: 1: never, 2: <50% of the time, 3: >50% of the time, 4: always

(iii) Clients per week were stratified by the median and quartiles in the 702 FSWs and were <16, 16-25, 26-35, ≥36 clients per week.

(iv) A measure of unprotected behavioral risk was generated, defined as the difference between the number of clients per week and the number of condoms used per week. A negative number indicates less unprotected exposure (i.e., more condoms use than clients), while a positive number represents greater unprotected exposure (i.e., less condom use relative to clients). Unprotected behavioral risk was categorized as <0, 0, 1-5, ≥6.

We observed that duration of sex work did not associate with baseline prevalence rates of CD8-CD4 disequilibrium (IHG-III or IHG-IV) in FSWs whereas less condom use, more clients per week, and greater unprotected behavioral risk associated with higher rates of CD8-CD4 disequilibrium grades IHG-III or IHG-IV. To summate the latter 3 risk activities, we derived a BAS that accounted for condom use, clients per week and unprotected behavioral risk as follows:

- Condom use frequencies of 1, 2, 3, 4 were coded as -1, -2, -3, -4, respectively
- Clients per week of ≤15, 16-25, 26-35, ≥36 were coded as 1, 2, 3, 4, respectively
- Unprotected behavioral risk of <0, 0, 1-5, ≥6 were coded as -1, 0, 1, 2, respectively

The summated score ranged from -4 to 5, indicating the least to the highest BAS. As the frequency of FSWs with scores of 4 and 5 were infrequent, we assigned scores of 3 and above as 3+. In the analysis shown in the main text (**Fig. 5a-b**), we trichotomized risk scores as <0, 0, >0 to signify low, moderate, and high BAS, respectively.

4.2. Sexually transmitted infections (STI) scores

We evaluated the 7 STI related factors that were recorded as categorical variables. The total STI scores were derived from 5 indirect and 2 direct STI scores. The theoretical minimal and maximal STI score is from zero to 7. The factors comprising the indirect and direct STI scores noted in the table below.

Indirect STI score	Direct STI score	Total STI score
• Vaginal Discharge<br>• Abdominal Pain<br>• Genital Ulcer<br>• Dysuria<br>• Vulva Itch	Rapid plasma regain (RPR test for syphilis)<br>• Gonorrhea	• Vaginal Discharge<br>• Abdominal Pain<br>• Genital Ulcer<br>• Dysuria<br>• Vulva Itch<br>• RPR (Syphilis)<br>• Gonorrhea

Predictably, the BAS was highly correlated with STI scores. For example, among the 449 FSWs, both BAS and STI scores could be computed in 405 participants. The correlation r (*P* values) between BAS with indirect, direct, and total STI scores were 0.21 (*P*<0.001), 0.14 (*P*=0.005) and 0.22 (*P*<0.001), respectively (**Supplementary Fig. 3c**).

4.3. COVID-19 WHO ordinal scale

COVID-19 patients' respiratory status was indexed to the World Health Organization (WHO) 8-point ordinal scale[44] while hospitalized and at every encounter.

The pointwise categories of the WHO ordinal scale are as follows:

1. not hospitalized and no limitations of activities
2. not hospitalized, with limitation of activities, home oxygen requirement, or both
3. hospitalized, not requiring supplemental oxygen and no longer requiring ongoing medical care (used if hospitalization was extended for nonmedical reasons)
4. hospitalized, not requiring supplemental oxygen but requiring ongoing medical care
5. hospitalized, requiring any supplemental oxygen
6. hospitalized, requiring noninvasive ventilation or use of high-flow oxygen devices

7. hospitalized, receiving invasive mechanical ventilation (MV) or extracorporeal membrane oxygenation (ECMO)
8. death

The COVID-19 disease severity was defined as mild, moderate, and severe using the WHO ordinal scale values of 1-4, 5, and 6-8 respectively and their association with IHG status is presented in **Fig. 3f**.

Section 5. Immunophenotyping methods used for assessment of immune correlates that associated with IHG status vs. age in the SardiNIA cohort

Immune markers were assessed on fresh blood samples. Cells were processed within 2 hours after sample collection to avoid time-dependent artefacts. A set of multiplexed fluorescent surface antibodies were used to characterize the major leukocyte cell populations circulating in peripheral blood belonging to both adaptive and innate immunity. Briefly, with the antibody panel designated as T-B-NK in **Supplementary Data 12**, we identified NK, B, and T-cells and their subsets. We also used the HLA-DR marker to assess the activation status of T and NK cells. The regulatory T-cell panel (Treg in **Supplementary Data 12**) was used to characterize regulatory T-cells subdivided into resting, activated, and secreting nonsuppressive cells[45,46]. Moreover, in selected T-cell subpopulations, we assessed the positivity for the ectoenzyme CD39 and the CD28 co-stimulatory antigen[47]. The antibody panel named T-cell maturation (Mat in **Supplementary Data 12**) accounted for the chemokine receptor CCR7 and the CD45RA marker to distinguish between naïve, central memory (CM), effector memory (EM), and terminally differentiated (TD) subsets in CD4+ and CD8^{bright} and CD4–CD8– T-cells[48]. Finally, by the circulating dendritic cells (DC) panel we divided DCs into myeloid (conventional DCs, cDCs) and plasmacytoid (pDCs) cells and assessed the expression of the adhesion molecule CD62L and the co-stimulatory ligand CD86[49,50]. The cDC panel was labelled DC in **Supplementary Data 12**. Overall, through this process we measured 75 distinct, non-overlapping, informative, immune traits/markers (**Supplementary Data 12**). Detailed protocols and reproducibility of the measurements have been described[2].

Leukocytes were characterized on whole blood by polychromatic flow cytometry with 4 antibody panels, namely T-B-NK, regulatory T-cells (Treg), maturation stages of T-cells (Mat), and circulating dendritic cells (DC), as described elsewhere[2]. Brief descriptions follow.

i) *T-B-NK* panel (**Supplementary Fig. 15**). Total leukocytes (CD45+) were divided into lymphocytes, CD14+ monocytes, and granulocytes. Lymphocytes were separated based on CD3 expression. CD3+ cells, i.e., T lymphocytes, were further split into 6 subsets based on expression of the CD4 and CD8 markers: CD4– CD8–, CD4– CD8dim (CD8dim), CD4– CD8^{bright} (CD8br), CD4+ CD8br, CD4+ CD8dim, CD4+ CD8– (CD4+). CD8+ corresponds to the summation of CD8bright and CD8dim cells. T-cells expressing γδ heterodimer was also identified. CD3– lymphocytes were divided into B-cells (CD19+) and natural killer cells (CD16+ and/or CD56+). T-cells (including CD4+ and CD8bright)

and NK activation was detected by the HLA-DR marker.

ii) *Regulatory T-cell* (Treg) panel (**Supplementary Fig. 16**). CD4+ regulatory T-cells (Tregs) were identified according to their high expression of CD25 and low expression of CD127 markers. Tregs were subdivided into 3 subsets: activated (CD25+++ CD45RA–), resting (CD25++ CD45RA+), and secreting (CD25++ CD45RA–). We also considered CD4+ T-cells expressing elevated levels of CD25 and subtracting the Treg cells from them (CD25hi CD4+ not Tregs). The resulting population was further divided based on CD45RA expression. Moreover, we assessed the CD8+ T-cells considering their expression of CD25, CD28, CD127, and CD45RA antigens. Finally, CD39 expression was assessed on the Treg subsets, total CD4, and CD8 T lymphocytes.

iii) *Maturation stages of T-cell* (Mat) panel (**Supplementary Fig. 17**). The maturation phases of CD4+, CD8br and CD4–CD8– T lymphocytes was evaluated according to expression of the CD45RA and CCR7 antigens[48]. In each subset, we analyzed naïve (CD45RA+ CCR7+), CM (CCR7+ CD45RA–), EM (CD45RA– CCR7–) and TD (CCR7– CD45RA+) stages.

iv) *Circulating Dendritic Cells* (DC) panel (**Supplementary Fig. 18**). Circulating DCs were identified based on their brightness for the HLA-DR surface molecule and their negativity for the Lineage cocktail (Lin) targeting CD3, CD14, CD16, CD19, CD20, and CD56. The circulating DCs were split into myeloid (conventional DC, cDC) and plasmacytoid (pDC) cell types based on their positivity for CD11c and CD123 antigens, respectively. The circulating DC maturation status was determined using the adhesion molecule CD62L and the co-stimulatory molecules CD86. Furthermore, the monocyte population was morphologically taken and analyzed for HLA-DR, CD62L, and CD11c expression.

Section 6. Assessment of the correlation between indicators of T-cell responsiveness, T-cell dysfunction, and systemic inflammation and expression levels of SAS-1 and MAS-1

6.1. Indicators of T-cell responsiveness, T-cell dysfunction, and systemic inflammation

These indicators were evaluated in a subset of EIC participants whose characteristics were described previously[10,51].

Participant characteristics were:

(i) ART-naïve controls (*n*=28)
(ii) virally suppressed on long-term ART (*n*=124)
(iii) HIV-uninfected controls (*n*=13); as described in Supplementary Information Section 1.1.6.

Correlates analyzed were as follows using previously described methods [10,51].

Integrity of the IL-7/IL-7 receptor axis (T-cell responsiveness) was investigated by determining level of responsiveness of T-cells assessed as the proportion of T-cells responding to IL-7, based on the percentage of CD3+ T-cells positive for phosphorylated signal transducer and activator of transcription (STAT5) (%CD3^{+}pSTAT5^{+} T-cells) after *in vitro* stimulation of peripheral blood mononuclear cells with IL-7. IL-7 is a critical T-cell trophic cytokine. Methods were as described previously[10,51].

Levels of T-cell exhaustion (dysfunction) were assessed as proportion of CD4+ T-cells positive for programmed cell death protein-1 (PD1) (%CD4+PD1+ T-cells) (proxy for exhaustion).

Systemic inflammation was assessed by measuring plasma IL-6 levels using Luminex assays, employing methods described by the manufacturer.

To link the gene expression to functional response, in the same biological sample from virally-suppressed HIV+ patients (*n*=56, Supplementary Information Section 7.2), we determined the indicators (noted above) and gene expression in PBMCs. Then, we assessed the correlations between these indicators and expression of the genes in the SAS-1, MAS-1, and IMM-AGE gene signatures (figure below). The derivation of these signatures is described in Supplementary Information Sections 8.1 – 8.2. Data are shown in **Fig. 9c** and **Supplementary Data 10**.

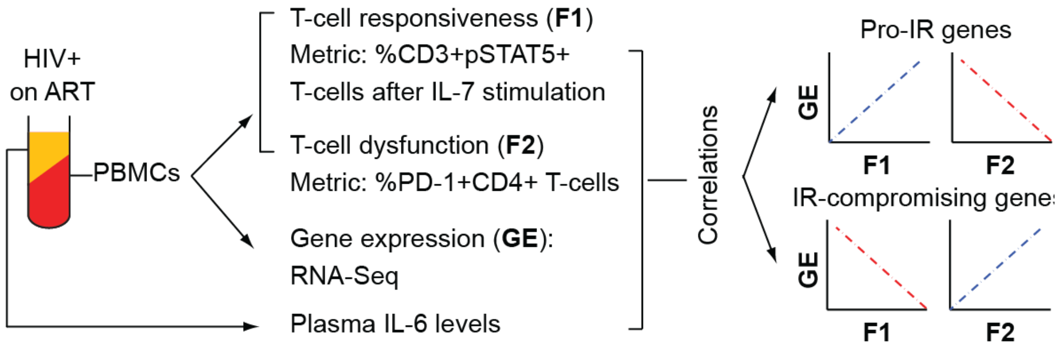

Section 7. RNA-seq in HIV+ persons and health controls

7.1. Methods

Whole genome RNA sequencing (RNA-seq) was used to determine the expression levels of genes in PBMCs. Methods and bioinformatics analysis were similar to those reported previously[52,53] and summarized below.

7.2. Study groups

RNA-seq analysis was performed in the following groups. Except for the HIV-seronegative participants, all participants were from the EIC (**Supplementary Fig. 1b**).

(i) 56 HIV-infected patients on virally suppressive ART from EIC; *in vitro* immunological correlates (T-cell responsiveness, exhaustion, plasma IL-6)

were also evaluated in these patients (i.e., gene expression and three immunological correlates were assessed in the same biological sample (Supplementary Information Section 6)).

(ii) 10 HIV spontaneous virologic controllers (SVC) from San Antonio, VA Medical Center

(iii) 3 HIV-infected ART-naïve participants from EIC

(iv) 3 HIV-seronegative controls.

7.3. RNA isolation

Total RNA was isolated from PBMCs using the RNeasy Mini Kit (Qiagen, Hilden, Germany) with DNase I digestion according to the manufacturer's instructions. RNA quantity and purity were determined by spectrophotometry (260/280=1.8-2.0) (Nanodrop), and integrity (RIN) was determined using an Agilent 2100 Bioanalyzer with an RNA 6000 Nano assay (Agilent Technologies, Palo Alto, CA). Samples with RIN ≥7.0 were selected for RNA sequencing.

7.4. Library preparation and sequencing

A double-stranded cDNA library was prepared starting with 1 µg of total RNA input according to the TrueSeq RNA v2 sample preparation kit protocol (Illumina, San Diego, CA). Briefly, mRNA was selected using poly-T oligo-attached magnetic beads and then fragmented. First and second cDNA strands were synthesized and end-repaired. Multiplexed adaptors were ligated after 3'-adenylation. Double-stranded cDNA templates were enriched by PCR. Libraries were validated using a DNA high-sensitivity assay on the Agilent 2100 Bioanalyzer (Agilent Technologies, Palo Alto, CA) and quantified by a Kapa Library quantification kit (Kapa Biosystems, Woburn, MA).

Libraries were clustered using the Illumina cBot (Illumina, San Diego, CA) and then paired-end sequenced (2 x 101 bp) on an Illumina HiSeq 2000. Base calling and quality filtering were performed using the CASAVA v1.8.2 (Illumina) pipeline. Sequences were aligned and mapped to the UCSC hg19 build of the *Homo sapiens* genome (from Illumina igenomes) using tophat v2.0.1[54]. Gene-counts for 23,239 unique, well-curated genes were obtained using HTSeq framework v0.5.3P3. (http://www-huber.embl.de/users/anders/HTSeq/doc/history.html).

7.5. Normalization and quality control for RNA-seq data

Gene counts were normalized, and dispersion values were estimated using the R package, DESeq v1.10.1[55]. The design matrix (row – samples; column – experimental variables) used in DESeq, along with gene-expression matrix (row – genes; column – gene counts in each sample), included the group variable (therapy-naïve, HIV–, IHG), CMV serostatus, and the personal identification number, all as factors, and other variables.

Genes with a gene count of 0 across all samples were removed; the remaining 0s were changed to 1s and these genes were used in the gene-expression matrix in DESeq. The size factors were estimated using the gene-expression matrix taking library sizes into account; these were used to normalize the gene counts. Genes with expression levels <25% of total expression from all samples were removed, leaving a total of 15,610 genes evaluated for differential expression. Of note, the filtered genes are expressed at low-levels across all samples and would not be differentially expressed (FDR<0.05) in comparisons. The dispersion factors were estimated using the options: method=blind and sharingMode=fit-only, as there were too many variables (due to personal identification numbers) to use the default. Cross-sectional differences between the groups were assessed.

Since data for functional markers (indicators, Supplementary Information Section 6.1) were available only in 56 HIV-infected patients on virally suppressive ART (Supplementary Information Sections 6.1 and 7.2) a filtered gene expression dataset was created and assessed. Of note, there were 15,403 genes in the filtered dataset (using above-mentioned filter criteria). This dataset was used to perform correlation analysis with functional markers (T-cell responsiveness, T-cell dysfunction, and plasma IL-6). Briefly, Pearson's correlation coefficient (r) and *P* value were derived for the correlation of $\log_2$ transformed gene expression with $\log_2$ transformed functional (immunologic) markers. The *P* values were adjusted for multiple comparisons using the Benjamini-Hochberg method [56]. The correlation data and the correlation cutoffs that meet FDR <0.05 are shown in **Supplementary Data 10**.

Section 8. Transcriptomic signature scores

We evaluated three SAS, seven MAS and the IMM-AGE transcriptomic signature scores.

8.1. Methods for IMM-AGE transcriptomic signature score

A list of 57 genes (tabulated below) reported by Alpert *et al.*[57] as immune-aging transcriptomic signature (IMM-AGE) was used to derive this signature. The genes significantly and consistently correlated with both age and cell-based IMM-AGE score that predicted all-cause mortality in the Framingham Heart Study offspring cohort[57]. Note: the directionality of association of IMM-AGE (transcriptomic-based) with mortality reported by us in **Fig. 2d** (higher IMM-AGE score associated with lower mortality) is opposite to the association of IMM-AGE (cell-based) with mortality reported by Alpert *et al.*[57], as all the 57 genes used in IMM-AGE (transcriptomic-based; reported by us in this study) are inversely correlated with IMM-AGE (cell-based) score they derived. The IMM-AGE transcriptomic signature score was examined in different datasets to assess its association with survival. To generate the z-score, the $\log_2$ normalized expression of each gene is z-transformed (mean centered then divided by standard deviation) across all samples and then averaged.

ABLIM1<br>*AFF3*	*CCR7*<br>*CD200*	*E2F5*<br>*EPHX2*	*IL6ST*<br>*IMPDH2*	*PLAG1*<br>*PTPRK*	*TCF7*<br>*TCL1A*

BACH2<br>*BCL11A*<br>*BIRC3*<br>*BLNK*<br>*BTLA*<br>*C11ORF31*<br>*C6ORF48*<br>*CCR6*	*CD22*<br>*CD27*<br>*CD28*<br>*CDCA7L*<br>*CHMP7*<br>*CR2*<br>*CRTC3*<br>*DPP4*	*FAIM3*<br>*FAM102A*<br>*FAM134B*<br>*FCRL1*<br>*FCRL2*<br>*HLA-DOB*<br>*HOOK1*<br>*HVCN1*	*KIAA0748*<br>*LEF1*<br>*LRRN3*<br>*MYC*<br>*NELL2*<br>*NT5E*<br>*P2RX5*<br>*PAQR8*	*RCAN3*<br>*SCML1*<br>*SLC7A6*<br>*SNX9*<br>*STAP1*<br>*STRBP*<br>*SUSD3*<br>*TCF4*	*TCTN1*<br>*UXT*<br>*VPREB3*<br>*ZNF101*<br>*ZNF671*

8.2. Survival-associated Score 1 (SAS-1) and Mortality-associated Score 1 (MAS-1)
From our previous work on immunological resilience in COVID-19[8], three survival-associated signatures (SAS) and seven mortality-associated signatures (MAS) was derived from peripheral blood transcriptome of 48 patients of the COVID-19 cohort. Of which, the topmost hits in each category (SAS-1 and MAS-1) were used in this study. Briefly, a generalized linear model (GLM) based on the negative binomial (NB) distribution with the likelihood ratio test (LRT) was used to examine the associations with outcomes: non-hospitalized [NH], hospitalized [H], nonhospitalized survivors [NH-S], hospitalized survivors [H-S], hospitalized-nonsurvivors [H-NS], and all non-survivors [NS] at 120 days. FDR<0.05 cutoff was used to identify differentially expressed (DE) genes between the comparisons.

Genes that were DE (FDR<0.05) between H vs. NH groups (genes that associated with hospitalization status), H-NS vs. H-S (genes that associated with survival in hospitalized patients) were identified. Next, in peripheral blood transcriptomes, genes that were DE between H-S vs. NH-S, NS vs. H-S, NS vs. NH-S groups were identified and the genes that overlapped in these comparisons with a concordant direction of expression were examined. This approach allowed us to identify genes that track from less- to more-severe disease severity and *vice versa* (i.e., NH-S > H-S > NS vs. NS > H-S > NH-S, respectively). Note: NS in whole peripheral blood analysis include both NH and H patients who died. DAVID v6.8 [58,59] with default settings except for selection of biological process (BP) gene ontology (GO) terms (GO-BP terms) was used to identify GO-BP terms associated with differentially expressed genes that had a concordant direction of response at an FDR <0.05.

Based on the differentially expressed genes identified in each comparison and their direction of expression (upregulated vs. downregulated) in the study group comparisons, a filtering process was applied to reduce the number of redundant GO-BP terms: a GO-BP term with a lower significant FDR (higher *P* values) was filtered if at least 75% of the genes in them were represented in another GO-BP term with a more significant FDR (lower *P* values). The filtering resulted in 51 GO-BP terms (51 set of gene signatures) and 1 signature set of 28 genes, a top of 52 gene signatures.

After adjusting for age and sex, as well as controlling for multiple comparisons (FDR correction), 29 signatures and 16 signatures out of the 52 signatures significantly associated (FDR<0.05) with hazard of mortality in COVID-19 cohort and FHS cohort,

respectively (**Supplementary Data 9a**). Ten signatures overlapped between both cohorts and were further examined. **Supplementary Data 9b** describes the gene compositions of the 3 SAS and 7 MAS gene signatures. Of these 10 signatures, the three signatures that associated (after controlling for age/sex) with lower and the seven signatures that associated with higher mortality hazards in both cohorts were termed as, Survival-Associated Signatures (SAS) and Mortality-Associated Signatures (MAS), respectively. SASs and MASs were numbered according to their prognostic capacity for predicting survival or mortality, respectively in the FHS [lowest to highest Akaike information criteria (AIC); SAS-1 to SAS-3 and MAS-1 to MAS-7] (**Supplementary Data 9c-d**). The top associated signature in each category (SAS-1 and MAS-1) were used in this study as z-scores. SAS-1 and MAS-1 correspond to the gene signature #32 (Immune response) and #4 (defense response to Gram positive bacterium), respectively as detailed in our recent report[8].

These genes in these signatures are listed below.

SAS-1 genes (*n*=21)			
CCL4L2	*CXCR5*	*ICOS*	*TCF7*
CCR4	*ETS1*	*IL24*	*TNFRSF25*
CCR7	*GPR183*	*IL7R*	*VPREB3*
CD27	*HLA-DQA1*	*MS4A2*	
CD40LG	*HLA-DRB1*	*PTGDR2*	
CXCL8	*HLA-DRB5*	*SUSD2*	

MAS-1 genes (*n*=22)			
ADAM17	*DEFA3*	*HIST1H2BF*	*RNASE3*
ADM	*DEFA4*	*HIST1H2BG*	*TBK1*
ANG	*DEFB1*	*HIST1H2BK*	*TLR2*
C5AR1	*HAVCR2*	*HIST2H2BE*	*TNFSF8*
CAMP	*HIST1H2BC*	*HMGB2*	
CD36	*HIST1H2BD*	*MYD88*	

To generate the z-scores, the normalized expression of each gene is z-transformed (mean centered then divided by standard deviation) across all samples and then averaged. Categorical score bins (high/low) of SAS-1 and MAS-1 were determined using the calculated median score values relative to each cohort.

8.2.1. SAS-1/MAS-1 profiles

The difference or change in proportions of the SAS-1/MAS-1 profiles was derived by combining the high/low expression of SAS-1 and MAS-1 scores based on median values in the entire dataset (which is dataset specific) was evaluated. High indicates expression of the score in the sample greater than the median expression of the score in the dataset whereas low indicates expression of the score in the sample less than or equal to the median expression of the score in the dataset. The following labels were used to indicate the profiles compared in the detailed statistical methods per figure panel (**Supplementary Information Sections 11.3 and 11.4**).

SAS-1	MAS-1	labels
Low	Low	SAS-1^{low}-MAS-1^{low}
Low	High	SAS-1^{low}-MAS-1^{high}
High	Low	SAS-1^{high}-MAS-1^{low}
High	High	SAS-1^{high}-MAS-1^{high}

Based on our analysis and interpretation of the data (SAS-1/MAS-1 profiles associated with mortality after controlling for age and sex as presented in **Supplementary Data 9e**), the following order of SAS-1/MAS-1 (predictors; best to worst) applies for most of the outcomes (e.g., Mortality, change during age) in the study.

SAS-1^{high}-MAS-1^{low} > SAS-1^{high}-MAS-1^{high} > SAS-1^{low}-MAS-1^{low} > SAS-1^{low}-MAS-1^{high}

How to interpret the plots: The barplots depciting the proportions of SAS-1/MAS-1 profiles should be interpreted as relative levels within one group compared to the other groups in the same panel as the median calculated data is dataset specific. Comparisons of absolute levels of the proportions of the SAS-1/MAS-1 profiles between panels or datasets should be avoided unless for meta-analysis panels. For data presented in meta-analysis panels, careful selection criteria as described in Supplementary Information Section 9.4.1 was followed so relative levels could be compared across multiple datasets presented in the same meta-analysis panel.

8.3. Publicly available expression datasets

The summary of datasets studied are presented in **Supplementary Data 13a**.

8.3.1. SLE cohort (GSE49454; **Fig. 3g, 6d, 7e, 7i; Supplementary Fig. 13c**). *Cohort/sample description*. The LUPUCE study[60] recruited patients with SLE fulfilling the 1997 ACR criteria between 2009 and 2011. Blood was taken at baseline and at each visit and CD4+ and CD8+ T-cell counts were determined for samples included in the analysis. Gene expression profiling of healthy controls (*n*=20) and SLE patients stratified according to their immune health grade (IHG). SLE patients were stratified according to first available laboratory (CD4+ count and CD4:CD8 ratio) measurements: IHG-I (*n*=12), IHG-II (*n*=23), IHG-III (*n*=4), and IHG-IV (*n*=14) (median age [IQR] years = 39 [30-51]; females: 85%)[60].

8.3.2 HIV− Offspring cohort of the Framingham Heart Study (**Fig. 7b**, **8a-b**; **Supplementary Fig. 11-13**)
The NHLBI Framingham Heart Study (FHS) was started to identify contributions to cardiovascular disease (CVD) by studying a large group of otherwise healthy individuals longitudinally. Participants from Framingham, MA were recruited in 1948 as the first generation and had biennial physical examinations and lifestyle interviews. The second-generation cohort (Offspring) was enrolled in 1971 and comprised of the first-generation participants' adult children with their spouses. The SABRe CVD Initiative performed multi-

omic analyses of FHS participants to generate biomarker data to advance personalized medicine.

In the present study, the gene expression profiling of whole blood collected at the FHS Offspring study Exam 8 conducted by the SABRe CVD Initiative in their Project 3 was analyzed. Microarray expression profiles of 2,306 participants of the FHS Offspring study were paired with clinical exam measurements, laboratory diagnostics, lifestyle evaluations, and mortality outcomes to evaluate the contributions of IR erosion to all-cause mortality. Follow-up time onwards from Exam 8 was calculated for survival analyses (median: 8.163 years, IQR: 7.483 – 8.841, min: 0.178, max: 9.815). Data was accessed through dbGaP study accessions phs000007.v30.p11 and phs000363.v17.p11.

Framingham Heart Study dbGaP Acknowledgement Statement: The Framingham Heart Study is conducted and supported by the National Heart, Lung, and Blood Institute (NHLBI) in collaboration with Boston University (Contract No. N01-HC-25195 and HHSN268201500001I). This manuscript was not prepared in collaboration with investigators of the Framingham Heart Study and does not necessarily reflect the opinions or views of the Framingham Heart Study, Boston University, or NHLBI. Additional funding for SABRe was provided by Division of Intramural Research, NHLBI, and Center for Population Studies, NHLBI.

8.3.3 San Antonio Family Heart Study (**Fig. 7c; Supplementary Fig. 11-12**)
Transcription profiling of human lymphocytes from 1240 Mexican Americans (E-TABM-305)[61]. *Cohort/sample description.* To date, most human studies have compared gene expression between age classes of participants (e.g., young vs. old), but such categorical comparisons do not reveal the potential trajectories of changes that may occur during aging. Moreover, while many genes are expected to show changes in expression with age, a substantial proportion of the individual variation in aging may result from genotype × age interaction (G × AI) effects on a smaller number of genes. We sought to understand the expression levels across age of gene signatures that associate with survival/mortality and IHG status in this cohort.

Recruitment of the Mexican American families[62] in the San Antonio Family Heart Study (SAFHS) began in 1991 with ascertainment on family size rather than any disease state, although the cohort reflects the elevated risk of this ethnic stratum for Type 2 diabetes (15.3% at recruitment) and other cardiovascular risk factors[63]. Participants have been recalled up to three times to provide a wealth of genetic and phenotypic data. The 1240 SAFHS participants with gene expression data represent 46 extended families ranging in size from 3 to 87 phenotyped relatives.

8.3.4. Meta-analysis for comparison of SAS-1/MAS-1 signatures in controls and patients with mild dementia (**Fig. 7d; Supplementary Fig. 12b**).
For these analyses shown in **Fig. 7d** (main text), gene expression datasets from whole blood (GSE140829, and GSE140830) analyzed on the same expression platform

(HumanHT-12 v4 Expression BeadChip (nuID) also known as GPL15988)[64] was evaluated.

8.3.4.1. GSE140829. (**Fig. 7d; Supplementary Fig. 12b**) *Cohort/sample description.* Whole blood samples from controls (*n*=249; median age [IQR] years = 73 [69 -78]; females: 56%) and dementia patients (including Alzheimer's disease and mild cognitive impairment; *n*=338; median age [IQR] years = 72 [68-79]; females: 51%)[64].

8.3.4.2. GSE140830. (**Fig. 7d; Supplementary Fig. 12b**) *Cohort/sample description.* Whole blood samples from controls (*n*=281; median age [IQR] years = 72 [68-77]; females: 56%) and dementia patients (multiple disorders within the frontotemporal dementia; *n*=261; median age [IQR] years = 66 [60-72]; females: 51%)[64]. The multiple disorders within the frontotemporal dementia included behavioral variant frontotemporal dementia; semantic variant primary progressive aphasia; non-fluent variant primary progressive aphasia; progressive supranuclear palsy; corticobasal syndrome.

8.3.5. GSE16363 (**Fig. 7i**). *Cohort/sample description.* Lymph node samples from 5 uninfected donors (*n*=10 samples), and HIV+ patients classified into 3 groups. They were 9 asymptomatic HIV+ patients (*n*=18 samples), 9 acute HIV+ patients (*n*=16 samples), and 4 AIDS patients (*n*=8 samples) (median age [IQR] years = 39 [32-45]; females: 15%). Note: Two samples from each donor and patient were available in GEO and all samples were used in the analysis [65,66].

8.3.6. Meta-analysis for comparison of SAS-1/MAS-1 signatures in community acquired pneumonia (CAP) and fecal peritonitis (FP) cohort from UK (**Fig. 8d; Supplementary Fig. 11c**)

For these analyses shown in **Fig. 8d**, expression datasets of Leukocytes (E-MTAB-4421, E-MATB-4451, E-MATB-5273 and E-MATB-5274) analyzed on the same expression platform (A-MEXP-2210 – Illumina HumanHT-12_V4_0_R1_15002873_B also known as GPL10558) [67,68] was evaluated.

8.3.6.1. E-MTAB-4421. (**Fig. 8d; Supplementary Fig. 13f**) *Cohort/sample description.* Leukocyte samples from patient with severe sepsis due to community acquired pneumonia admitted to the intensive care unit with either sepsis response signature 1 (SRS1, G1; *n*=108) or sepsis response signature 2 (SRS2, G2; *n*=157) (median age [IQR] years = 64 [52-75]; females: 46%) [67]. G1 (SRS1) is associated with higher mortality than G2 (SRS2); the SRS1 and SRS2 were defined using baseline samples at admission into the intensive care unit. This dataset is part of their discovery cohort.

8.3.6.2. E-MTAB-4451. (**Fig. 8d; Supplementary Fig. 13f**) Cohort/sample description. Leukocyte samples from patient with severe sepsis due to community acquired pneumonia admitted to the intensive care unit with either sepsis response signature 1, SRS1 (G1; *n*=37) or sepsis response signature 2, SRS2 (G2; *n*=69) (median age [IQR] years = 73.5 [63.5-80.0]; females: 26%) [67]. G1 (SRS1) is associated with higher mortality

than G2 (SRS2); the SRS1 and SRS1 was defined using baseline samples at admission into the intensive care unit. This dataset is part of their validation cohort.

8.3.6.3. E-MTAB-5273. (**Fig. 8d; Supplementary Fig. 13f**) *Cohort/sample description.* Leukocyte samples from healthy controls and patients with community acquired pneumonia (CAP) who had the transcriptional sepsis response signature 1 (SRS1, CAP:G1), or CAP patients with the transcriptional SRS2 signature (CAP:G2) or patients with fecal peritonitis (FP) with SRS1 (FP:G1), or FP-patients with SRS2 (FP:G2) (*n*=147, median age [IQR] years = 66 [52.5-76.0]; females: 50%) [68]. G1 (SRS1) is associated with higher mortality than G2 (SRS2); the SRS1 and SRS1 was defined using baseline samples at admission into the intensive care unit. This dataset is part of their discovery cohort. The first available samples (147 of 231) from 147 patients ordered by patient ID were used for plots and statistics.

8.3.6.4. E-MTAB-5274. (**Fig. 8d; Supplementary Fig. 13f**) *Cohort/sample description.* Leukocyte samples from patients with community acquired pneumonia (CAP) who had the transcriptional sepsis response signature 1 (SRS1, CAP:G1), or CAP patients with the transcriptional SRS2 signature (CAP:G2) or patients with fecal peritonitis (FP) with SRS1 (FP:G1), or FP-patients with SRS2 (FP:G2) (*n*=147, median age [IQR] years = 71 [62.2-77.0]; females: 36%) [68]. G1 (SRS1) is associated with higher mortality than G2 (SRS2); the SRS1 and SRS1 was defined using baseline samples at admission into the intensive care unit. This dataset is part of their validation cohort.

8.3.7. E-MTAB-1548. (**Fig. 8d**) *Cohort/sample description.* Whole blood samples from patients with sepsis (*n*=43), post-surgical patients with septic shock who are non-survivors (septic shock_NS; *n*=17), post-surgical patients with septic shock who are survivors (septic shock_S; *n*=22), patients with systemic inflammatory response syndrome (SIRS; *n*=58) and healthy controls (normal; *n*=15). Median age [IQR] years of patients (excluding the healthy controls) in the study = 72 [62-79]; females among patients (excluding the healthy controls): 28% [69,70]. The samples were collected as part of the EXPRESS study (Gene Expression in Sepsis), which was an observational prospective study aimed at evaluating gene expression profiles in patients with sepsis. During the observation period, 104 patients undergoing surgery were recruited. Seventy-four of these patients presented with sepsis, 30 patients showed Systemic Inflammatory Response Syndrome (SIRS) with no sepsis (control group). Fifteen healthy volunteers of similar ages to the patients were recruited from the hospital staff for gene expression data normalization.

8.3.8. Natural influenza season and other acute respiratory viral infections (GSE68310 [71]; **Fig. 8e**). *Cohort/sample description*. To understand the molecular basis and network orchestration of host responses, Zhai et al. [71] prospectively enrolled 1,610 healthy adults in the fall of 2009 and 2010, followed the subjects with influenza-like illness (*n*=133) for 3 weeks, and examined changes in their peripheral blood gene expression. About 133 participants completed all study visits and yielded technically adequate peripheral blood microarray gene expression data. Seventy-three (55%) had an influenza virus infection, 64 influenza A and 9 influenza B. The remaining subjects had a rhinovirus infection

(*n*=32), other viral infections (*n*=4), or no viral agent identified (*n*=24). They analyzed the global gene expression profiles of peripheral whole blood in the 133 adults with an acute respiratory infection at up to seven time points before, during, and after the occurrence of illness (**adjacent figure**). They discovered distinct phases of the host response spanning 6 days after infection and identified genes that differentiate influenza from non-influenza virus infection. Samples from consecutive influenza seasons (2009-2010 and 2010-2011) were pooled for this analysis.

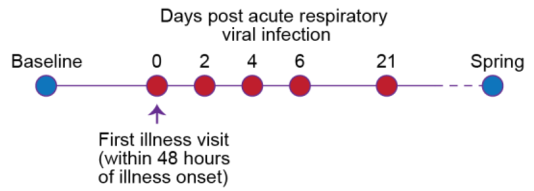

8.3.9. Symptomatic respiratory viral infections in adults (GSE17156[72]; **Fig. 8g**) *Cohort/sample description*. Gene expression profiling of peripheral blood samples from healthy volunteers inoculated experimentally with (i) H3N2 and were asymptomatic (Influenza:Asy; *n*=9) or became symptomatic (Influenza:Sym; *n*=8); (ii) RSV and were either asymptomatic (RSV:Asy; *n*=11) or became symptomatic (RSV:Sym; *n*=9), and (iii) rhinovirus and were asymptomatic (Rhinovirus:Asy; *n*=10) or became symptomatic (Rhinovirus:Sym; *n*=9); samples were obtained at baseline (B) and when peak (P) symptoms developed after inoculation in persons who became symptomatic. Infections were pooled for the analysis presented.

8.3.10. Influenza A H1N1 and H3N2 virus infection in adults (GSE52428 [73]; **Fig. 8h**) *Cohort/sample description*. Woods et al. [73] used microarrays to assay peripheral blood gene expression at baseline and every 8 hours for 7 days following intranasal influenza A H1N1 or H3N2 inoculation in healthy volunteers (**adjacent Figure**).

Associations between total symptom score after inoculation and SAS-1/MAS-1 profiles are shown in **Fig. 8h**.

Timepoints for Baseline (-24hr and 0hr) were pooled and timepoints for Peak infection (60hr and 69.5hr) were pooled as categorical time variables.

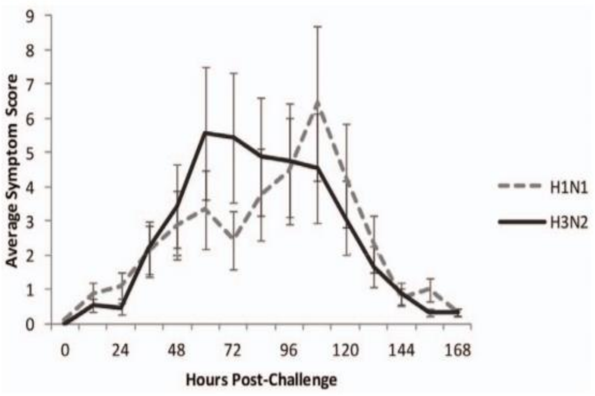

Figure adopted from Woods et al. *PLoS One* 8, e52198 (2013)

8.3.11. Influenza cohort (GSE111368[74]; **Fig. 8i**) *Cohort/sample description*. Gene expression profiling of whole blood samples from patients with severe influenza infection requiring hospitalization collected at three timepoints: T1 (recruitment); T2 (approximately 48h after T1); T3 (at least 4 weeks after T1) studied by age groups (18-39 years (*n*=46) and 31-71 years (*n*=63)). Severity was defined at recruitment using a three-point scale: severity 1, no supplemental oxygen required; severity 2, oxygen by mask; severity 3,

mechanical ventilation. (Source: GSE111368, median age [IQR] years = 72 [67-76]; females: 54%).

8.3.12. Meta-analysis of ageing, HIV, and TB. For these analyses, we evaluated expression datases of whole blood samples (E-TABM-1036, GSE29429, GSE19439, GSE19442, and GSE19444) analyzed on the same expression platform (GPL6947)[75-77] [**Supplementary Fig. 12c, 13b**].

8.3.12.1. E-TABM-1036. (**Supplementary Fig. 12c, 13b**) *Cohort/sample description.* Finnish DILGOM cohort[78]. Transcription profiling by gene expression array of a human population-based collection of participants from the capital region of Finland[77]. Samples were collected as part of the Dietary, Lifestyle, and Genetic determinants of Obesity and Metabolic syndrome (DILGOM) survey (2007). The DILGOM survey was originally performed as an extension of the FINRISK 2007 study[78] (*n*=7993; age 25–74 years; from five geographical areas in Finland). The national, cross-sectional FINRISK surveys have been carried out every 5 years since 1972 to assess the risk factors of chronic diseases (e.g., CVD, diabetes, obesity, cancer) and health behaviour in the working age population in Finland. The DILGOM study (*n*=5024) aimed to observe and characterize the risk factors for metabolic and cardiovascular diseases in the Finnish population both at the epidemiological (diet, psychosocial factors, lifestyle, environment etc.) and at the genetic level[77]. In addition to questionnaire data on health and lifestyle, a blood sample was drawn in the morning after at least 10h of overnight fasting for genetic and biomedical analyses. Genome-wide gene expression were measured for a subsample from Helsinki/Vantaa metropolitan area (*n*=518, age 25–74 years, 54% females)[79,80].

8.3.12.2. GSE29429. (**Supplementary Fig. 12c**) *Cohort/sample description.* 58 acute HIV patients were recruited from locations in Africa (*n*=43) and the United States (*n*=15). Patient samples were collected at study enrollment (confirmed acute) for all patients and at weeks 1, 2, 4, 12, and 24 for training and test set constituents. 13 of 15 US patients initiated ART after enollment and before the week 1 visit. The remining 2 US patients and 43 African patients were not placed on ART through the 24 week time course. Matched uninfected controls patients were also recuited from each site (*n*=55). In total 232 samples were collected[75].

8.3.12.3. GSE19439. (**Supplementary Fig. 12c**) *Cohort/sample description*. Whole blood collected in tempus tubes from patients with different spectra of TB disease and healthy controls from UK. All patients were sampled prior to the initiation of any antimycobacterial therapy. Active Pulmonary TB: PTB – All patients confirmed by isolation of *Mycobacterium tuberculosis* on culture of sputum or bronchoalvelolar lavage fluid. Latent TB: LTB – All patients were screened at a tuberculosis clinic, being either new entrants to the UK from endemic countries or being household contacts of infectious cases[76].

8.3.12.4. GSE19442. (**Supplementary Fig. 12c**) *Cohort/sample description*. Whole blood collected in tempus tubes from patients from South Africa with different spectra of TB disease. All patients were sampled prior to the initiation of any antimycobacterial therapy. Active Pulmonary TB: PTB – All patients confirmed by isolation of *Mycobacterium*

tuberculosis on culture of sputum or bronchoalvelolar lavage fluid. Latent TB infection: LTBI – All patients were screened at a tuberculosis clinic, being either new entrants to the UK from endemic countries or being household contacts of infectious cases[76].

8.3.12.5. GSE19444. (**Supplementary Fig. 12c**) *Cohort/sample description*. Whole blood collected in tempus tubes from patients with different spectra of TB disease and healthy controls from UK. All patients were sampled prior to the initiation of any antimycobacterial therapy. Active Pulmonary TB: PTB – All patients confirmed by isolation of *Mycobacterium tuberculosis* on culture of sputum or bronchoalvelolar lavage fluid. Latent TB: LTB – All patients were screened at a tuberculosis clinic, being either new entrants to the UK from endemic countries or being household contacts of infectious cases[76].

8.3.13. Datasets from Vitality 90+ cohort (**Supplementary Fig. 13e**):

8.3.13.1 GSE65219. (**Supplementary Fig. 13e**) *Cohort/sample description.* PBMC samples from nonagenarian (*n*=146, ages: ≥90 years; females: 71%) and young (*n*=30, median age [IQR] years = 22.5 [20.2-24.0]; females: 70%) participants[81]. All of the study subjects were of Western European descent. The nonagenarians participants were from the Vitality 90+ Study[82-84], which is an ongoing prospective population-based study involving individuals that includes both home-dwelling and institutionalized individuals aged 90 years and older, living in the city of Tampere, Finland. The recruitment and characterization of the participants were performed as previously reported for earlier Vitality 90+ study cohorts (Goebeler et al.[84]). The individuals in the current study were born in 1920, and the samples used in this study were collected in the year 2010. The nonagenarians included in the study had not had any infections or received any vaccinations in the 30 days prior to the blood sample collection. The young control subjects consisted of healthy, non-smoking laboratory personnel without medically diagnosed chronic illnesses or infectious conditions or received any vaccinations within the two weeks prior to the blood sample collection.

8.3.13.2. GSE65218. (**Supplementary Fig. 13e**) *Cohort/sample description.* PBMC samples from nonagenarian participants (*n*=151; ages: ≥90 years; females: 70%) in the Vitality 90+ Study[67,70]. The details of the cohort and participants are described above and same as for GSE65219. The all-cause mortality data (the median follow-up time was 2.55 years)[67], including the dates of death, were collected from the Population Register Center. The mortality rate during the follow-up was 32.5%; of the 151 individuals, 49 died and 102 survived the follow-up period. There were no losses to follow-up. The frailty data reported for these participants were used to assess the IC-IF states.

8.3.14. Burn injury patients (GSE182616[85]; **Supplementary Fig. 13g**)
Cohort/sample description. Gene expression profiling of blood samples from patients with severe burn injury presenting to a regional center within 5 hours of thermal injury due to flash, flame, or contact with anticipated need for hospital admission. Data grouped by total body surface area (TBSA) ≤ or > 20%. Sixty patients were analyzed, most patients were male (80%) with a median [IQR] age of 39 [30.5-55.0] years, and median [IQR]

TBSA of 19.2% [11.7-33.6%]. Thirty-five patients had %TBSA injury >20%, and this group experienced greater mortality.

8.3.15. Sepsis (GSE185263[86]; **Supplementary Fig. 13h**)
Cohort/sample description. Gene expression profiling of blood samples from patients with early/pre-sepsis (*n*=348, median [IQR] age = 61 [44-72] years; females = 42%) admitted to ER/ICU. The patients categorized by survival status and by Sequential Organ Failure Assessment (SOFA) score in patients. Increasing SOFA score associates with increased disease severity, organ dysfunction, and mortality. The dataset also included data from healthy controls (*n*=44, median [IQR] age = 51 [29-59] years; females = 59%).

8.3.16. pre-CC mice with influenza infection (GSE30506; **Supplementary Fig. 13i**).
Cohort/sample description. Gene expression profiling on lung samples from different pre-Collaborative Cross (CC) mice lines (*n*=44) collected at day 4 post infection with the mouse adapted influenza A strain A/PR/8/34 (H1N1)[87]. Based on the distribution of Influenza-infected phenotypes (immunohistochemical (IHC) staining for viral protein and weight loss at 4 days post infection) the mice were categorized into the extreme phenotypes with regard to host response: severe (high) response to infection (*n*=26, IHC score: 4 or 5, % Weight loss >15%) and mild (low) response to infection (*n*=18, IHC score: 0 or 1, % Weight loss <5%).

Section 9. Analysis of datasets of GEO and ArrayExpress

9.1. Overall

After we derived the transcriptomic signature scores, we evaluated them in publicly available databases and selected datasets available in NCBI (GEO) and EMBL (ArrayExpress) which are detailed in Supplementary Information Section 8.3.

9.2. Data download and normalization

All download, normalization and score analysis was performed in R statistical environment. Following packages were used: GEOquery, ArrayExpress, Biobase, limma, preprocessCore, stringr, geepack, corrplot, DESeq, reshape, downloader etc. Modified version of GEO2R script sourced from NCBI GEO was used for GEO datasets and custom script based on ArrayExpress R package was used for ArrayExpress datasets. These scripts were used to download and normalize the data. Details on the value type provided and normalization performed are shown in **Supplementary Data 13a** and methods are summarized below.

9.2.1. Microarray datasets

For microarray dataset, whenever possible, $\log_2$ and quantile normalization was performed before computing transcriptomic signature scores. If both normalizations were not feasible, either $\log_2$ (if the data was already quantile normalized) or quantile (if the

data was already log_2 normalized or >5% of the data had negative values) normalization was performed. For datasets having values provided as log_2 ratios of samples, scores for signatures were computed after quantile normalization.

The normalization performed on the GEO and ArrayExpress datasets reported in the main text and supplementary information are listed in the **Supplementary Data 13a**. The details on platforms of the datasets we analyzed are listed in the **Supplementary Data 13a-b**.

9.3. Probe-Gene information

For microarray datasets, the gene symbol information for probes were determined from the feature data associated with platforms on which the gene expression data was generated. In cases where gene symbol information was not available as part of feature data, they were manually annotated using standard resources: HUGO Gene Nomenclature Committee (HGNC) at the European Bioinformatics Institute (https://www.genenames.org) and ID Converter System (ICS) at http://biodb.jp[88].

In instances where there were multiple probes or transcripts representing a single gene, mean values were determined and used for computing the scores for signatures.

9.4. Data analysis

9.4.1 Meta-analysis

For meta-analyses (e.g., data presented in **Fig. 7d**), the samples from two or more datasets combined for analyses were from the same source (tissue or cell type) and assayed on the same platform. All datasets were filtered for common probes. Then an expression matrix of the probes and samples was created and concurrently normalized as stated in Supplementary Information Section 9.2 before scores for signatures were computed. Example: if dataset #1 provided log_2 values and dataset #2 was quantile normalized, dataset #1 would be un-log transformed by exponentiation with the base 2 before combining with dataset #2 for concurrent normalization and computation of scores.

9.4.2 Handling of phenotype data

The phenotype groups for plots were determined from the phenotype data deposited in the GEO or ArrayExpress along with the dataset. The phenotype groups were classified based on the hypothesis evaluated.

9.4.3 Statistics and plots

All analysis was conducted using R. Reported *P* values are 2-sided and set at the 0.05 significance level. The models and *P* values were not adjusted for multiple comparisons in the pre-specified subgroup analyses, unless otherwise noted.

9.4.3.1 Statistics for transcriptomic signature scores

For cross-sectional comparisons, Wilcoxon rank-sum test (unpaired, Mann-Whitney U-test) was used to evaluate the difference in scores between two groups. Kruskal-Wallis test was used to evaluate the overall differences between three or more groups. For comparison of groups with multiple samples from same individuals, we used a linear generalized estimating equation (GEE) model based on the normal distribution with an exchangeable correlation structure unless otherwise stated. Pearson's correlation was used to evaluate the correlation between transcriptomic signature scores.

9.4.3.2 Plots

Boxplots were used to represents the median [IQR] of the indicated variables. Line plots with error bars were used represent the mean ± standard error of the mean [SEM] of the indicated variable. Line plots with bands were used to represent either the odds with 95% confidence bands or mean ± standard error of the mean [SEM] of the indicated variable. Line plots were used to represent proportions of indicated variables. Kaplan-Meier plots were used to represent proportion survived over time since score calculation (baseline) by indicated groups. Heatmaps were used to represent correlations of gene signature scores and continuous age. Stacked barplots or barplots were used to represented proportions or correlation coefficient of indicated variables. Forest plots were used to plot OR or HR [either unadjusted or adjusted]. Pie charts were used to represent proportions of indicated variables.

9.5. Quality control of the dataset and interpretation

We stress that the transcriptomic signature score is a relative term within a dataset, and it is challenging to compare the score across different datasets. For the meta-analyses, we used a series of criteria as described in Supplementary Information Section 9.4.1 to make comparisons more equitable between the datasets.

We are mindful that different RNA microarray or RNA-seq platforms have differences in the availability of gene probes corresponding to the genes in a given transcriptomic signature score. This could affect the interpretation of the findings. Thus, we indicated the gene count range in each dataset (**Supplementary Data 13b**). As the overall median (IQR) percentage of available genes are high, 90.9% (85.7%-100%), the chance of those unavailable genes impacting on our interpretation are low. In addition, we stress that transcriptomic signature scores were defined in relative terms and caution is needed for cross-dataset comparisons.

9.6. Survival analysis

In the COVID-19 cohort, a Cox proportional hazards model, adjusted for sex and age as a continuous variable, was used to determine if the gene scores associated with 90-day survival. In the Framingham Heart Study offspring cohort, a Cox proportional hazards model, adjusted by sex and age as a continuous variable, was used to determine if the

gene scores associated with survival. An FDR was used to correct the *P* values from the Cox proportional hazards models for multiple comparisons and FDR<0.05 used to determine whether gene score significantly associated with hazard of mortality. Kaplan-Meier survival plots of in the Framingham Heart Study offspring cohort are accompanied by *P* values determined by log-rank test.

Section 10. Predictors and outcomes

10.1. Predictors: grades of antigenic stimulation and IR metrics

For determining the association between level of antigenic stimulation and IHG status in HIV– persons, proxies were used to grade this level and quantify host antigenic burden accumulated: (1) age was considered as a proxy for repetitive, low-grade antigenic experiences accrued during natural aging; (2) a behavioral activity score (BAS) based on behavioral risk factors (condom use, number of clients, number of condoms used per client) and a total STI score based on direct [syphilis (rapid plasma reagin test) and gonorrhea] and indirect (vaginal discharge, abdominal pain, genital ulcer, dysuria, and vulvar itch) indicators of STI were used as proxies in HIV– FSWs for whom this information was available; and (3) *S. haematobium* egg count in the urine was a proxy in children with this infection. For HIV+ persons, plasma HIV VL was a proxy for level of HIV-associated antigenic stimulation.

10.2. Key predictor-outcome dyads

(1) IHGs-age (proxy for accumulated antigenic experience); (2) IHGs at first episode of CSCC in RTRs with the outcome of second episode of CSCC; (3) IHG at baseline in HIV+ persons and AIDS development; (4) IHGs at baseline in FSWs with future HIV seroconversion; (5) IHG at baseline with COVID-19 outcomes; (6) gene signatures and survival rates in either persons with or without acute COVID-19; (7) gene signatures and sepsis survival; and (8) gene signatures and influenza outcomes.

Section 11. Statistical analysis and analytical approach

11.1. Overview

Logistic regression analyses were used to evaluate entry/baseline IR status and future HIV seroconversions; results reported in odds ratios (ORs) with 95% confidence intervals (Cis). Kaplan-Meier plots were generated to depict rates of development of CSCC in HIV− RTRs and the rate of disease progression to AIDS (1993 CDC criteria) in HIV+ participants. The log-rank test was used to evaluate for overall significance.

When comparing categorical data, the χ^2 test was used when sample sizes were large (defined as when 80% or more of the values in the contingency table were ≥5 or none of the values in the contingency table was 0 or n's in the contingency table make Fisher's exact test computationally infeasible). In cases were the χ^2 was not applicable the Fisher's exact test was used. For continuous variables, ANOVA, linear

regression, Wilcoxon rank-sum test, Kruskal-Wallis test, and Pearson or Spearman correlation coefficient analyses were used where appropriate. Follow-up times and analyses were prespecified.

Pearson vs. Spearman Correlation coefficient:

1. Pearson's and Spearman's correlation coefficient were used appropriately depending on the assumption of normality in the data evaluated.
2. For correlations of BAS vs. STI scores, Spearman's correlation coefficient was used.
3. For correlation of genes with immunologic correlates, since log_2 transformed gene expression and log_2 transformed functional (immunologic) markers were used and as they all fit normal distribution, Pearson's method was used to estimate the correlation coefficients.
4. The gene scores that are linear combination of Z-transformed log_2 normalized gene expression are well approximated by normal distribution and therefore Pearson's correlation coefficient was used.

Of note, for the association of gene scores with outcomes, linear regression (linear model) was used to test them, instead of non-parametric tests as highlighted below in the panel-by-panel detailed statistical methods for each of the figures. For median-based SAS-1/MAS-1 profile distribution analysis, non-parametric tests were used as appropriate.

Reported *P* values are 2-sided and set at the 0.05 significance level. The models were not adjusted for multiple comparisons in the pre-specified subgroup analyses, unless otherwise noted. All analysis was conducted using R (https://cran.r-project.org/). All cutoffs and statistical tests were determined pre hoc. The statistical methods used in each figure panels are detailed in **Supplementary Information Sections 11.3 and 11.4**.

11.2. Statistical analysis for levels of immune traits analyzed in the SardiNIA cohort

Overview

We evaluated 75 distinct, non-overlapping, informative, immune traits from SardiNIA cohort (**Supplementary Data 12**) for their association with **nonoptimal IR linked to CD8-CD4 disequilibrium grades IHG-III or IHG-IV** vs. **age** as follows. The trait levels were normalized using inverse normal transformations, and the covariate-adjusted residuals were used and are depicted in **Fig. 10a-c**. Data are presented as inverse-normalized residuals after adjusting for sex and/or age, as necessary. **Supplementary Data 12** provides additional details.

Comparisons by IHG status

Comparisons of the marker levels were made between IHG-I vs. IHG-III and IHG-II vs. IHG-IV. This approach mitigated confounding that might be attributable to differences in CD4+ T-cell counts since both IHG-I and IHG-III are defined by higher CD4+ T-cell counts (≥800 cells/mm^3), whereas IHG-II and IHG-IV are both defined by lower CD4+ T-cell counts. For assignment of a trait as being associated with CD8-CD4 disequilibrium (IHG-III or IHG-IV), differences in the levels of the trait in both comparisons (IHG-I vs. IHG-III and IHG-II vs. IHG-IV) had to be significant (by Wilcoxon rank-sum test), after adjusting

for covariates (age and sex) and correcting for multiple comparisons. For purposes of description, when the trait levels differed in both comparisons, those variations were attributed to CD8-CD4 disequilibrium (IHG-III or IHG-IV) in **Fig. 10a-b**.

Comparisons by age within participants preserving IHG-I and IHG-II grades
IHG-I and IHG-II are the most prevalent grades across lifespan. Differences in the levels of the immune traits by age were analyzed within participants having IHG-I and IHG-II and were adjusted for sex (**Fig. 10a-b**). The age stratum for younger vs. older participants was 18-39 vs. ≥70 years. We chose a cutoff of ≥70 years for older age, as we observed shifts in the prevalence of the IHGs within this age range. In order to assign a trait as being associated with age, differences in the levels of the trait in both comparisons (younger vs. older within IHG-I and younger vs. older within IHG-II) had to be significant (by Wilcoxon rank-sum test), after adjusting for covariates (sex) and correcting for multiple comparisons. For purposes of description, when the marker levels differed in both comparisons, those variations were attributed to age in **Fig. 10a-b**.

Association of traits into 4 groups
A significance value of $P<1.67 \times 10^{-4}$ (75 traits compared x 4 comparisons: IHG-I vs. IHG-III; IHG-II vs. IHG-IV; younger vs. older within IHG-I and younger vs. older within IHG-II) was ascribed to the comparisons mentioned above to determine the immune traits' associations with CD8-CD4 disequilibrium (IHG-III or IHG-IV) and/or age or Neutral as per the criteria noted in the following table.

Table: Criteria used for ascribing an immune trait to be associated with HG-III or IHG-IV (CD8-CD4 disequilibrium) and/or age or neither (neutral)

Grp #	Group	CD8-CD4 disequilibrium comparisons		Age comparisons	
		IHG-I vs. IHG-III	IHG-II vs. IHG-IV	IHG-I: Young vs. Old	IHG-II: Young vs. Old
1	Nonoptimal IR linked to IHG-III or IHG-IV, after controlling for age	Sig	Sig	--	--
		Sig	Sig	Sig	--
		Sig	Sig	--	Sig
2	Age in persons with IHG-I or IHG-II	--	--	Sig	Sig
		Sig	--	Sig	Sig
		--	Sig	Sig	Sig
3	IHG-III or IHG-IV and Age	Sig	Sig	Sig	Sig
4	Neutral	--	--	--	--
		Sig	--	--	--
		--	Sig	--	--
		--	--	Sig	--
		--	--	--	Sig
		Sig	--	Sig	--
		Sig	--	--	Sig
		--	Sig	Sig	--
		--	Sig	--	Sig

Sig - $P<1.67 \times 10^{-4}$; -- no difference

Signature groups: Based on the direction of association of the traits with nonoptimal IR linked to CD8-CD4 disequilibrium grades IHG-III or IHG-IV and age, the markers were grouped into signatures # 1-19. The direction was assigned based on whether the levels of traits were higher or lower in IHG III in the comparison for IHG III vs. IHG I or in IHG IV in the comparison for IHG II vs. IHG IV; and in older participants in the comparison for younger vs. older with IHG I and IHG II. The directionality to the trait association with

nonoptimal IR linked to CD8-CD4 disequilibrium grades IHG-III or IHG-IV or age was assigned only when at least one of two comparisons was significant at $P<1.67 \times 10^{-4}$.

	Group 1				Group 2				Group 3				Group 4						
	Nonoptimal IR linked to IHG-III or IHG-IV, after controlling for age				Age in IHG-I or IHG-II				IHG-III or IHG-IV and age				Neutral						
Total # of traits	13				22				10				30						
Signature #	1	2	3	4	5	6	7	8	9	10	11	12	13	14	15	16	17	18	19
IHG-III or IHG-IV	↑↑	↓↓	↑↑	↑↑	ND	ND	↑	↑	↑↑	↑↑	↓↓	↓↓	ND	↑	↓	↑	↓	ND	ND
Age in IHG-I or IHG-II	ND	ND	↑	↓	↑↑	↓↓	↑↑	↓↓	↑↑	↓↓	↑↑	↓↓	ND	↑	↓	ND	ND	↑	↓
# of traits	5	1	4	3	5	15	1	1	2	5	1	2	12	1	1	2	2	10	2

ND – No Difference; ↑↑ or ↓↓ - both comparisons for IHG-III/IV or age are significant; ↑ or ↓ - only one of the comparisons for IHG-III/IHG-IV or age is significant

11.3. Details of statistical methods and analytical strategies per main figure panel

11.3.1. Figure 1

- *Panels* a-b:
  - Schema. No statistical analysis was performed.

11.3.2. Figure 2

- *Panels* a-c:
  - Schema. No statistical analysis was performed.
- *Panel* d:
  - Data derived from COVID-19 cohort and FHS offspring cohort.
  - Table of results from Cox proportional hazards models evaluating the association of 90-day mortality in COVID-19 cohort (left) or 9-year all-cause mortality in FHS offspring cohort (right) (outcomes) with selected transcriptomic signatures (SAS-1, MAS-1, and IMM-AGE scores; predictors) adjusted by age and sex. FHS follow-up time median: 8.163 yrs, IQR: 7.483 – 8.841, min: 0.178, max: 9.815. Adjusted hazards ratios (aHR), 95% confidence intervals (CI), and nominal *P* values are shown for each model.
  - SAS-1 and MAS-1 were identified by examining 52 gene signature scores in the acute COVID-19 and FHS cohorts using an FDR correction in both cohorts. In this report, we focused on the 10 signatures (3 SAS and 7 MAS gene signatures) that associated with survival in both cohorts, after correcting for FDR, as reported in **Supplementary Data 9**. Based on the AIC values, SAS-1 and MAS-1 were selected as being most prognostic. The associations of the IMM-AGE score with lifespan in the FHS cohort has been reported previously and used herein as a control and hence no additional corrections for multiple comparisons were warranted.
- *Panel* e:
  - Schema. No statistical analysis was performed.

- *Panel* f:
  - Data derived from SardiNIA cohort.
  - *Left*: Distribution of IHGs by age strata. *P* value determined by χ^2 test.
  - *Right*: Distribution of IHGs by sex and age strata. *P* values determined by χ^2 test.
  - Two *P* values presented in this panel. We did not correct for multiple comparisons, as the right panel is an expansion of the left panel by sex. Additionally, since both *P* values were <0.001, a Bonferroni corrected *P* values would remain significant.
- *Panel* g:
  - Data derived from SardiNIA cohort.
  - Odds with 95% CI bands of indicated IHG comparisons by sex across age. *P* values determined by logistic regression.
  - There are four separate hypotheses (different sample groups) being tested, each with two *P* values. We did not apply a correction for multiple comparisons for the two *P* values within each hypothesis. We also did not apply a correction for multiple comparisons across the four plots, as they depict results for separate hypotheses. Moreover, since seven of the eight significant *P* values were <0.001, a Bonferroni corrected *P* value would remain significant.
- *Panel* h:
  - Schema. No statistical analysis was performed.

11.3.3. Figure 3

- *Panel* a:
  - Data derived from cohort of Kenyan HIV− children with Schistosomiasis.
  - Distribution of IHGs (outcome) by egg counts (predictor). *P* value determined by Fisher's exact test.
  - One *P* value; no correction required.
- *Panel* b:
  - Data derived from COVID-19 cohort.
  - Paired Distribution of IHGs (outcome) at baseline and during convalescence (predictors) among 220 COVID-19 patients in the overall group (left) and younger (middle; <60 years) patients by overall and CMV serostatus, and older (right; ≥ 60 years) patients by overall and CMV serostatus. *P* values determined using a logsitic GEE model with an exchangeable correlation structure for evaluating if the odds of IHG-I, II, or IV differ by time (baseline vs. convalescence).
  - We examined the changes in IHG distributions in the same patients evaluated at baseline vs. convalescence. Since the overall changes between baseline and convalescence were highly significant (*P*<0.001), we performed post-hoc analyses to determine the changes according to IHG status (IHG-I, IHG-II, and IHG-IV), reporting three *P* values in the overall group, the younger age group, and the older age group. Since the *P* values within each analysis were highly significant, a Bonferroni corrected *P* value would remain significant. We did not compare whether the changes in IHGs between younger vs. older were different. For these reasons, additional tests for multiple comparisons were not warranted.
- *Panel* c:
  - Schema. No statistical analysis was performed.
- *Panel* d:

- Data derived from COVID-19 cohort.
- Distribution of IHGs (outcome) by CMV serostatus (predictor) in overall, younger (Y, age<60 years), and older (O, age≥60 years). *P* values determined by Fisher's exact test for overall differences or IHG-II vs. rest or IHG-IV vs, rest in the indicated comparisons.
- Here, we first determined whether there were differences in IHG distributions by CMV serostatus in the overall cohort (All). Since the differences by CMV serostatus were significant (*P*<0.001) in the overall group, we performed a post-hoc analysis to determine whether similar differences were observed in the younger and older group, and whether these differences were attributable to changes in IHG-II and/or IHG-IV. Since the *P* values within each analysis were highly significant, a Bonferroni corrected *P* value would remain significant. Additionally, we did not compare whether the changes between younger vs. older were different. For these reasons, additional tests for multiple comparisons were not warranted.

o *Panel* e:
- Data derived from COVID-19 cohort.
- Distribution of IHG (outcome), hospitalization rates, and CMV seropositivity rate by age strata (predictor). $P_{IHG\text{-}I}$ and $P_{IHG\text{-}IV}$ is for the change in proportion of IHG-I vs. rest and IHG-IV vs. rest across age strata. *P* values determined by Fisher's exact test. Note: CMV seropositivity rates are based on 496 patients with available CMV serostatus.
- There are two *P* values presented in this panel, each addressing a specific hypothesis, i.e., whether akin to what is observed in individuals without COVID-19, the prevalence of IHG-I decreases whereas the prevalence of IHG-IV increases with age in patients with COVID-19. Since, we are testing two separate hypotheses, a test for multiple comparisons were not warranted. Moreover, since comparisons between IHG-I and IHG-IV were not made, additional testing for multiple comparisons was not warranted.

o *Panel* f:
- Data derived from COVID-19 cohort.
- Distribution of IHGs (outcome) at presentation in the overall COVID-19 cohort, by sex, hospitalization status, survivor status, age, and disease severity indexed by WHO ordinal scale strata (predictors). *P* values determined by Fisher's exact test.
- Three separate hypotheses (differences in IHGs by sex, hospitalization status, survivorship, WHO ordinal scale strata), were tested with one *P* value for each comparison. All results are reported, and additional testing for multiple comparisons was not warranted.

o *Panel* g:
- Data derived from RTR cohort and SLE cohort
- *Left*: Distribution of IHGs (outcome) in 114 renal transplant recipients (RTR) .
- *Right*: Distribution of IHGs (outcome) in 53 patients with SLE (source: GSE49454).

o *Panel* h:
- Data derived from HIV+ PIC cohort.
- *Left*: Distribution of IHGs (outcome) by entry viral load (VL) strata (predictor) in 592 participants from the PIC cohort. *P* values determined by χ^2 test.

- *Right*: Distribution of IHGs (outcome) at baseline and during ART treatment course (predictors) for a subset of PIC cohort (*n*=75). *P* values determined by linear GEE model based on a binomial distribution with an AR1 correlation structure comparing the change in IHG-I vs. rest and the change in IHG-IV vs. rest over time.
- Analysis was performed in two separate subsets of the same cohort testing different hypotheses. *Left*, examines the overall differences in IHG distributions in therapy-naïve patients categorized according to HIV VL strata; an overall difference is reported, and additional testing for multiple comparison was not warranted. *Right*, tests two separate hypotheses, i.e., whether suppression of HIV-VL was associated with reconstitution of IHG-I and a decrease in the prevalence of IHG-I. Additional testing for multiple comparisons was not warranted.

- *Panel* i:
  - Schema. No statistical analysis was performed.
- *Panel* j:
  - Data derived from subset of HIV+ EIC participants who are therapy-naïve.
  - Distribution of IHGs (outcome) at entry and years since entry (predictor) who remained therapy-naïve. *P* value determined by χ^2 test.
  - One *P* value; no correction required.
- *Panel* k:
  - Data derived from HIV− FSW cohort.
  - *Top:* Distribution of IHGs (outcome) by the indicated factors (predictor). *P* values determined by χ^2 test.
  - *Bottom*: Percentage of FSW who had later HIV seroconversion (outcome) according to study groups (predictor) in the top panel. *P* values determined by logistic regression.
  - Groupings in top and bottom panels are by following predictors: duration of sex work (in years) strata, condom use, clients per week strata, clients per week minus condoms per week strata, behavioral activity score (BAS), direct STI score, indirect STI score, and total STI score.
  - Distinct hypotheses were tested, i.e., whether the extent of distinct correlates of sex work associated with changes in IHG status. Each comparison has one *P* value. All results are reported, and additional testing for multiple comparisons was not warranted.

11.3.4. Figure 4

- *Panel* a:
  - Data derived from HIV− FSW cohort using the first available CD4+ and CD8+ T-cell counts, CD4:CD8 ratio, and BAS during each 2 years intervals.
  - *Left*: Distribution of IHGs (outcomes) within each 2-year intervals (predictor) in 27 FSWs who remained HIV– for at least 10 years. *P* value determined by Fisher's exact test comparing window >0-≤2 vs. >8-≤10.
  - *Right*: Boxplots (center line, median; box, the interquartile range (IQR); whiskers, rest of the data distribution (±1.5 × IQR); and points, outliers greater than ±1.5 × IQR) of BAS (outcome) in each 2-year intervals (predictor). *P* value determined by linear GEE model with an AR1 correlation structure evaluating the change in BAS over indicated time windows.

        - Two different hypotheses were tested; *left and right*, depict whether a decrease in sex work associated with a reconstitution in IHG-I and BAS, respectively. One *P* value per hypothesis is reported; additional testing for multiple comparisons was not warranted.
    - *Panel* b:
        - Data derived from HIV− FSW cohort.
        - *Left*: Distribution of IHGs (outcome) at baseline and first available IHG at or after year 4 of follow-up (predictors) in FSW who remained HIV-seronegative for at least 4 years (*n*=101); IHG distribution (outcome) is depicted in the overall group of FSWs and by baseline IHG (predictor). The outcomes are the IHG distributions with the predictors being baseline and first available measurement at year 4. *P* values determined by χ^2 test.
        - *Right*: Boxplots (center line, median; box, the interquartile range (IQR); whiskers, rest of the data distribution (±1.5 × IQR); and points, outliers greater than ±1.5 × IQR) of BAS (outcomes) in 101 FSWs who remained HIV-seronegative for at least 4 years (predictor); data are for values at baseline and first available measurement on or after the fourth anniversary (predictors). *P* values determined by Wilcoxon signed-rank test.
        - Two different hypotheses similar to those shown in *Panel* a are tested. One *P* value per hypothesis is reported; additional testing for multiple comparisons was not warranted.
    - *Panel* c:
        - Data derived from HIV− FSW cohort.
        - *Left*: Distribution of IHGs (outcome) pre- and post- HIV seroconversion (predictor) in 43 FSWs (paired samples; predictor). *P* value determined by Fisher’s exact test.
        - *Right*: Distribution of IHGs (outcome) in Sooty Mangabeys stratified by SIV serostatus (non-paired samples; predictor). *P* value determined by χ^2 test.
        - Two different hypotheses are tested; overall differences in humans (*left*) and Sooty mangabeys (*right*) by lentiviral serostatus are tested. One *P* value per hypothesis is reported; additional testing for multiple comparisons was not warranted.
    - *Panel* d:
        - Data derived from SIV−/SIV+ sooty mangabeys.
        - Distribution of IHGs (outcome) in Sooty mangabeys stratified by SIV serostatus, sex and age strata (predictors). *P* values determined by Fisher’s exact test.
        - Two different hypotheses were evaluated, examining differences in IHG distributions in SIV− and SIV+ animals by sex and age. One *P* value per hypothesis is reported. Differences between SIV− and SIV+ animals were not examined. Additional testing for multiple comparisons was not warranted.
    - *Panel* e:
        - Data derived from SIV− Chinese rhesus macaques.
        - Distribution of IHGs (outcome) in SIV− Chinese Rhesus macaques overall, by sex, and age strata (predictors). *P* values determined by Fisher’s exact test.
        - Two different hypotheses akin to *Panel* d were tested. Additional testing for multiple comparisons was not warranted.
    - *Panel* f:
        - Data derived from CC-RIX mice cohort.

- Distribution of IHGs (outcome) by indicated infectious outcome groups (predictor) in uninfected counterparts of CC-RIX mice infected with Ebola. *P* value determined by Fisher's exact test comparing mice groups lethal vs. resistant to Ebola infection.
- One *P* value; no correction required.

- *Panel* g:
  - Data derived from COVID-19 cohort.
  - *Top*: Schema. No statistical analysis was performed.
  - *Middle*: Forest plots depicting the odds ratio (OR) with 95% CI of hospitalization (outcome) from two separate logistic regression models both adjusted by age strata as indicated in **Supplementary Fig. 7** (21-39, 40-49, 50-59, 60-69, ≥70 yrs): Hospitalization by IHG and hospitalization by CMV serostatus (predictors).
  - *Bottom*: Plots depciting the hazard ratio (HR) with 95% CI of 30-day mortality (outcome) from two separate Cox proportional hazards models both adjusted by age strata as indicated in **Supplementary Fig. 7** (21-39, 40-49, 50-59, 60-69, ≥70 yrs): Mortality by IHG and mortality by CMV serostatus (predictors).
  - Point estimates and confidence intervals are reported, with no comparisons between the study groups. Additional testing for multiple comparisons was not warranted.
- *Panel* h:
  - Data derived from RTRs cohort.
  - Kaplan-Meier (KM) plot depicting time to second cutaneous squamous cell carcinoma (CSCC; outcome) in a group of 40 RTRs with CSCC in the preceding year (as detailed previously[5]) and was re-analyzed for CSCC-free (i.e., time to second CSCC during study follow-up) by IHG (predictor). *P* value determined by log-rank test.
  - One *P* value; no correction required.
- *Panel* i:
  - Data derived from HIV+ EIC participants.
  - KM plot depicting time to AIDS (CDC 1993 criteria; outcome) and the median entry CD4+ T-cell counts, CD8+ T-cell counts, CD4:CD8 ratios, and $\log_{10}$ VL (outcomes) by entry IHG (predictors). *P* value for KM plot were determined by log-rank test. *P* values for difference in VL from IHG-I to other IHGs were determined by linear regression.
  - Two separate hypotheses are tested, reporting the first with one *P* value, and the second with three *P* values from a single model testing whether the VL in patients presenting with a non-IHG-I grade differs from that of IHG-I. All *P* values in the latter model were <0.001 and remain significant after Bonferroni correction.
- *Panel* j:
  - Data derived from HIV+ PIC cohort.
  - Boxplots (center line, median; box, the interquartile range (IQR); whiskers, rest of the data distribution (±1.5 × IQR); and points, outliers greater than ±1.5 × IQR) of entry $\log_{10}$ VL levels (outcome) by entry IHG status (predictor). *P* values determined by Wilcoxon rank-sum test.
  - Three *P* values are reported; the two significant *P* values were <0.001 and remain significant after Bonferroni correction.

- *Panel* k:
  - Data derived from HIV+ EIC cohort.
  - Boxplots (center line, median; box, the interquartile range (IQR); whiskers, rest of the data distribution (±1.5 × IQR); and points, outliers greater than ±1.5 × IQR) of $\log_{10}$ VL levels (outcome) by entry IHG-I vs. IHG-II/III/IV (rest) of the cohort, and among participants with entry IHG-I stratified by those preserving IHG-I vs. switching from IHG-I to another IHG during each year therapy-naïve follow-up (predictors) The data illustrate the first IHG with an available coincident VL measurement during the indicated time window. *P* value determined by Wilcoxon rank-sum test.
  - A single hypothesis across six different timepoints was tested to determine whether VL differed between individuals who preserved IHG-I vs. had the other grades. A consistent pattern was observed across timepoints, and additional testing for multiple comparisons was not warranted.

11.3.5. Figure 5

- *Panel* a:
  - Data derived from HIV− FSW cohort.
  - Distribution of IHGs (outcome) in 449 FSWs by their behavioral activity score (BAS) categorized as less than 0, equal to 0, or greater than 0 (predictor) and according to whether they did vs. did not become HIV+ later (predictor). *P* value determined by χ^2 test.
  - Two different hypotheses with one *P* value each were examined; additional testing for multiple comparisons was not warranted.
- *Panel* b:
  - Data derived from HIV− FSW cohort.
  - Logistic regression was used to determine the OR with 95% CI of having IHG-III or IHG-IV at baseline and/or future HIV seroconversion (outcomes) by BAS, total STI scores, and IHGs (predictors). Three separate models were used.
  - Coefficients with 95% CI separate logistic regression models are depicted. No *P* values are shown. Additional testing for multiple comparisons was not warranted.
- *Panel* c:
  - Data derived from (i) Literature review for the proportion of CD8-CD4 disequilibrium (i.e., CD4:CD8 ratio <1.0 as a proxy for IHG-III or IHG-IV; outcome) as listed in **Supplementary Table 2**, and (ii) participants from HIV– SardiNIA, HIV–UCSD and HIV– FSWs as listed in Fig. 1a. Percentage of participants with IHG-III/IV (outcome) by indicated groups (predictor). All *P* values were determined by χ^2 test (except for Tetanus vaccine data where Fisher's exact test was used) for comparing difference in proportion of IHG-III or IHG-IV within the specified study groups.
  - This was an exploratory survey performed to determine whether the indicated factors (e.g., age, sex, varied inducers, or outcomes) associated with differences in the proportions in individuals with vs. without IHG-III or IHG-IV. All results are reported. We did not compare for differences across different study groups. Additional testing for multiple comparisons was not warranted.

- *Panels* d-f:
  - Schema. No statistical analysis was performed.

11.3.6. Figure 6

- *Panel* a:
  - Schema. No statistical analysis was performed.
- *Panel* b:
  - Data derived from HIV− SardiNIA cohort.
  - Distribution of IHG subgrades (outcome) by age strata (predictor). *P* value determined by Fisher's exact test.
  - One *P* value; no correction required.
- *Panel* c:
  - Data derived from COVID-19 cohort.
  - Distribution of IHG subgrades (outcome) in overall (*n*=541) and at baseline and at convalescence in 220 COVID-19 patients (predictor). *P* value determined by Fisher's exact test.
  - One *P* value; no correction required.
- *Panel* d:
  - Data derived from multiple cohorts.
  - *Left-most*: Distribution of IHG subgrades (outcome) in SLE cohort (source: GSE49454). No statistical analysis was performed.
  - *Left*: Distribution of IHG subgrades (outcome) in 114 RTRs.
  - *Right*: Distribution of IHG subgrades (outcome) in HIV+ PIC cohort pre-ART and during ART (predictor). *P* value determined by Fisher's exact test.
  - *Right-most*: Distribution of IHG subgrades (outcome) at entry by BAS<0 vs. ≥0 (predictor) in participants from HIV− FSW cohort. *P* value determined by Fisher's exact test.
  - One *P* value per comparison of IHG distributions across two cohorts, and no comparisons made between cohorts. Additional testing for multiple comparisons was not warranted.
- *Panel* e:
  - Schema. No statistical analysis was performed.

11.3.7. Figure 7

- *Panel* a:
  - Schema. No statistical analysis was performed.
- *Panel* b:
  - All analyses were performed in the FHS offspring cohort (Source: dbGaP: phs000007.v30.p11; median age [IQR] years = 66 [60-73]; females: 54%) and all *P* values were determined by χ^2 test.
  - *Left*: Distribution of SAS-1/MAS-1 profiles (outcome) in participants stratified by sex (predictor). *P* values were determined for differences in proportions of the SAS-1/MAS-1 profiles and the differences in proportion of SAS-1^{high}-MAS-1^{low} or SAS-1^{low}-MAS-1^{high} vs. rest between males vs. females.
  - *Right*: Distribution of SAS-1/MAS-1 profiles (outcome) in participants stratified by indicated age bins (predictor). *P* values were determined for differences in

proportions of the SAS-1/MAS-1 profiles and the differences in proportion of SAS-1^{high}-MAS-1^{low} or SAS-1^{low}-MAS-1^{high} vs. rest overall across the indicated age bins.

  - There are four distinct hypotheses being tested: differences in SAS-1/MAS-1 profiles by sex and by age, and whether age is associated with an increase in the SAS-1/MAS-1 profiles that associated with mortality and a decrease in the profile that is associated with survival. Additional testing for multiple comparisons was not warranted.

- *Panel* c:
  - All analyses were performed in the San Antonio Family Heart Study (Source: E-TABM-305) and all *P* values were determined by χ^2 test.
  - *Left*: Distribution of SAS-1/MAS-1 profiles (outcome) in participants stratified by sex (predictor).
  - *Middle*: Distribution of SAS-1/MAS-1 profiles (outcome) in participants stratified by indicated age bins (predictor). *P* values were determined for differences in proportions of the SAS-1/MAS-1 profiles and the differences in proportion of SAS-1^{high}-MAS-1^{low} or SAS-1^{low}-MAS-1^{high} vs. rest overall across the indicated age bins.
  - *Right*: Distribution of SAS-1/MAS-1 profiles (outcome) in participants stratified by indicated age bins and sex (predictor). *P* values were determined for differences in proportions of the SAS-1/MAS-1 profiles by sex within each indicated age bins.
  - This series of analyses were hypothesis driven and performed to corroborate findings shown in *Panel* a in a larger dataset across a wider age range. All *P* values are reported, and the overall group differences by sex are highly significant. The far-right panel depicts whether group level differences in sex are evident across age strata. Additional testing for multiple comparisons was not warranted.
- *Panel* d:
  - All analyses were performed in the Alzheimer's disease (AD) and other dementia disorders cohort.
  - Distribution of SAS-1/MAS-1 profiles (outcome) in first four groups*:* Pooled sets of control groups [set 1 (*n*=249; median age [IQR] years = 73 [69 -78]; females: 56%; source: GSE140829) and set 2 (*n*=281; median age [IQR] years = 72 [68-77]; females: 56%; source: GSE140830)] stratified by indicated age bins (predictor).
  - Distribution of SAS-1/MAS-1 profiles (outcome) in last four groups*:* Pooled sets of disease groups [set 1 includes patients with AD and mild cognitive impairment (*n*=338; median age [IQR] years = 72 [68-79]; females: 51%; source: GSE140829) and set 2 includes patients one or multiple disorders within the frontotemporal dementia (*n*=261; median age [IQR] years = 66 [60-72]; females: 51%; source: GSE140830) were pooled together and stratified by indicated age bins (predictor).
  - *P* values were determined using GEE model with an exchangeable correlation structure, binomial family, and ANOVA for comparing differences in proportions of SAS-1^{high}-MAS-1^{low} vs. rest across indicated all age bins within control or disease groups. *P* values determined using GEE model with an exchangeable correlation structure, and binomial family for comparing differences in proportions of SAS-1^{high}-MAS-1^{low} vs. rest between indicated age bins of control vs. disease groups.
  - Specific hypotheses are tested within and across two distinct datasets differing by disease status. Additional testing for multiple comparisons was not warranted.

- *Panel* e:
  - All analyses were performed in the SLE dataset (GSE49454).
  - *Left to right*: Distribution of SAS-1/MAS-1 profiles (outcome) in healthy controls stratified by median age of the overall cohort (median age [IQR] years = 39 [30-51]; females: 85%) and SLE patients stratified by IHG subgrades (predictor). Note: Healthy controls and SLE patients in IHG-I were further stratified by median age of the overall cohort (Younger/Older). For this analysis, only first available laboratory (CD4+ count and CD4:CD8 ratio) measurements and corresponding gene expression measurement from patients with SLE was used.
  - *P* values were determined using Fisher's exact test for comparing differences in proportions of SAS-1/MAS-1 profiles between IHG-I (pooling younger and older) vs. IHG-II (pooling IHG-IIa/b/c subgrades) and IHG-I (pooling younger and older) vs. IHG-IV (pooling IHG-IVa/b/c subgrades).
  - Two *P* values are shown to depict differences in SAS-1/MAS-1 profiles in patients with IHG-II or IHG-IV vs. IHG-I. Additional testing for multiple comparisons was not warranted.
- *Panel* f:
  - Data derived from COVID-19 cohort.
  - Distribution of SAS-1/MAS-1 profiles (outcome) in COVID-19 patients stratified by baseline IHG subgrades (predictor).
  - *P* values were determined using Fisher's exact test for comparing differences in proportions of SAS-1/MAS-1 profiles across IHG subgrades as well as between IHG-I vs. IHG-II (pooling IHG-IIa/b/c subgrades) and IHG-I vs. IHG-IV (pooling IHG-IVa/b/c subgrades).
  - Group level differences in IHG status were significant, and two post hoc comparisons were performed to corroborate findings in *Panel* e using an acute COVID-19 cohort. Additional testing for multiple comparisons was not warranted.
- *Panel* g:
  - Data derived from HIV+ EIC cohort.
  - Distribution of SAS-1/MAS-1 profiles in participants with HIV on ART (EIC subset) stratified by IHG subgrades at the time of experiment and spontaneous virologic controllers (SVC) not on ART. Note: For this analysis, HIV– participants (n=3) and HIV+ participants not on ART (n=3) in the dataset were excluded from the plot.
  - *P* values were determined using Fisher's exact test for comparing differences in proportions of SAS-1/MAS-1 profiles between IHG-I vs. IHG-II (pooling IHG-IIa/b subgrades), IHG-I vs. IHG-IV (pooling IHG-Iva/b subgrades), and IHG-I vs. SVC.
  - Two comparisons performed to corroborate findings in *panel* e using an HIV cohort; additional testing for multiple comparisons was not warranted. A single comparison between IHG-I and SVCs was performed.
- *Panel* h:
  - Data derived from COVID-19 cohort.
  - Distribution of SAS-1/MAS-1 profiles (outcome) in COVID-19 patients stratified by age, hospitalization and 30-day survival categories (predictor). *P* values determined by Fisher's exact test for indicated comparisons.

        - Two separate hypotheses tested comparing differences in profiles by IHG status and COVID-19 outcomes. Single *P* value was reported for each comparison. Additional testing for multiple comparisons was not warranted.
    - *Panel* i:
        - Data derived from multiple cohorts.
        - *Left*, COVID-19: Distribution of SAS-1/MAS-1 profiles (outcome) in COVID-19 patients (source: COVID-19 cohort) stratified by baseline IHG status (predictor).
        - *Middle*, SLE: Distribution of SAS-1/MAS-1 profiles (outcome) in SLE patients (source: GSE49454) stratified by first IHG measurement (predictor).
        - *Right*, Therapy-naïve: Distribution of SAS-1/MAS-1 profiles (outcome) in 5 uninfected controls (*n*=10 samples), and HIV+ patients classified into three groups (predictors): 9 asymptomatic HIV+ patients (*n*=18 samples); 9 acute HIV+ patients (*n*=16 samples); and 4 AIDS patients (*n*=8 samples) (Source: GSE16363, median age [IQR] years = 39 [32-45]; females: 15%). Note: Two samples from each donor or patient were available in GEO and all samples were used in the analysis.
        - *P* values for first two panels were determined using Fisher's exact test for comparing proportions of SAS-1^{high}-MAS-1^{low} vs. rest between IHG-I vs. rest. *P* value for right panel was determined using GEE model with an exchangeable correlation structure, binomial family, and ANOVA for comparing differences in proportions of SAS-1^{high}-MAS-1^{low} vs. rest across indicated groups.
        - Single *P* value per comparison in three separate cohorts. Left and middle comparisons are corroborative analyses; comparison for the right panel is for change in IHG-I distributions. Additional testing for multiple comparisons was not warranted.
    - *Panel* j:
        - Schema. No statistical analysis was performed.

11.3.8. Figure 8

- *Panel* a:
    - Data derived from FHS offspring cohort (Source: dbGaP: phs000007.v30.p11; median age [IQR] years = 66 [60-73]; females: 54%).
    - KM plot depicting proportion survived (outcome) over 9-year follow-up time in FHS participants stratified by SAS-1/MAS-1 profiles (predictors). *P* value determined by log rank test.
    - One *P* value; no correction required.
- *Panel* b:
    - Data derived from FHS offspring cohort (Source: dbGaP: phs000007.v30.p11; median age [IQR] years = 66 [60-73]; females: 54%)
    - Distribution of SAS-1/MAS-1 profiles (outcome) in FHS participants stratified by survival status (predictor). *P* values were determined using χ^2 test for differences in proportions of the SAS-1/MAS-1 profiles and the differences in proportion of SAS-1^{high}-MAS-1^{low} or SAS-1^{low}-MAS-1^{high} vs. rest between survivors vs. non-survivors.
    - Group level differences by survivorship in SAS-1/MAS-1 profiles were significant, followed by specific post-hoc testing whether there were differences in the extremes of the SAS-1/MAS-1 profiles by survivorship status.

- *Panel* c:
  - Schema. No statistical analysis was performed.
- *Panel* d:
  - Data derived from sepsis cohorts.
  - *Left*: Distribution of SAS-1/MAS-1 profiles [outcome] in the meta-analysis of patients with severe sepsis due to community acquired pneumonia (CAP) or fecal peritonitis (FP) admitted to the intensive care unit with either sepsis response signature 1 (SRS1, G1) or sepsis response signature 2 (SRS2, G2) [predictor] (Sources: E-MTAB-4421, median age [IQR] years = 64 [52-75], females: 46%; E-MTAB-4451, median age [IQR] years = 73.5 [63.5-80.0], females: 26%; E-MTAB-5273, *n*=147, median age [IQR] years = 66 [52.5-76.0], females: 50%; and E-MTAB-5274, *n*=106, median age [IQR] years = 71 [62.2-77.0], females: 36%) and healthy controls. Note: G1 (SRS1) is associated with higher mortality than G2 (SRS2); the SRS1 and SRS2 were defined using baseline samples at admission into the intensive care unit. All the four datasets used for this analysis were from same microarray platform.
  - *P* values were determined using Fisher's exact test comparing differences in proportions of SAS-1/MAS-1 profiles between G1 vs. G2 within patients with CAP and FP.
  - Analysis was performed in two separate cohorts with a single comparison per cohort; comparisons across cohorts were not performed. Additional testing for multiple comparisons was not warranted.
  - *Right*: Distribution of SAS-1/MAS-1 profiles [outcome] in patients with sepsis (*n*=43), post-surgical patients with septic shock who are non-survivors (septic shock NS; *n*=17), post-surgical patients with septic shock who are survivors (septic shock S; *n*=22), patients with systemic inflammatory response syndrome (SIRS; *n*=58) and healthy controls (normal; *n*=15) [predictor] (Source: E-MTAB-1548). Median age [IQR] years of patients (excluding the healthy controls) in the study = 72 [62-79]; females among patients (excluding the healthy controls): 28%.
  - *P* values were determined using χ^2 test for comparing differences in proportions of SAS-1/MAS-1 profiles across all groups as well as within disease groups and GEE model with an exchangeable correlation structure, binomial family, and ANOVA for comparing differences in proportions of SAS-1^{high}-MAS-1^{low} vs. rest or SAS-1^{low}-MAS-1^{high} vs. rest across all groups. Note: For determination of overall or within disease groups *P* value using χ^2 test, the repeated measurements from the same individual were considered as unique. In the calculation of *P* value for SAS-1^{high}-MAS-1^{low} vs. rest, the septic shock NS group was removed as the proportion of SAS-1^{high}-MAS-1^{low} in this group was zero. In the calculation of *P* value for SAS-1^{low}-MAS-1^{high} vs. rest, the controls group was removed as the proportion of SAS-1^{low}-MAS-1^{high} in this group was zero and SIRS group was used as reference in this analysis.
  - In this cohort, an overall difference was observed in SAS-1/MAS-1 profiles; post-hoc testing with three comparisons within the sepsis group was performed to illustrate changes across the sepsis spectrum. Additional testing for multiple comparisons was not warranted.

- *Panel* e:
  - Data derived from natural viral infection cohort (GSE68310; age range: 18-49 years)
  - *Left*): Distribution of SAS-1/MAS-1 profiles [outcomes] in 133 adults at enrollment (baseline: B; Group 1) and followed longitudinally during a seasonal acute respiratory illness (days 0, 2, 4, 6, 21; Groups 2-6) and the following spring (SP; Group 7) the next year [predictor] (Source: GSE68310, females: 52%). *P* values were determined using GEE model with an exchangeable correlation structure and binomial family for comparing differences in proportions of SAS-1^{low}/MAS-1^{high} vs. rest between baseline vs. Day 0, and Day 0 vs. spring timepoints.
  - *Middle to Right*: Distribution of SAS-1/MAS-1 profiles (outcome) in participants presenting with a SAS-1^{high}/MAS-1^{low} (*middle*) and SAS-1^{low}/MAS-1^{high} (*right*) and followed longitudinally. *P* values were determined by Fisher's exact test for comparing distributions between the two SAS-1/MAS-1 profile groups (SAS-1^{high}/MAS-1^{low} vs. SAS-1^{low}/MAS-1^{high}) at the indicated timepoints.
  - *Left,* two hypotheses were tested: ARI causes a shift in SAS-1/MAS-1 profiles, and SAS-1/MAS-1 profiles are restored during convalescence. One *P* value per comparison, and additional testing for multiple comparisons was not warranted. *Middle and right*: Based on findings in the left panel, post-hoc we examined whether the changes in SAS-1/MAS-1 profiles during convalescence differed by the SAS-1/MAS-1 profile that antedated ARI. Four of the five significant *P* values from six comparisons remain significant after Bonferroni correction and do not meaningfully impact on interpretation (Table below).

Comparison at time	*P* value	P_{adj} (Bonferroni)
Day 0	0.4332 (ns)	1 (ns)
Day 2	0.0482 (*)	0.2893 (ns)
Day 4	0.0049 (**)	0.0296 (*)
Day 6	<0.001 (***)	<0.001 (***)
Day 21	<0.001 (***)	<0.001 (***)
Spring	0.0025 (**)	0.0151 (*)

- *Panel* f:
  - Schema. No statistical analysis was performed.
- *Panel* g:
  - Data derived from viral challenge study (GSE17156).
  - Distribution of SAS-1/MAS-1 profiles [outcome] in healthy volunteers (18-49 year old) inoculated experimentally with H3N2, RSV, or rhinovirus (infections pooled for analysis) at baseline (T1) and when peak symptoms (T2) developed after inoculation stratified by symptom status [predictor] (Source: GSE17156). *P* values were determined using GEE model with an exchangeable correlation structure and binomial family for comparing differences in proportions of SAS-1^{low}/MAS-1^{high} vs. rest between asymptomatics vs. symptomatics at baseline as well as peak time point.

- Comparisons are made in two separate groups with distinct phenotypes. Single comparison per group. Additional testing for multiple comparisons was not warranted.
- *Panel* h:
  - Data derived from viral challenge study (GSE52428).
  - Distribution of SAS-1/MAS-1 profiles [outcome] evaluated following intranasal inoculation with influenza A (H1N1 and H3N2, pooled for analysis) in healthy persons who became symptomatic at baseline (T1; -24 hr. and 0 hr. pooled) and peak symptoms (T2; 60 hr. and 69.5 hr. pooled) [predictor (Source: GSE52428, median age [IQR] years = 25 [23-28]; females: 37%)]. Note: Timepoint 60 and 69.5 hours were selected as peak timepoint based on highest IFN response in symptomatic participants after which the IFN response achieved a plateau, and these two time points was the midpoint of the study. *P* values were determined using GEE model with an exchangeable correlation structure and binomial family for comparing differences in proportions of SAS-1^{low}/MAS-1^{high} vs. rest between asymptomatic vs. symptomatic individuals at baseline as well as peak time point.
  - Comparisons are made in two separate groups with distinct phenotypes. Single comparison per group. Additional testing for multiple comparisons was not warranted.
- *Panel* i:
  - Data derived from hospitalized influenza study (source: GSE111368)
  - *Left*: Distribution of SAS-1/MAS-1 profiles [outcome] in healthy controls and patients hospitalized with severe influenza collected at T1 (recruitment), T2 (approx. 48hr after T1), and T3 (at least 4 weeks after T1) [predictor] (source: GSE111368; median age [IQR] years = 72 [67-76]; females: 54%). *P*-values were determined by GEE model with an exchangeable correlation structure and binomial family for comparing differences in proportions of SAS-1^{high}/MAS-1^{low} vs. rest between HC and T1, and between T1 and T3.
  - *Middle* to *Right*: Distribution of SAS-1/MAS-1 profiles [outcome] in patients stratified by severity, time, and age < or ≥40 years: 18-39 year old (*middle*) and 40-71 year old (*right*) [predictor]. Descriptive analysis. No statistical analysis was performed.
  - Two separate hypotheses are tested requiring comparisons in profile distributions between health controls vs. acute infection, and changes in profiles at baseline (T1) vs. recovery (T3). Additional testing for multiple comparisons was not warranted.

11.3.9. Figure 9

- *Panels* a-b:
  - Schema. No statistical analysis was performed.
- *Panel* c:
  - Data derived from subset of HIV+ EIC cohort.
  - Correlation (r; Pearson correlation coefficient) [outcomes] between expression levels of representative genes within the SAS-1 or MAS-1 scores with levels of T-cell responsiveness (orange), T-cell dysfunction (green), and plasma IL-6 (blue) [predictors]. Correlation coefficient (r), and *P* values determined by correlation test

using Pearson's method; and thresholds of r values at false discovery rate (FDR; Benjamini-Hochberg) <0.05 are shown in **Supplementary Data 10**. Measures of T-cell responsiveness, T-cell dysfunction and plasma IL-6 were from 55, 56 and 50 HIV+ persons, respectively.

- *Panel* d:
  - Data derived from SIV+ sooty mangabeys.
  - Boxplots (center line, median; box, the interquartile range (IQR); whiskers, rest of the data distribution (±1.5 × IQR); and points, outliers greater than ±1.5 × IQR) of the indicated immune traits (outcome) by IHG (predictor). *P* values determined by Wilcoxon rank-sum test.
  - Three different hypotheses are being tested here. The first is the %cell type differs by IHG-I and II vs. IHG-III and IV. The second is the %cell type differs by IHG-I vs. IHG-III. The third is the %cell type differs by IHG-II vs. IHG-IV. These comparisons are made for specific cell types and not across cell types. Additional testing for multiple comparisons was not warranted.
- *Panel* e:
  - Data derived from SIV– Chinese Rhesus macaques.
  - Boxplots (center line, median; box, the interquartile range (IQR); whiskers, rest of the data distribution (±1.5 × IQR); and points, outliers greater than ±1.5 × IQR) of percent PD1+CD8+ cells (outcome) by age and IHG status (predictor]. *P* values determined by Kruskal-Wallis test across the indicated groups.
  - Two different hypotheses are tested reporting one *P* value per comparison. Additional testing for multiple comparisons was not warranted.

11.3.10. Figure 10

- *Panel* a:
  - Data derived from SardiNIA cohort.
  - 75 Immune traits sub-grouped into 19 signatures that classify into four groups, as described in Supplementary Information Section 11.2.
- *Panel* b:
  - Data derived from SardiNIA cohort.
  - Boxplots (center line, median; box, the interquartile range (IQR); whiskers, rest of the data distribution (±1.5 × IQR); and points, outliers greater than ±1.5 × IQR) of the indicated immune traits (outcome) by age (<40 and ≥70) within IHG-I and IHG-II and by IHGs (predictor). The trait levels are presented as inverse-normalized residuals after adjusting for sex and/or age using linear regression. *P* values determined by Wilcoxon rank-sum test and corrected for multiple comparisons using Bonferroni method. Detailed methods are as described in Supplementary Information Section 11.2.
- *Panel* c*:*
  - Data derived from SardiNIA cohort.
  - Log_2 Levels of CD127-CD8^{bright} T-cells and CD28-CD8^{bright}T-cells (outcome) across age stratified by IHGs (predictors). Analysis was performed using linear regression with log_2 cell counts as the outcome and age and IHG as predictors. Fitted lines, 95% confidence band, and *P* values determined using linear regression and are corrected for multiple comparisons using FDR.

- *Panel* d:
  - Schema. No statistical analysis was performed.

11.4. Details of statistical methods and analytical strategy per supplementary figure panel

11.4.1. Supplementary Fig. 1

- *Panels* a-d:
  - Schemas of Study cohorts. No statistical analysis was performed.

11.4.2. Supplementary Fig. 2

- *Panel* a:
  - Data derived from COVID-19 cohort
  - Schema of gene score derivation and analysis. The methods are detailed in Supplementary Information Section 8.2. No statistical analysis was performed.
- *Panel* b:
  - Data derived from FHS offspring cohort (Source: dbGaP: phs000007.v30.p11; median age [IQR] years = 66 [60-73]; females: 54%)
  - KM plots depicting proportion survived (outcome) over 9-year follow-up time in the FHS participants stratified by quartiles (first through fourth, Q1-Q4) of indicated gene signature scores (predictors). *P* values determined by log-rank test.
  - Three different hypotheses are tested with one *P* value per comparison. Additional testing for multiple comparisons was not warranted.
- *Panel* c:
  - Data derived from cohort of Kenyan HIV− children with Schistosomiasis.
  - Boxplots (center line, median; box, the interquartile range (IQR); whiskers, rest of the data distribution (±1.5 × IQR); and points, outliers greater than ±1.5 × IQR) of percent CD25+CD127–CD4+ (outcome) by *S. haematobium* egg counts (predictor). *P* value determined by Kruskal-Wallis test.
  - One *P* value; no correction required.

11.4.3. Supplementary Fig. 3

- *Panels* a-d:
  - All data derived from HIV− FSWs cohort.
- *Panel* a:
  - Schema of participant subsets. No statistical analysis was performed.
- *Panel* b:
  - Similar to **Fig. 3k**
  - *Top*: Distribution of IHGs (outcome) by the indicated factors (predictor). *P* values determined by χ^2 test.
  - *Bottom*: Percentage of FSW who had later HIV seroconversion (outcome) according to study groups (predictor) in the *top* panel. *P* values determined by logistic regression.
  - Groupings in *top* and *bottom* panels are by following predictors: duration of sex work (in years) strata, condom use, clients per week strata, clients per week minus

condoms per week strata, behavioral activity score (BAS), direct STI score, indirect STI score, and total STI score.
    - These analyses mirror those in main Fig. 3k but in a larger group of FSWs. Separate hypotheses were tested, i.e., whether the extent of distinct correlates of sex work associated with changes in IHG status. Each comparison has one *P* value. All results are reported, and additional testing for multiple comparisons was not warranted.
- *Panel* c:
    - Barplots depicting the proportion of each value of the indirect (*top left*), direct *(top right*), and total (*bottom*) STI score (outcomes) by the BAS (predictor). Spearman's correlation coefficient is shown at the top. *P* values determined by a test statistic based on Spearman's product moment correlation coefficient.
    - Three separate correlations between distinct correlates of sex work are shown. Additional testing for multiple comparisons was not warranted.
- *Panel* d:
    - KM plot depicting proportion free of IHG-III or IHG- (outcome) over time by baseline BAS (predictor) in FSW that were IHG-I or IHG-II at baseline. *P* value determined by log-rank test.
    - One *P* value; no correction required.

11.4.4. Supplementary Fig. 4

- *Panels* a-d:
    - All data derived from HIV− FSWs cohort.
- *Panel* a: Similar to **Fig. 4a**, except that all data for indicated follow-up windows are shown in boxplots using the first available CD4+ and CD8+ T-cell counts, CD4:CD8 ratio and accompanying BAS data during each 2-year intervals.
    - *Left*: Distribution of IHGs (outcome) within each 2-year intervals (predictor) in 27 FSWs who remained HIV– for at least 10 years. *P* value determined by Fisher's exact test comparing window >0-≤2 vs. >8-≤10.
    - *Middle*: Boxplots (center line, median; box, the interquartile range (IQR); whiskers, rest of the data distribution (±1.5 × IQR); and points, outliers greater than ±1.5 × IQR) of BAS (outcome) in each 2-year intervals (predictor). *P* value determined by linear GEE model with an AR1 correlation structure evaluating the change in BAS over indicated time windows.
    - *Right*: Boxplots (center line, median; box, the interquartile range (IQR); whiskers, rest of the data distribution (±1.5 × IQR); and points, outliers greater than ±1.5 × IQR) of CD4+, CD8+ T-cell counts, and CD4:CD8 ratio (outcomes) in each 2-year intervals (predictors). *P* value determined by linear GEE model with an AR1 correlation structure evaluating the change in $\log_2$ transformed data over indicated time windows.
    - This panel examines three hypotheses and parallels the findings shown in main **Fig. 4a**: IHGs over time, BAS over time, and clinical metrics over time. A single *P* value per comparison is shown. Additional testing for multiple comparisons was not warranted.
- *Panel* b:
    - Same as **Fig. 4b,** left.

- Distribution of IHGs (outcome) at baseline and first available IHG at or after year 4 of follow-up (predictors) in FSW who remained HIV-seronegative for at least 4 years (*n*=101); IHG distribution (outcome) is depicted in the overall group of FSWs and by baseline IHG (predictor). The outcomes are the IHG distributions with the predictors being baseline and first available measurement at year 4. *P* values determined by χ^2 test, testing if the pooled distributions of IHG-I and IHG-II differ from pooled distributions of IHG-III and IHG-IV.
- One *P* value; no correction required.

o *Panel* c:
- Similar to **Fig. 4b**, right, except that all data for indicated timepoints are shown in boxplots.
- Boxplots (center line, median; box, the interquartile range (IQR); whiskers, rest of the data distribution (±1.5 × IQR); and points, outliers greater than ±1.5 × IQR) of BAS, CD4+, CD8+ T-cell counts, and CD4:CD8 ratio (outcomes) in 101 FSWs who remained HIV-seronegative for at least 4 years (predictor); data are for values at baseline and first available measurement on or after the fourth anniversary (predictors). *P* values determined by Wilcoxon signed-rank test.
- This panel examines two hypotheses: BAS over time and clinical metrics over time. Single *P* value per comparison is shown. Additional testing for multiple comparisons was not warranted.

o *Panel* d:
- *Top*: Median values of CD8+ cell counts (outcomes) by comparison groups indicated in bottom panel (predictor). *P* values determined by Wilcoxon signed-rank test.
- *Bottom*: similar to Supplementary **Fig. 4b**, except that the data is shown for baseline and year 6. Distribution of IHGs (outcome) at baseline and first available IHG at year 6 of follow-up (predictor) in FSW who remained HIV-seronegative for at least 6 years (*n*=73); IHG distribution (outcome) is depicted in the overall group of FSWs and by baseline IHG (predictor). The outcomes are the IHG distributions with the predictors being baseline and first available measurement at year 6. *P* value for overall was determined by χ^2 test. *P* value for baseline IHG-III comparison group was determined by Fisher's exact test.
- This panel examines two hypotheses: comparing CD8+ counts overall and by IHG status, and reconstitution of IHGs according to baseline IHG. Additional testing for multiple comparisons was not warranted.

11.4.5. Supplementary Fig. 5

o *Panels* a-c:
- All data derived from subset (*n*=43) of HIV+ FSW.

o *Panel* a:
- Distribution of post-infection IHGs (outcome) by IHG-III/IV (CD8-CD4 disequilibrium) vs. IHG-I/II (CD8-CD4 equilibrium) at baseline (predictor) in 43 FSWs who have longitudinal data while HIV− and at least one CD4+ and CD8+ T-cell counts measurement within 1 year of HIV seroconversion. No statistical analysis was performed.

- *Panel* b:
  - Distribution of post-infection IHGs (outcome) by pre-infection IHGs (predictor) in 43 FSW who acquired HIV. Pre-infection IHGs were calculated using the first available CD4+ T-cell counts and CD4:CD8 ratio before HIV seroconversion for each subject. Post-infection IHGs were calculated using the first available CD4+ T-cell counts and CD4:CD8 ratio after HIV seroconversion for each subject. No statistical analysis was performed.
- *Panel* c:
  - Pre- vs. post-HIV seroconversion changes in IHG status (predictor) within 43 FSWs who acquired HIV with accompanied percent change in CD4+, CD8+ T-cell counts, and ratio (outcomes) between first available measurements before and after HIV seroconversion. The time interval between baseline IHG to the first available IHG after seroconversion was 4.70 (2.40-7.05) years. CD8+ expansion was classified as suppressed, restrained, and yes based on % change in CD8+ T-cell counts of <–20, –20 to 20, and >20, respectively. No statistical analysis was performed.

11.4.6. Supplementary Fig. 6
- Data derived from sooty mangabeys.
- Distribution of IHGs (outcome) in sooty mangabeys stratified by SIV serostatus and age strata (predictors). *P* values determined by Fisher's exact test.
- Two separate hypotheses tested; single *P* value per comparison. Additional testing for multiple comparisons was not warranted.

11.4.7. Supplementary Fig. 7
- Data derived from COVID-19 cohort. Note: COVID-19 patients who are IHG-III at baseline were excluded from both analysis.
- *Left*: IHG-adjusted OR of hospitalization (outcome) stratified by categorical age (predictor) from logistic regression model adjusted by IHG status.
- *Right*: IHG-adjusted HR of 30-day mortality (outcome) stratified by categorical age (predictor) from Cox proportional hazards model adjusted by IHG status.
- Two separate hypotheses tested. Point estimates with CI reported. Additional testing for multiple comparisons was not warranted.

11.4.8. Supplementary Fig. 8
- Data derived from RTR cohort.
- Distribution of CD57+CD8+ high/low groups (outcome) by IHG (predictor). Each participant was dichotomized into high/low CD57+CD8+ group on the basis of a majority (i.e., >50%, referred to as CD57+ high) or minority (CD57+ low) of CD57+ cells within the CD8+ population. *P* value determined by Fisher's exact test.
- One *P* value; no correction required.

11.4.9. Supplementary Fig. 9
- Data derived from HIV− SardiNIA cohort.
- The fitted line and 95% confidence band derived from linear regression for (*left to right*) $\log_2$ transformed values of CD4+, CD8+ T-cell counts, and CD4:CD8 ratio

(outcome) versus age (predictor) stratified overall (top row) and by sex (middle and bottom rows). R^2 and P values for $\log_2$ transformed data versus age determined by linear regression. To evaluate the differences between change in CD4+ T-cell count across age vs. change in CD8+ T-cell count across age, a linear GEE model with an exchangeable correlation structure of the $\log_2$ cell counts (both CD4 and CD8) as outcomes, and age, cell type (CD4 or CD8), and an interaction term between age and cell type was used. The *P* value for the interaction term shown if the change in CD8+ T-cell counts across age differed from the change in CD4+ T-cell counts across age.

- We examined trajectories of different clinical metrics in overall, males, and females with age. These constitutes three different hypotheses. Additional testing for multiple comparisons was not warranted.

11.4.10. Supplementary Fig. 10

- All data derived from HIV− FSWs cohort.
- *Panel* a:
  - Distribution of future HIV seroconversion status (outcome) by BAS strata, STI score strata, and IHGs with subgrades (predictors). *P* values determined by χ^2 test.
  - Three separate hypotheses are tested: whether BAS, STI and IHG status associated with HIV serostatus. For BAS and STI analysis single comparisons are performed. For IHG analysis, the hypothesis tested was whether increasing deviations from IHG-I associated with HIV+ serostatus. All significant *P* values remain significant after Bonferroni correction. Additional testing for multiple comparisons was not warranted.
- *Panel* b:
  - aOR with 95% CI of future HIV seroconversion (outcome) determined using logistic regression by BAS strata, STI score strata, and IHGs with subgrades (predictors). BAS strata are adjusted by STI score strata and IHGs with subgrades. STI score strata is adjusted by BAS strata and IHGs with subgrades. IHGs with subgrades is adjusted by BAS strata and STI score strata.
  - Statistical plan same as *Panel* a but examining the adjusted odds ratio of incident HIV. Three separate hypotheses are tested: whether BAS, STI and IHG status associated with HIV serostatus. For BAS and STI analysis single comparisons are performed. For IHG analysis, the hypothesis tested was whether increasing deviations from IHG-I associated with HIV+ serostatus. The odds ratios were derived from a single model. Additional testing for multiple comparisons was not warranted.

11.4.11. Supplementary Fig. 11

- Data derived from multiple cohorts.
  - Unsupervised hierarchical clustering of SAS-1, MAS-1, IMM-AGE, and age in the FHS-offspring cohort (*left*), San Antonio Family Heart Study (*middle*), COVID-19 cohort (*right*) performed using 1 – Pearson's correlation coefficient as the distance with Ward's D linkage. *P* value determined by correlation test using Pearson's method.

- All comparisons are within three separate cohorts. There are no inter-cohort comparisons. Additional testing for multiple comparisons was not warranted.

11.4.12. Supplementary Fig. 12

- *Panel* a:
  - All data derived from FHS-offspring cohort (Source: dbGaP: phs000007.v30.p11; median age [IQR] years = 66 [60-73]; females: 54%)
  - *Top*: Line plots of SAS-1 and MAS-1 scores (mean ± SEM) [outcomes] in the FHS cohort stratified by categorical age and sex [predictors]. *P* values determined by linear model comparing males vs. females adjusting for categorical age.
  - *Bottom*: Line plots of SAS-1 and MAS-1 scores (mean ± SEM) [outcomes] in the San Antonio Family Heart Study stratified by categorical age and sex [predictors]. *P* values determined by linear model comparing males vs. females adjusting for categorical age.
  - Three separate associations are described with a single *P* value per gene signature score in two separate cohorts. There are no inter-cohort comparisons. Additional testing for multiple comparisons was not warranted.
- *Panel* b:
  - Boxplots (center line, median; box, the interquartile range (IQR); whiskers, rest of the data distribution (±1.5 × IQR); and points, outliers greater than ±1.5 × IQR) of indicated gene signature scores (outcomes) in meta-analysis of Alzheimer's disease (AD) and other dementia disorders cohorts.
  - *Left*: Two sets of control groups [set 1 (*n*=249; median age [IQR] years = 73 [69 - 78]; females: 56%; source: GSE140829) and set 2 (n= 281; median age [IQR] years = 72 [68-77]; females: 56%; source: GSE140830)] were pooled together and stratified by indicated age bins (predictors).
  - *Right*: Two sets of disease groups [set 1 includes patients with Alzheimer's disease and mild cognitive impairment (*n*=338; median age [IQR] years = 72 [68-79]; females: 51%; source: GSE140829) and set 2 includes patients one or multiple disorders within the frontotemporal dementia (*n*=261; median age [IQR] years = 66 [60-72]; females: 51%; source: GSE140830) were pooled together and stratified by indicated age bins (predictors).
  - *P* values determined by GEE model with an exchangeable correlation structure for and ANOVA overall comparison of the groups.
  - Three separate gene signature scores were examined and a single *P* value per signature is shown. Additional testing for multiple comparisons was not warranted.
- *Panel* c:
  - Boxplots (center line, median; box, the interquartile range (IQR); whiskers, rest of the data distribution (±1.5 × IQR); and points, outliers greater than ±1.5 × IQR) of indicated gene signature scores (outcomes) in meta-analysis of whole blood samples from/of the following groups:
  - *Left*: 518 HIV− participants from Finnish DILGOM cohort stratified into indicated age bins (predictor). (Source: E-TABM-1036). *P* value determined by ANOVA for overall comparison of the age bins.

- *Middle-Left*: Participants from USA who are HIV−, HIV+ without ART, or HIV+ with ART (predictors). (Source: GSE29429). *P* values determined by GEE model with an exchangeable correlation structure comparing between indicated groups.
- *Middle-Right*: Participants from UK who are HIV− healthy control, and patients with LTBI or PTB (predictors). (Source: GSE19439, GSE19444). *P* value determined by Welch's t-test between indicated groups.
- *Right*: Participants from Africa who are HIV− healthy controls, and patients with LTBI or PTB, and HIV+ without ART (predictors). (Source: GSE29429). *P* values between control and HIV+ groups determined by GEE model with an exchangeable correlation structure.
- Four different cohorts are evaluated for levels of three gene signature scores. In each cohort the association of distinct variables with score expression was determined. The differences in the significance values relate to the underlying biology (e.g., age is associated with a decrease in SAS-1, or patients with PTB and HIV have higher antigenic loads). Additional testing for multiple comparisons was not warranted.

11.4.13. Supplementary Fig. 13

- *Panel* a:
  - All data derived from FHS-offspring cohort (Source: dbGaP: phs000007.v30.p11; median age [IQR] years = 66 [60-73]; females: 54%) and all *P* values were determined using χ^2 test for indicated overall comparisons.
  - *Left*: Distribution of SAS-1/MAS-1 profiles (outcome) in participants stratified by sex (predictor).
  - *Middle*: Distribution of SAS-1/MAS-1 profiles (outcome) in participants stratified by indicated age bins (predictor). *P* values were determined for overall differences in proportions of the SAS-1/MAS-1 profiles and the differences in proportion of SAS-1^{high}-MAS-1^{low} or SAS-1^{low}-MAS-1^{high} vs rest across the indicated age bins.
  - *Right*: Distribution of SAS-1/MAS-1 profiles (outcome) in participants stratified by indicated age bins and sex (predictors). *P* values were determined for differences in proportions of the SAS-1/MAS-1 profiles between males vs females within each of the indicated age bins.
  - This series of analyses was hypothesis driven and performed to corroborate findings in **Fig. 7c** (main text). Overall differences in SAS-1/MAS-1 profiles by sex and age examined individually were highly significant. The stratified analysis was performed to determine the pattern of sex and age combinations remained consistent. Differences between age/sex strata were not examined. Additional testing for multiple comparisons was not warranted.
- *Panel* b:
  - All data derived from Finnish DILGOM cohort (Source: E-TABM-1036) and *P* values were determined using χ^2 test for indicated overall comparisons. Note: Data presented in this panel is an excerpt from the meta-analysis of datasets described in Supplementary Information Section 8.3.12.
  - *Left*: Distribution of SAS-1/MAS-1 profiles (outcome) in participants stratified by sex (predictor).

    - *Middle*: Distribution of SAS-1/MAS-1 profiles (outcome) in participants stratified by age strata (predictor). *P* values were determined for overall differences in proportions of the SAS-1/MAS-1 profiles and the differences in proportion of SAS-1^{high}-MAS-1^{low} or SAS-1^{low}-MAS-1^{high} vs. rest across the indicated age strata.
    - *Right*: Distribution of SAS-1/MAS-1 profiles (outcome) in participants stratified by indicated age strata and sex (predictors). *P* values were determined for differences in proportions of the SAS-1/MAS-1 profiles between males vs. females within each of the indicated age strata.
    - This series of analyses was hypothesis driven and performed to corroborate findings in **Fig. 7c** (main text). Overall differences in SAS-1/MAS-1 profiles by sex and age examined individually were highly significant. The stratified analysis was performed to determine the pattern of sex and age combinations remained consistent. Differences between age/sex strata were not examined. Additional testing for multiple comparisons was not warranted.
- *Panel* c:
    - Data derived from multiple cohorts.
    - Boxplots (center line, median; box, the interquartile range (IQR); whiskers, rest of the data distribution (±1.5 × IQR); and points, outliers greater than ±1.5 × IQR) of indicated gene signature scores (outcomes) in the following groups (predictor):
    - *Left*: HIV+CMV+ EIC subjects on ART by IHG-I vs. rest.
    - *Middle-left*: HIV− SLE patients by IHG-I vs. rest.
    - M*iddle-right*: COVID-19 patients by IHG-I vs. rest.
    - *Right*: COVID-19 patients by IHG status.
    - *P* values for the data shown in first three columns were determined by Welch's t-test for comparison of the IHG-I vs. rest.
    - *P* values for the data shown in last column was determined by ANOVA for overall comparison of the IHG groups.
    - This panel tests three hypothesis in three different cohorts: differences in SAS-1, MAS-1 and IMM-AGE in patients classified as IHG-I vs. rest. Single *P* value per comparison is shown. Since the overall difference in the COVID-19 cohort was significant, a post-hoc analysis was performed comparing IHG-I vs. IHG-II or IHG-II. Additional testing for multiple comparisons was not warranted.
- *Panel* d:
    - All data derived from FHS-offspring cohort (Source: dbGaP: phs000007.v30.p11; median age [IQR] years = 66 [60-73]; females: 54%)
    - KM plots depicting proportion survived (outcome) over 9-year follow-up time in the FHS participants stratified by sex with SAS-1 and MAS-1 profiles by age (*top*, younger; *bottom*, older). *P* values determined by log-rank test.
    - Single *P* value are reported per comparison for SAS-1 and MAS-1. The scores were associated with time to death as a continuous variable after application of an FDR (**Supplementary Data 9a**). Additional testing for multiple comparisons was not warranted.
- *Panel* e:
    - Data derived from 90+ Vitality cohort (source: GSE65218 and GSE65219)
    - *Left*: Distribution of SAS-1/MAS-1 profiles [outcome] in nonagenarian (*n*=146, ages: ≥90 years; females: 71%) and young (*n*=30, median age [IQR] years = 22.5

[20.2-24.0]; females: 70%) participants stratified by sex [predictor] (Source: GSE65219, *n*=176). *P* value determined by Fisher's exact test for comparing overall distributions of SAS-1/MAS-1 profiles between nonagenarians vs. young.
    - *Right*: KM plots depicting the proportion survived [outcome] over 4 years follow-up in nonagenarian (*n*=151) participants stratified by median based low/high categorical groups of (*left*) SAS-1 and (*right*) MAS-1 scores [predictors]. (Source: GSE65218, ages: ≥90 years; females: 70%). *P* values determined by log-rank test.
    - There are two different hypotheses being tested and single *P* value per comparison are reported. Additional testing for multiple comparisons was not warranted.
- *Panel* f:
    - Data derived from multiple sepsis cohorts
    - Distribution of SAS-1/MAS-1 profiles [outcome] in the meta-analysis of patients with severe sepsis due to community acquired pneumonia (CAP) or fecal peritonitis (FP) admitted to the intensive care unit with either sepsis response signature 1 (SRS1, G1) or sepsis response signature 2 (SRS2, G2) [predictor] (Sources: E-MTAB-4421, median age [IQR] years = 64 [52-75]; females: 46%; E-MTAB-4451, median age [IQR] years = 73.5 [63.5-80.0]; females: 26%; E-MTAB-5273, *n*=147, median age [IQR] years = 66 [52.5-76.0]; females: 50%; and E-MTAB-5274, n=106, median age [IQR] years = 71 [62.2-77.0]; females: 36%) and healthy controls. Note: G1 (SRS1) is associated with higher mortality than G2 (SRS2); the SRS1 and SRS2 were defined using baseline samples at admission into the intensive care unit. All the four datasets used for this analysis were from same microarray platform.
    - *P* values were determined using Fisher's exact test comparing differences in proportions of SAS-1/MAS-1 profiles between G1 vs G2 within patients with CAP and FP.
    - There are four cohorts being examined. The results of the grouped analysis according to condition (CAP or FP) are reported in **Fig. 8d**. These analyses were performed to show the results in the individual cohorts. We did not perform cross-cohort comparisons. Additional testing for multiple comparisons was not warranted.
- *Panel* g:
    - Data derived from burn cohort (GSE182616)
    - Distribution of SAS-1/MAS-1 profiles [outcomes] in burn injury patients longitudinally sampled through hospitalization stratified by total burn surface area ≤20% or >20% [predictors] (Source: GSE182616; median [IQR] age of 39 [30.5-55.0] years). *P* values were determined using GEE model with an exchangeable correlation structure, binomial family, and ANOVA for changes in proportion of SAS-1^{high}-MAS-1^{low} or SAS-1^{low}-MAS-1^{high} vs. rest over time.
    - Two hypothesis were tested in two separate groups of burn patients, examining whether recovery is associated with changes in SAS-1/MAS-1 profiles. Two *P* values per hypothesis is shown. Additional testing for multiple comparisons was not warranted.
- *Panel* h:
    - Data derived from sepsis cohort (GSE185263)

- Distribution of SAS-1/MAS-1 profiles [outcomes] in healthy controls (*n*=44, median [IQR] age = 51 [29-59] years; females = 59%) and hospitalized patients with sepsis (*n*=348, median [IQR] age = 61 [44-72] years; females = 42%) stratified by mortality and SOFA score [predictors] (Source: GSE185263). SOFA categorical bins were defined as having a SOFA score of 0 (*n*=95), score of 1 (*n*=43), score of 2 (*n*=57), score of 3 (*n*=39), score of 4 (*n*=29), score of 5 (*n*=20), score of 6-7 (*n*=20), score of 8-12 (*n*=29), or a score of 13-16 (*n*=13). *P* values determined by Fisher's exact test.
- Two separate hypotheses were examined, testing SAS-1/MAS-1 profile distributions according to survival and SOFA status. Single *P* value per comparison. Additional testing for multiple comparisons was not warranted.

o *Panel* i: pre-CC mice cohort
- Distributions of SAS-1/MAS-1 profiles [outcome] in pre-CC mice by high or low response (predictor] to influenza infection. *P* value determined by Fisher's exact test.
- Only one *P* value; no correction required.

11.4.14. Supplementary Fig. 14

o Data derived from SIV– Chinese Rhesus macaques.
- Boxplots (center line, median; box, the interquartile range (IQR); whiskers, rest of the data distribution (±1.5 × IQR); and points, outliers greater than ±1.5 × IQR) of percent PD1+CD4+ (*left*) and PD1+CD8+ (*right*) [outcomes] by age, IHG, and age & IHG [predictor]. *P* values determined by Kruskal-Wallis test across the indicated groups.
- Two separate hypotheses were tested, examining trait levels by age and IHG status. Single *P* value reported per comparison. Additional testing for multiple comparisons was not warranted.

11.4.15. Supplementary Fig. 15

o Flow cytometry gating strategy for T-B-NK cell antibody panel
o No statistical analysis was performed.

11.4.16. Supplementary Fig. 16

o Flow cytometry gating strategy for Treg antibody panel
o No statistical analysis was performed.

11.4.17. Supplementary Fig. 17

o Flow cytometry gating strategy and traits assessment related to the maturation stages of T-cell antibody panel
o No statistical analysis was performed.

11.4.18. Supplementary Fig. 18

o Flow cytometry gating strategy and traits assessment related to the circulating dendritic cell (DC) antibody panel.
o No statistical analysis was performed.

11.4.19. Supplementary Fig. 19

- Data derived from SardiNIA cohort.
- *Panel* a: Group 1
  - Distribution of IHGs (outcome) in the previous analysis of the SardiNIA cohort stratified by age and sex and age (predictor). *P* values determined by χ^2 test.
- *Panel* b: Group 2
  - Distribution of IHGs (outcome)in the updated analysis of the SardiNIA cohort stratified by age and sex and age (predictor). *P* values determined by χ^2 test.
- *Panel* c: Comparison
  - Distribution of IHGs in the previous (Group 1) and updated (Group 2) analysis of the SardiNIA cohort stratified by age. *P* values determined by χ^2 test.
- These analyses in *Panels* a to c were performed to quality control the two set of SardiNIA cohorts analyzed and corroborate the modeling data presented in **Fig. 2g** (main text). Additional testing for multiple comparisons was not warranted.

11.4.20. Supplementary Fig. 20

- *Panel* a:
  - Data derived from HIV– UCSD cohort, acute COVID-19, and COVID-19 convalescent (left to right).
  - *Top*: Median CD8+ cell counts, CD4+ cell counts, and CD4:CD8 ratio by CMV serostatus (outcomes) in overall and by IHG subdivided by CD4:CD8 ratio (predictors). *P* values comparing CD8+ T-cell counts by CMV serostatus determined using Wilcoxon rank sum test.
  - *Bottom*: Distribution of CMV serostatus (outcome) in overall and by IHG subdivided by CD4:CD8 ratio (predictors). *P* values determined by χ^2 test.
  - Two separate hypotheses are tested, examining differences in CD8+ counts by IHG status and CMV serostatus by IHG status in three different cohorts. No cross-cohort comparisons were performed. Additional testing for multiple comparisons was not warranted.
- *Panel* b:
  - Data derived from HIV– UCSD cohort, acute COVID-19, and COVID-19 convalescent (left to right).
  - Distribution of CMV serostatus (outcome) by age strata (predictor). *P* values determined by χ^2 test.
  - In three different cohorts, the association of age and CMV serostatus across age is examined. Cross-cohort comparisons were not made. Single *P* value per cohort is reported. Additional testing for multiple comparisons was not warranted.
- *Panel* c:
  - Data derived from HIV– UCSD cohort, acute COVID-19, and COVID-19 convalescent (left to right).
  - Line plots depicting the % CMV+ vs. Age strata in those with a CD4:CD8 ratio <1.75 and those with a CD4:CD8 ratio ≥ 1.75. *P* values comparing proportion CMV+ by CD4:CD8 ratio within each age strata were determined by χ^2 test.
  - In three different cohorts, the association of CD4:CD8 ratio with CMV serostatus across age is examined. The focus was on the consistency of the pattern across

age and cross-cohort comparisons were not made. Additional testing for multiple comparisons was not warranted.

- *Panel* d:
  - Data derived from RTR cohort
  - Distribution of CMV serostatus (top) and CD57+CD8+ high/low (bottom) [outcomes] by IHG (left) or IHG subdivided by CD4:CD8 ratio (right) [predictors]. Each participant was dichotomized into high/low CD57+CD8+ trait on the basis of a majority (i.e., >50%, referred to as CD57hi) or minority (CD57lo) of CD57+ cells within the CD8+ population. *P* value determined by Fisher's exact test.
  - Two separate hypotheses are tested, examining the association of trait levels and CMV serostatus by IHG status. Single *P* value per comparison is shown. Additional testing for multiple comparisons was not warranted.
- *Panel* e:
  - Data derived from HIV– UCSD cohort.
  - Distribution of IHGs (outcome) by CMV serostatus and urine drug test status (predictors). *P* values determined by χ^2 test, testing if the pooled distributions of IHG-I and IHG-II differ from the pooled distributions of IHG-III and IHG-IV.
  - Two separate hypotheses were tested, examining differences in IHG distributions by CMV serostatus, after controlling for drug use, and differences in IHG distributions in CMV+ persons with vs. without positive urine drug test. Single *P* value reported for each comparison. Additional testing for multiple comparisons was not warranted.
- *Panel* f:
  - Data derived from HIV– UCSD cohort, acute COVID-19, and COVID-19 convalescent (left to right).
  - Distribution of IHGs (outcome) by sex and CMV serostatus (predictors). *P* values comparing females vs. males within CMV+ was determined by Fisher's exact test.
  - In three different cohorts/groups of individuals, differences in IHG distributions by sex in CMV+ persons were examined. A single *P* value per comparison is reported. Additional testing for multiple comparisons was not warranted.

11.4.21. Supplementary Fig. 21

- Same as **Fig. 4f** except an additional schema about the groups is presented in the right.
  - Distribution of IHGs (outcome) by indicated infectious outcome groups (predictor) in uninfected counterparts of CC-RIX mice infected with Ebola. *P* value determined by Fisher's exact test comparing mice groups lethal vs. resistant to Ebola infection.
  - One *P* value; no correction required.

11.4.22. Supplementary Fig. 22

- *Panel* a:
  - Schema. No statistical analysis was performed.
- *Panel* b:
  - Data derived from HIV− UCSD cohort.
  - Distribution of rs2524054 genotypes (outcome) by IHG subdivided by CD4:CD8 ratio groupings (predictors) in overall (left) and in European Americans only (right). *P* value determined using a trend test in which the number of minor alleles for

rs2524054 was used as the dependent variable and the indicated IHG ratio groups as a continuous variable (in the same order as depicted in the figure) in a linear model. The group order shown is based on highest to lowest CD8 counts within each ratio strata.
    - One *P* value; no correction required.
- *Panel* c:
    - Data derived from HIV− UCSD cohort.
    - Distribution of CMV serostatus (outcome) by indicated CD4:CD8 ratio groupings (predictor). *P* values determined by χ^2 test.
    - A single hypothesis was tested, examining whether decreasing ratio values associated with increasing CMV seropositivity status. Additional testing for multiple comparisons was not warranted.

11.4.23. Supplementary Fig. 23

- *Panel* a:
    - Data derived from SardiNIA cohort.
    - The n, median, and IQR for CD4+, CD8+ T-cell counts, and CD4:CD8 ratio (outcomes) in participants with ages 15-39 (younger) or ≥70 (older) years old by IHG (predictors) with the %difference between older and younger (outcome). *P* values determined by Wilcoxon rank-sum test.
    - Three separate hypotheses are tested, examining the differences in three distinct variables by age according to IHG status. Mechanistic hypothesis driven questions addressed. Additional testing for multiple comparisons was not warranted.
- *Panel* b:
    - Data derived from SardiNIA cohort
    - Line plots of median CD4+, CD8+ T-cell counts, and CD4:CD8 ratio (left to right; outcome) by age strata (predictor) in overall, males, and females.
- *Panel* c:
    - Data derived from SardiNIA cohort
    - Line plot of the median CD4:CD8 ratio (outcome) by age strata (predictor) in those with IHG-I or IHG-II.
- *Panel* d:
    - Data derived from HIV– UCSD cohort
    - Kernel density estimate plot of CD4:CD8 ratio. *P* value determined by using the Kolmogorov-Smirnov test evaluating the differences in distribution of CD4:CD8 ratio between participants with aged <40 vs. ≥40 years old.
- *Panel* e:
    - Data derived from SardiNIA cohort
    - Kernel density estimate plot of CD4:CD8 ratio. *P* value determined by using the Kolmogorov-Smirnov test evaluating the differences in distribution of CD4:CD8 ratio between participants with ages <40 vs. ≥40 years old.
    - Note: one participant with CD4:CD8 ratio >300 was eliminated from this analysis. The plot was trimmed at CD4:CD8 ratio cutoff of 7. The estimated density for participants aged <40 years old spans only till CD4:CD8 ratio of 6.84. However, for participants aged ≥40 years old, the CD4:CD8 ratio spans till 12.13. The tail of density distribution (between CD4:CD8 ratio of 7 to 12.13) in participants aged ≥40

years old accounts for only 1.4% of the population and was removed from the plot for visualization and head-to-head comparison with data presented in panel d.

- *Panels* d and e show results of a single hypothesis tested in two separate cohorts. Overall *P* value in differences in distributions (kernel density estimate plots by age) is depicted. Single *P* value per comparison is shown. Additional testing for multiple comparisons was not warranted.

11.4.24. Supplementary Fig. 24

- *Panel* a:
  - Data derived from SardiNIA cohort and same as **Fig. 10a**.
  - 75 Immune traits sub-grouped into 19 signatures that classify into four groups, as described in Supplementary Information Section 11.2.
- *Panel* b:
  - Data derived from SardiNIA cohort and similar to **Fig. 10b**, except that additional signatures are shown.
  - Boxplots (center line, median; box, the interquartile range (IQR); whiskers, rest of the data distribution (±1.5 × IQR); and points, outliers greater than ±1.5 × IQR) of the indicated immune traits (outcome) by age (<40 and ≥70) within IHG-I and IHG-II and by IHGs (predictor). The trait levels are presented as inverse-normalized residuals after adjusting for sex and/or age using linear regression.
  - *P* values determined by Wilcoxon rank-sum test and corrected for multiple comparisons using conservative Bonferroni method. Instead of using $P<0.05$, a stringent $P<1.67 \times 10^{-4}$ (adjusting for multiple comparisons: 75 traits compared x 4 comparisons: IHG-I vs. IHG-III; IHG-II vs. IHG-IV; younger vs. older within IHG-I and younger vs. older within IHG-II) was used to ascribe significance of traits as detailed in the Supplementary Information Section 11.2 of the supplementary information.

11.4.25. Supplementary Tables 1 to 2

- No statistical analysis was performed.

11.4.26. Supplementary Data 1 to 14

- *P* values were corrected for multiple comparisons where appropriate if the comparisons were all testing the same hypothesis or sample groups.

D. Supplementary Notes

Supplementary Note 1. Immunologic health grades (IHGs): rationale and mitigation of confounding

The generation of the IHGs were not derived arbitrarily. Rather, the derivation was based on deliberate deductions and a large body of prior data and observations as discussed below. The utility of the IHGs in assessment of immune status in a less-confounded manner is also discussed.

(i) Conundrums that prompted the generation of IHGs:
The concept that the high-grade CD8-CD4 equilibrium tracked by IHG-I may associate with proximal processes linked to immunocompetence emerged from conundrums that we reported in the *New England Journal of Medicine* (2013) and *JAMA Internal Medicine* (2015)[9,10]. These conundrums indicated that (a) the CD4+ count is an imperfect indicator of immunologic status and (b) age is an imperfect proxy for antigenic experience, such that younger, HIV-positive persons manifest disease observed more commonly in older, HIV-negative individuals (e.g., cancer, cardiovascular diseases).

(ii) IHGs track CD8-CD4 equilibrium/disequilibrium states:
IHGs were derived on the principles of immune allostasis (discussed in introduction) and that (a) optimal IR status tracks superior immunocompetence, (b) peripheral blood CD8+ and CD4+ T-cell levels influence immunity, (c) CD4+ lymphopenia is a feature of aging, SLE, and HIV infection[8,57,89-101], and (d) GWAS-identified polymorphisms that associate with CD4+ and CD8+ T-cell counts are distinct[30]. However, because we found that the absolute CD4+ count or ratio values alone were imperfect indicators of immune status, we surmised that the level of equilibrium vs. disequilibrium between peripheral blood CD8+ and CD4+ T-cells (CD8-CD4 equilibrium/disequilibrium states), rather than their absolute values, may be more predictive metrics of IR. We termed these CD8-CD4 equilibrium/disequilibrium states as immune health grades (IHGs; **Fig. 2b**).

(iii) Cutoff rationale:
We focused on CD8-CD4 equilibrium/disequilibrium states to capture the possibility that higher or lower levels of CD8+ expansion, with or without lower CD4+ counts, associates with varying levels of immunocompetence. To this end, IHGs were derived by co-indexing the CD4:CD8 T-cell ratio and CD4+ T-cell count using cutoffs selected *a priori* (**Fig. 2b**, Ref. [8]). We conducted a large literature survey of the CD4+ count of adults (males and females) with confirmed or presumed HIV-seronegativity[9,10]. We conducted this survey, as we sought CD4+ levels that represent a more normal range of the CD4+ count independent of age or sex. The conventional range for a normal CD4+ count is very wide (>350 cells/mm^3). The CD4+ cutoff level to derive IHGs was based on our finding in previous studies that the lower bounds of the interquartile range (IQR) of CD4+ counts in 16,126 HIV− persons was close to 800 cells/mm^3 (median: 952, IQR: 840 – 1,036; Ref. [9,10] and **Supplementary Table 1**).In HIV+ individuals, reconstitution of CD4+ counts to this cutoff associated with near-normalization of indicators of T-cell function[10]. An inverted CD4:CD8 ratio was used as a cut-off as it is a mathematical reflection of or proxy for higher CD8+ levels.

Thus, we use the CD4:CD8 T-cell ratio as a proxy for the level of CD8+ T-cells relative to CD4+ counts, as ratio values <1.0 are simply a mathematical representation of or proxy for higher levels of CD8+ T-cells that are uncompensated by higher levels of CD4+ counts. To generate the IHGs, we capitalized on this proxy function of ratio values <1.0 and the average CD4+ T-cell count found in otherwise healthy persons (800 cells/mm^3) (**Fig. 2b**). Hence, the grades are not representative of 4 categories of ratio values; instead, the IHGs track two CD8-CD4 equilibrium states represented by IHG-I and IHG-II and two CD8-CD4 disequilibrium states represented by IHG-III and IHG-IV.

(iv) IHG features:
The lowest and highest median CD8+ levels were in the IHG-II and IHG-III grades, respectively, whereas the highest and lowest median CD4+ levels were in the IHG-I and IHG-IV grades, respectively. Yet, the median CD4:CD8 T-cell ratios were relatively similar or identical in IHG-III and IHG-IV; IHG-I and IHG-II were characterized by similarly higher CD4:CD8 T-cell ratios (**Supplementary Data 3**). These findings indicate that CD8-CD4 equilibrium is distinct from the CD4:CD8 T-cell ratio. Since the CD4:CD8 T-cell ratio is a mathematical construct, persons with the same ratio could have divergent CD4+ and CD8+ counts and, thus, CD8-CD4 equilibrium or disequilibrium states. This divergence suggests that, although the CD4:CD8 ratio has been recommended as a biomarker of immune status[33-35], the biomarker functions of the ratio may be imprecise. Inspection of the CD4+ values by IHG status suggests that the CD4+ count may also have imprecise biomarker functions. For example, IHG-I and IHG-III would signify normal CD4+ counts (≥800 cells/mm^3). However, in IHG-III, CD8+ counts were nearly double those in IHG-I (**Supplementary Data 3**).

Thus, the IHGs track an equilibrium between CD8+ and CD4+ T-cells that cannot be intuited by inspection of the individual levels of these lymphocytes or the CD4:CD8 ratio.

- The IHGs track distinct CD8-CD4 profiles as follows: IHG-I (CD8^{lower}-CD4^{highest}), IHG-II (CD8^{lowest}-CD4^{lower}), IHG-III (CD8^{highest}-CD4^{higher}), and IHG-IV (CD8^{higher}-CD4^{lowest}) (**Supplementary Data 3**).
- Hence, CD8-CD4 equilibrium grades IHG-I and IHG-II indicate restrained CD8+ expansion, with higher and lower CD4+ counts, respectively; CD8-CD4 disequilibrium grades IHG-III and IHG-IV indicate unrestrained CD8+ expansion, with higher and lower CD4+ counts, respectively (hence, the terms equilibrium vs. disequilibrium).
- The importance of these distinctions is underscored by the fact that persons may have higher CD4+ counts with relatively lower (IHG-I) vs. higher (IHG-III) CD8+ counts; conversely, persons may have lower CD4+ counts with relatively lower (IHG-II) vs. higher (IHG-IV) CD8+ counts (**Supplementary Data 3**).
- Similarly, some persons with IHG-I or IHG-II may have identical higher ratio values, despite having contrasting CD8-CD4 profiles; similarly, some persons with IHG-III or IHG-IV may have identical lower ratio values, despite contrasting CD8-CD4 profiles (**Supplementary Data 3**).

Summary: The IHGs do not represent four CD4:CD8 ratio categories; rather, they represent the relative equilibrium between peripheral blood levels of CD8+ and CD4+ T-

cells. While the CD4+ count and the ratio are the conventional metrics of immune status, we note that their use may have resulted in a confounded assessment of immune status.

(v) IHGs as a metric of IR or immunocompetence:
In our model, IHG-I signifies high-grade equilibrium between CD8+ and CD4+ levels (**Fig. 2b**). Therefore, we posited that IHG-I would associate with proxies for superior immunocompetence. Below, we include our hypotheses and corresponding findings justifying the use of IHGs as gauges of immunocompetence.

a. *Prediction 1*: If IHG-I is an indicator of optimal IR (superior immunocompetence), then i) IHG-I is the most common grade in a general population irrespective of age and ii) increasing levels of antigenic stimulation should associate with progressive erosion of IHG-I, i.e., a switch from optimal to suboptimal or nonoptimal IR.

To assess this prediction, we evaluated the distribution of IHGs in cohorts in which participants were, as at the group-level, experiencing lower, moderate, and higher levels of antigenic stimulation [SardiNIA aging cohort < FSWs (female sex workers) < RTRs (renal transplant recipients) ~ SLE (systemic lupus erythematosus) ~ COVID-19 << HIV). Our results, support our proposition that IHG-I is the most common grade in human populations (primordial state) and that progressively higher levels of antigenic stimulation was associated with a progressive decline in persons preserving IHG-I and a reciprocal increase in persons manifesting a non-IHG-I grade signifying suboptimal or nonoptimal IR. Thus, the erosion of IHG-I was attributable to antigenic stimulation rather than age per se, and the erosion of IHG-I with age in the SardiNIA cohort suggests that age serves as an imperfect proxy for antigenic experience.

b. *Prediction 2:* IHG-I is reconstituted with mitigation of antigenic stimulation.

In the COVID-19, FSWs and HIV cohorts, we found that mitigation of antigenic stimulation (during COVID-19 convalescence, with reduction in sex work, and with antiretroviral therapy-induced suppression of HIV viremia, respectively) associated with reconstitution of IHG-I.

c. *Prediction 3:* IHG-I associates with immunoprotective effects in FSWs, patients with HIV infection, RTRs and persons with acute COVID-19.

The collective sum of our data suggests that presentation with IHG-I (baseline in respective cohorts) is associated with resistance against HIV seroconversion, AIDS progression, recurrence of cutaneous squamous cell cancer, and COVID-19-associated hospitalization and mortality.

d. *Prediction 4:* IHG-I associates with the transcriptomic metrics that associate with superior immunocompetence and lower inflammation.

We identified gene signatures (SAS-1 and MAS-1) whose expression levels associated with increased survival in persons with COVID-19 as well as in the

Framingham Heart Study, after controlling for age and sex (**Fig. 2d**). These gene expression signatures were enriched in persons with IHG-I, regardless of disease status (**Fig. 7e,f,g,i**). The gene composition of these gene signatures suggests that higher immunocompetence and lower inflammation linked to preservation of IHG-I is associated with increased survival rates (**Supplementary Data 9b-c**).

e. *Prediction 4:* IHG-I is more prevalent in females.

This prediction aligns with the observation that female sex associates with features of superior immunocompetence (longevity, resistance to some infections[102-106]). Consistent with this prediction, optimal IR (preservation of IHG-I) was greater in female vs. males in the SardiNIA, RTR, and COVID-19 cohorts.

f. *Prediction 5:* Confounding by conflation

Conflation of IHG-I or IHG-II (ratio ≥1.0) into a single category or conflation of IHG-III and IHG-IV into single category (inverted ratio) would have resulted in a confounded analysis as it combines grades with contrasting associations. For example, IHG-I and IHG-III are marked by higher CD4+ counts whereas IHG-II and IHG-IV are both defined by lower CD4+ counts yet these sets of IHGs were associated with divergent risks for AIDS.

Summary: For a metric of immune status to have value for prognostication in clinical medicine, it must have the fundamental capacity to differentiate persons with a normal vs. abnormal immune status when applied across age, sex or varied chronic conditions/infections. Our studies demonstrate that IHGs serve as an independent metric of immune status applicable in diverse conditions, after controlling for age and/or sex. It is this generalizable feature of IHGs that provided the basis to prognosticate immunity-dependent health outcomes, irrespective of age or sex.

Supplementary Note 2. Study design: strategies to mitigate confounding

To mitigate confounding, we undertook the following steps.

First, the immunologic framework, laboratory metrics, analytic plan, predictor-outcome dyads, and study groups were formulated *pre hoc*. In **Supplementary Note 1**, we discuss the basis to derive the immune health grades (IHGs) as metrics of IR status. Also discussed in **Supplementary Note 1** are the reasons why the IR metrics may mitigate confounding in association studies aimed at identifying the mechanistic (immunologic and transcriptomic) correlates of immunocompetence and inflammation that may mediate survival and other superior immunity-dependent outcomes (e.g., resistance to severe COVID-19 and HIV-AIDS).

Second, for corroboration and replication, we examined multiple cohorts in which proxies for the grade of antigenic stimulation could be estimated, including HIV− individuals experiencing low- and moderate-grade antigenic stimuli. Cumulative levels of antigenic

stimulation in these cohorts were controlled by comparing proxies for durations and/or levels of antigenic stimulation. These proxies included *Schistosoma haematobium* urine egg counts in children with schistosomiasis, behavioral risk factor score in female sex workers; duration of HIV infection by focusing on primary and early HIV infection cohorts; HIV viral load in HIV seropositive persons; and age as an imperfect proxy for antigenic experience in large HIV seronegative cohorts comprising participants from early adulthood to >100 years: Framingham Heart Study (*n*=2306), San Antonio Family Heart Study (*n*=1240); Finnish DILGOM Cohort (*n*=518), and COVID-19 cohort (*n*=541). The large sample size in which these proxies of antigenic experience were analyzed further mitigated confounding.

Third, to distinguish the health-influencing effects attributable to IR status vs. age, we juxtaposed the distributions of IR metrics (both laboratory and transcriptomic) in cohorts differing by age and cohorts in which participants were grouped according to defined levels of antigenic stimulation. Additionally, we examined cohorts in which the reversibility of eroded IR (non-IHG-I grade) and reconstitution of optimal IR (IHG-I) could be evaluated, mitigating the misattribution of the effects of IR status to age.

Fourth, evolutionary conservation of IR metrics and associations was examined in two non-human primate species (*Sooty mangabeys* and *Rhesus macaques*) and Collaborative Cross-RIX mice, a large panel of recombinant, inbred intercrosses (RIX) designed for complex trait analysis[107]. Analysis of this panel of mice permitted evaluation of whether IR status had a genetic component and whether genetically associated IR status influenced Ebola virus infection outcomes. Analysis of *Sooty mangabeys* with or without SIV infection permitted evaluation of a dose (one vs. two sources of antigenic stimulation, i.e., non-SIV vs. non-SIV with SIV, respectively) and response (increased rates of erosion of IHG-I) relationships.

Fifth, to minimize correlative associations, outcomes were examined in prospective cohorts that permitted an examination of whether cause (eroded IR signified by non-IHG-I grade and SAS-1/MAS-1 profiles other than SAS-1^{high}-MAS-1^{low}) antedated effect (outcomes). Thus, the renal transplant recipient cohort permitted an evaluation of the contribution of IR (IHG) status at time of initial diagnosis of cutaneous squamous cell cancer on rate of development of the second episode of cutaneous squamous cell cancer (**Fig. 4h**). The female sex worker cohort permitted an examination of whether HIV risk factor-associated antigenic stimulation associated with non-IHG-I grades, and whether pre-existing non-IHG-I grades increased risk of subsequent HIV seroconversion (**Fig. 5b**). The HIV infection cohorts permitted an examination of whether IR (IHG) status at baseline associated with rates of progression of AIDS (**Fig. 4i**). While we could not evaluate incident COVID-19 cohorts, the juxtaposition of the distributions and associations of IHG and transcriptomic metrics of IR status in cohorts with vs. without COVID-19 provided a framework to uncover the impact of IR status that may have antedated SARS-CoV-2 infection on COVID-19 outcomes (**Fig. 4g**). Additionally, we examined whether transcriptomic signatures that associate with COVID-19 mortality also influenced lifespan in persons without COVID-19 [in the Framingham Heart Study (FHS); **Fig. 2d,8a**]. This approach provided a non-confounded approach to identify immune and inflammatory

mechanistic traits that may antedate and contribute to COVID-19 survival. Finally, we evaluated prospective intranasal challenge studies with common respiratory viruses to determine whether the IR response following viral inoculation differentiated persons with asymptomatic vs. symptomatic infection (**Fig. 8g,h**).

Sixth, when examining immunologic traits shown in **Fig. 10a-c**, our study design accounted for potential confounding factors: (i) a comparison of immunologic traits between younger and older HIV− persons is a potentially confounded analysis, conflating four IHGs whose distributions vary to a greater extent by antigenic experience/stimulation than age, (ii) differences in trait levels between IHG-I vs. IHG-II or IHG-III vs. IHG-IV may be attributable to differences in CD4+ counts, and (iii) trait levels may differ by sex. For additional corroboration, we determined whether the immunologic features that associated with CD8-CD4 disequilibrium grades IHG-III or IHG-IV was similar in humans and nonhuman primates (Sooty mangabeys; **Fig. 9d**).

Seventh, to evaluate whether IR status has a genetic component, i.e., is not stochastic, we evaluated a single nucleotide polymorphism in the MHC that associated with lower CD8+ counts and a higher ratio[30]. In these genetic studies, we accounted for ethnicity and increasing levels of CD8+ levels in the context of IHGs. This approach mitigates the confounding that different IHGs may have similar CD8+ levels but with differing levels of CD4+ counts. Additionally, this approach mitigates the confounding that the association of this polymorphism relates to serostatus of cytomegalovirus (CMV).

Eighth, we accounted for the possibility that our findings could be confounded by CMV serostatus. We considered this possibility, as the laboratory metrics of IR status relate to the balance between peripheral blood CD8+ and CD4+ T-cells (**Fig. 2b**). Seropositivity for CMV increases with age and has been associated with CD8+ T-cell expansion and imbalances in CD8-CD4 profiles akin to CD8-CD4 disequilibrium IHG-III or IHG-IV grades. For example, the immune risk phenotype is characterized by an inverted CD4:CD8 T-cell ratio and CMV seropositivity; IHG-III and IHG-IV are also defined by an inverted ratio and signify nonoptimal IR status indexed to CD8-CD4 disequilibrium IHG-III or IHG-IV grades. Additionally, CMV seropositivity has been associated with adverse health outcomes[108-122], which conceivably could be attributable to nonoptimal IR vs. CMV seropositivity.

Ninth, comparing gene expression scores between cohorts may be affected by availability of genes, the median expression value of the genes within a study, batch effect, and microarray chip used. We accounted for these variables in our transcriptomic analyses.

Tenth, to mitigate the confounding that the gene signatures associated with survival may be tracking younger age and the female longevity advantage vs. IR status, in Cox proportional hazards models we identified gene signatures that associated with survival after controlling for age and sex. We then examined the association of these signatures with survival in sepsis cohorts and viral infection challenge cohorts.

Eleventh, we sought to identify in a non-confounded manner survival-associated mechanisms that independently associated with immunocompetence vs. inflammation. One argument could be that higher inflammation results in lower immunocompetence and vice versa. Another interpretation is that immunocompetence and inflammation are independent traits such that they have additive effects on survival. Our study design examined this possibility.

Twelfth, to differentiate whether the association of IR status were correlative vs. causal, we also focused on Hill's criteria for causation which are 10 criteria widely used in public health research to establish whether there is epidemiologic evidence of a causal relationship between a presumed cause and an observed effect (**Supplementary Note 11**).

Supplementary Note 3. CD8-CD4 equilibrium vs. disequilibrium and CMV serostatus

Higher CD8+ T-cell levels is viewed as a hallmark of CMV seropositivity[109,110,123,124]. Additionally, CMV seropositivity has been associated with mortality and a myriad of diseases[108-122]. However, our findings suggest that associations attributed to CMV seropositivity may instead relate to the susceptibility to develop CD8-CD4 disequilibrium. This possibility was examined in the control HIV– UCSD, COVID-19 and RTR cohorts as well as a large literature survey. The COVID-19 cohort allowed examination of the associations of CMV serostatus and the IHG grades that emerged during the acute stages of SARS-CoV-2 infection. These approaches permitted an evaluation of associations of IR status and CMV serostatus in constitutive (HIV– UCSD cohort) and inflammatory settings (COVID-19 and RTRs). In these analyses, we used the median CD4:CD8 ratio found in HIV– persons as a cut-off (1.75; **Supplementary Table 1**) to further stratify IHG-I or IHG-II; higher ratio values serve as a proxy for lower CD8+ T-cell counts.

Potential misattribution of CMV seropositivity as a cause of higher CD8+ levels. Results described below are aligned with the **Supplementary Fig. 20.**

A) HIV– UCSD cohort (non-COVID-19) cohort: (**Supplementary Fig. 20a,** left most)

At the overall cohort levels, consistent with conventional views, median CD8+ levels were higher in CMV+ vs. CMV– persons (582 vs. 442 cells/mm^3, respectively) (**Supplementary Fig. 20a**, leftmost). To further examine this relationship, we ordered the cohort according to increasing CD8+ levels (**Supplementary Fig. 20a,** left most). When ordered in this manner, while CMV seropositivity rates tracked CD8+ levels, CD8+ counts were not consistently statistically higher in CMV+ vs. CMV– persons; the only group in which CD8+ levels were higher was IHG-I with higher ratio values (481 vs. 434 cells/mm^3, respectively). IHG-II with higher ratio values had the lowest CMV seropositivity rates. IHG-III associated with the highest CMV seropositivity rates and CD8+ levels, yet CD8+ counts were only marginally higher in CMV+ vs. CMV– persons (1060 vs. 944 cells/mm^3, respectively); a similar pattern was observed with IHG-IV (721 vs. 704 cells/mm^3, respectively).

B) COVID-19 cohort: (**Supplementary Fig. 20a,** middle and right)

The overall association patterns between IHG status and CMV serostatus in the acute COVID-19 cohort during the acute and convalescent phases mirrored that of the UCSD cohort. CD4+ and CD8+ levels were higher in each of the groups during the convalescent vs. acute phases of COVID-19. Akin to the UCSD cohort, CMV seropositivity rates tracked CD8+ levels, and CD8+ counts were not higher in the CMV+ vs. CMV– persons in the group with the highest CMV seropositivity rates (IHG-III) in the COVID-19 cohorts.

C) Explained variability of CD8+ levels by CMV serostatus in the UCSD and COVID cohorts

While overall median CD8+ levels were higher in CMV+ persons, CMV serostatus explained a small proportion of the variability of CD8+ levels in the overall HIV– UCSD cohort (r^2=0.07; P<0.001; n=759). Similar results were observed in the early HIV infection cohort (r^2=0.01; P<0.001; n=3791), the acute COVID-19 cohort (r^2=0.07; P<0.001; n=496), and the convalescent COVID-19 cohort (r^2=0.06; P=0.001; n=203).

D) CMV serostatus by age in the UCSD and COVID-19 cohorts (**Supplementary Fig. 20b-c**).

CMV seropositivity increased with age. This age-associated increase was lower among persons with IHG-I or IHG-II maintaining higher ratio levels (proxy for lower CD8+ T-cells).

E) RTRs: **Supplementary Fig. 20d**

All renal transplant recipients with CD8-CD4 disequilibrium grades IHG-III or IHG-IV were CMV+ (**Supplementary Fig. 20d**). CMV seropositivity rates were also higher in person with IHG-I or IHG-II with lower ratio values. Proportions of CD57+CD8+ T-cells were higher in persons with CD8-CD4 disequilibrium grades IHG-III or IHG-IV.

F) CD8-CD4 disequilibrium status and CMV serostatus in conditions associated with increased antigenic stimulation: HIV– UCSD cohort: **Supplementary Fig. 20e.**

In the UCSD cohort, we considered a positive urine test for recreational drugs as a proxy of increased antigenic stimulation. After stratification of the cohort by CMV serostatus and urine drug test, we observed an increased rate of CD8-CD4 disequilibrium grades IHG-III or IHG-IV in CMV+ vs. CMV- persons, especially in CMV+ individuals with a positive urine drug test.

G) Sexual dimorphism: **Supplementary Fig. 20f** (HIV– UCSD and COVID-19 cohorts)

Rates of CD8-CD4 disequilibrium grades IHG-III or IHG-IV were elevated in CMV+ vs. CMV– persons, and sexual dimorphism was only observed in CMV+ persons: IHG-III and IHG-IV was overrepresented in CMV+ males vs. females.

Interpretation and discussion: While CMV seropositivity rates increased progressively with higher CD8+ levels, CD8+ counts between CMV+ and CMV– persons were not

statistically different between groups with the highest CMV seropositivity rates (IHG-III or IHG-IV). These findings favor the possibility that CMV seroconversion may relate to CD8-CD4 disequilibrium status, i.e., persons with a proclivity to restrict CD8+ expansion relative to CD4+ counts are more likely to restrict CMV seroconversion; this restriction is greatest in persons with the capacity to preserve IHG-II with higher ratio values. Because CMV seropositivity increases with age, higher CD8+ levels in older persons have been attributed to CMV seropositivity[109,110,112,123]. However, rates of CD8-CD4 disequilibrium (IHG-III or IHG-IV) and CMV seropositivity both increase with age (**Supplementary Fig. 20b-c**), and within each age stratum, CMV seropositivity rates were lower in those preserving IHG-I or IHG-II with higher ratio values (**Supplementary Fig. 20c**). The sexual dimorphism of CD8-CD4 disequilibrium status appears to be more prominent in CMV+ persons; however, a limitation of this inference was that females were under-represented in our cohorts.

Thus, we suggest that CMV seropositivity was unlikely to be a cause of CD8-CD4 disequilibrium grades IHG-III or IHG-IV. First, in each of the populations we evaluated, CD8-CD4 disequilibrium rates were proportionate to the grade of the proxy for antigenic stimulation including groups (FSWs, HIV+) in whom CMV seropositivity rates can approach 90%)[125]. That is higher levels of proxies for antigenic stimulation associated with higher rates of IHG-III or IHG-IV (e.g., age, BAS and STI scores, HIV viral load). Second, the reversibility of CD8-CD4 disequilibrium with mitigation of antigenic stimulation, including in CMV+ HIV-seronegative COVID-19 patients, HIV+ persons and FSWs made it unlikely that CD8-CD4 disequilibrium was a fixed trait attributable to chronic CMV infection/seropositivity. An alternative explanation was that akin to the model for the association between susceptibility to develop CD8-CD4 disequilibrium and increased HIV seroconversion risk (**Fig. 5d**), susceptibility to develop CD8-CD4 disequilibrium predisposes individuals to acquire CMV; post CMV seroconversion, CMV+ persons may restore IHG-I or IHG-II after dissipation of antigenic stimulation, akin to what we observed in HIV-seronegative FSWs and patients in the COVID-19 cohort.

HIV and CMV infection share risk factors[125]. These epidemiologic observations raised the possibility that akin to HIV serostatus, CMV serostatus has indicator functions of immunologic health linked to susceptibility/resistance to develop CD8-CD4 disequilibrium, wherein, CMV seropositivity is an indicator for susceptibility to develop CD8-CD4 disequilibrium whereas CMV seronegativity is an indicator of resistance to develop CD8-CD4 disequilibrium. With indicator function, some of the reported associations of CMV seropositivity may relate to a subset of CMV+ persons with heightened susceptibility to develop CD8-CD4 disequilibrium vs. CMV infection *per se*. Hence, akin to the HIV model (**Fig. 5d**), a tripartite model may be applied to CMV infection: (i) at time of exposure to CMV, chances of CMV seroconversion are greater in the presence of CD8-CD4 disequilibrium vs. equilibrium, predicting that CMV seropositivity is disproportionately overrepresented and underrepresented in persons with CD8-CD4 disequilibrium vs. equilibrium with higher ratios (IHG-I or IHG-II with ratio levels, respectively), (ii) post CMV seroconversion, antigenic stimulation associated with CMV viremia (akin to HIV viremia; **Fig. 3h**) further skews CD8-CD4 equilibrium toward CD8-CD4 disequilibrium, and (iii) skewing can be transient and reversed with mitigation

of antigenic stimulation attributable to CMV viremia [spontaneously/self-limiting or with therapy, akin to HIV viremia (**Fig. 3h**)] or other causes (e.g., during COVID-19; **Fig. 3b-f**). Hence, CD8-CD4 disequilibrium in a CMV+ person may point to a host with a heightened susceptibility to erode IR status in response to CMV viremia or other sources of antigenic stimulation vs. an attribute of CMV seropositivity *per se*. Contrarily, CD8-CD4 equilibrium in CMV+ persons may represent those who, subsequent to mitigation of antigenic stimulation, have re-attained CD8-CD4 equilibrium status or individuals acquiring CMV with an CD8-CD4 equilibrium status.

Hence, dependent on when cross-sectional sampling is performed relative to level of antigenic stimulation, comparisons of persons according to CMV serostatus may result in misattributing features of the host (susceptibility to develop CD8-CD4 disequilibrium) to the virus. Taken together, these findings suggest that development of CD8-CD4 disequilibrium grades IHG-III or IHG-IV and associated higher CD8+ levels in CMV+ persons is an indicator of a subset of persons with a heightened susceptibility to develop CD8-CD4 disequilibrium in response to ongoing antigenic stimulation vs. an invariant attribute of CMV seropositivity. Conversely, persons with a diathesis to restrict CD8+ expansion and preserve CD8-CD4 equilibrium states of IHG-I or IHG-II restrict CMV seroconversion. These inferences support the possibility that the reported associations of CMV seropositivity with higher CD8+ levels and diseases/mortality may relate to the subset of CMV+ with heightened susceptibility to develop CD8-CD4 disequilibrium.

Supplementary Note 4. IHG status in Ebola virus outcomes in Collaborative Cross-RIX mice

To extend the concept of evolutionary conservation of IR status to mice, we focused on the Collaborative Cross-RIX mice, a large panel of recombinant, inbred intercrosses (RIX) designed for complex trait analysis[107]. Based on median values of splenic CD4+ and CD8+ counts, we derived IHGs for 334 mice. At baseline, 48.5% (*n*=162), 38.3% (*n*=128), 1.5% (*n*=5), and 11.7% (*n*=39) were IHG-I, IHG-II, IHG-III, and IHG-IV, respectively. We evaluated the subset of the Collaborative Cross-RIX (CC-RIX) mice infected with Ebola virus (*n*=99) [17]; post-infection, mice strains were classified as resistant, partly resistant, and lethal. Among the strains of mice with lethal infection, there was an overrepresentation of strains whose uninfected counterparts had IHG-III or IHG-IV (CD8-CD4 disequilibrium) (**Fig. 4f; Supplementary Fig. 21**). Conversely, the uninfected counterparts of mice displaying resistance to Ebola infection were enriched for IHG-I. Thus, lethal infection, a proxy for lower immunocompetence, was more common in mice with CD8-CD4 disequilibrium before experimental Ebola infection whereas resistance may relate to the proclivity to preserve IHG-I. Due to the observed strain differences in IHG status, it is plausible that CD8-CD4 disequilibrium status may in part be a genetically-mediated trait.

Supplementary Note 5. Host genetic features that may associate with susceptibility vs. resistance to develop CD8-CD4 disequilibrium

In light of our findings in **Supplementary Note 3**, we examined whether resistance to develop CD8-CD4 disequilibrium had a genetic component vs. was stochastic. Previous studies identified a single-nucleotide polymorphism (SNP; rs2524054) in the major histocompatibility locus that, in genome-wide association studies of HIV– individuals, correlated with higher CD4:CD8 ratios and lower CD8+ levels[30] (**Supplementary Fig. 22a**). This raised the possibility that HIV– persons with IHGs that are defined by expanded CD8+ levels uncompensated by an increase in CD4+ counts, i.e., IHG-III or IHG-IV, were less likely to harbor this SNP.

To determine whether this SNP was associated with CD8-CD4 disequilibrium status, we categorized individuals into three groups: (i) individuals with CD8-CD4 disequilibrium (IHG-III or IHG-IV); (ii) individuals with IHG-I or IHG-II with ratio levels between ≥1 to 1.75; and (iii) individuals with IHG-I or IHG-II with ratio levels ≥1.75. We included ratio 1.75 as a cut-off, as in our literature survey of 13,703 HIV− persons worldwide, the median CD4:CD8 ratio was 1.75 (IQR: 1.57-2.04; **Supplementary Table 1**). Higher ratio values (≥1.75) associates with relatively lower CD8+ T-cell counts in persons with higher CD4+ (IHG-I) or lower CD4+ (IHG-II) counts. Thus, this approach permitted evaluation of genotype distributions in individuals with IHG-I or IHG-II with progressively lower CD8+ levels in the context of higher (IHG-I) or lower (IHG-II) CD4+ counts vs. individuals with higher CD8+ levels in the context of individuals with higher (IHG-III) or lower (IHG-IV) CD4+ counts. This approach mitigates the confounding that CD8+ levels can be similar in persons with different IHGs: for example, the median CD8+ levels in persons with IHG-IV were similar to those in individuals with IHG-I with ratio levels between ≥1 to 1.75 (**Supplementary Fig. 22b**, left). The highest CD8+ levels were in IHG-III.

In the overall HIV– UCSD (University of California of San Diego) cohort and its European-American component, homozygosity and heterozygosity for the rs2524054-A allele were incrementally higher in persons with IHG-I and IHG-II with higher ratio values, whereas homozygosity for the SNP was absent in persons with CD8-CD4 disequilibrium (**Supplementary Fig. 22b**). However, CMV seropositivity rates were progressively lower in the same groups enriched for homozygosity and heterozygosity for the rs2524054-A allele. Thus, the SNP is underrepresented vs. overrepresented in the same group of individuals distinguished by the highest or lowest CMV seropositivity rates, respectively (**Supplementary Fig. 22c**). Since CMV seropositivity is an acquired condition, these data indicate that CD8-CD4 disequilibrium (defined by CD8+ expansion relative to CD4+ counts) enriched in CMV seropositive persons may precede and contribute to CMV seroconversion, i.e., CMV seropositivity is not the cause of CD8-CD4 disequilibrium.

We termed this SNP as the CD8-CD4 disequilibrium-restricting SNP. Homozygosity for the disequilibrium-restricting SNP was associated with a 39% reduced likelihood of having CMV seropositivity; however, in the small sample size in which both genotype and CMV serostatus were available (n=635), this association did not achieve statistical significance at $P<0.05$ (OR=0.61; 95% CI: 0.31-1.19; P=0.146). These associations of the CD8-CD4

disequilibrium-restricting SNP in HIV– persons suggests that CD8-CD4 disequilibrium vs. equilibrium status are genetically influenced traits and CD8-CD4 equilibrium is less likely to be due to the absence of CMV seropositivity. Instead, as noted above in **Supplementary Note 3**, CD8-CD4 disequilibrium may precede and contribute to CMV seroconversion.

Supplementary Note 6. Age-appropriate evaluations of CD4+ and CD8+ counts and the CD4:CD8 ratio: accounting for IHGs mitigates confounding

A. *CD4+ lymphopenia of age, and disproportionately greater age-associated decline in CD8+ T-cells than CD4+ T-cells revealed after accounting for IHG status*

CD4+ lymphopenia of age is an aspect of aging after one accounts for confounding factors. Confounding occurs because of a) small numbers and b) a comparison is made between younger vs. older persons without regard to the fact that there are 4 IHG states that may be present in both groups and the CD8-CD4 profile of the IHGs differs. Thus, a comparison of younger vs. older persons without regard to the IHGs is confounded because it **masks** the immense complexity in CD8-CD4 profile changes that occur with age.

- IHG-I is the most common grade in human populations, as well as in younger nonhuman primates (**Fig. 2f, 4d**). With age, there is a switch from IHG-I to a non-IHG-I grade (**Fig. 2f-g, 6b**). Thus, a comparison between younger and older persons does not account for the fact that the prevalence of two IHGs is showing opposing frequencies: the prevalence of IHG-I is decreasing whereas the prevalence of IHG-III is increasing. That is, by failing to preserve IHG-I, some persons are experiencing a CD4+ loss, whereas, by developing IHG-III, others are preserving higher CD4+ counts with age. Immune traits differ by equilibrium status (**Fig. 10a,b**). Thus, depending on the representation of the IHGs in a study population, the CD4+ count decline may not be discernable.
- Similarly, during aging, persons may switch from IHG-I to IHG-II or IHG-IV (**Fig. 2f**); a switch to IHG-II signifies CD4+ lymphopenia but with restrained expansion of CD8+ T-cell counts, preserving CD8-CD4 equilibrium; in contrast, a switch to IHG-IV signifies CD4+ lymphopenia but with relatively higher CD8+ counts.
- The less-confounded way to examine CD4+ lymphopenia seen with age is to focus on CD4+ counts among younger and older persons by IHG status.
- **Supplementary Fig. 23a** depicts the differences in CD4+ and CD8+ counts and ratios in younger and older persons by IHG status. The data depicted indicate the following:
    i. No substantial differences in the values were observed in younger or older persons with IHG-III.
    ii. CD4+ counts were lower by 9.9%, 9.9%, and 15.9% in older persons with IHG-I, IHG-II, and IHG-III, respectively, vs. younger persons with the same grades.
    iii. CD8+ counts were lower by 28.7%, 23.5%, and 16.4% in older persons with IHG-I, IHG-II, and IHG-IV, respectively, vs. younger persons with the same

grades. There were no significant differences in CD8+ levels in younger or older persons with IHG-IV.

iv. Thus, there was a disproportionately greater decline in CD8+ T-cells than CD4+ T-cells in older persons with IHG-I or IHG-II. Because of this disproportionality, ratio values were higher by 22.2% and 21.7% in older persons with IHG-I and IHG-II, respectively, compared to younger persons with these same grades.

v. In contrast, ratio values were lower in older persons with IHG-IV compared with younger persons with the same grade.

B. *Why does the CD4:CD8 ratio value increase with age and then decline?*

The nuances noted above are obscured if these variables had been examined by age alone, which is plotted in **Supplementary Fig. 23b**. The data shown are a conflated assessment, as they show a progressive decline in CD4+ and CD8+ T-cell counts by age and an increase in the CD4:CD8 ratio followed by a decrease beginning at approximately age 70 years.

The question arises: why do ratio values increase initially with age and then decline in the older age population?

- This increase in ratio values with age is attributable to the changes in distributions of the IHGs that occur with age. The initial rise in ratio values is attributable to increases in ratio values in older persons with IHG-I and IHG-II (**Supplementary Fig. 23a,c**).
- However, with age there is a concomitant increase in IHG-III and IHG-IV tracking an inverted ratio (6.2% individuals in the 70- to 79-yr and 12.3% of those in the 80- to 103-yr age stratum have IHG-III or IHG-IV vs. 3.3% in individuals <40 yr) (**Fig. 2f**).
- Thus, the conflation of ratio values of IHG-I, IHG-II, IHG-III, and IHG-IV are lower in older persons attributable to the increased prevalence of IHG-III and IHG-IV in this group (**Supplementary Fig. 23c**).

C. *Age-dependent evaluation/interpretation of the CD4:CD8 ratio*

We have rigorously evaluated the data in an attempt to understand the basis of higher ratio values in older persons preserving IHG-I or IHG-II and their possible clinical implications. As described above, age is associated with an increase in ratio values in persons with CD8-CD4 equilibrium grades IHG-I or IHG-II. An increase in ratio values is not possible in persons with IHG-III or IHG-IV because, by definition, these IHGs have ratio values less than unity. To illustrate these points, we depict the kernel density plots of ratios by IHG status.

These kernel density estimate plots are shown in **Supplementary Fig. 23d-e**. They describe the prevalence (density) of ratio values in younger (<40 yrs) and older (≥40 yrs) individuals from the HIV- UCSD cohort (Panel a) and the SardiNIA cohort (Panel b). The median ratio values in 13,703 otherwise healthy individuals approximates 1.75 (**Supplementary Table 1**). These findings illustrate that:

i. Younger and older individuals may have ratio values <1.0, signifying CD8-CD4 disequilibrium grades IHG-III or IHG-IV.
ii. Younger compared with older persons with IHG-I or IHG-II are more likely to have ratio values that approximate the median ratio values in healthy persons (~1.75).
iii. Older compared with younger persons with IHG-I or IHG-II are more likely to have ratio values that exceed the point where the kernel density plots by age intersect, i.e., 2.5 in the UCSD cohort and 3.0 in the SardiNIA cohort.
iv. Thus, older persons (red line) preserving IHG-I or IHG-II are more likely to manifest higher ratio values. Higher ratio values were observed in a subset of older persons with CD8-CD4 equilibrium grades IHG-I or IHG-II.

Summary: Therefore, regardless of age, ratio values that approximate the median ratio values (~1.75) in persons with IHG-I or IHG-II are a sign of greater immunocompetence. Ratio values >2.5 in older persons with IHG-I or IHG-II are a sign of an aged CD8-CD4 equilibrium and may be associated with negative outcomes.

We wish to emphasize that we are agnostic to mechanisms. Thus, we are not ascribing the same mechanism behind the ratios, CD4+ or CD8+ counts, or IHGs. Our focus is twofold: (i) understanding the extent to which different diseases or conditions are associated with IHGs, regardless of mechanism, and (ii) defining whether the IHGs in different disease contexts have similar biomarker functions of predicting/associating with immunity-dependent outcomes. Being mechanism agnostic is important, as it is common in the scientific field to compare groups of individuals with differing host characteristics (e.g., age, HIV serostatus). However, such comparisons are prone to confounding as they do not account for the underlying heterogeneity in immune status. For example, a comparison of younger vs. older persons is a conflated assessment of populations with differing IHG distributions: IHG-I is more common in younger persons and non-IHG-I grades associated with inferior immunity-dependent outcomes are more common in older persons (**Fig. 6**). Mitigation of this confounding may be an important step, as we found that some immune trait levels are similar in younger and older persons with IHG-I or IHG-II (**Fig. 10a-c**). Thus, persons with the same IHGs but arising in the context of different diseases or conditions may associate with similar levels of immunosuppression regardless of age.

Supplementary Note 7. Survival rates in the Framingham Heart Study and the Vitality 90+ study

A. *Framingham Heart Study (FHS)*

In the FHS, age, sex, as well as expression levels of SAS-1 and MAS-1 were independent predictors of lifespan. Persons from the FHS with available gene expression data comprised 2,306 individuals (median age: 66 [IQR: 60 – 73] years; range 40-92 years; males: 45.7%), and associations with all-cause mortality over a 9-year follow-up period was determined. In a multivariate model, both age and sex independently associated with all-cause mortality: each 10-year increase in age associated with a higher mortality

hazard (HR, 2.98; 95% CI, 2.59-3.42) and males compared with females had a higher mortality hazard (HR, 1.61; 95% CI, 1.29-2.02). However, higher expression levels of SAS-1 were associated with a 41% lower mortality hazard over 9 years of prospective follow-up in the FHS (HR, 0.59; 95% CI, 0.45-0.78), after controlling for age and sex (**Fig. 2d**). In contrast, higher expression of MAS-1 associated with an 89% increased mortality hazards (HR, 1.89, 95% CI, 1.31-2.71), after controlling for age and sex (**Fig. 2d**).

B. *Vitality 90+ study* (**Supplementary Fig. 13e**)
The Vitality 90+ study examined peripheral blood transcriptomes from otherwise healthy younger individuals (*n*=30, median age [IQR] years = 22.5 [20.2-24.0]; females: 70%) and nonagenarians (*n*=146, ages: ≥90 years; females: 71%). Highlighting the primordial nature of IHG-I, most younger individuals had IHG-I (**Supplementary Fig. 13e**, left). In both younger individuals and nonagenarians, representation of the mortality-associated SAS-1^{low}-MAS-1^{high} profile was greater in males than females ((**Supplementary Fig. 13e**, left). The representation of the SAS-1/MAS-1 profiles in nonagenarians resembled that of older (81-92 years) FHS participants (compare **Supplementary Fig. 13a vs. 13e**). A total of 40% of nonagenarians died during a 4-year prospective follow-up; mortality hazards did not differ significantly by baseline SAS-1 status. However, even among a study group with extremely high mortality rates, lower baseline MAS-1 levels were associated with a trend for lower mortality rates (**Supplementary Fig. 13e**, right). That is, MAS-1^{low} in the context of SAS-1^{high}-MAS-1^{low} or SAS-1^{low}-MAS-1^{low} provided a further survival advantage to nonagenarians.

Supplementary Note 8. Immunologic traits that associated with IR status vs. age vs. both

A hallmark of aging was that, even among persons older than 80 years, there was strong preference to preserve CD8-CD4 equilibrium vs. disequilibrium (i.e., IHG-I vs. IHG-III or IHG-II vs. IHG-IV). This raised the possibility that, after controlling for age, immunologic traits are associated with CD8-CD4 equilibrium vs. disequilibrium. IHG-I and IHG-II are the most and second-most prevalent grades, respectively, in humans (**Fig. 2f**). That is, a diathesis to develop CD8-CD4 disequilibrium grades IHG-III and IHG-IV, a feature of the IR erosion-susceptible phenotype, is associated with a distinct set of immunologic traits at any age vs. those associated with aging in persons with the CD8-CD4 equilibrium grades IHG-I or IHG-II. We determined the levels of 75 immunologic traits according to (i) age, i.e., <40 vs. ≥70 years in persons with IHG-I or IHG-II, adjusting for sex, and (ii) IHG status, i.e., IHG-III vs. IHG-I and IHG-IV vs. IHG-II after controlling for age and sex (**Supplementary Fig. 24a**). Trait levels that varied to a greater extent by age in persons with IHG-I and IHG-II but did not differ substantially by IHG status for both comparisons that control for CD4+ count (IHG-III vs. IHG-I and IHG-IV vs. IHG-II) were categorized as age-associated traits. Traits that varied to a greater extent by IHG status but did not differ by age in persons with IHG-I or IHG-II were categorized as traits attributable to nonoptimal IR linked to CD8-CD4 disequilibrium grades (IHG-III or IHG-IV) (**Supplementary Fig. 24a; Supplementary Data 12**). Thus, in these analyses, we mitigated the confounding

by CD4+ count, sex, and age by comparing IHGs with similar levels of CD4+ counts (i.e., IHG-III vs. IHG-I and IHG-IV vs. IHG-II) and adjusting for sex and age.

Depending on whether the immunologic traits differed by age in persons with IHG-I or IHG-II and/or by IHG status, four immunologic trait groups emerged (**Supplementary Fig. 24a**; **Supplementary Data 12**). Within each group, depending on the directionality of the trait (higher or lower proportions of the trait by age and/or IHG status), the traits were subgrouped into signatures. Group 1 consisted of 13 traits and were subgrouped into signatures 1-4 and these associated with nonoptimal IR linked to CD8-CD4 disequilibrium grades IHG-III or IHG-IV (IR-associated traits after controlling for age and sex); Group 2 consisted of 22 age-associated traits (signatures 5-8); Group 3 consisted of 10 traits (signatures 9-12) that associated with both CD8-CD4 disequilibrium (IHG-III or IHG-IV) and age, albeit in some instances, the directionality of the trait levels showed opposite patterns in IHG-III or IHG-IV vs. age; and Group 4 consisted of 30 neutral traits (signatures 13-19). Representative signatures for IHG-III or IHG-IV, age, IHG-III or IHG-IV and age, and the neutral category are depicted [signatures 1, 6, 9, 10, and 13, respectively; **Supplementary Fig. 24b**).

Group 1, IHG-III or IHG-IV)-specific traits (signatures 1-4; **Supplementary Fig. 24a**; **Supplementary Data 12**) tracking higher levels in IHG-III and IHG-IV were natural killer (NK) T-cells, CD8+NKT-like cells, $CD127^{-}CD8^{bright}$ T-cells (effector-memory), $CD25^{++}CD8^{bright}$ (activated/proliferating) T-cells, and $CD28^{-}CD8^{dim}$ (senescent/terminally differentiated) T-cells [e.g., signature 1 ($CD127^{-}CD8^{bright}$ T-cells depicted in **Supplementary Fig. 24a**)]. The only group 1 trait lower in IHG-III and IHG-IV was naïve-transitioning T-cells ($CD4^{+}CD45RA^{+}CD25^{hi}$, not Tregs) (signature 2).

Group 2, age-associated traits (e.g., signatures 5-8 and lower levels of signature 10) included increased proportions of memory subsets within the CD4+ T-cell compartment (except for the central memory subset) and decreased proportions of naïve $CD8^{+}$ T-cells [e.g., signature 6 (naïve $CD8^{bright}$ depicted in **Supplementary Fig. 24b**], B cells ($CD19^{+}$), and plasmacytoid dendritic cells ($CD123^{+}CD11c^{-}$). While some traits showed similar directionality with IHG-III or IHG-IV and age (e.g., signature 12; **Supplementary Fig. 24a**), others had opposite patterns.

$CD28^{-}CD8^{bright}$ T-cells are viewed as a hallmark of aging[126]; while levels of these cells were higher with both age and IHG-III or IHG-IV, they were disproportionately higher in persons with IHG-III or IHG-IV(signature 9 depicted in **Supplementary Fig. 24b**). Naïve CD4+ T-cells were lower with both age and IHG-III or IHG-IV(**Supplementary Data 12**). However, levels of $CD28^{-}CD25^{++}CD127^{-}CD8^{bright}$ T-cells, likely representing regulatory CD8+ T-cells[127], were higher with IHG-III and IHG-IV but lower with age (signature 10 depicted in **Supplementary Fig. 24b**). A similar discordant pattern was observed with $CD28^{-}CD25^{++}CD8^{bright}$ T-cells (**Supplementary Data 12**). Neutral traits included mature dendritic cells (CD86+), monocytes, and some Treg subsets (e.g., signature 13 depicted in **Supplementary Fig. 24b**; **Supplementary Data 12**).

These findings suggest that the immunologic traits associated with nonoptimal IR linked to IHG-III or IHG-IV (IR associated traits) vs. age in persons with IHG-I or IHG-II are distinct (e.g., $CD127^{-}CD8^{bright}$ T-cells) whereas others are overlapping (e.g., $CD28^{-}CD8^{bright}$). Underscoring this viewpoint, across age levels of $CD127^{-}CD8^{bright}$ T-cells declined modestly but differed extensively by IHG status (**Fig. 10c**). In contrast, levels of $CD28^{-}CD8^{bright}$ T-cells increased significantly with age, but also differed significantly by IHG status (**Fig. 10c**).

To confirm these findings, we examined immunologic traits that differed by IHG-III or IHG-IV vs. age in nonhuman primates. Results presented from SIV+ sooty mangabeys (**Fig. 9d**) and rhesus macaques (**Fig. 9e**), suggest that immunologic features of CD8-CD4 disequilibrium grades IHG-III and IHG-IV were relatively conserved in humans and nonhuman primates and underscore that some immunologic traits previously attributed to age are likely to be attributable to CD8-CD4 disequilibrium after controlling for age. The CD8-CD4 disequilibrium grades (IHG-III or IHG-IV)-associated immunologic traits are commonly elevated in conditions associated with increased antigenic simulation and lower immunocompetence, regardless of age [e.g., chronic viral infections, transplant rejection, and some cancers[126,128-133]]. However, because CD8-CD4 disequilibrium (IHG-III or IHG-IV) rates also increase with age, immunologic traits and transcriptomic features associated with IHG-III or IHG-IV may be misattributed to those of chronological age.

Supplementary Note 9. Supplementary discussion - immunologic resilience program

A. Overview: We propose the following algorithm with respect to the association of optimal immunologic resilience (IR) with longevity and other superior immunity-dependent health outcomes.

1. Immunologic resilience (IR) signifies the capacity to preserve and/or restore immunocompetence (IC) coupled with control of inflammation (IF) during antigenic stimulation.
2. IR metrics indicate an IR continuum: optimal, suboptimal, and nonoptimal.
3. Optimal IR tracks an IC^{high}-IF^{low} state.
4. Some persons have a proclivity to preserve optimal IR even during high-grade antigenic stimulation. This proclivity signifies the IR erosion-resistant phenotype.
5. Conversely, some persons have a predilection to erode IR even in settings of low-grade antigenic stimulation. This predilection signifies the IR erosion-susceptible phenotype.
6. The IR erosion-resistant phenotype is associated with superior immunity-dependent health outcomes, including survival/longevity.
7. The optimal IR associated with superior outcomes after controlling for age and sex.
8. The IR erosion-resistant phenotype is more common in females than males.
9. Females have a longevity advantage independent of IR status.
10. Age, sex, and IR status are independent determinants of lifespan and possibly other immunity-dependent togethers.

Taken together, we suggest that IR tracks a fundamental, antigen-activated program that is intrinsically linked to IC and IF status. The IR erosion-resistant and erosion-susceptible phenotypes provide a parsimonious basis to explain the wide inter-individual variations in immune status among individuals of similar ages.

B. IR erosion-resistant vs. erosion-susceptible phenotypes.

We demonstrate that, depending on extrinsic (e.g., microbial burden) and intrinsic (genetics, sex) factors, immunologic health attributable to the IR program resides along a continuum quantifiable using CD8-CD4 equilibrium metrics. To determine the consequences of the IR erosion-resistant vs. susceptible phenotypes, we performed a large-scale disease association analysis, and additionally focused on experimental model systems of SARS-CoV-2, HIV, and viral infections, as well as a cancer (CSCC) that has high incidence and recurrence rates in solid organ transplant recipients. The sum of our results suggest that identification of the IR erosion phenotypes provides a cogent framework for (i) mapping individual- and population-level trajectories of immunologic health as well as disentangling potential cause-effect relationships and understanding sex-specific differences in disease risks and lifespan in a non-confounded manner, and (ii) reframing our understanding of the determinants that have shaped the SARS-CoV-2 and HIV pandemics. Our data suggest that a key factor for SARS-CoV-2 and HIV acquisition and/or disease progression is a person's susceptibility to erode IR in response to antigenic stimulation that antedated exposure to these viruses. That is, the likelihood of severe COVID-19 and HIV acquisition and AIDS progression rates are greater in persons with lower immunocompetence, and higher inflammation linked to the IR erosion-susceptible phenotype. Thus, a person's resistance to severe COVID-19 and HIV seroconversion and AIDS are indicators of persons with the IR erosion-resistant phenotype. We demonstrate that preservation of IHG-I, an indicator of high-grade CD8-CD4 equilibrium, is a marker of the IR erosion-resistant phenotype, whereas non-IHG-I grades are a marker of the IR erosion-susceptible phenotype.

Thus, we suggest that people vary in their proclivity to erode IR, regardless of the grade of antigenic stimulation. Our findings suggest that susceptibility to erode IR is a proximate risk factor for some age-associated inflammatory diseases (including features attributed to inflammaging), shorter lifespan, immunosuppressive conditions (some cancers), and susceptibility to and outcomes of chronic viral infections, such as HIV and CMV as well as acute infections such as SARS-CoV-2 and influenza.

B. IR erosion attributable to antigenic experience vs. age as an imperfect proxy for antigenic experience. Our findings indicate that a lower immunocompetence-high inflammation state attributable to the IR erosion-susceptible phenotype is not dependent on chronological age in itself. We believe that the distinction resides in the fact that erosion of IR is the byproduct of aging vs. due to aging, i.e., the longer one lives the greater the exposure to antigens. In turn, greater exposure increases the chances that an individual will erode IR.

This viewpoint is supported by several lines of evidence. We found that the IR erosion-susceptible phenotype is the expression of an evolutionarily conserved IR program activated in response to varied antigenic stimuli correlating with a switch from IHG-I to non-IHG-I grades. This switch reflects the effects of increased accumulation of host antigenic burden and is potentially reversible with a cause-specific decrease or cessation of antigenic stimulation. While erosion of IR may occur at any age conditional on host and environmental factors, an increase in antigenic exposures is an inevitable aspect of aging; hence, a switch from an IHG-I to non-IHG-I grade occurs with age. Thus, in persons experiencing moderate- or high-grade antigenic stimulation, erosion of IR can occur at any age; however, an eroded IR status at older ages (without a condition associated with moderate/high grade antigenic stimulation) is attributable to cumulative host antigenic burden of low-grade, repetitive antigenic challenges accrued over a lifetime. Consequently, features of the lower immunologic health and immune traits linked to an eroded IR in older persons may be misattributed to processes related to age and assigned monikers such as inflammaging, immune risk phenotype, and immunosenescence of age.

C. How might antigenic stimulation erode IR?

The sum of our data, including the viral challenge/infection studies (**Fig. 8e-h**) suggest the following algorithm by which antigenic stimulation erodes immune status. We show that:

1. Metrics indicative of optimal IR track an IC^{high}-IF^{low} state.
2. Settings associated with increased antigenic stimulation are associated with a switch from an optimal IR status to suboptimal or nonoptimal IR status.
3. This switch corresponds to a switch from an IC^{high}-IF^{low} to IC^{low}-IF^{high} state.
4. This switch was observed in the settings of low-grade (e.g., aging), moderate-grade (COVID-19, risk factor-associated antigenic stimulation, influenza), and severe-grade (sepsis, HIV) antigenic stimulation.
5. Mitigation of antigenic stimulation was associated with reconstitution of optimal IR status (i.e., switch from an IC^{low}-IF^{high} to IC^{high}-IF^{low} state).
6. However, despite mitigation of antigenic stimulation, some persons manifest persistent IC^{low}-IF^{high} state.
7. Metrics tracking an IC^{low}-IF^{high} state show an association of this state with mortality and other inferior immunity-dependent health outcomes.

D. Immunologic traits of IR vs. age.

We distinguished immunologic traits that track nonoptimal IR linked to CD-CD4 disequilibrium grades IHG-III or IHG-IV vs. age in persons preserving the two most common grades in humans. Immunologic traits of CD8-CD4 disequilibrium [IHG-III or IHG-IV] (e.g., expansion of NK T-cells and senescent and terminally differentiated CD8+ T-cells) appear to share conserved characteristics across humans and non-human primates, regardless of HIV or SIV serostatus. Some of these traits are hallmarks of conditions/diseases associated with increased antigenic stimulation and lower immunologic health (e.g., chronic viral infections, some cancers, older age). In contrast, age is likely an irreversible process and correlates of an aged CD8-CD4 equilibrium included a distinct set of immunologic traits, e.g., loss of naïve CD8+ T-cells, B cells and plasmacytoid DC.

E. Erosion of IR as an essential host defense strategy – a trade-off to mitigate autoimmunity?

An eroded IR (non-IHG-I grades) was higher in epidemiologic contexts with greater infectious disease burdens. Thus, evolutionary preservation of a trait associated with lower immunologic health suggests that immune processes leading to an eroded IR are part of an essential host defense strategy, potentially mitigating autoimmunity and reducing the effect of infections on host fitness in settings of higher infectious disease burden. The concept of disease tolerance as a defense strategy was first advanced by Medzhitov et al.[134].

Homozygosity for a polymorphism (CD8-CD4 disequilibrium-restricting SNP) in the major histocompatibility locus was underrepresented in those with CD8-CD4 disequilibrium, suggesting that susceptibility to develop disequilibrium grades IHG-III or IHG-IV may have a genetic basis (**Supplementary Note 5**). However, irrespective of this genetic basis, females resist erosion of IR throughout life. Women manifest a survival advantage and greater immunocompetence, reflected by resistance to infections, better vaccine responses and resistance to some age-associated diseases (e.g., some cancers)[104-106,135-142]. The female survival advantage is also seen in other species[104]. Our data suggest that females are more likely to manifest the IR erosion-resistant phenotype – even in the context of high-grade antigenic stimulation. Resistance to erode IR may underpin observations by others that women have lower HIV viral loads[143] and reservoir sizes[144]. Greater preservation of the IR erosion-resistant phenotype in women may reflect preservation of traits required for population survival allowing women to enter reproductive years with greater immunologic health, promoting reproduction and fetal health as well as lifespan. The tradeoff is that females are more susceptible to autoimmune diseases at a younger age[145,146]. Congruent with the idea that resistance to erode IR is a correlate of autoimmunity, as the CD8-CD4 disequilibrium-restricting SNP has been associated with autoimmune diseases[30]. Thus, we advance the idea that the IR erosion-resistant vs. erosion-susceptible phenotype may represent proximate risk factors for diseases that reside on the autoimmune vs. inflammatory disease spectrum as well as contribute to individual variation in lifespan.

F. Eroded IR prior to HIV seroconversion: implications

We extend the idea first advanced by Levy and Ziegler[147] and Sonnabend et al.[148] that immunosuppression may both precede and contribute to HIV infection. Our epidemiologic survey indicates a high rate of CD8-CD4 disequilibrium grades IHG-III or IHG-IV in HIV-seronegative persons with behavioral and non-behavioral (e.g., schistosomiasis) risk factors for HIV infection. The elevated rates of disequilibrium (IHG-III or IHG-IV) in persons with high-risk behaviors may relate to antigenic stimuli attributable to STIs and alloimmunization to sperm, semen, and/or blood antigens[147-153]. In FSWs, the contribution of the IR erosion-resistant phenotype to HIV acquisition was high. Consistent with our findings, CD8+ and CD4+ T-cell values reflective of incipient disequilibrium (reflected by CD4:CD8 ratio values close to 1.0) predicted HIV seroconversion in hemophiliacs after controlling for the quantity of non-recombinant clotting products received[154]. In a prophylaxis trial in men who have sex with men, those who subsequently seroconverted

had CD8+ and CD4+ T-cell values consistent with incipient disequilibrium at baseline[155]. In contrast, preservation of CD8-CD4 equilibrium grades IHG-I or IHG-II despite ongoing risk factor-associated increased antigenic stimulation may be a correlate of individuals designated previously as highly exposed, HIV-seronegative individuals[156].

Collectively, our findings suggest that a trifecta of factors that favor HIV acquisition: (i) a host with an IR erosion-susceptible phenotype with a proclivity for developing CD8-CD4 disequilibrium grades IHG-III or IHG-IV; (ii) causes of antigenic stimulation (behavioral or nonbehavioral related) of enough severity and/or chronicity to induce disequilibrium; and (iii) exposure to HIV in the presence of disequilibrium. Hence, HIV may be restricted to the subset of individuals with this proclivity experiencing moderate- to high-grade sources of antigenic stimulation. We therefore suggest that (i) susceptibility to develop CD8-CD4 disequilibrium in response to antigenic stimulation rather than the risk factors *per se* undergirds increased HIV acquisition risk, and (ii) in persons at high risk for exposure to HIV, seropositivity vs. seronegativity for HIV is an indicator of persons who, respectively, have a proclivity for developing vs. resisting development of CD8-CD4 disequilibrium. We suggest similar parallels for CMV seroconversion.

Evaluations of incident cohorts supported the notion that the contribution of the IR program is consistent along a continua: (i) seroconversion with HIV or CMV is more likely to occur in the presence of CD8-CD4 disequilibrium grades IHG-III or IHG-IV; (ii) following infection, viremia further precipitates immune skewing toward disequilibrium; and (iii) with cessation of virus-associated antigenic stimulation, skewness dissipates. In these continua, the indicator/proxy functions of HIV or CMV serostatus are masked in settings of higher antigenic stimulation and revealed after stimulation is suppressed. While pre-existing immunosuppression linked to CD8-CD4 disequilibrium may predispose individuals to HIV acquisition, disequilibrium induced by HIV-associated antigenic stimulation further compounds the immunosuppression post-infection. Thus, post-HIV infection, persons may have CD8-CD4 disequilibrium attributable to two causes of antigenic stimulation: risk factor-induced disequilibrium and HIV viremia-induced disequilibrium. However, only the latter cause of disequilibrium is responsive to ART.

Our findings have three practical applications for HIV prevention and treatment-associated immunologic reconstitution. First, although pre-exposure prophylaxis with antiretroviral medications may protect against HIV infection[157], in the absence of barrier protection, antigenic stimulation associated with high-risk behavioral activity may induce CD8-CD4 disequilibrium grades IHG-III or IHG-IV and its associated sequelae. Second, our results point to a risk continuum, as susceptibility to develop CD8-CD4 disequilibrium predisposes to not only HIV acquisition but also poorer outcomes both before and during ART. Hence, persistent IHG-III or IHG-IV in HIV+ persons, despite early and durable suppression of viremia, may reflect effects of pre-existing or ongoing risk factor-associated antigenic stimulation vs. effects of HIV infection per se. Third, curative strategies may be less effective in persons who fail to reconstitute IHG-I after ART, and vaccines to prevent HIV infection may be less effective in those with incipient or full-scale CD8-CD4 disequilibrium.

G. IR status and CMV serostatus

CMV seropositivity as an indicator persons with a proclivity to develop IHG-III or IHG-IV may explain its association with varied adverse outcomes[108-122,158,159]. That is, these associations may reflect those of a subset of CMV+ persons with a proclivity to develop disequilibrium grades IHG-III or IHG-IV in response to varied antigenic stimuli rather than effects of CMV *per se*. Consistent with the idea that CMV seronegativity is an indicator of the IR erosion-resistance phenotype, individuals with familial longevity in the Leiden Longevity Study have immunologic features consistent with this phenotype, including CMV seronegativity[160,161]. The distinction of CMV serostatus as an indicator of the IR erosion-susceptible has therapeutic implications. For example, In immunocompromised HIV– persons (e.g., bone marrow transplantation), CMV seropositivity is correlated with adverse outcomes[111,162]. However, treatment of CMV infection may be insufficient, as the proximate defect may reside in the host, i.e., an increased susceptibility to develop CD8-CD4 disequilibrium in response to either CMV viremia or other environmental triggers.

H. Summary:

Taken together, we describe an antigen-activated, sexually dimorphic, genetically influenced, immunosuppression-mediating program that shapes the trajectory of immunologic health and disease risks from early life. The IR erosion-resistant vs. erosion-susceptible phenotypes may contribute to contrasting disease risks (autoimmune vs. inflammatory diseases), and some of the associations of HIV and CMV seropositivity with adverse outcomes may stem from hosts with heightened susceptibility to erode IR (increased CD8-CD4 disequilibrium) in response to antigenic stimulation, related or unrelated to HIV or CMV viremia. Although further work is needed, our results (i) underscore the need to distinguish between reductions in immune status attributable to an eroded IR vs. the direct effects of age to avoid confounding interpretation of studies aiming to investigate disease mechanisms and lifespan; (ii) provide a roadmap for personalized medicine, proposing IR metrics as a facile method to monitor immunologic status, regardless of HIV serostatus or age; and (iii) advance the concept that selection of immunocompromised hosts (susceptibility to develop CD8-CD4 disequilibrium) for infection by HIV may reflect an evolutionary strategy to promote the life cycle of HIV and sustain the HIV epidemic.

Supplementary Note 10. Strengths and limitations

Strengths of this study include our evaluation of large multi-cohorts of longitudinal and cross-sectional data of HIV-seronegative and HIV+ individuals to describe the conceptual underpinnings of a novel IR program. We derived laboratory and transcriptomic metrics to gauge the degree to which this program is activated in response to antigenic stimulation, as well as reversal. This model was conceived a priori based on conundrums conveyed by the results of two prior studies from our group[9,10] (**Supplementary Note 1**). Moreover, as described in the Methods and **Supplementary Note 2**, we took several measures to limit bias and confounding and for replication purposes, we evaluated complementary cohorts. There were several limitations.

First, the extent and exact sources of individual cumulative antigenic stimulation was not possible to measure. We approximated these levels of stimulation using specific populations representative of relatively homogenous antigenic stimuli [e.g., egg counts in persons with schistosomiasis; accounting for duration of immunosuppression in RTRs, risk factors in FSWs, HIV viral load in HIV+ persons, SIV serostatus in Sooty mangabeys, and age (imperfect proxy for duration of antigenic experience)].

Second, our suggestion that IHG-I is the most common status in HIV– adults worldwide was derived from cohorts with distinct characteristics. The SardiNIA cohort is a large representative sample of the adult population geographically residing within four villages of eastern Sardinia. However, similar age-appropriate IHG distributions were observed in SardiNIA participants and during the convalescence phase after acute COVID-19. Transcriptomic metrics of IR status were evaluated in the Framingham Heart Study, Finnish cohort, San Antonio Heart Study, and other cohorts. However, irrespective of geography, analysis of other HIV– cohorts that included the UCSD HIV– cohort from USA and HIV– FSW cohorts in Nairobi, Kenya, suggested the generalizability that IHG-I is the most common status in adults. Additionally, the reconstitution of IHG-I after mitigation of antigenic stimulation in three separate cohorts (COVID-19, FSWs and HIV+) supported this thesis.

Third, in HIV+ cohorts, some measurements occurred at different intervals from infection or seroconversion. However, the large sample sizes of the EIC and PIC cohort helps mitigate these possible concerns, and the general pattern of associations were similar in both cohorts.

Fourth, in the FSW cohort, there is the possibility that some of the tests for HIV are false negatives so that the measurement being recorded as HIV– are HIV+. To control for this, when evaluating HIV– measurements, we initially focused only on those measurements that occurred at least 3 months prior to their last HIV– measurement, or for those that subsequently seroconverted, at least 3 months prior to their seroconversion date.

Fifth, comparing gene expression scores from cohort to cohort may be affected by availability of genes, the median expression value of the genes within a study, batch effect, and microarray chip used. In general, the same trends were observed across studies strengthening our viewpoint that the results are representative and accurate. Additionally, when directly comparing studies, we used only studies that used the same microarray chip, merged the studies together to create a uniform set of genes, and normalized the studies concomitantly, which reduces, but not completely mitigates, batch effect, and then calculated the median expression value across all of the combined studies.

Supplementary Note 11. Bradford Hill criteria – framework for causal inference

Hill's criteria for causation[163] are 10 criteria widely used in public health research to establish whether there is epidemiologic evidence of a causal relationship between a

presumed cause and an observed effect. We applied this framework to interpret the associations reported. The sum of our findings met 10 criteria to suggest that the association between IR metrics and level of immunocompetence and inflammation as well as proxies for superior immunocompetence is potentially causal, and are independent of (after controlling for) age, sex, and HIV or SIV serostatus:

1) Strength of association (effect size).

Criterion: The larger the association, the more likely that it is causal; however, small associations do not mean lack of a causal effect. Strength is assessed by both statistical significance ($P<0.05$, $P<0.01$, $P<0.001$) and magnitude of an association. Where the statistical significance is hindered by the number of samples, the strength is assessed by the magnitude/directionality of the association.

Criterion evidence in our study:

a. Clinical outcomes
- The effect sizes for the associations of IHG status with risk of HIV acquisition, AIDS progression rates, progression rates to second occurrence of CSCC in RTRs and COVID-19 outcomes were large.
- The directionality of these associations was consistent wherein, IHG-I consistently associated with a protective effect.

b. Mechanistic transcriptomic correlates of IR status:
- In our report, we focused on two transcriptomic signatures. The scores of the SAS-1 and MAS-1 signatures associated with mortality hazards in persons with or without COVID-19, and the score of these 2 signatures distinguished persons with vs. without IHG-I.
- The scores of these signatures in older HIV-seronegative persons is similar to that of younger persons with increased antigenic stimulation (HIV+ regardless of therapy status; latent or active tuberculosis).
- The effect size of the associations of the SAS-1 and MAS-1 signatures with mortality hazards in the COVID-19 cohort and the Framingham Heart Study were large.

c. Immunologic outcomes:
- The effect size of the association of the IHG status with immunologic traits was large.

2) Consistency (reproducibility)

Criterion: Different datasets comprising different populations, geographical locations, samples, and methods show a consistent association between two variables with respect to the null hypothesis. The more consistent the findings, the more likely that an association is meaningful.

Criterion evidence in our study:
- Across several cohorts, increasing levels of antigenic stimulation (proxied for example by age, HIV viral load, behavioral risk factors) associated with incrementally higher levels of a shift from an IHG-I grade to non-IHG-I grades

- Conversely, mitigation of antigenic stimulation resulted in restoration of IHG-I.
- Across several non-COVID-19 cohorts and both sexes, the pattern of change in levels of the SAS-1 and MAS-1 across age was consistent.
- While we did not have a replication COVID-19 cohort, similar association patterns were observed when examining older vs. younger persons, or CMV seronegative vs. seropositive persons.

3) Specificity

Criterion: Causation is likely if there is a specific population at a specific site and disease with no other likely explanation. The more specific an association between a factor and an effect is, the bigger the probability of a causal relationship.

Criterion evidence in our study

- In the populations studied (FSWs, HIV cohorts, SIV), a stepwise increase in the level of antigenic stimulation associated with incremental increase in the proportion of individuals developing non-IHG-I grades. Reflecting specificity, restoration of IHG-I with mitigation of antigenic stimulation (in FSWs, HIV+ persons and COVID-19 patients) reinforces the specificity of the association between antigenic stimulation and erosion of IR.

4) Temporality:

Criterion: The effect has to occur after the cause (and if there is an expected delay between the cause and expected effect, then the effect must occur after that delay).

Criterion evidence in our study

- Presence of IHG-I at baseline associated with resistance to incident HIV seroconversion, AIDS, recurrent cutaneous squamous cell cancer, and severe COVID-19 (hospitalization and mortality).
- Representation of the combinations of the SAS-1 and MAS-1 signatures at baseline associated with mortality hazards.
- Murine studies suggest that IHG-I also associates with immunity-dependent outcomes (resistant Ebola virus infection).

5) Biological gradient:

Criterion: Greater exposure should generally lead to greater incidence of the effect. However, in some cases, the mere presence of the factor can trigger the effect. In other cases, an inverse proportion is observed: greater exposure leads to lower incidence.

Criterion evidence in our study

- Age serves as an imperfect proxy for accumulated antigenic experience, and accordingly in both humans and non-human primates, age associated with a stepwise increase in the shifts from IHG-I to non-IHG-I grades.
- Higher levels of proxies for antigenic stimulation (behavioral activity score, sexually transmitted infection scores, HIV viral load) associated with increased rates of non-IHG-I grades.

- In general, as well as dependent on the study population and cause of antigenic stimulation, IHG-I associated with the best clinical outcomes whereas IHG-II, IHG-III and IHG-IV associated with worse outcomes.
- SAS-1 and MAS-1 gene signatures had additive effects as SAS-1^{high}-MAS-1^{low} vs. SAS-1^{low}-MAS-1^{high} associated with the extremes of mortality hazards, whereas combinations of SAS-1 and MAS-1 gene signature scores associated with intermediate mortality hazards.

6) Reversibility:
Criterion: If the cause is deleted then the effect should disappear as well.

Criterion evidence in our study
- Reconstitution of IHG-I status was observed with mitigation of antigenic stimulation in FSWs and HIV+ persons, as well as in patients with acute COVID-19.

7) Plausibility:
Criterion: A plausible mechanism between cause and effect is helpful (this is limited by current knowledge).

Criterion evidence in our study
- The concept that increased antigenic stimulation may induce a pro-inflammatory, immunosuppressive state is supported by studies in HIV+ individuals. Additionally, low-grade ongoing antigenic stimulation in younger HIV+ persons on antiretroviral therapy is thought to contribute to the earlier emergence of diseases that are typically observed in older HIV-seronegative persons (e.g., cardiovascular disease, cancer).

8) Coherence:
Criterion: Coherence between epidemiological and laboratory findings increases the likelihood of an effect. Lack of evidence cannot nullify the epidemiologic effect on associations.

Criterion evidence in our study
- Consistent with the association of IHG-I with better clinical outcomes, IHG-I also associated with immunologic and transcriptomic signatures tracking higher immunocompetence and lower inflammation.

9) Experiment:
Criterion: Evidence is drawn from experimental manipulation. In epidemiologic studies of disease, association declines following an intervention or cessation of exposure.

Criterion evidence in our study
- We used three experimental systems in which proxies for antigenic stimulation could be quantified: risk factor-associated antigenic stimulation, HIV viral load, and nonhuman primates with vs. without SIV infection. In each of these systems, a

consistent theme emerged: higher levels of antigenic stimulation association with increased CD8-CD4 disequilibrium (increase in IHG-III/IV).

- Concordance was observed in immunologic traits that associated with CD8-CD4 disequilibrium in humans and nonhuman primates.
- We evaluated experimental viral infection challenge studies and observed congruent results. Influenza infection challenge studies in mice were supportive.
- Both transcriptomics and immunologic studies indicated that CD8-CD4 disequilibrium grades IHG-III or IHG-IV associates with markers of immunosenescence that had previously been misattributed to aging.

10) Analogy:

Criterion: The use of analogies or similarities between the observed association and any other associations means that when one causal agent is known, the standards of evidence are lowered for a second causal agent that is similar in some way. Lack of analogy does not preclude causation.

Criterion evidence in our study

- We observed similar associations of non-IHG-I grades with (i) immunity-dependent health outcomes in humans and nonhumans, and (ii) immunologic traits in humans and nonhumans.
- We observed similar associations of SAS-1/MAS-1 profile responses following varied infection challenges.

Of the ten Bradford Hill criteria mentioned above, the key criterion is temporality, i.e., cause (higher levels of CD8-CD4 disequilibrium [IHG-III or IHG-IV]) must precede effect (inferior immunity-dependent outcomes, and immunologic/transcriptomic traits of immunosenescence). This criterion is supported in our analyses of longitudinal cohorts. Thus, taken together, within the framework of Bradford-Hill criteria, our results suggest a potentially causal association for following pathway: antigenic exposures—increased risk for developing CD8-CD4 disequilibrium—lower immunocompetence—adverse outcomes, including mortality. However, as illustrated by our findings, individuals vary in their susceptibility to erode IR.

E. Supplementary Figures

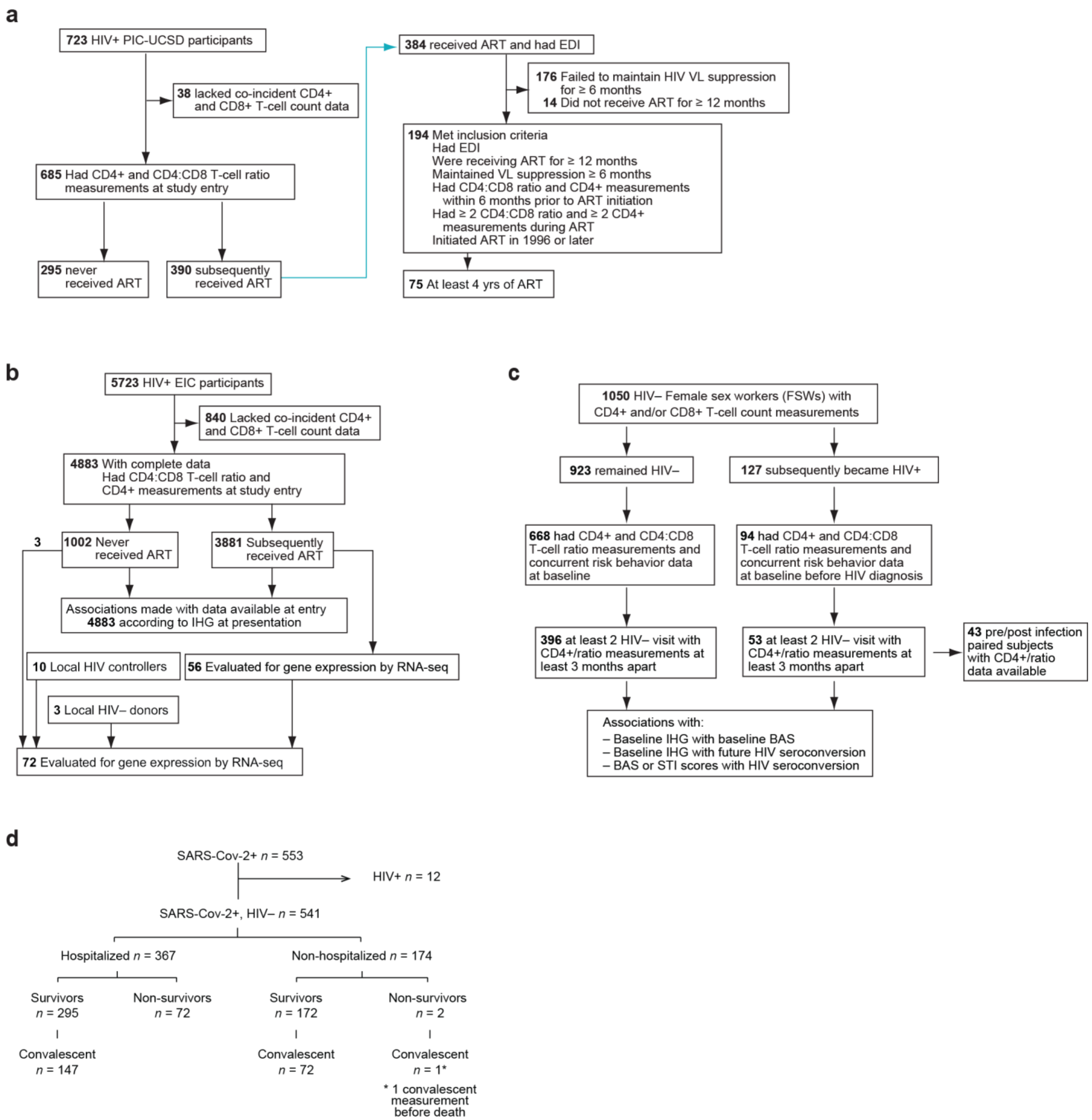

Supplementary Fig. 1. Study cohorts. Inclusion criteria applied to cull the study participants in the indicated cohorts. (**a**) Primary HIV+ infection cohort (PIC) from the University of California San Diego. (**b**) Early HIV+ infection cohort (EIC) reflecting participants of the U.S. Military HIV Natural History Study. (**c**) Female sex worker (FSW) cohort from the Majengo Observational Cohort Study (Kenya). (**d**) COVID-19 cohort. ART, antiretroviral therapy; BAS, behavioral activity score; EDI, estimated date of infection; STI, sexually transmitted infection; VL, plasma HIV viral load.

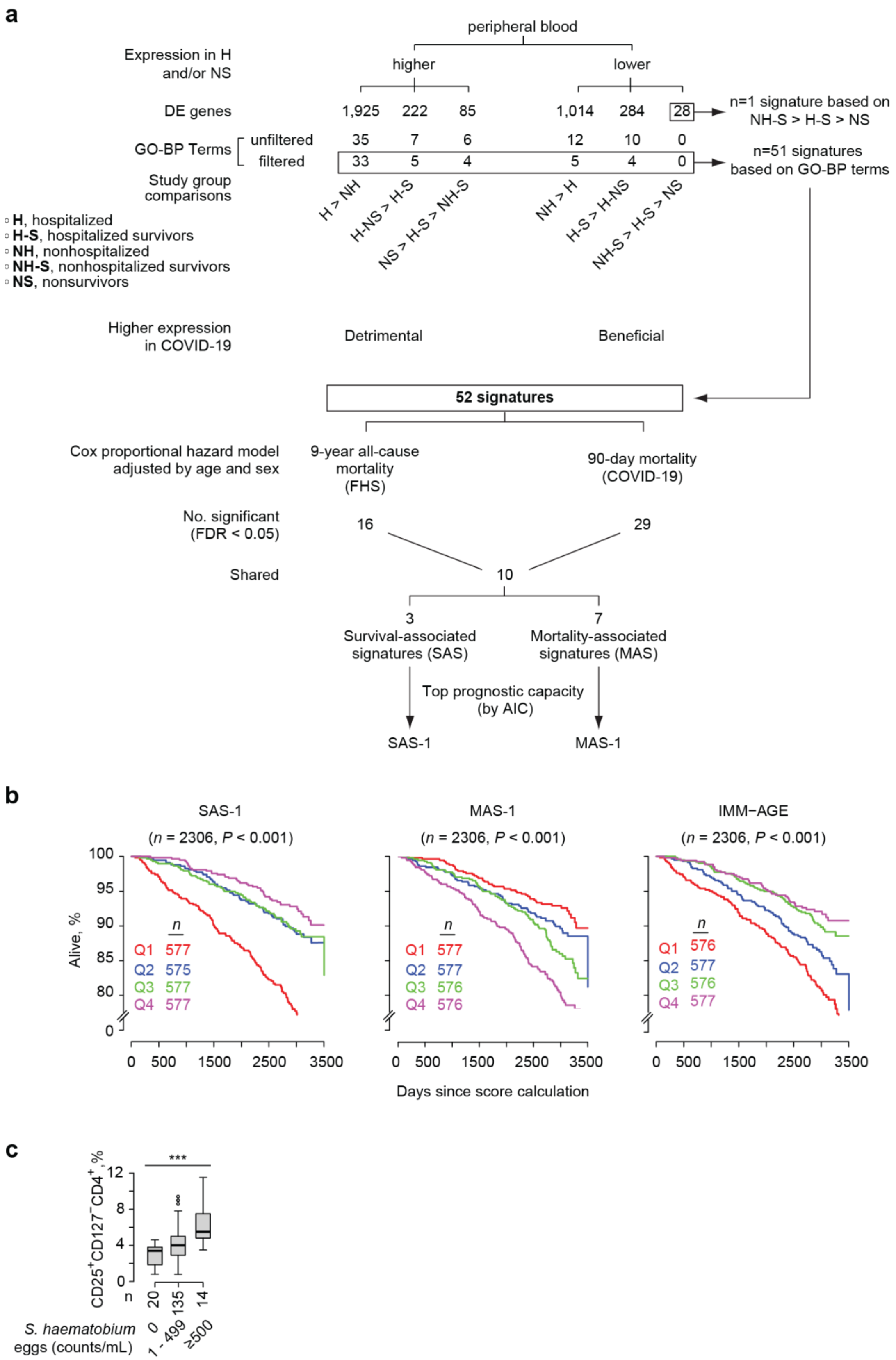

Supplementary Fig. 2. Derivation of the SAS-1 and MAS-1 signature and their association with survival in Framingham Heart Study (FHS); and association of immune trait with *Schistosoma haematobium* egg counts. (**a**) Methods used to derive beneficial and detrimental traits in

transcriptomes from peripheral blood derived from persons in the COVID-19 cohort. Top to bottom: Gene or trait expression levels either higher (GoDF, gain of detrimental function) or lower (LoBF, loss of beneficial function) in hospitalized (H) patients and/or nonsurvivors (NS). Number of significantly (FDR<0.05) differentially expressed (DE) genes for each pattern are shown. Significant (FDR<0.05) gene ontology terms for biological process (GO-BP) terms derived based on DE genes and subjected to a filtering process to reduce redundant terms as detailed in Supplementary Information Section 8.2. Study group comparisons. Association of higher expression levels of detrimental and beneficial traits in COVID-19 patients associated with detrimental or beneficial outcomes, respectively). See Supplementary Information section 8.2 for a detailed description of the analysis. Additional filtering was applied using age-adjusted Cox proportional hazards models of mortality in the COVID-19 cohort and FHS offspring cohort (**Fig. 2d**). (**b**) Kaplan-Meier (KM) plots of proportion survived (9-year) in the FHS offspring cohort stratified by quartiles (first through fourth, Q1-Q4) of indicated gene signature scores. (**c**) Percent CD25+CD127-CD4+ T-cells in children with schistosomiasis stratified by urine egg counts. ****P*<0.001. For boxplots, center line represents the median, box represents the interquartile range (IQR), whiskers represent the rest of the data distribution and outliers greater than ±1.5 × IQR are represented as points. Two-sided tests were used. Statistics are outlined in Supplementary Information Section 11.4.2., *P* values are in Supplementary Data 14, and Source data are provided as Source data file.

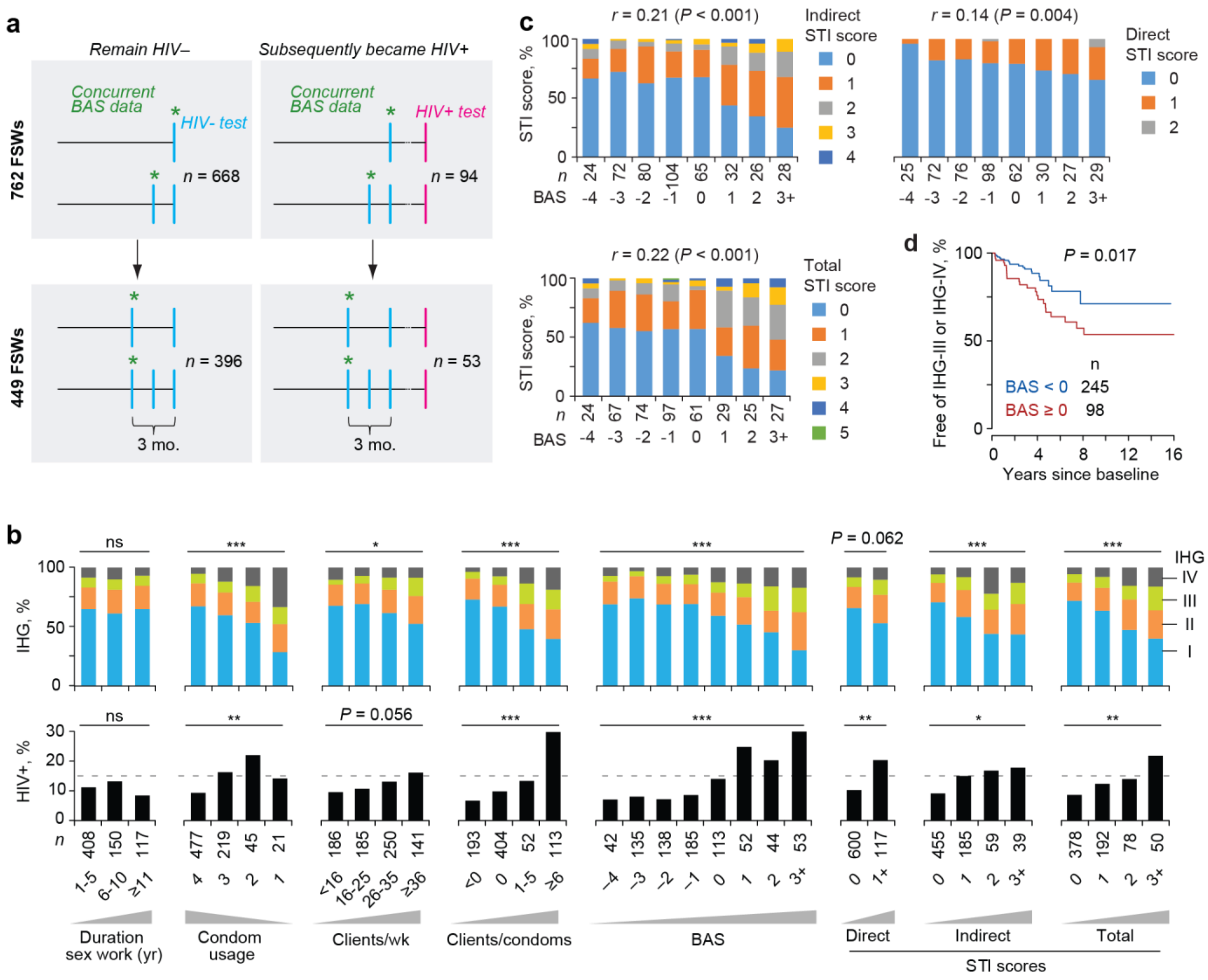

Supplementary Fig. 3. Associations of behavior and biologic risk factors with outcomes in HIV– female sex workers (FSWs). (**a**) Selection of FSWs based on HIV antibody tests. The 449 FSWs were selected because they had at least 2 HIV seronegative tests that were at least 3 months apart, regardless of whether they seroconverted (see **Supplementary Fig. 1c** as well)**. *** (specific for this panel)**,** timepoint had concurrent data to compute behavioral activity score (BAS). (**b**) Distribution of immune health grade (IHG) at baseline (top) and subsequent HIV seroconversion rates (bottom) in the overall cohort of 762 FSWs who were HIV− at baseline stratified according to the indicated behavioral and biological risk factors graded at baseline. Behavioral risk factors were duration of sex work, condom use (1, never; 2, <50%; 3, ≥50%; and 4, always), clients/week, and Δ (clients – condoms) (the difference between the number of clients/wk and condoms used/wk). (**c**) Proportions of indirect, direct, and total sexually transmitted infection (STI) scores by BAS score. (**d**) Time to IHG-I or IHG-II by baseline BAS in FSWs that were IHG-III or IHG-IV at baseline and remained HIV−. ns, nonsignificant; *, *P*<0.05; **, *P*<0.01; ***; *P*<0.001. Two-sided tests were used. Statistics are outlined in Supplementary Information Section 11.4.3., *P* values are in Supplementary Data 14, and Source data are provided as Source data file.

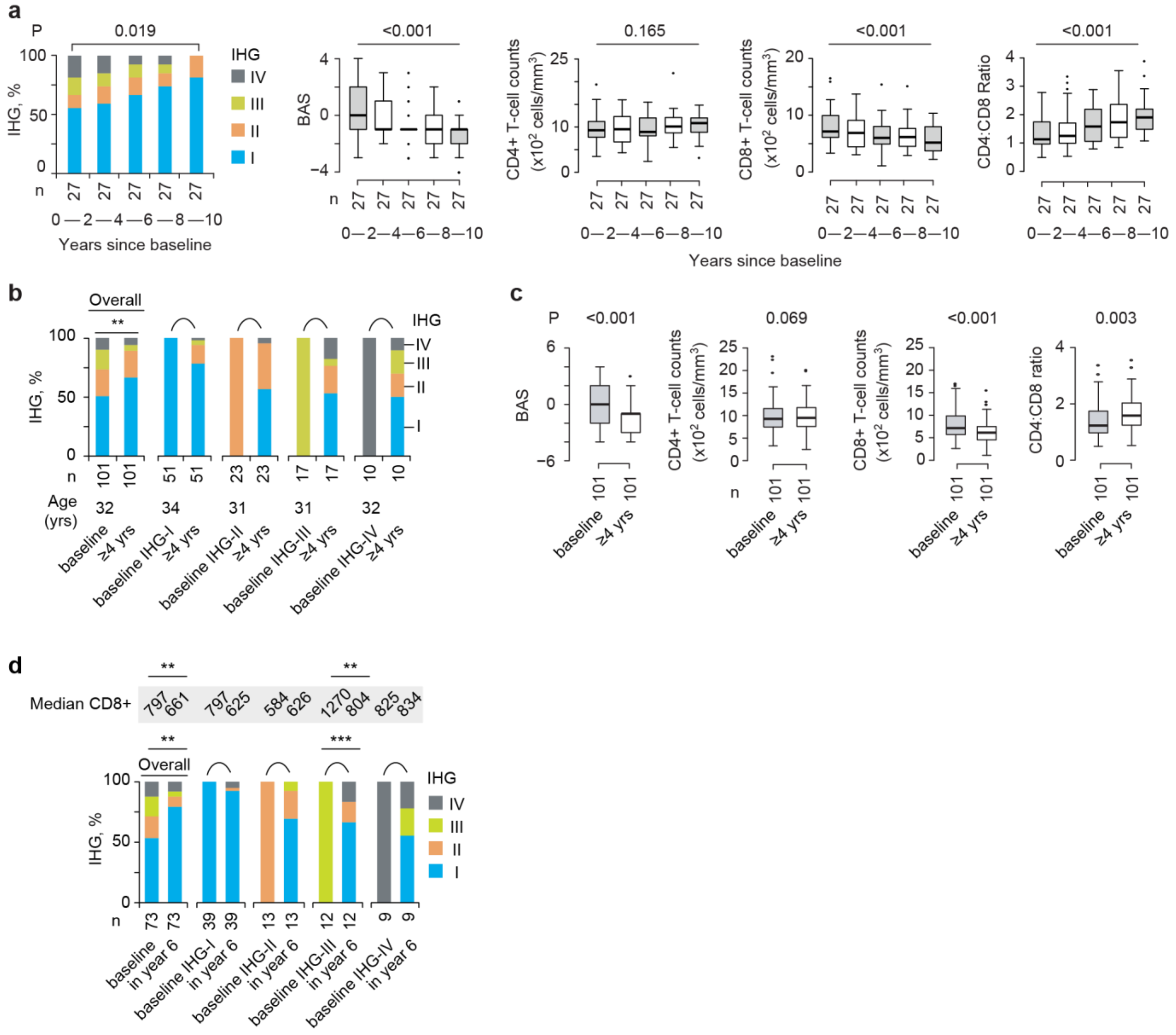

Supplementary Fig. 4. Induction and reversibility of CD8-CD4 disequilibrium in female sex workers (FSWs). (**a**) Characteristics of 27 HIV– FSWs with available CD8-CD4 equilibrium and behavioral activity score (BAS) data within every 2-year window for 10 years. Immune health grade (IHG) distribution within time intervals from cohort entry. The first available CD4+ and CD8+ T-cell counts, CD4:CD8 ratio, and accompanying BAS data during each 2-year interval are shown. (**b**) Distribution of IHGs at baseline (overall and by IHG) and at first available IHG after 4 years in 101 FSWs who remained HIV− for at least 4 years. Half-moon arrows depict change in IHG distribution from baseline. (**c**) Baseline median (interquartile range [IQR]) measurements of the first concurrently available CD4+ T-cell count, CD8+ T-cell count, CD4:CD8 T-cell ratio, and BAS in 101 FSWs who remained HIV-seronegative for at least 4 years. Differences between the values recorded at baseline and after 4 years of follow-up are noted. (**d**) Median CD8+ T-cell counts shown in top for groups indicated in bottom panel. Distribution of IHGs at baseline (overall and by IHG) and at first available IHG at year 6 in 73 FSWs who remained HIV− for at least 6 years. Half-moon arrows depict change in IHG distribution from baseline. **, *P*<0.01; ***; *P*<0.001. For boxplots, center line represents the median, box represents the IQR, whiskers represent the rest of the data distribution and outliers greater than ±1.5 × IQR are represented as points. Two-sided tests were used. Statistics are outlined in

Supplementary Information Section 11.4.4., *P* values are in Supplementary Data 14, and Source data are provided as Source data file.

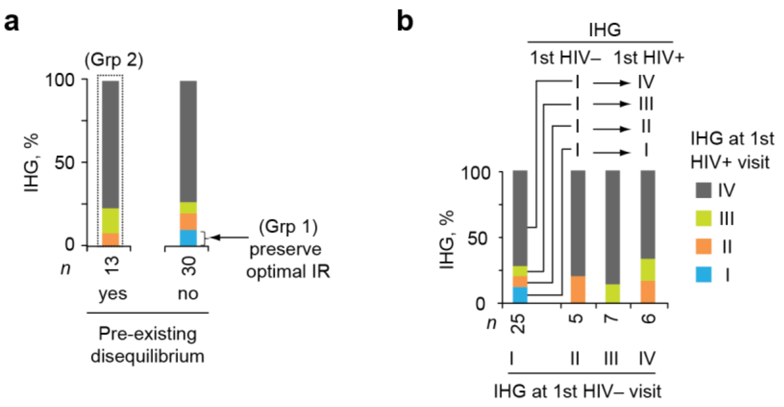

c

pre-HIV IHG		post-HIV IHG	*n*	% change (post- vs pre-HIV) CD8+	CD4+	Ratio	CD8+ expansion (Yes/No) Pre-existing	HIV-induced
I	→	I	3	–10.7	–12.1	1.0	No	Restrained
I	→	II	2	–38.8	–38.0	2.4	No	Suppressed
I	→	III	2	78.3	–10.4	–49.3	No	Yes
I	→	IV	18	46.6	–58.0	–68.5	No	Yes
II	→	II	1	–2.5	–12.3	–9.8	No	Restrained
II	→	IV	4	115.5	–24.8	–63.4	No	Yes
III	→	III	1	34.6	11.5	–16.7	Yes	Yes
III	→	IV	6	12.8	–40.8	–33.4	Yes	Restrained
IV	→	II	1	–11.6	30.5	47.4	Yes	Restrained
IV	→	III	1	11.6	47.2	31.1	Yes	Restrained
IV	→	IV	4	2.7	–13.2	–13.6	Yes	Restrained

Supplementary Fig. 5. Baseline immune health grade (IHG) and HIV risk and seroconversion. (**a**) Proportion of post-infection IHGs in HIV+ female sex workers (FSWs) by whether they had CD4:CD8 disequilibrium grades IHG-III or IHG-IV at baseline in 43 FSWs who have longitudinal data while HIV− and at least one CD4+ and CD8+ T-cell count measurement within 1 year of seroconversion. (**b**) Distribution of post-infection IHGs (outcomes) by pre-infection IHGs (predictor) in 43 FSWs who acquired HIV. Pre-infection IHGs were calculated using the first available CD4+ T-cell counts and CD4:CD8 ratio before HIV seroconversion for each subject. Post-infection IHGs were calculated using the first available CD4+ T-cell counts and CD4:CD8 ratio after HIV seroconversion for each subject. (**c**) Pre- vs. post-HIV seroconversion changes in IHG status in 43 FSWs who acquired HIV with accompanying percent change in CD4+, CD8+ T-cell counts and ratio between first available measurements before and after HIV seroconversion. CD8+ expansion was classified as suppressed, restrained, and yes based on % change in CD8+ T-cell counts of <–20, –20 to 20, and >20, respectively. Statistics are outlined in Supplementary Information Section 11.4.5. Source data are provided as Source data file.

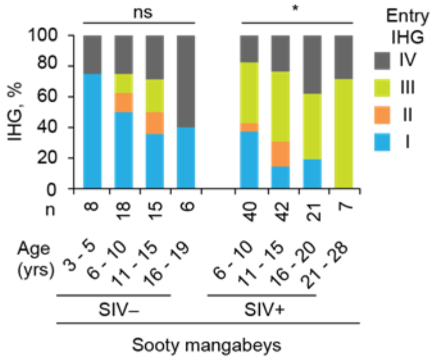

Supplementary Fig. 6. Immune health grade (IHG) by age and simian immunodeficiency virus (SIV) serostatus in Sooty mangabeys. Distribution of IHGs stratified by SIV status and age groups in the sooty mangabeys. ns, nonsignificant; *, *P*<0.05; ***; *P*<0.001. Two-sided tests were used. Statistics are outlined in Supplementary Information Section 11.4.6., *P* values are in Supplementary Data 14, and Source data are provided as Source data file.

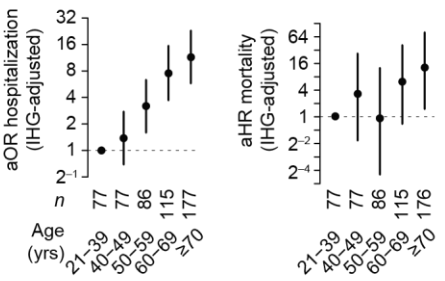

Supplementary Fig. 7. COVID-19 outcomes with age. Immune health grade (IHG)-adjusted odds ratios of hospitalization (with 95% confidence intervals) stratified by categorical age and IHG-adjusted hazard ratios of 30-day mortality stratified by categorical age in the COVID-19 cohort. Source data are provided as Source data file.

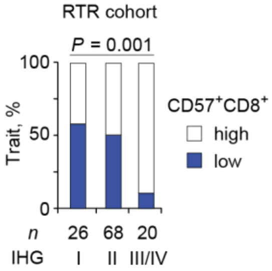

Supplementary Fig. 8. Immune traits in renal transplant recipients (RTRs). Levels of CD57+CD8+ T-cells (high vs. low) in RTRs by immune health grade (IHG) status. High and low CD57+CD8+ T-cell levels were defined by the median values in the studied population. Two-sided tests were used. Statistics are outlined in Supplementary Information Section 11.4.8., *P* values are in Supplementary Data 14, and Source data are provided as Source data file.

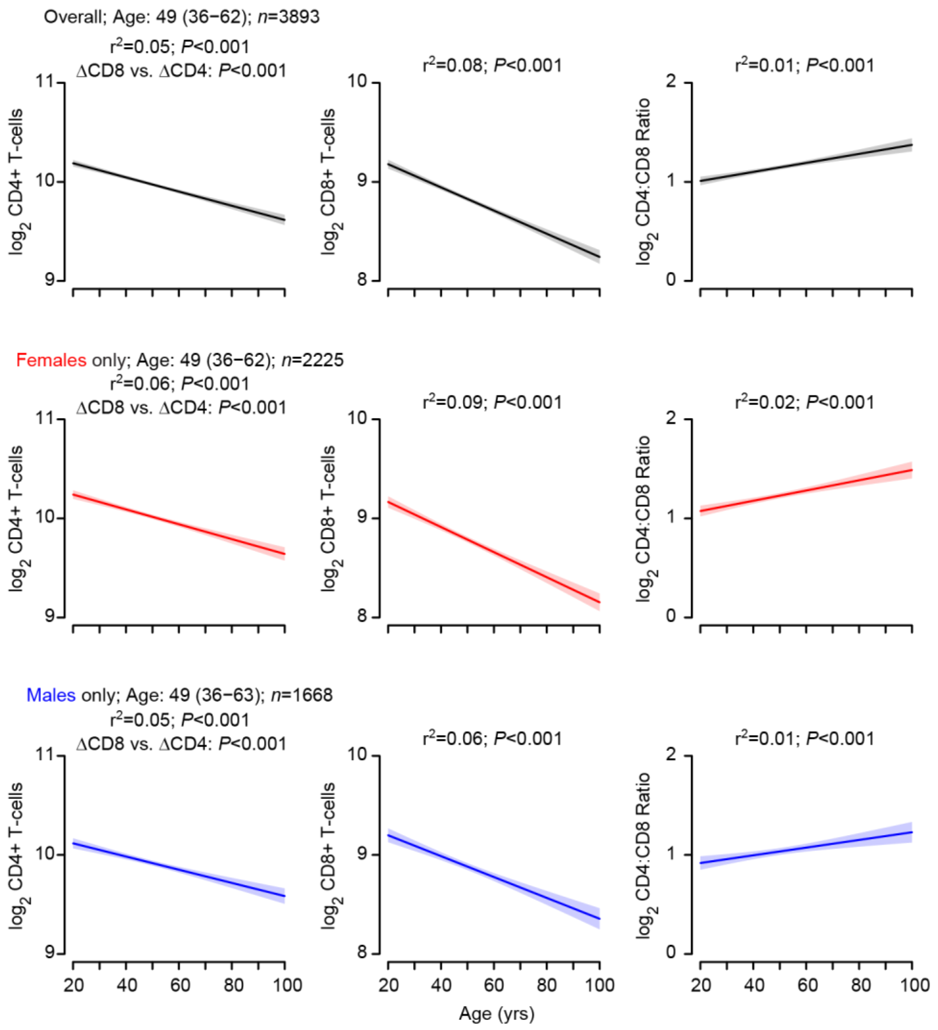

Supplementary Fig. 9. Changes in CD4+ and CD8+ T-cell counts and CD4:CD8 ratio with age. Line plots of fitted values with 95% confidence bands for Log_2 transformed data of CD4+, CD8+ T-cell counts, and CD4:CD8 ratio vs. age in the HIV− SardiNIA cohort overall and by sex (red, females; blue; males). Two-sided tests were used. Statistics are outlined in Supplementary Information Section 11.4.9., *P* values are in Supplementary Data 14, and Source data are provided as Source data file.

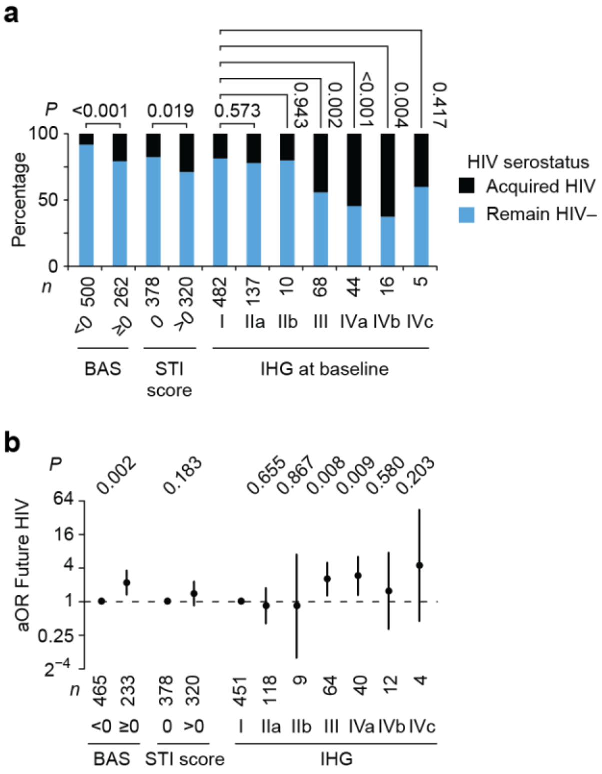

Supplementary Fig. 10. Future HIV seroconversion in female sex workers (FSWs). (**a**) Proportion of future HIV seroconversion by behavioral activity score (BAS) strata, sexually transmitted infection (STI) score strata, and immune health grades (IHGs) with subgrades. (**b**) Adjusted odds ratios (aORs) with 95% confidence intervals of future HIV seroconversion generated using regression by BAS strata, STI score strata, and IHGs with subgrades. Predictors adjusted by each other. Two-sided tests were used. Statistics are outlined in Supplementary Information Section 11.4.10., *P* values are in Supplementary Data 14, and Source data are provided as Source data file.

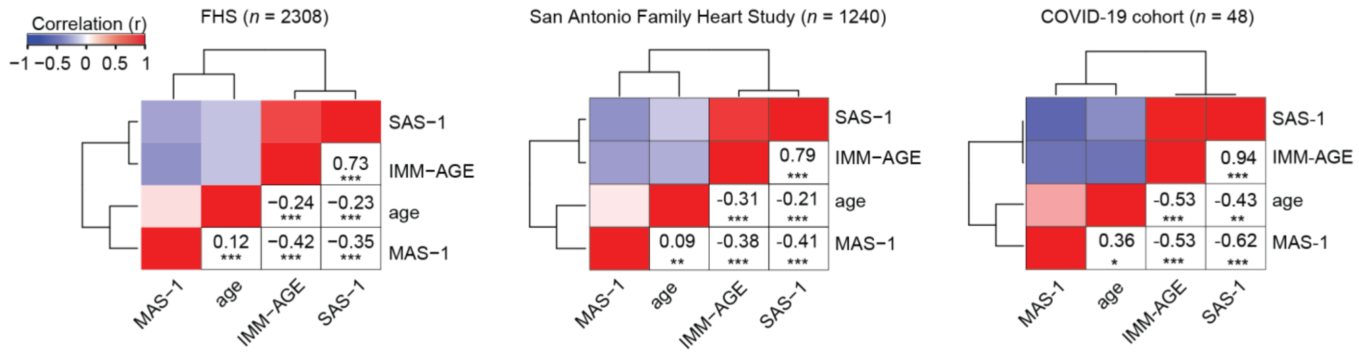

Supplementary Fig. 11. Survival-associated signature-1 (SAS-1) and mortality-associated signature-1 (MAS-1) correlations. Unsupervised hierarchical clustering of SAS-1, MAS-1, IMM-AGE, and age in the Framingham Heart Study (FHS) cohort, San Antonio Family Heart Study, and COVID-19 cohort. *, *P*<0.05; **, *P*<0.01; ***; *P*<0.001. Two-sided tests were used. Statistics are outlined in Supplementary Information Section 11.4.11., *P* values are in Supplementary Data 14, and Source data are provided as Source data file.

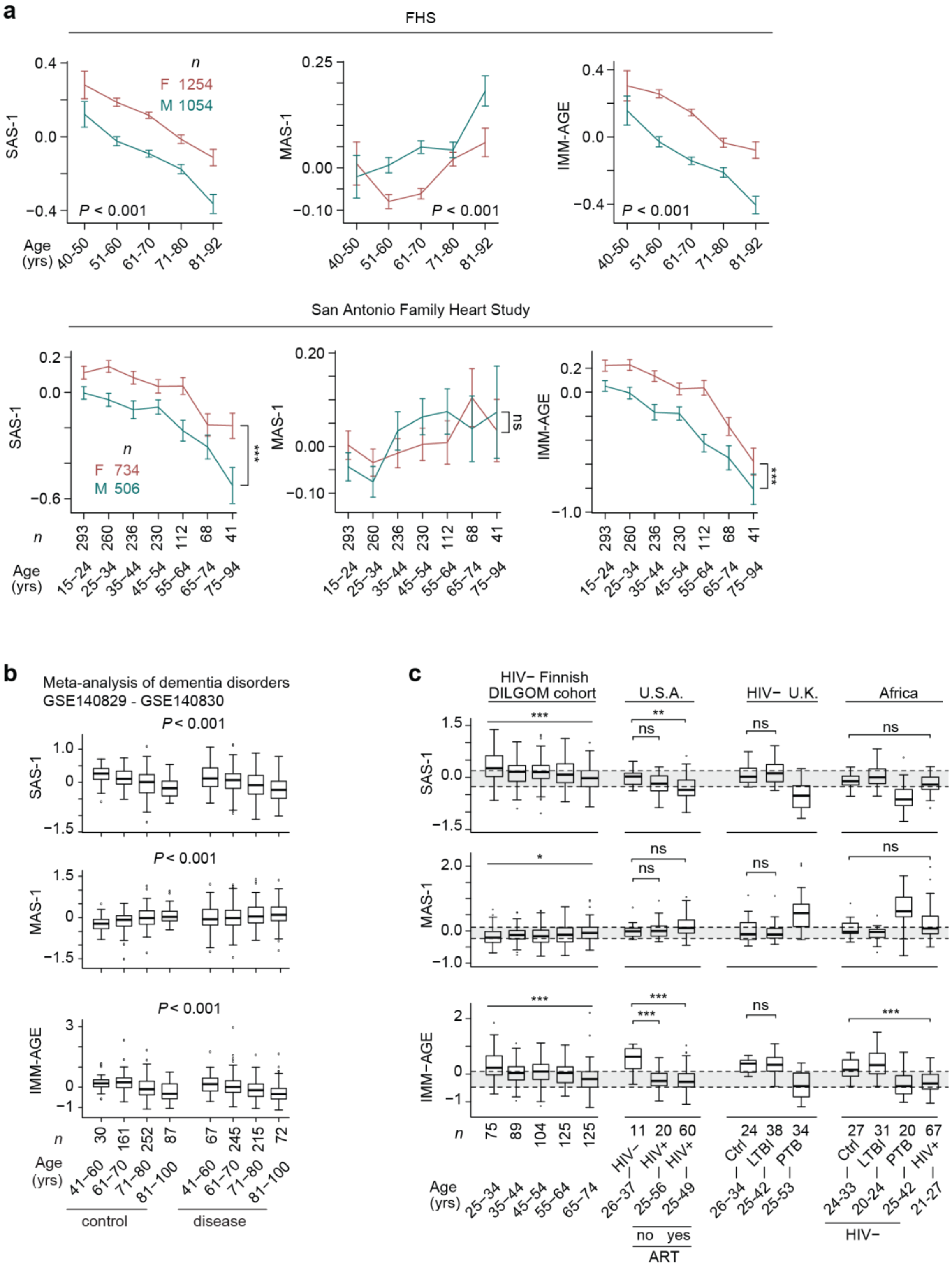

Supplementary Fig. 12. Gene signature scores in aging and infection cohorts. (**a**) Scores (mean ± SEM) stratified by age and sex in the Framingham Heart Study (FHS) offspring cohort and San Antonio Family Heart Study. (**b**) Gene signature scores in the meta-analysis of dementia studies stratified by indicated age bins within controls vs. disease (**c**) Gene signature scores in the meta-analysis of gene expression data sets from whole blood of HIV− or HIV+ persons from Finland, the USA, the UK, and Africa. Primary (PTB) and latent (LTBI) tuberculosis infection status is indicated.

HIV− persons were otherwise healthy. Antiretroviral therapy (ART) status (yes/no) of HIV+ persons is indicated. Horizontal dashed line and grey band: interquartile range (IQR) of transcriptomic signature score in HIV− healthy older persons (65-74 years) from the Finnish cohort. Representative cohorts from Finland, the USA, the UK, and Africa (Sources: meta-analysis of E-TABM-1036, GSE29429, GSE19439, GSE19442, GSE19444). ns, nonsignificant; *, $P<0.05$; **, $P<0.01$; ***; $P<0.001$. For boxplots, center line represents the median, box represents the interquartile range (IQR), whiskers represent the rest of the data distribution, and outliers greater than ±1.5 × IQR are represented as points. Two-sided tests were used. Statistics are outlined in Supplementary Information Section 11.4.12., *P* values are in Supplementary Data 14, and Source data are provided as Source data file.

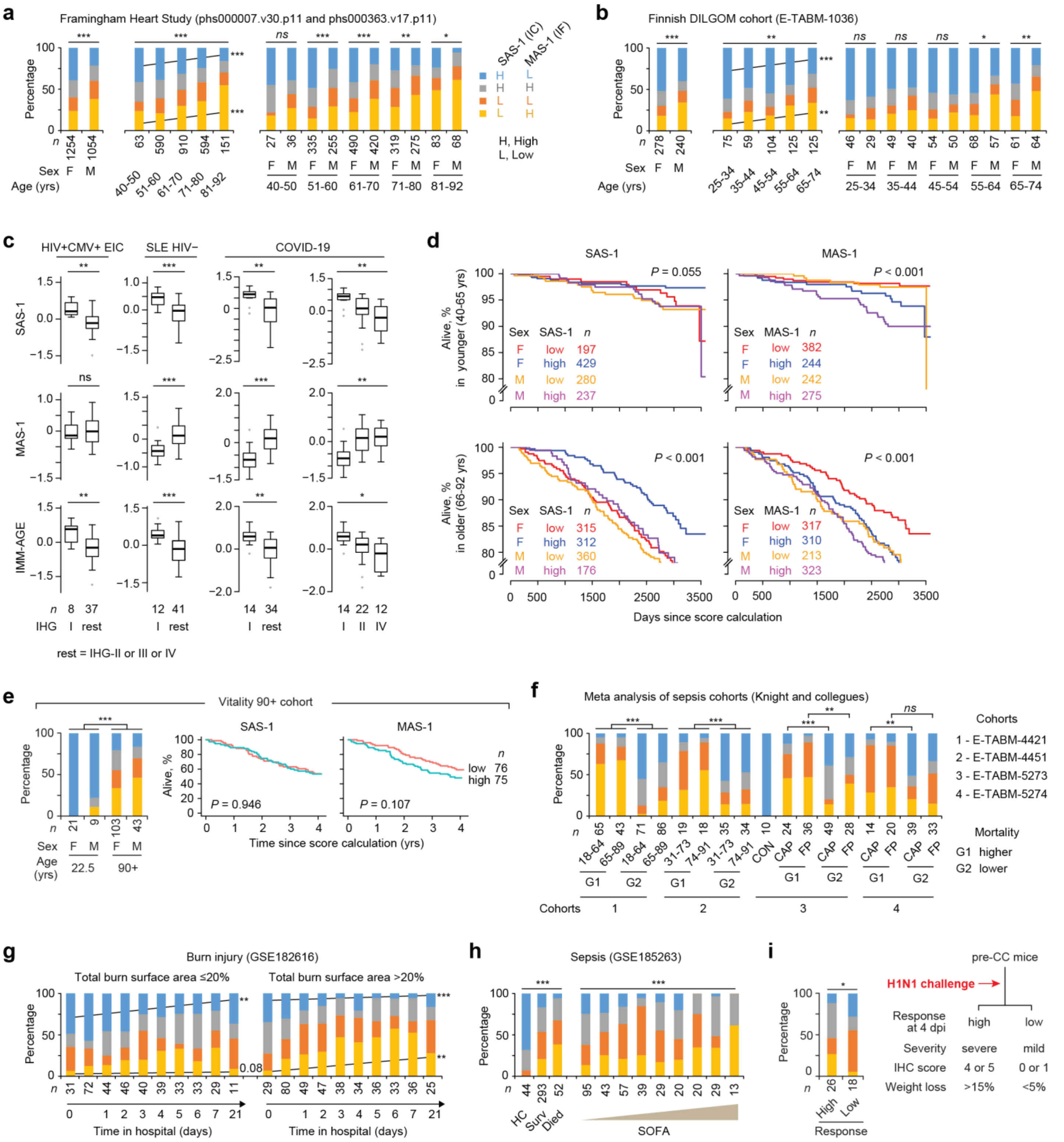

Supplementary Fig. 13. Gene signature scores with aging, infection, sepsis, burn injury, and flu response. (**a-b**, **e-i**) Distribution of survival-associated signature-1 (SAS-1)/mortality-associated signature-1 (MAS-1) profiles in: (**a**) Framingham Heart Study (FHS) and (**b**) Finnish DILGOM cohort stratified by sex and age; (**e**) young and nonagenarian participants stratified by sex; (**f**) Sepsis cohorts comprising individuals with community-acquired pneumonia (CAP) or fecal peritonitis (FP) stratified by sepsis response signature groups (G1 and G2 associated with higher and lower mortality, respectively)

and age; (**g**) Burn injury patients longitudinally sampled through hospitalization stratified by total burn surface area ≤20% or >20%; (**h**) Healthy controls and hospitalized patients with sepsis stratified by mortality and sequential organ failure assessment (SOFA) score. Categorical bins were defined as having a SOFA score of 0 (*n*=95), 1 (*n*=43), 2 (*n*=57), 3 (*n*=39), 4 (*n*=29), 5 (*n*=20), 6-7 (*n*=20), 8-12 (*n*=29), or 13-16 (*n*=13); and (**i**) pre-CC (Collaborative Cross) mice classified into high vs. low response groups at day 4 post infection (4 dpi) with influenza. (**c**) Gene signature scores comparing immune health grade (IHG)-I vs. the rest (IHGs II, III, and IV) in the HIV+CMV+ participants from early infection cohort (EIC), HIV− systemic lupus erythematosus (SLE) patients, and COVID-19 cohorts, with additional comparisons by overall IHG status in the COVID-19 cohort. (**d**) Proportion of patients who survived over 9-year follow-up in the FHS stratified by categorical age (determined using median age of 66 yrs; younger vs. older) and combinations of sex and median (low/high) expression of SAS-1 and MAS-1 scores. (**e**) In addition to distribution of SAS-1/MAS-1 profile, the proportion that survived over 4 years in nonagenarian (*n*=151) participants stratified by median-based low/high categorical groups of SAS-1 and MAS-1 scores. ns, nonsignificant; *, $P<0.05$; **, $P<0.01$; ***; $P<0.001$. For boxplots, center line represents the median, box represents the interquartile range (IQR), whiskers represent the rest of the data distribution and outliers greater than ±1.5 × IQR are represented as points. Two-sided tests were used. Statistics are outlined in Supplementary Information Section 11.4.13., *P* values are in Supplementary Data 14, and Source data are provided as Source data file.

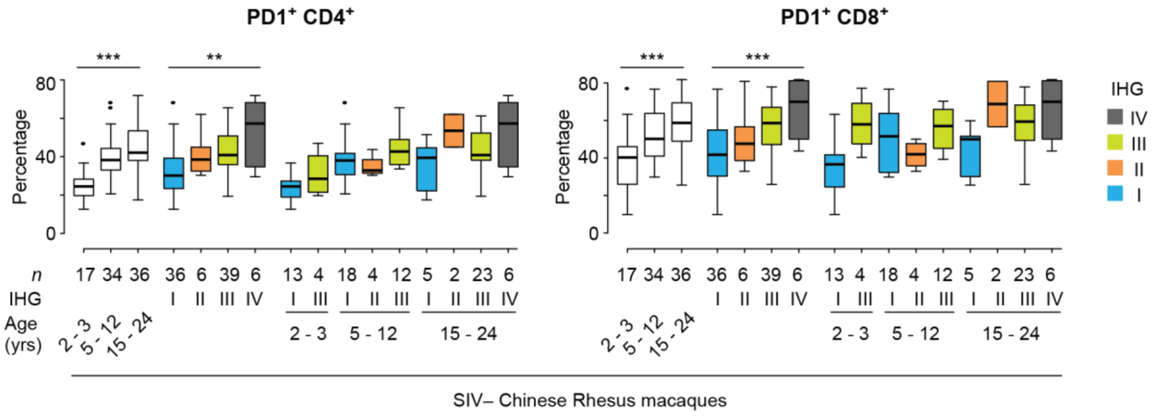

Supplementary Fig. 14. Association between immune health grades (IHGs) and immunophenotypes in non-human primates. Expression of PD-1 on CD4+ and CD8+ T-cells by age, IHG status, and age in simian immunodeficiency virus (SIV)– Chinese Rhesus macaques. ***P*<0.01, ****P*<0.001. For boxplots, center line represents the median, box represents the interquartile range (IQR), whiskers represent the rest of the data distribution and outliers greater than ±1.5 × IQR are represented as points. Two-sided tests were used. Statistics are outlined in Supplementary Information Section 11.4.14., *P* values are in Supplementary Data 14, and Source data are provided as Source data file.

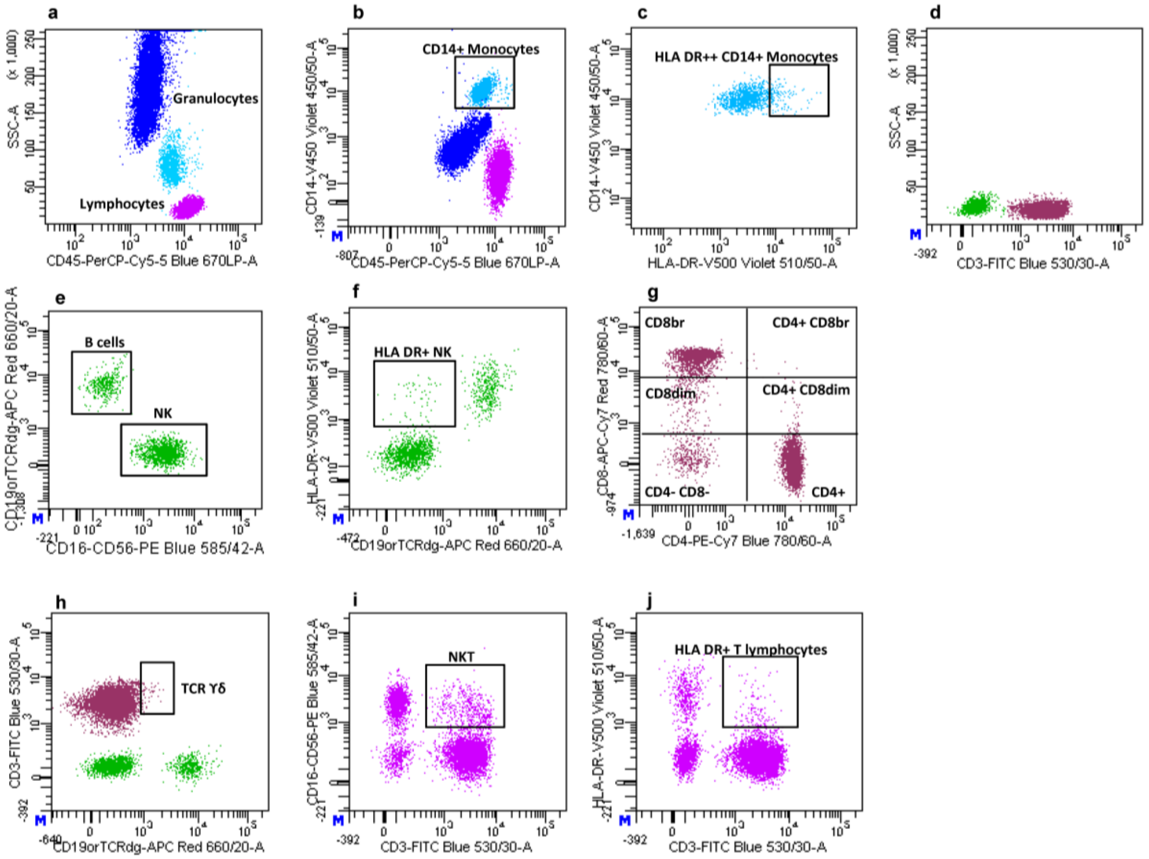

Supplementary Fig. 15. Flow cytometry gating strategy and phenotype assessment related to the T-cell, B-cell, and NK-cell antibody panel. (**a**) Lymphocytes (violet) and granulocytes (dark blue) were identified by their morphological properties and CD45 expression level. (**b**) Monocytes (light blue) were identified according to the CD45 expression and CD14 brightness. (**c**) HLA-DR++ monocytes (light blue) were identified according to the CD14 expression and HLA-DR brightness. (**d**) Lymphocytes were divided into CD3+ (T-cells, purple) and CD3– (green) populations. (**e**) CD3– cells were divided into B and NK cells based on the CD19 and CD16 and/or CD56 expression levels, respectively. (**f**) HLA DR activation marker was analyzed on NK-gated cells. (**g**) T-cells were assessed for CD4 and CD8 expression levels and divided into 6 gates. Starting from the lower left quadrant and proceeding clockwise, the following populations were distinguished: CD4– CD8–, CD4– CD8dim (CD8dim), CD4– CD8bright (CD8br), CD4+ CD8br, CD4+ CD8dim, and CD4+ CD8- (CD4+). (**h**) TCR-$\gamma\delta$ T-cells were defined on the CD3 and TCR-$\gamma\delta$ expression. (**i**) NKT cells were identified considering their positivity for CD3, CD16, and/or CD56 antigens. (**j**) Activated T-cells were identified by their positivity for CD3 and HLA-DR antigens. See **Supplementary Data 12** for traits obtained by logic gates. Two-sided tests were used.

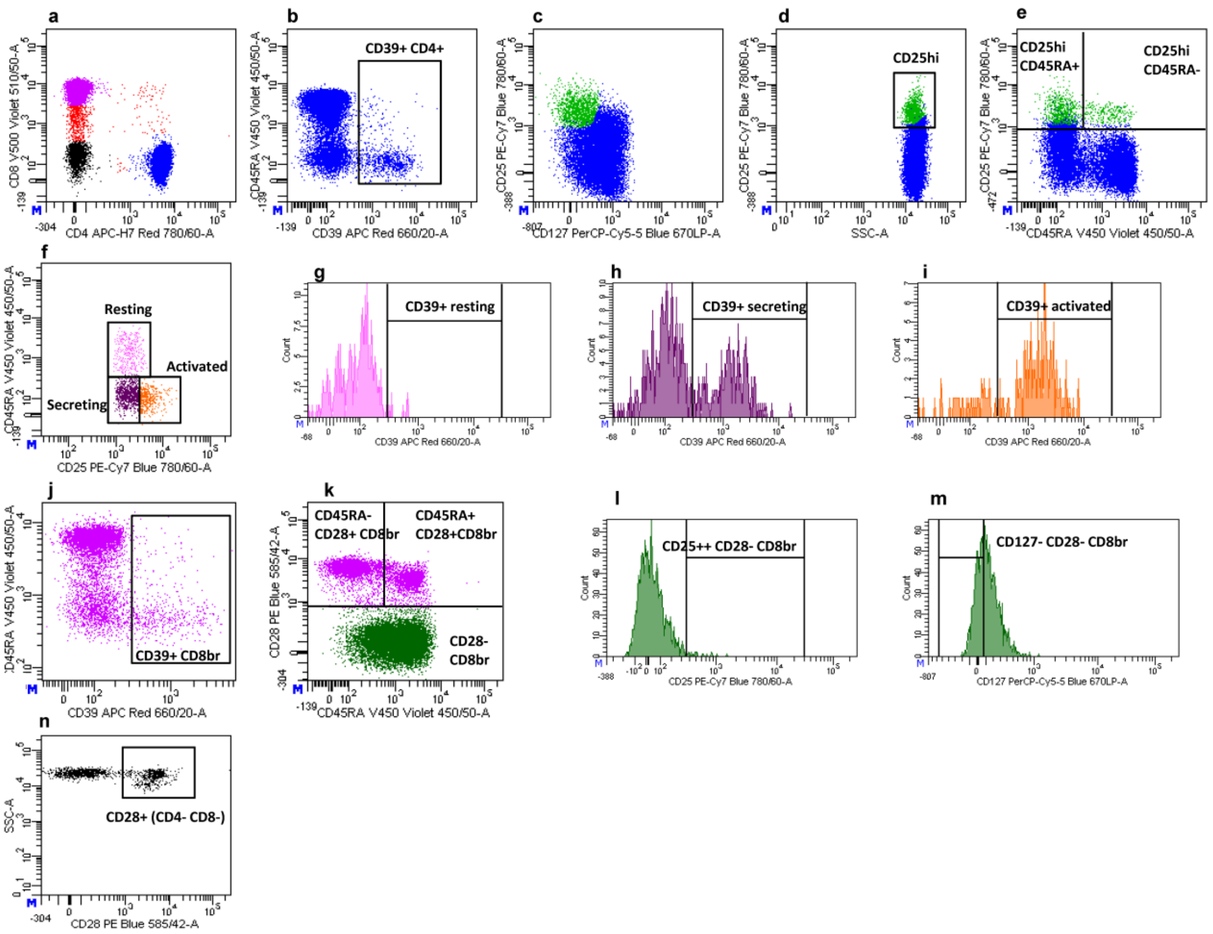

Supplementary Fig. 16. Flow cytometry gating strategy and phenotype assessment related to the regulatory T-cell antibody panel. (**a**) T-cells were assessed for CD4 and CD8 expression levels. (**b**) CD4+ subset (blue) was analyzed for CD39 expression. (**c**) CD4+ subset (blue) was analyzed for CD127 and CD25 markers to identify CD25hi CD127low cells, considered as CD4+ Tregs (green). (**d-e**) CD4+ cells were assessed for CD25 expression defining CD25 highly positive cells (CD4+ CD25hi) that include the CD4+ Tregs and other CD4+ subsets. Tregs were subtracted from total CD4+ CD25hi cells and the resulting population was further divided based on the expression of CD45RA. (**f**) CD4+ Tregs were divided into resting (CD45RA+ CD25++, pink), activated (CD45RA– CD25+++, orange) and secreting (CD45RA– CD25++, violet). (**g-i**) Resting, secreting, and activated CD4+ Treg cells were analyzed for the CD39 activation. (**j**) CD8br T-cells were analyzed for CD39 expression. (**k**) CD8br cells were divided into 3 subsets based on the expression of CD45RA and CD28 antigens. The same division in 3 subsets has also been applied to CD8dim (plot not shown). (**l-m**) CD8br CD28– cells were assessed for CD25 high expression (CD25++ CD28– CD8br) and for the low/absent CD127 expression (CD127– CD28– CD8br), respectively. (**n**) CD28 positivity was measured on CD4– CD8– T-cells. See **Supplementary Data 12** for traits obtained by logic gates. Two-sided tests were used.

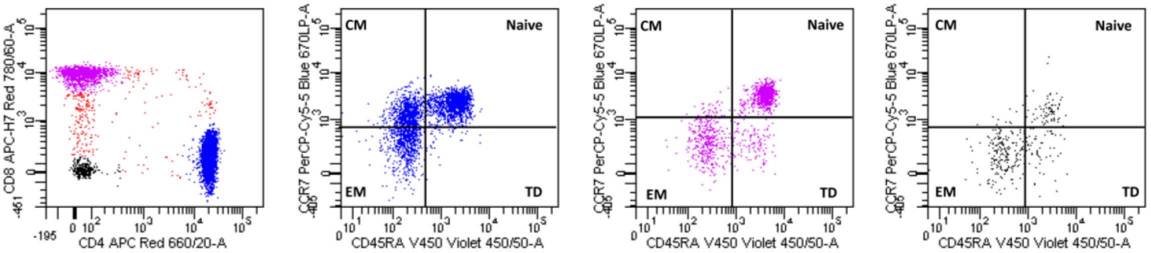

Supplementary Fig. 17. Flow cytometry gating strategy and traits assessment related to the maturation stages of T-cell antibody panel. CD4+ (blue), CD8br (violet), and CD4–CD8– (black) T-cells were divided in 4 maturation subsets according to the expression of CD45RA (X-axis) and CCR7 (Y-axis). Starting from the lower left quadrant and proceeding clockwise, we find the effector memory (EM, CCR7– CD45RA–), the central memory (CM, CCR7+ CD45RA–); the naïve cells (CCR7+ CD45RA+), and the terminally differentiated cells (TD, CCR7– CD45RA+). See **Supplementary Data 12** for traits obtained by logic gates.

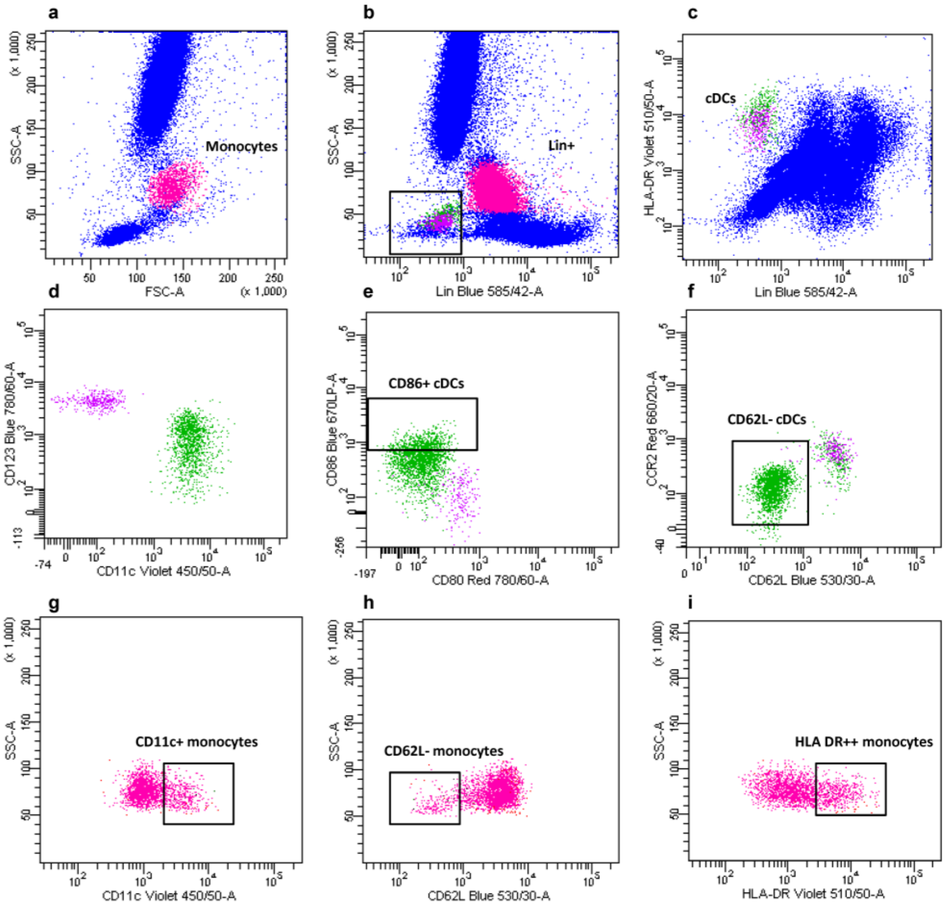

Supplementary Fig. 18. Flow cytometry gating strategy and traits assessment related to the circulating dendritic cell (DC) antibody panel. (**a-b**) Monocytes (bright pink) were obtained intersecting monocytes identified by morphological parameters and Lin+ cells. (**c**) Circulating DCs were characterized by their negativity for Lineage cocktail and positivity for HLA-DR antigen. (**d**) Circulating DCs were divided in CD11c+ (myeloid – conventional DCs, cDCs, green) and CD123+ (plasmacytoid, pDC, violet). (**e-f**) Circulating DCs were assessed for the co-stimulatory marker CD86 and the adhesion molecule CD62L. (**g-i**) Monocytes were analyzed for the CD11c, CD62L, and HLA DR markers. See **Supplementary Data 12** for traits obtained by logic gates.

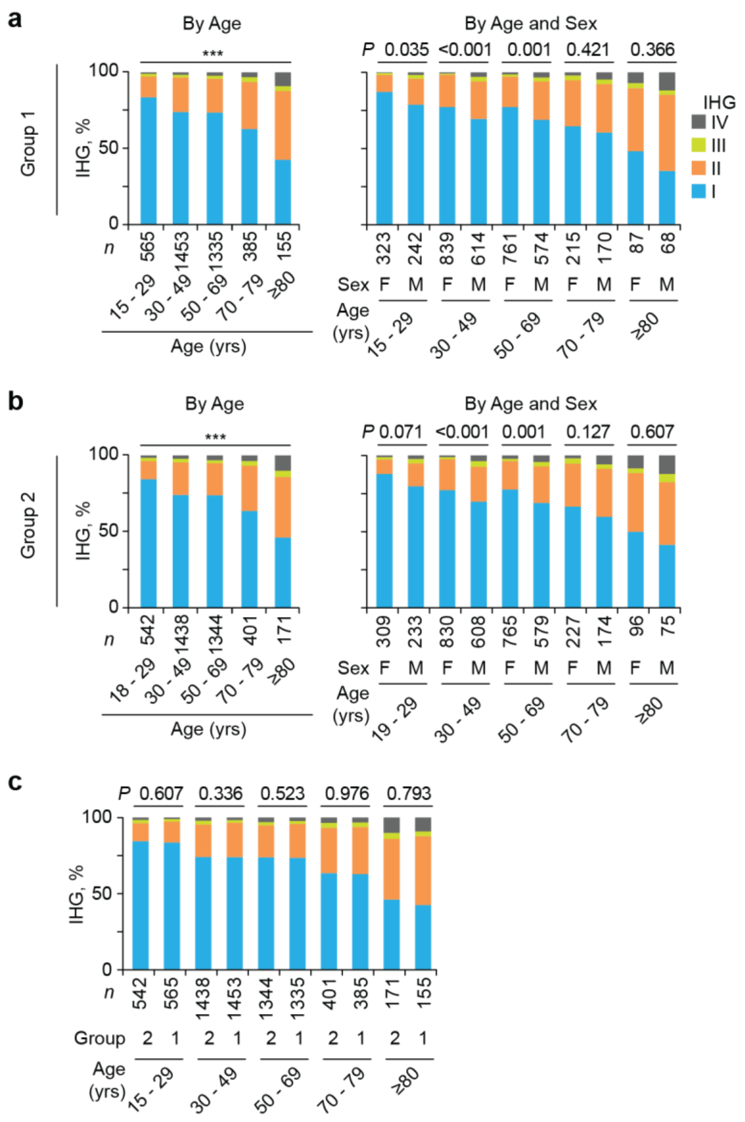

Supplementary Fig. 19. Comparison of SardiNIA groups 1 and 2. Barplots depicting the proportion of each IHG by age strata, sex within age strata in (**a**) group 1 and (**b**) group 2, and (**c**) group within age strata. Group features are noted in Supplementary Information Section 1.1.1. *P*, determined by χ^2 test. Two-sided tests were used. Statistics are outlined in Supplementary Information Section 11.4.19., *P* values are in Supplementary Data 14, and Source data are provided as Source data file.

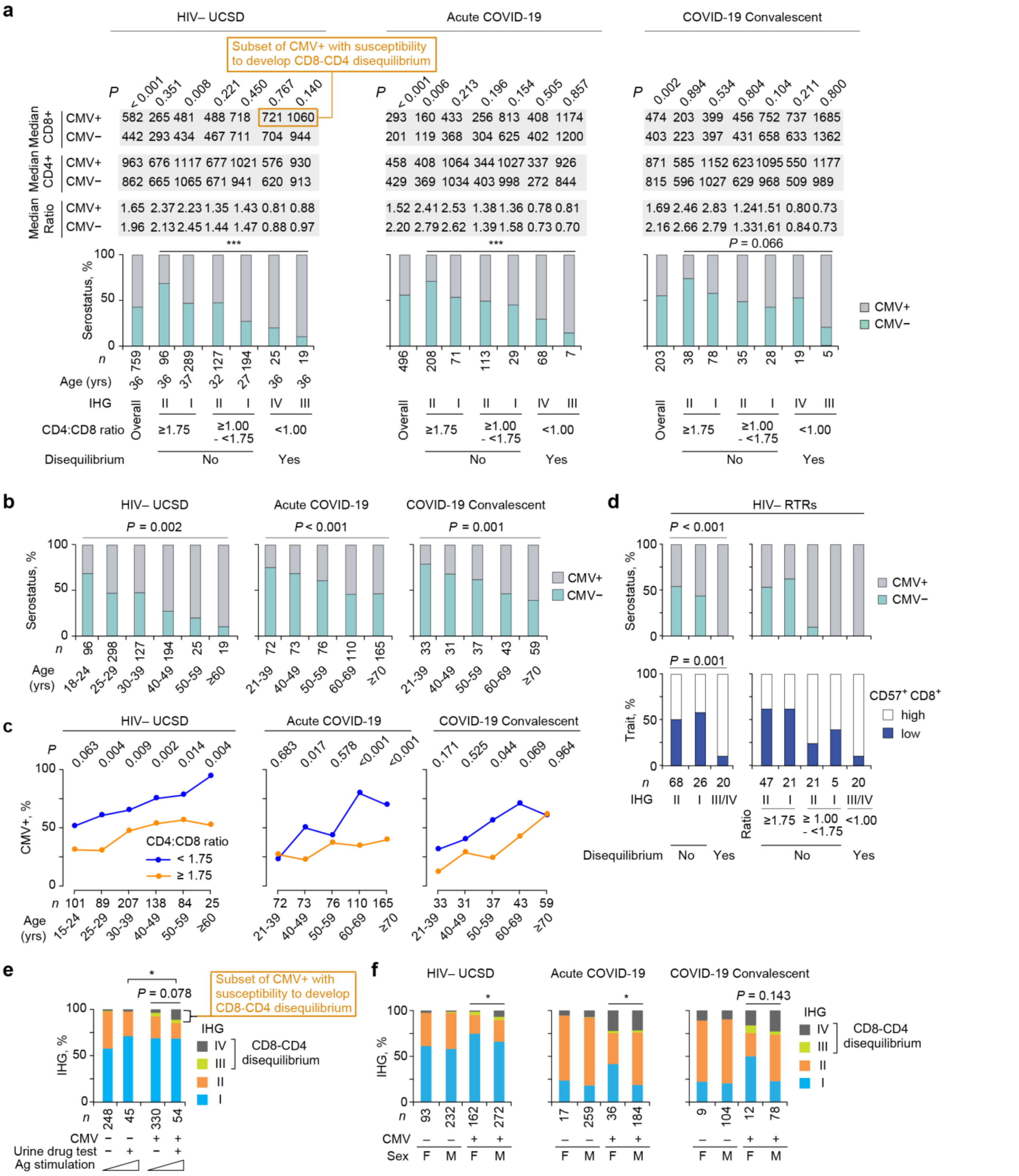

Supplementary Fig. 20. CD8-CD4 equilibrium vs. disequilibrium and CMV serostatus. (a) In (left to right) HIV− UCSD, acute COVID-19, and convalescent COVID-19 cohorts. Top: Median CD8+ cell counts, CD4+ cell counts, and CD4:CD8 ratio by CMV serostatus in overall and by CD8-CD4 equilibrium status with equilibrium status (IHG-I or IHG-II) subdivided by CD4:CD8 ratio (cutoff of 1.75, representing the median ratio value in otherwise healthy persons (**Supplementary Table 1**). *P* comparing CD8+ T-cell counts by CMV serostatus. Bottom, proportion CMV serostatus. **(b)** Proportion CMV serostatus by age strata in (left to right) HIV− UCSD, acute COVID-19,

and convalescent COVID-19 cohorts. **(c)** Line plots depicting the proportion of CMV seropositivity in age strata in those with a CD4:CD8 ratio <1.75 and those with a CD4:CD8 ratio ≥ 1.75 in (left to right) HIV− UCSD, acute COVID-19, and convalescent COVID-19 cohorts. *P* value comparing proportion CMV+ by CD4:CD8 ratio within each age strata. **(d)** Proportion CMV serostatus (top) and $CD57^{+}CD8^{+}$ T-cells (high/low; bottom) by IHG status (left) or IHG-I or IHG-II subdivided by CD4:CD8 ratio (right). **(e)** Proportions of IHG by CMV serostatus and urine drug test status. **(f)** Proportions of IHG by CMV serostatus and sex in (left to right) HIV− UCSD, acute COVID-19, and convalescent COVID-19 cohorts. Two-sided tests were used. Statistics are outlined in Supplementary Information Section 11.4.20., *P* values are in Supplementary Data 14, and Source data are provided as Source data file.

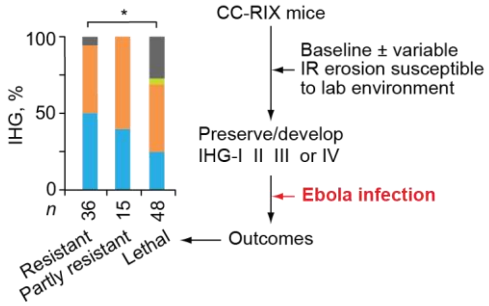

Supplementary Fig. 21. IHG distribution in the uninfected counterparts of CC-RIX mice grouped by outcomes after Ebola infection. Two-sided tests were used. Statistics are outlined in Supplementary Information Section 11.4.21., *P* values are in Supplementary Data 14, and Source data are provided as Source data file.

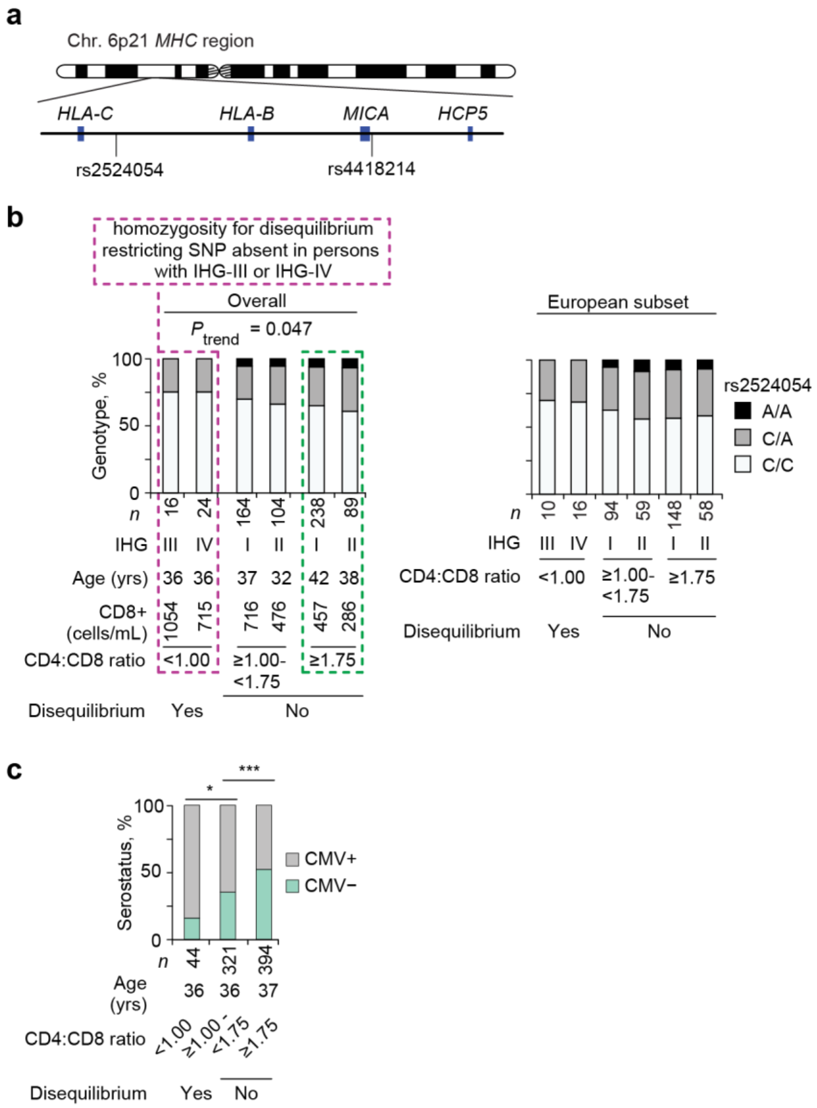

Supplementary Fig. 22. Host genetic features associated with susceptibility vs. resistance to develop CD8-CD4 disequilibrium. (**a**) Map of major histocompatibility locus and single-nucleotide polymorphisms (SNPs) associated with CD8+ levels in HIV-seronegative persons (rs2524054) and for reference lower HIV viral load in HIV+ persons (rs4418214[164]). (**b**) Distribution of the rs2524054 SNP in all HIV− UCSD participants (*left*) and European subset of the cohort (*right*) categorized as having CD8-CD4 disequilibrium (IHG-III or IHG-IV) vs. equilibrium (IHG-I or IHG-II) stratified by ratio cut-off of 1.75. Note, IHGs ranked according to median CD8+ levels by IHG and CD8-CD4 disequilibrium vs. equilibrium status. (**c**) CMV serostatus by CD8-CD4 disequilibrium vs. equilibrium status conflating IHG-I and IHG-II and stratifying ratio levels between ≥1 to 1.75 vs. ≥ 1.75. Median age in each study group is depicted. **P*<0.05, ****P*<0.001. Two-sided tests were used. Statistics are outlined in Supplementary Information Section 11.4.22., *P* values are in Supplementary Data 14, and Source data are provided as Source data file.

a

	Variables	15-39 yrs (younger) *n*	median	IQR	≥70 yrs (older) *n*	median	IQR	older vs. younger % difference	Wilcoxon-*P*
IHG-I	CD4+	986	1199	1005 - 1472	308	1081	931 - 1238	– 9.9	< 0.001
	CD8+	986	570	454 - 725	308	406	289 - 574	– 28.7	< 0.001
	CD4+:CD8+ ratio	986	2.18	1.74 - 2.72	308	2.66	2.00 - 3.69	+22.2	< 0.001
IHG-II	CD4+	216	689	610 - 752	189	621	526 - 709	– 9.9	< 0.001
	CD8+	216	377	295 - 470	189	288	191 - 389	– 23.5	< 0.001
	CD4+:CD8+ ratio	216	1.75	1.37 - 2.25	189	2.13	1.51 - 2.93	+21.7	< 0.001
IHG-III	CD4+	23	1046	885-1238	17	996	912-1140	-4.8	0.626
	CD8+	23	1281	1027-1410	17	1217	1070-1392	-5.0	0.787
	CD4+:CD8+ ratio	23	0.9	0.81-0.96	17	0.91	0.78-0.94	1.1	0.584
IHG-IV	CD4+	18	658	594-713	26	553	430-670	-15.9	0.034
	CD8+	18	752	674-857	26	807	589-998	7.3	0.456
	CD4+:CD8+ ratio	18	0.86	0.77-0.93	26	0.72	0.60-0.82	-16.4	0.005

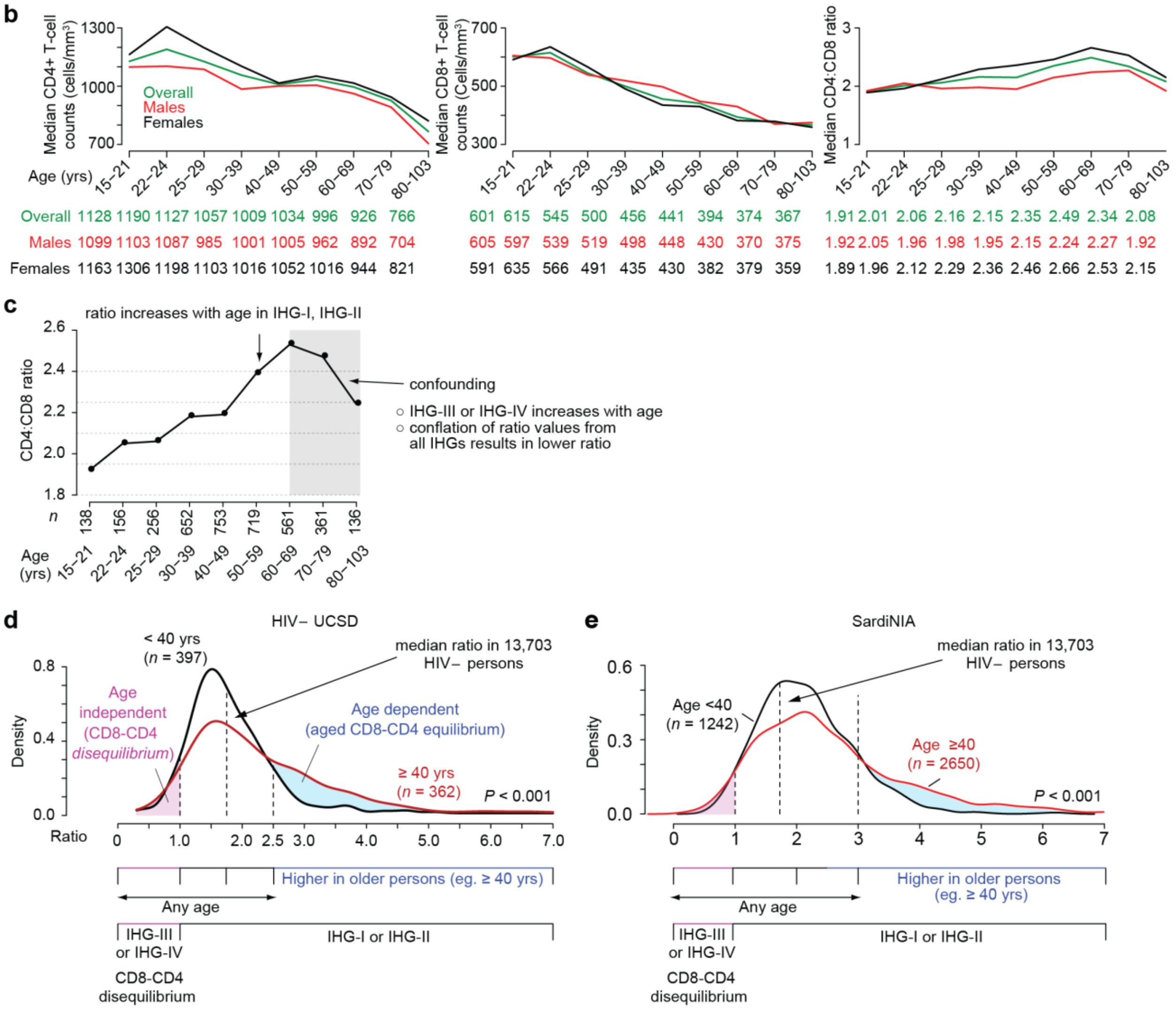

Supplementary Fig. 23. Age-appropriate evaluations of CD4+ and CD8+ counts and the CD4:CD8 ratio: accounting for IHGs mitigates confounding. (**a**) The number of participants and median and IQR of CD4+ T-cell counts, CD8+ T-cell counts, and CD4:CD8 ratio by age strata and IHG in the SardiNIA cohort. The %difference refers to difference in median values between older (≥70 years old) vs. younger (<40 years old) participants. (**b**) Line plots of median CD4+ and CD8+ T-cell counts as well as CD4:CD8 T-cell ratio by age and sex in the SardiNIA cohort. (**c**) Line plots of overall median CD4:CD8 ratio by age in the SardiNIA cohort depicting the basis for confounding. (**d,e**) CD8-CD4

equilibrium states in the HIV− UCSD (**d**) and SardiNIA cohorts (**e**). Kernel density estimates plots of the CD4:CD8 T-cell ratio and the corresponding CD8-CD4 equilibrium/disequilibrium states noted on the x-axis by ages: <40 vs. ≥40 years old. *P* values for differences in distribution. Vertical line at 1.0 represents the cut-off to demarcate CD8-CD4 disequilibrium status, i.e., persons with IHG-III or IHG-IV (indicated by red shaded area). Of note, the intersection of the plots at ratio of 2.5 in panel **d** and at ratio of 3 in panel **e** is indicated by vertical lines. suggesting that older persons with IHGs I or II with ratio ≥2.5 or ≥3 may show sign of aged CD8-CD4 equilibrium (i.e., age dependent changes in immune profiles; indicated by blue shaded area). In our literature survey of 13,703 HIV− persons worldwide, the median CD4:CD8 ratio was 1.76 (IQR: 1.57-2.04; **Supplementary Table 1**) and is indicated by a vertical dashed line. Two-sided tests were used. Statistics are outlined in Supplementary Information Section 11.4.23., *P* values are in Supplementary Data 14, and Source data are provided as Source data file.

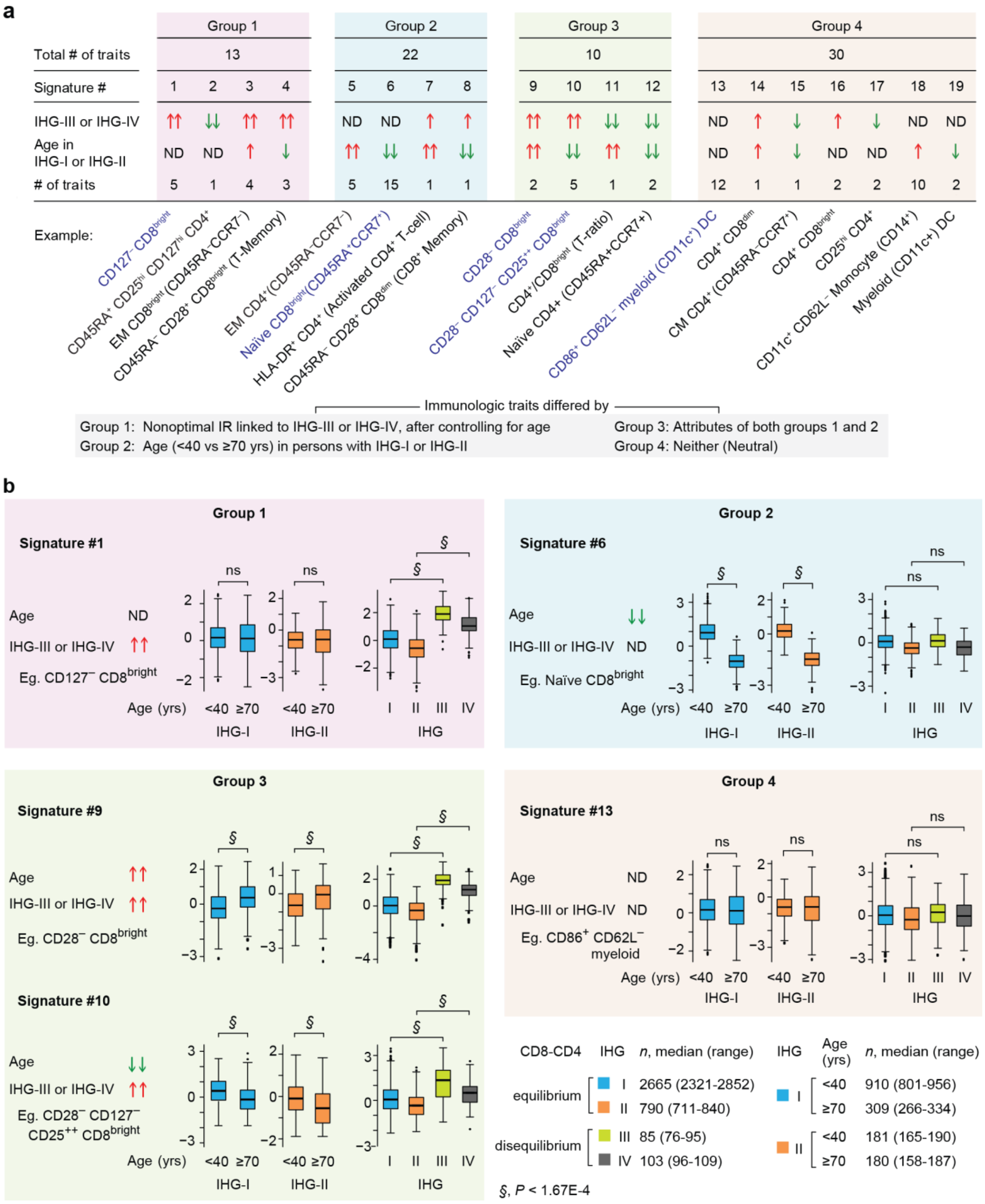

Supplementary Fig. 24. Immunologic traits associated with nonoptimal IR linked to CD8-CD4 disequilibrium grades IHG-III or IHG-IV) and/or age in those preserving IHG-I or IHG-II, and neither (neutral). (**a**) 75 immune traits sub-grouped into 19 signatures that cluster into four groups in the HIV− SardiNIA cohort with representative examples of cell types from each signature. cDC, conventional dendritic cells. Arrows indicate significant difference at P<1.67E-4; ND indicates no difference at P<1.67E-4. Two arrows indicate both comparisons for IHG-I vs. IHG-III and IHG-II vs. IHG-IV or age within IHG-I and IHG-II are significant, one arrow indicates only one of the comparisons

for IHG status or age is significant. (**b**) Boxplots of representative traits in younger vs. older persons with IHG-I or IHG-II (the most and second-most prevalent grades in humans, respectively) and by IHG status. Comparisons were made between IHG-I vs. IHG-III and IHG-II vs. IHG-IV to mitigate the confounding effects of higher and lower CD4+ counts, respectively in these sets of IHGs. Trait levels were normalized using inverse normal transformations with values ranging from −3 to 3; boxplots show covariate-adjusted residuals. Bottom right, median number of individuals evaluated by IHG status and age within IHG-I or IHG-II. ns, not significant. For boxplots, center line represents the median, box represents the interquartile range (IQR), whiskers represent the rest of the data distribution and outliers greater than ±1.5 × IQR are represented as points. Two-sided tests were used. Statistics are outlined in Supplementary Information Section 11.4.24., *P* values are in Supplementary Data 14, and Source data are provided as Source data file.

F. Supplementary Tables

Supplementary Table 1. Survey by literature review of CD4+ T-cell counts and CD4:CD8 T-cell ratio in presumed or demonstrated HIV-1 seronegative individuals of European descent and African Americans.

Summary statistics for CD4+ T-cell counts and CD4:CD8 T-cell ratio[a]

	Number of Reports (Number of Individuals)	Weighted Mean (95% CI)	Median (IQR)	Range
**CD4+ T-cell counts**				
Caucasian	16 (11,037)	1,011 (1,005 – 1,017)	940 (834 – 1,030)	796 – 1,109
Mixed USA	8 (4,083)	1,006 (995 – 1,018)	998 (882 – 1,027)	771 – 1,075
African American	2 (1,006)	1,077 (1,054 – 1,099)	1,078 (1,055 – 1,100)	1,055 – 1,100
Total sample size	25 (16,126)	1,014 (1,008 – 1,019)	952 (840 – 1,036)	771 – 1,109
**CD4:CD8 ratio**				
Caucasian	15 (11,318)	1.87 (1.86 – 1.88)	1.77 (1.75 – 1.77)	1.40 – 2.58
Mixed USA	4 (1,892)	1.67 (1.63 – 1.71)	1.83 (1.71 – 2.02)	1.58 – 2.20
African American	1 (493)	1.70 (1.65 – 1.73)	1.70	–
Total sample size	19 (13,703)	1.84 (1.83 – 1.85)	1.76 (1.57 – 2.04)	1.40 – 2.58

Reports from which the summary statistics were derived

Race/ Ethnicity	Reason for Study	Age (Years) Mean (SD, range)	Sex	HIV Status	*n*	CD4+ counts (cells/mm^3) Mean (SD or SE, range) or Median (IQR)	CD4:CD8 ratio Mean (SD or SE, range) or Median (IQR)	Ref
**Caucasian**								
Australian	GN	Mean: 15 (range: 10 – 37)	52% Female	PN	2,538	Mean: 1,030 (SD: 270, range: 210 – 2,530)	Mean: 1.79 (SD: 0.52, range: 0.43 – 4.09)	30
Australian	GN	Mean: 14 (range: 10 – 22)	48% Female	PN	592	Mean: 1,040 (SD: 300, range: 200 – 2,800)	Mean: 1.73 (SD: 0.55, range: 0.32 – 3.93)	30
UK	GN	Mean: 50 (range: 19 – 80)	Female	PN	396	Mean: 870 (SD: 330, Range: 390 – 2,380)	Mean: 2.00 (SD: 0.84, range: 0.46 – 5.70)	30
UK/Belgium	RF	(range: 7 – 17)[b]	22% Female	PN	22	Median: 800 (IQR: 700 – 1,100)	Median: 1.3 (IQR: 1.1 – 1.4)	165
UK/Belgium	RF	(range: 18 – 70)[b]	55% Female	PN	101	Median: 800 (IQR: 700 – 1,100)	Median: 1.2 (IQR: 1.0 – 1.5)	165
Sweden	RF	(range: 20 – 39)[b]	Combined	PN	75	Mean: 1,020 (SE: 39)	Mean: 1.67 (SE: 0.06)	166
Sweden	RF	(range: 20 – 39)[b]	Male	PN	34	Mean: 929 (SE: 58)	Mean: 1.55 (SE: 0.09)	166
Sweden	RF	(range: 20 – 39[b]	Female	PN	41	Mean: 1,096 (SE: 52)	Mean: 1.77 (SE: 0.09)	166
Sweden	RF	(range: 40 – 59)[b]	Combined	PN	76	Mean: 834 (SE: 35)	Mean: 2.05 (SE: 0.11)	166
Sweden	RF	(range: 40 – 59)[b]	Male	PN	39	Mean: 718 (SE: 32)	Mean: 1.89 (SE: 0.14)	166

Supplementary Table 1. Continued.								
Race/ Ethnicity	Reason for Study	Age (Years)	Sex	HIV Status	*n*	CD4+ counts (cells/mm^3)	CD4:CD8 ratio	Ref
		Mean (SD, range)				Mean (SD or SE, range) or Median (IQR)	Mean (SD or SE, range) or Median (IQR)	
Sweden	CT	(range: 40 – 59)[b]	Female	PN	37	Mean: 956 (SE: 59)	Mean: 2.23(SE: 0.17)	[166]
Sweden	RF	(range: 60 – 79)[b]	Combined	PN	68	Mean: 796 (SE: 37)	Mean: 2.14 (SE: 0.16)	[166]
Sweden	RF	(range: 60 – 79)[b]	Male	PN	36	Mean: 722 (SE: 47)	Mean: 1.74 (SE: 0.17)	[166]
Sweden	RF	(range: 60 – 79)[b]	Female	PN	32	Mean: 880 (SE: 55)	Mean: 2.58 (SE: 0.25)	[166]
Germany	RF	(range: 19 – 85)[b]	Combined	PN	100	Median: 870 (IQR: 490 – 1,640)	Median: 1.9 (IQR: 0.9 – 5.0)	[167]
Germany	RF		Male	PN	50	Median: 830	Median: 2.0	[167]
Germany	RF		Female	PN	50	Median: 930	Median: 1.8	[167]
Switzerland	RF	Mean: 50 (range: 24 – 68)	Combined	PN	70	Median: 691 (IQR: 309 – 1,139)	Median: 2.1 (IQR: 1.0 – 5.0)	[168]
Switzerland	RF	Mean: 49 (range: 23 – 70)	Male	PN	44	Median: 656 (IQR: 336 – 1,126)	Median: 2.0 (IQR: 0.9 – 6.0)	[168]
Switzerland		Mean: 51 (range: 25 – 70)	Female	PN	26	Median: 761 (IQR: 314 – 1,270)	Median: 2.3 (IQR: 1.0 – 4.9)	[168]
Italy	RF	Mean: 37 (range: 18 – 70)	Combined	PN	946	Mean: 940 (range: 493 – 1,666)		[169]
Italy	RF	–	Male	PN	532	Mean: 902		[169]
Italy	RF	–	Female	PN	436	Mean: 989		[169]
England	RF	(range: 11 – 79)	Combined	Neg.	600	Mean: 830 (SD: 288, range: 410 – 1,540)	Mean: 1.51 (range: 0.66 – 3.52)	[170]
England	RF	Mean: 30 (range: 19 – 41)	Male	Neg.	50	Mean: 840 (SD: 285)	Mean: 1.56	[170]
England	RF	Mean: 31 (range: 20 – 49)	Female	Neg.	50	Mean: 1,050 (SD: 377)	Mean: 1.70	[170]
Belgium	RF	(range: 18 – 70)	46% Female	Neg.	271		Mean: 1.4 (SD: 0.6, range: 0.6 – 2.8)	[171]

Supplementary Table 1. Continued.								
Race/ Ethnicity	Reason for Study	Age (Years)	Sex	HIV Status	*n*	CD4+ counts (cells/mm^3)	CD4:CD8 ratio	Ref
		Mean (SD, range)				Mean (SD or SE, range) or Median (IQR)	Mean (SD or SE, range) or Median (IQR)	
UK	RF	–	Combined	Neg.	234	Mean: 831		[172]
UK	RF	Mean: 31 (range: 19 – 67)	Male	Neg.	91	Mean: 754		[172]
UK	RF	Mean: 28 (range:17 – 58)	Female	Neg.	195	Mean: 865		[172]
UK	RF	Mean: 33 (SD: 6; range: 23 – 44)	Male	PN	32	Mean: 954 (SD: 272; range: 460 – 1,430)	Mean: 1.4 (SD: 0.5; range: 0.7 – 2.3)	[173]
USA	RF	Mean: 38	Male	PN	3467	Mean: 1,100 (SD: 400)	Mean: 2.0 (SD: 0.7)	[174]
France	CT	–	–	Neg.	61	Mean: 807 (SD: 378)	Mean: 1.75 (SD: 0.49)	[175]
France	CT	–	Male	Neg.	16	Mean: 1,109 (SD: 399)	Mean: 1.40 (SD: 0.80)	[175]
France	CT	–	–	PN	12	Mean: 844 (SD: 247)		[176]
Italy	GN	–	Combined	PN	468	–	Mean: 2.13 (SD: 1.04; range: 0.39 – 7.43)	[177]
Italy	GN	Mean: 41 (SD: 15)	Male	PN	263	Mean: 903 (SD: 308)	Mean: 2.05 (SD: 1.10)	[177]
Italy	GN	Mean: 40 (SD: 16)	Female	PN	205	Mean: 1,018 (SD: 319)	Mean: 2.27 (SD: 1.06)	[177]
Netherlands	RF	–	–	Neg.	1356	Mean: 993 (SD: 319; range: 509 – 1,761)	Mean: 2.2 (SD: 1.0, range: 0.9 – 4.8)	[178]
Netherlands	RF	–	–	Neg.	678	Median: 930 (IQR: 490 – 1,750)	Median:1.96 (IQR:0.89 – 4.67)	[179]
Netherlands	CT	(range: 18 – 64)	~48% Female	PN	59	Median: 908 (IQR: 513 – 1,606)		[180]

Supplementary Table 1. Continued.								
Race/ Ethnicity	Reason for Study	Age (Years)	Sex	HIV Status	*n*	CD4+ counts (cells/mm^3)	CD4:CD8 ratio	Ref
		Mean (SD, range)				Mean (SD or SE, range) or Median (IQR)	Mean (SD or SE, range) or Median (IQR)	
**Mixed USA**								
Baltimore/LA	RF	(range: 18 – 60)	–	Neg.	2,787	Mean: 1,017 (SD: 329)		181
LA	RF	–	–	Neg.	743		Mean: 1.58 (SD: 0.66)	99
USA	CT	–	–	PN	19	Mean: 839 (SD: 276)		182
New Mexico	CT	(range: 21 – 53)	Combined	PN	20	Mean: 1,075 (SD: 586)		183
New Mexico	CT	Mean: 76 (range: 67 – 88)	Combined	PN	25	Mean: 924 (SD: 416)		183
New York	CT	Mean: 39 (SD: 6.7)	–	Neg.	34	Mean: 1,013 (SD: 274)		184
USA	CT	median: 25 (IQR: 18 – 30)	58% Female	Neg.	24	Median: 785 (IQR: 662 – 860)		185
USA	CT	median: 49 (IQR: 45 – 66)	54% Female	Neg.	24	Median: 869 (IQR: 658 – 1,111)		185
USA	RF	(range: 20 – 69)	Combined	PN	266	Mean: 1,036 (SD: 296; range: 294 – 1,590)	Mean: 1.83 (SD: 0.54; range: 0.83 – 3.04)	186
USA	RF		Male	PN	143		Mean: 2.01 (SE: 0.07)	186
USA	RF		Female	PN	123		Mean: 2.25 (SE: 0.09)	186
California	CT	Median:38 (IQR: 20 – 58)	65% Female	PN	49	Mean: 771 (range: 326 – 1,344)		187
US Air Force	RF	Mean: 49	Male	Neg.	883	Mean: 982 (range: 417 – 1,841)	Mean: 2.2 (range: 0.7 – 4.5)	188
**African Americans**								
USA	CT	Median: 32	Female	Neg.	513	Mean: 1,055 (SE: 15)		189
USA	RF	Mean: 38 (range: 31 – 45)	Male	PN	493	Mean: 1,100 (SD: 400)	Mean: 1.7 (SD: 0.6)	174

**Supplementary Table 1. Continued**
IQR, interquartile range. SD, standard deviation. SE, standard error. UK, United Kingdom. USA, United States of America. LA, Los Angeles. Mixed USA refers to CD4+ T-cell counts and CD4:CD8 ratio data from the USA where the number of individuals who were of European or African descent was not specified.<br><br>[a]Summary statistics were derived from the CD4+ T-cell counts or CD4:CD8 T-cell ratio values reported for presumed HIV-seronegative (PN) or documented HIV-1-seronegative (Neg.) individuals in the studies outlined in the lower half of this table. Reason for study refers to the main purpose of why the study was conducted. Genetics (GN) indicates that the study was conducted to test the genetic basis of inheritance of T-cell counts. Reference (RF) means that the study was conducted to obtain a reference CD4+ or ratio value specific for the healthy population studied. Control (CT) denotes that the group studied served as a control for a study in which the association of a process (e.g., aging, HIV+) with CD4+ T-cell counts or ratio was examined. In this instance, only the CD4+ T-cell counts or CD4:CD8 ratio values from the healthy individuals were used.<br><br>[b]The original publication did not report mean values.

Supplementary Table 2. Literature survey for the inducers and consequences of CD8-CD4 disequilibrium grades IHG-III or IHG-IV.

Disease conditions	Major study objectives	Age (yrs)	Race/ Geographic	Male (%)	Phenotype	*n*	%IHG-III or %IHG-IV	Ref
Normal aging	Combination of HIV─ populations	18-29	Mixed	45.5%	Younger	890	4.3	Current study and 42
		30-59			Middle aged	2,831	5.0	Current study and 166,170
		60-102			Older	2,561	11.2	Current study and 36,37,41,166,190
	Younger	Mean±SD 21±3	Australia, 90% White-British	50%	Younger	158	2.5	42
		18-29	Italy, Sardinia	42.9%	Younger	542	3.7	Current study
		18-29	North America, 84.2% European and Hispanic American	55.8%	Younger	190	7.4	Current study
	Middle-aged	Mean 30.4	UK	50%	Middle aged	100	11.0	170
		30-59	Italy, Sardinia	41.7%	Middle aged	2,202	4.7	Current study
		30-59	North America, 76.6% European and Hispanic American	69.4%	Middle aged	529	4.9	Current study
	Older	Range 60-95, mean 68.7±7.0	Brazil with 50.4% Caucasian	36.1%	Older	421	14.5	41
		60-94	Swedish KRIS, OCTO, and NONA	35.6%	Older	303	15.5	166
		88-92	Swedish		Older	132	7.6	36,37
					Older non-survivors	89	25.8	36,37
		60-102	Italy, Sardinia	44.8%	Older	1,152	6.9	Current study
		60-79	North America, 80.0% European and	77.5%	Older	40	10.0	Current study

			Hispanic American					
		66	Swedish HEXA-1	46.5%	Older	424	14.6	190
Sex by age	Combination of HIV– populations	18-29	Mixed	0%	Female	393	2.8	Current study
				100%	Male	339	6.8	
		30-59		0%	Female	1,445	2.9	
				100%	Male	1,286	6.8	
		60-102		0%	Female	871	6.9	Current study and 190
				100%	Male	745	11.5	
	Genotypic and phenotypic features in population-based SardiNIA cohort from Italy	18-29	Italy, Sardinia	0%	Female	309	2.6	Current study
				100%	Male	233	5.2	
		30-59		0%	Female	1,283	2.9	
				100%	Male	919	7.3	
		60-102		0%	Female	635	5.2	
				100%	Male	517	9.1	
	HIV— UCSD cohort	18-29	North American, European, and Hispanic American, 78.7%, African American 14.8%	0%	Female	84	3.6	Current study
				100%	Male	106	10.4	
		30-59		0%	Female	162	3.1	
				100%	Male	367	5.7	
		60-79		0%	Female	9	22.2	
				100%	Male	31	6.5	
	Swedish HEXA-1	66	Swedish	0%	Female	227	11.0	190
				100%	Male	197	18.8	
Health care staff	Ratio study among health care staff with exposure to microbes (pediatric hospital staff)	Range 22-65 Mean±SD 36±10	North American	29.4%	Health care staff	34	20.6	191
Aging exposure	Aging in developing country	Range 18.0-29.2 Mean±SD 24.2±3.4	Pakistan	100%	Younger	50	20.0	192
		Range 50.0-85.5 Mean±SD 67.3±8.7		100%	Older	50	54.0	
Helminthic infection	Impact of helminthic infection		Ethiopia		Ethiopians-1	76	40.8	193

	on CD4+ and CD8+ T-cell counts				Ethiopians-2	63	44.4	[194]
Hemophilia	Impact of impure clotting factors on CD4+ and CD8+ T-cell counts	Range 11-78 Mean 35.9	UK		Mild hemophilia	219	11.9	[170,195]
		Mean 8.1	Birmingham		Severe hemophilia A	37	48.6	[195]
Risk behaviors	Female sex workers (FSW)	Median IQR 31 (27-37)	Kenya	0%	FSW	1,050	18.7	Current study
	Risk factors for HIV infection	Mean 25.0	Italy	76.6%	IDU	681	21.0	[196]
	Drug use and immune response		Chicago		Heroin addicts	21	23.8	[197]
	Men who have sex with women (MSW) and men who have sex with men (MSM)	Median IQR 52 (48-60)	Amsterdam	100%	MSW	72	5.6	[198]
		Median IQR 53 (48-59)			Low risk MSM (≤10 partners)	254	9.1	
		Median IQR 51 (47-56)			High risk MSM (>10 partners)	114	21.9	
	Men who have sex with men (MSM)	Mean 33.7 (19-61)	UK	100%	MSM	100	27.0	[170]
Tetanus booster vaccine	Impact of vaccination on CD4+ and CD8+ T-cell counts	20-50	Netherlands	81.8%	1wk before vaccination	11	0.0	[199]
					1-2wk after vaccination	11	36.4	
Alloresponse	Impact of GVHD on CD4+ and CD8+ T-cell counts	3.2-60.9	USA	70%	No	22	13.6	[200]
					Yes	57	43.9	
Population survival	T-cell immune risk phenotype and survival in an older population-based cohort	Range 88-92	Sweden		Older survivors	132	7.6	[36,37]
					Older non-survivors	89	25.8	
Cognitive function	Older population-based cohorts for cognitive impairment	Range 86-94	Sweden		Cognitive intact	97	14.4	[36]
					Cognitive impaired	30	26.7	
Cancer	Virus-associated cancer incidence among HIV– MSM	Median (IQR) 35 (30-42)	American (non-black, 79.5%)	100%	Age, race, and smoking status	500	29.2	[201]

					matched control			
					Virus-associated cancer	32	56.3	
	Lung cancer incidence among HIV+ †	Median (IQR) 45 (39-52)	American (non-Hispanic white, 41%; non-Hispanic black, 48%)	98%	No lung cancer	21,389	88.6†	[202]
		Median (IQR) 50 (46-57)	American (non-Hispanic white, 46%; non-Hispanic black, 48%))	99%	Lung cancer diagnosed	277	93.5†	
	Untreated chronic lymphocytic leukemia	Range 48-83			Slow progressors	51	39.2	[39]
					Fast progressors	20	70.0	
Influenza	Vaccine response in older participants	69	Sweden	50%	Responder s	22	13.6	[38]
					Non-responders	66	33.3	
CMV	Healthy young students	Mean±SD 20.5±1.6	UK, 90% White-British	49.4%	CMV–	110	0.0	[42]
					CMV+	48	8.3	
	HIV– UCSD cohort (Young)	Median (IQR) 24 (22-26), range 18-29	North American, European, and Hispanic American, 85.1%	55.8%	CMV–	104	1.9	Current study
					CMV+	86	14	
	HIV– UCSD cohort (middle-aged)	Median (IQR) 42 (36-49), range 30-59	North American, European, and Hispanic American, 76.9%	69.4%	CMV–	209	2.4	Current study
					CMV+	320	6.6	
	HIV– UCSD cohort (Old)	Median (IQR) 62 (61-66), range 60-79	North American, European, and Hispanic American,	77.5%	CMV–	12	0.0	Current study
					CMV+	28	14.3	

			82.1%					
	Swedish HEXA-1	66	Swedish	46.5%	CMV–	97*	6.2	[190]
					CMV+	327	17.1	
HIV infection	HIV infection risk in FSWs	Median (IQR) 31 (27-37)	Kenya	0%	Remain HIV–	923	16.0	Current study
					Became HIV+	127	37.8	
Non-human primate	Chimpanzee	Median (range) 17.5 (7-34)		35%	SIV–, HIV–	32	21.9	[203]

*2 persons with equivocal CMV serostatus from Swedish HEXA-1 cohort was considered to be CMV–.
† The original data from [202] stratified the CD4:CD8 ratio as ≤1.0 and >1.0. CMV, cytomegalovirus

G. References

1. Pilia, G., *et al.* Heritability of cardiovascular and personality traits in 6,148 Sardinians. *PLoS Genet* **2**, e132 (2006).
2. Orru, V., *et al.* Genetic variants regulating immune cell levels in health and disease. *Cell* **155**, 242-256 (2013).
3. Pistis, G., *et al.* Rare variant genotype imputation with thousands of study-specific whole-genome sequences: implications for cost-effective study designs. *Eur J Hum Genet* **23**, 975-983 (2015).
4. Bandewar, S.V., Kimani, J. & Lavery, J.V. The origins of a research community in the Majengo Observational Cohort Study, Nairobi, Kenya. *BMC Public Health* **10**, 630 (2010).
5. Bottomley, M.J., Harden, P.N. & Wood, K.J. CD8+ Immunosenescence Predicts Post-Transplant Cutaneous Squamous Cell Carcinoma in High-Risk Patients. *J Am Soc Nephrol* **27**, 1505-1515 (2016).
6. Wamachi, A.N., *et al.* Increased ratio of tumor necrosis factor-alpha to interleukin-10 production is associated with Schistosoma haematobium-induced urinary-tract morbidity. *The Journal of infectious diseases* **190**, 2020-2030 (2004).
7. Wongsawat, J., *et al.* CD4 cell count criteria to determine when to initiate antiretroviral therapy in human immunodeficiency virus-infected children. *Pediatr Infect Dis J* **29**, 966-968 (2010).
8. Lee, G.C., *et al.* Immunologic resilience and COVID-19 survival advantage. *J Allergy Clin Immunol* **148**, 1176-1191 (2021).
9. Le, T., *et al.* Enhanced CD4+ T-cell recovery with earlier HIV-1 antiretroviral therapy. *The New England journal of medicine* **368**, 218-230 (2013).
10. Okulicz, J.F., *et al.* Influence of the timing of antiretroviral therapy on the potential for normalization of immune status in human immunodeficiency virus 1-infected individuals. *JAMA Intern Med* **175**, 88-99 (2015).
11. Okulicz, J.F., *et al.* Clinical outcomes of elite controllers, viremic controllers, and long-term nonprogressors in the US Department of Defense HIV natural history study. *The Journal of infectious diseases* **200**, 1714-1723 (2009).
12. Marconi, V.C., *et al.* Outcomes of highly active antiretroviral therapy in the context of universal access to healthcare: the U.S. Military HIV Natural History Study. *AIDS research and therapy* **7**, 14 (2010).
13. Marconi, V.C., *et al.* Cumulative viral load and virologic decay patterns after antiretroviral therapy in HIV-infected subjects influence CD4 recovery and AIDS. *PLoS One* **6**, e17956 (2011).
14. Krantz, E.M., *et al.* Elevated CD8 counts during HAART are associated with HIV virologic treatment failure. *Journal of acquired immune deficiency syndromes* **57**, 396-403 (2011).
15. Sumpter, B., *et al.* Correlates of preserved CD4(+) T cell homeostasis during natural, nonpathogenic simian immunodeficiency virus infection of sooty mangabeys: implications for AIDS pathogenesis. *J Immunol* **178**, 1680-1691 (2007).
16. Zheng, H.Y., Zhang, M.X., Pang, W. & Zheng, Y.T. Aged Chinese rhesus macaques suffer severe phenotypic T- and B-cell aging accompanied with sex differences. *Experimental gerontology* **55**, 113-119 (2014).

17. Rasmussen, A.L., *et al.* Host genetic diversity enables Ebola hemorrhagic fever pathogenesis and resistance. *Science* **346**, 987-991 (2014).
18. CDC. 1993 Revised classification system for HIV infection and expanded surveillance case definition for AIDS among adolescents and adults. . *MMWR Recomm Rep* **41**, 1-19 (1992).
19. Perneger, T.V. What's wrong with Bonferroni adjustments. *BMJ* **316**, 1236-1238 (1998).
20. Savitz, D.A. & Olshan, A.F. Multiple comparisons and related issues in the interpretation of epidemiologic data. *Am J Epidemiol* **142**, 904-908 (1995).
21. Rothman, K.J. No adjustments are needed for multiple comparisons. *Epidemiology* **1**, 43-46 (1990).
22. Bacchetti, P. Peer review of statistics in medical research: the other problem. *BMJ* **324**, 1271-1273 (2002).
23. Armstrong, J.S. Significance Tests Harm Progress in Forecasting. *International Journal of Forecasting* **23**, 321-327 (2007).
24. Cohen, J. The earth is round (p < .05). *American Psychologist* **49**, 997–1003 (1994).
25. Gigerenzer, G. Mindless statistics. *Journal of Behavioral and Experimental Economics (formerly The Journal of Socio-Economics)* **33**, 587-606 (2004).
26. Goodman, S.N. Toward evidence-based medical statistics. 1: The P value fallacy. *Ann Intern Med* **130**, 995-1004 (1999).
27. Gardner, M.J. & Altman, D.G. Confidence intervals rather than P values: estimation rather than hypothesis testing. *Br Med J (Clin Res Ed)* **292**, 746-750 (1986).
28. Lecoutre, B., Lecoutre, M.-P. and Poitevineau, J. . Uses, abuses and misuses of significance tests in the scientific community: won't the Bayesian choice be unavoidable? . *International Statistical Review* **69**, 399–417 (2001).
29. Silva-Aycaguer, L.C., Suarez-Gil, P. & Fernandez-Somoano, A. The null hypothesis significance test in health sciences research (1995-2006): statistical analysis and interpretation. *BMC Med Res Methodol* **10**, 44 (2010).
30. Ferreira, M.A., *et al.* Quantitative trait loci for CD4:CD8 lymphocyte ratio are associated with risk of type 1 diabetes and HIV-1 immune control. *Am J Hum Genet* **86**, 88-92 (2010).
31. Valiathan, R., *et al.* Reference ranges of lymphocyte subsets in healthy adults and adolescents with special mention of T cell maturation subsets in adults of South Florida. *Immunobiology* **219**, 487-496 (2014).
32. Yarmohammadi, H. & Cunningham-Rundles, C. Idiopathic CD4 lymphocytopenia: Pathogenesis, etiologies, clinical presentations and treatment strategies. *Ann Allergy Asthma Immunol* **119**, 374-378 (2017).
33. McBride, J.A. & Striker, R. Imbalance in the game of T cells: What can the CD4/CD8 T-cell ratio tell us about HIV and health? *PLoS Pathog* **13**, e1006624 (2017).
34. Margolick, J.B., *et al.* Impact of inversion of the CD4/CD8 ratio on the natural history of HIV-1 infection. *J Acquir Immune Defic Syndr* **42**, 620-626 (2006).
35. Serrano-Villar, S. & Deeks, S.G. CD4/CD8 ratio: an emerging biomarker for HIV. *Lancet HIV* (2015).
36. Wikby, A., *et al.* An immune risk phenotype, cognitive impairment, and survival in very late life: impact of allostatic load in Swedish octogenarian and nonagenarian humans. *J Gerontol A Biol Sci Med Sci* **60**, 556-565 (2005).

37. Wikby, A., *et al.* The immune risk phenotype is associated with IL-6 in the terminal decline stage: findings from the Swedish NONA immune longitudinal study of very late life functioning. *Mechanisms of ageing and development* **127**, 695-704 (2006).
38. Strindhall, J., *et al.* Humoral response to influenza vaccination in relation to pre-vaccination antibody titres, vaccination history, cytomegalovirus serostatus and CD4/CD8 ratio. *Infect Dis (Lond)* **48**, 436-442 (2016).
39. Nunes, C., *et al.* Expansion of a CD8(+)PD-1(+) replicative senescence phenotype in early stage CLL patients is associated with inverted CD4:CD8 ratios and disease progression. *Clin Cancer Res* **18**, 678-687 (2012).
40. Gonzalez-Rodriguez, A.P., *et al.* Prognostic significance of CD8 and CD4 T cells in chronic lymphocytic leukemia. *Leuk Lymphoma* **51**, 1829-1836 (2010).
41. Muller, G.C., *et al.* The inverted CD4:CD8 ratio is associated with gender-related changes in oxidative stress during aging. *Cell Immunol* **296**, 149-154 (2015).
42. Turner, J.E., *et al.* Rudimentary signs of immunosenescence in Cytomegalovirus-seropositive healthy young adults. *Age* **36**, 287-297 (2014).
43. Tao, C.J., *et al.* A prognostic model combining CD4/CD8 ratio and N stage predicts the risk of distant metastasis for patients with nasopharyngeal carcinoma treated by intensity modulated radiotherapy. *Oncotarget* **7**, 46653-46661 (2016).
44. Blueprint, W.R.D. WHO R&D blueprint novel coronavirus (COVID-19) therapeutic trial synopsis. (2020).
45. Miyara, M., *et al.* Functional delineation and differentiation dynamics of human CD4+ T cells expressing the FoxP3 transcription factor. *Immunity* **30**, 899-911 (2009).
46. Shevach, E.M. Regulatory T cells in autoimmmunity*. *Annu Rev Immunol* **18**, 423-449 (2000).
47. Keir, M.E. & Sharpe, A.H. The B7/CD28 costimulatory family in autoimmunity. *Immunol Rev* **204**, 128-143 (2005).
48. Sallusto, F., Lenig, D., Forster, R., Lipp, M. & Lanzavecchia, A. Two subsets of memory T lymphocytes with distinct homing potentials and effector functions. *Nature* **401**, 708-712 (1999).
49. Steinman, R.M. & Banchereau, J. Taking dendritic cells into medicine. *Nature* **449**, 419-426 (2007).
50. Ohnmacht, C., *et al.* Constitutive ablation of dendritic cells breaks self-tolerance of CD4 T cells and results in spontaneous fatal autoimmunity. *J Exp Med* **206**, 549-559 (2009).
51. Camargo, J.F., *et al.* Responsiveness of T cells to interleukin-7 is associated with higher CD4+ T cell counts in HIV-1-positive individuals with highly active antiretroviral therapy-induced viral load suppression. *The Journal of infectious diseases* **199**, 1872-1882 (2009).
52. Ahuja, S.K., *et al.* Preservation of epithelial cell barrier function and muted inflammation in resistance to allergic rhinoconjunctivitis from house dust mite challenge. *J Allergy Clin Immunol* **139**, 844-854 (2017).
53. Smith, A.M., *et al.* Repetitive aeroallergen challenges elucidate maladaptive epithelial and inflammatory traits that underpin allergic airway diseases. *J Allergy Clin Immunol* (2021).
54. Trapnell, C., Pachter, L. & Salzberg, S.L. TopHat: discovering splice junctions with RNA-Seq. *Bioinformatics* **25**, 1105-1111 (2009).

55. Anders, S. & Huber, W. Differential expression analysis for sequence count data. *Genome Biol* **11**, R106 (2010).
56. Benjamini, Y. & Hochberg, Y. Controlling the False Discovery Rate - a Practical and Powerful Approach to Multiple Testing. *Journal of the Royal Statistical Society Series B-Methodological* **57**, 289-300 (1995).
57. Alpert, A.*, et al.* A clinically meaningful metric of immune age derived from high-dimensional longitudinal monitoring. *Nat Med* **25**, 487-495 (2019).
58. Huang da, W., Sherman, B.T. & Lempicki, R.A. Bioinformatics enrichment tools: paths toward the comprehensive functional analysis of large gene lists. *Nucleic Acids Res* **37**, 1-13 (2009).
59. Huang da, W., Sherman, B.T. & Lempicki, R.A. Systematic and integrative analysis of large gene lists using DAVID bioinformatics resources. *Nat Protoc* **4**, 44-57 (2009).
60. Chiche, L.*, et al.* Modular transcriptional repertoire analyses of adults with systemic lupus erythematosus reveal distinct type I and type II interferon signatures. *Arthritis Rheumatol* **66**, 1583-1595 (2014).
61. Kent, J.W., Jr.*, et al.* Genotypexage interaction in human transcriptional ageing. *Mech Ageing Dev* **133**, 581-590 (2012).
62. Hazuda, H.P., Mitchell, B.D., Haffner, S.M. & Stern, M.P. Obesity in Mexican American subgroups: findings from the San Antonio Heart Study. *Am J Clin Nutr* **53**, 1529S-1534S (1991).
63. Mitchell, B.D.*, et al.* Genetic and environmental contributions to cardiovascular risk factors in Mexican Americans. The San Antonio Family Heart Study. *Circulation* **94**, 2159-2170 (1996).
64. Nachun, D.*, et al.* Systems-level analysis of peripheral blood gene expression in dementia patients reveals an innate immune response shared across multiple disorders. *bioRxiv* (2019).
65. Smith, A.J.*, et al.* The immunosuppressive role of IL-32 in lymphatic tissue during HIV-1 infection. *J Immunol* **186**, 6576-6584 (2011).
66. Li, Q.*, et al.* Microarray analysis of lymphatic tissue reveals stage-specific, gene expression signatures in HIV-1 infection. *J Immunol* **183**, 1975-1982 (2009).
67. Davenport, E.E.*, et al.* Genomic landscape of the individual host response and outcomes in sepsis: a prospective cohort study. *Lancet Respir Med* **4**, 259-271 (2016).
68. Burnham, K.L.*, et al.* Shared and Distinct Aspects of the Sepsis Transcriptomic Response to Fecal Peritonitis and Pneumonia. *Am J Respir Crit Care Med* **196**, 328-339 (2017).
69. Almansa, R.*, et al.* Transcriptomic evidence of impaired immunoglobulin G production in fatal septic shock. *J Crit Care* **29**, 307-309 (2014).
70. Almansa, R.*, et al.* Transcriptomic correlates of organ failure extent in sepsis. *J Infect* **70**, 445-456 (2015).
71. Zhai, Y.*, et al.* Host Transcriptional Response to Influenza and Other Acute Respiratory Viral Infections--A Prospective Cohort Study. *PLoS Pathog* **11**, e1004869 (2015).

72. Zaas, A.K., *et al.* Gene expression signatures diagnose influenza and other symptomatic respiratory viral infections in humans. *Cell Host Microbe* **6**, 207-217 (2009).
73. Woods, C.W., *et al.* A host transcriptional signature for presymptomatic detection of infection in humans exposed to influenza H1N1 or H3N2. *PLoS One* **8**, e52198 (2013).
74. Dunning, J., *et al.* Progression of whole-blood transcriptional signatures from interferon-induced to neutrophil-associated patterns in severe influenza. *Nat Immunol* **19**, 625-635 (2018).
75. Chang, H.H., *et al.* Transcriptional network predicts viral set point during acute HIV-1 infection. *J Am Med Inform Assoc* **19**, 1103-1109 (2012).
76. Berry, M.P., *et al.* An interferon-inducible neutrophil-driven blood transcriptional signature in human tuberculosis. *Nature* **466**, 973-977 (2010).
77. Inouye, M., *et al.* Metabonomic, transcriptomic, and genomic variation of a population cohort. *Mol Syst Biol* **6**, 441 (2010).
78. Borodulin, K., *et al.* Cohort Profile: The National FINRISK Study. *Int J Epidemiol* **47**, 696-696i (2018).
79. Inouye, M., *et al.* An immune response network associated with blood lipid levels. *PLoS Genet* **6**, e1001113 (2010).
80. Aho, V., *et al.* Prolonged sleep restriction induces changes in pathways involved in cholesterol metabolism and inflammatory responses. *Sci Rep* **6**, 24828 (2016).
81. Marttila, S., *et al.* Transcriptional analysis reveals gender-specific changes in the aging of the human immune system. *PLoS One* **8**, e66229 (2013).
82. Nosraty, L., Jylha, M., Raittila, T. & Lumme-Sandt, K. Perceptions by the oldest old of successful aging, Vitality 90+ Study. *J Aging Stud* **32**, 50-58 (2015).
83. Nosraty, L., Enroth, L., Raitanen, J., Hervonen, A. & Jylha, M. Do successful agers live longer? The Vitality 90+ study. *J Aging Health* **27**, 35-53 (2015).
84. Goebeler, S., Jylha, M. & Hervonen, A. Medical history, cognitive status and mobility at the age of 90. A population-based study in Tampere, Finland. *Aging Clin Exp Res* **15**, 154-161 (2003).
85. Keyloun, J.W., *et al.* Early Transcriptomic Response to Burn Injury: Severe Burns Are Associated With Immune Pathway Shutdown. *J Burn Care Res* **43**, 306-314 (2022).
86. Baghela, A., *et al.* Predicting sepsis severity at first clinical presentation: The role of endotypes and mechanistic signatures. *EBioMedicine* **75**, 103776 (2022).
87. Bottomly, D., *et al.* Expression quantitative trait Loci for extreme host response to influenza a in pre-collaborative cross mice. *G3 (Bethesda)* **2**, 213-221 (2012).
88. Imanishi, T. & Nakaoka, H. Hyperlink Management System and ID Converter System: enabling maintenance-free hyperlinks among major biological databases. *Nucleic Acids Res* **37**, W17-22 (2009).
89. Hope, J.L., Stairiker, C.J., Bae, E.A., Otero, D.C. & Bradley, L.M. Striking a Balance-Cellular and Molecular Drivers of Memory T Cell Development and Responses to Chronic Stimulation. *Front Immunol* **10**, 1595 (2019).
90. Davis, M.M., *et al.* T cells as a self-referential, sensory organ. *Annu Rev Immunol* **25**, 681-695 (2007).

91. Urra, J.M., Cabrera, C.M., Porras, L. & Rodenas, I. Selective CD8 cell reduction by SARS-CoV-2 is associated with a worse prognosis and systemic inflammation in COVID-19 patients. *Clin Immunol* **217**, 108486 (2020).
92. Mathew, D.*, et al.* Deep immune profiling of COVID-19 patients reveals distinct immunotypes with therapeutic implications. *Science* (2020).
93. Carter, M.J.*, et al.* Peripheral immunophenotypes in children with multisystem inflammatory syndrome associated with SARS-CoV-2 infection. *Nat Med* (2020).
94. Blanco-Melo, D.*, et al.* Imbalanced Host Response to SARS-CoV-2 Drives Development of COVID-19. *Cell* (2020).
95. Wang, F.*, et al.* The laboratory tests and host immunity of COVID-19 patients with different severity of illness. *JCI Insight* **5**(2020).
96. Chen, G.*, et al.* Clinical and immunological features of severe and moderate coronavirus disease 2019. *J Clin Invest* **130**, 2620-2629 (2020).
97. Wang, F.*, et al.* Characteristics of Peripheral Lymphocyte Subset Alteration in COVID-19 Pneumonia. *J Infect Dis* **221**, 1762-1769 (2020).
98. Hue, S.*, et al.* Uncontrolled Innate and Impaired Adaptive Immune Responses in Patients with COVID-19 ARDS. *Am J Respir Crit Care Med* (2020).
99. Taylor, J.M., Fahey, J.L., Detels, R. & Giorgi, J.V. CD4 percentage, CD4 number, and CD4:CD8 ratio in HIV infection: which to choose and how to use. *Journal of acquired immune deficiency syndromes* **2**, 114-124 (1989).
100. Vila, L.M.*, et al.* Systemic lupus erythematosus in a multiethnic US cohort, XXXVII: association of lymphopenia with clinical manifestations, serologic abnormalities, disease activity, and damage accrual. *Arthritis Rheum* **55**, 799-806 (2006).
101. Liu, M.F., Wang, C.R., Fung, L.L. & Wu, C.R. Decreased CD4+CD25+ T cells in peripheral blood of patients with systemic lupus erythematosus. *Scand J Immunol* **59**, 198-202 (2004).
102. Takahashi, T.*, et al.* Sex differences in immune responses that underlie COVID-19 disease outcomes. *Nature* (2020).
103. Gebhard, C., Regitz-Zagrosek, V., Neuhauser, H.K., Morgan, R. & Klein, S.L. Impact of sex and gender on COVID-19 outcomes in Europe. *Biol Sex Differ* **11**, 29 (2020).
104. Austad, S.N. & Fischer, K.E. Sex Differences in Lifespan. *Cell Metab* **23**, 1022-1033 (2016).
105. Zarulli, V.*, et al.* Women live longer than men even during severe famines and epidemics. *Proc Natl Acad Sci U S A* **115**, E832-E840 (2018).
106. Ostan, R.*, et al.* Gender, aging and longevity in humans: an update of an intriguing/neglected scenario paving the way to a gender-specific medicine. *Clin Sci (Lond)* **130**, 1711-1725 (2016).
107. Threadgill, D.W. & Churchill, G.A. Ten years of the Collaborative Cross. *Genetics* **190**, 291-294 (2012).
108. Effros, R.B. The silent war of CMV in aging and HIV infection. *Mech Ageing Dev* **158**, 46-52 (2016).
109. Tu, W. & Rao, S. Mechanisms Underlying T Cell Immunosenescence: Aging and Cytomegalovirus Infection. *Front Microbiol* **7**, 2111 (2016).
110. Pawelec, G. Immunosenenescence: role of cytomegalovirus. *Experimental gerontology* **54**, 1-5 (2014).

111. Alyazidi, R., Murthy, S., Slyker, J.A. & Gantt, S. The Potential Harm of Cytomegalovirus Infection in Immunocompetent Critically Ill Children. *Front Pediatr* **6**, 96 (2018).
112. Klenerman, P. & Oxenius, A. T cell responses to cytomegalovirus. *Nat Rev Immunol* **16**, 367-377 (2016).
113. Wang, H.*, et al.* Cytomegalovirus Infection and Relative Risk of Cardiovascular Disease (Ischemic Heart Disease, Stroke, and Cardiovascular Death): A Meta-Analysis of Prospective Studies Up to 2016. *J Am Heart Assoc* **6**(2017).
114. Griffiths, P.D. & Mahungu, T. Why CMV is a candidate for elimination and then eradication. *J Virus Erad* **2**, 131-135 (2016).
115. Richardson, A.K.*, et al.* Breast cancer and cytomegalovirus. *Clin Transl Oncol* (2019).
116. Simanek, A.M.*, et al.* Seropositivity to cytomegalovirus, inflammation, all-cause and cardiovascular disease-related mortality in the United States. *PLoS One* **6**, e16103 (2011).
117. Luz Correa, B.*, et al.* The inverted CD4:CD8 ratio is associated with cytomegalovirus, poor cognitive and functional states in older adults. *Neuroimmunomodulation* **21**, 206-212 (2014).
118. Pawelec, G., McElhaney, J.E., Aiello, A.E. & Derhovanessian, E. The impact of CMV infection on survival in older humans. *Current opinion in immunology* **24**, 507-511 (2012).
119. Kawasaki, M.*, et al.* Carotid atherosclerosis, cytomegalovirus infection, and cognitive decline in the very old: a community-based prospective cohort study. *Age (Dordr)* **38**, 29 (2016).
120. Spyridopoulos, I.*, et al.* CMV seropositivity and T-cell senescence predict increased cardiovascular mortality in octogenarians: results from the Newcastle 85+ study. *Aging Cell* **15**, 389-392 (2016).
121. Savva, G.M.*, et al.* Cytomegalovirus infection is associated with increased mortality in the older population. *Aging Cell* **12**, 381-387 (2013).
122. Huppert, F.A., Pinto, E.M., Morgan, K. & Brayne, C. Survival in a population sample is predicted by proportions of lymphocyte subsets. *Mech Ageing Dev* **124**, 449-451 (2003).
123. Freeman, M.L.*, et al.* CD8 T-Cell Expansion and Inflammation Linked to CMV Coinfection in ART-treated HIV Infection. *Clinical infectious diseases : an official publication of the Infectious Diseases Society of America* **62**, 392-396 (2016).
124. Colonna-Romano, G.*, et al.* Impact of CMV and EBV seropositivity on CD8 T lymphocytes in an old population from West-Sicily. *Exp Gerontol* **42**, 995-1002 (2007).
125. Stover, C.T.*, et al.* Prevalence of and risk factors for viral infections among human immunodeficiency virus (HIV)-infected and high-risk HIV-uninfected women. *J Infect Dis* **187**, 1388-1396 (2003).
126. Strioga, M., Pasukoniene, V. & Characiejus, D. CD8+ CD28- and CD8+ CD57+ T cells and their role in health and disease. *Immunology* **134**, 17-32 (2011).
127. Vuddamalay, Y. & van Meerwijk, J.P. CD28(-) and CD28(low)CD8(+) Regulatory T Cells: Of Mice and Men. *Front Immunol* **8**, 31 (2017).

128. Nikolich-Zugich, J. Ageing and life-long maintenance of T-cell subsets in the face of latent persistent infections. *Nat Rev Immunol* **8**, 512-522 (2008).
129. Stelekati, E.*, et al.* Bystander chronic infection negatively impacts development of CD8(+) T cell memory. *Immunity* **40**, 801-813 (2014).
130. Wherry, E.J. & Ahmed, R. Memory CD8 T-cell differentiation during viral infection. *J Virol* **78**, 5535-5545 (2004).
131. Olsson, J.*, et al.* Age-related change in peripheral blood T-lymphocyte subpopulations and cytomegalovirus infection in the very old: the Swedish longitudinal OCTO immune study. *Mechanisms of ageing and development* **121**, 187-201 (2000).
132. Goronzy, J.J. & Weyand, C.M. Understanding immunosenescence to improve responses to vaccines. *Nat Immunol* **14**, 428-436 (2013).
133. Hashimoto, M.*, et al.* CD8 T Cell Exhaustion in Chronic Infection and Cancer: Opportunities for Interventions. *Annu Rev Med* **69**, 301-318 (2018).
134. Medzhitov, R., Schneider, D.S. & Soares, M.P. Disease tolerance as a defense strategy. *Science* **335**, 936-941 (2012).
135. Klein, S.L. & Flanagan, K.L. Sex differences in immune responses. *Nat Rev Immunol* **16**, 626-638 (2016).
136. Flanagan, K.L., Fink, A.L., Plebanski, M. & Klein, S.L. Sex and Gender Differences in the Outcomes of Vaccination over the Life Course. *Annu Rev Cell Dev Biol* **33**, 577-599 (2017).
137. Fish, E.N. The X-files in immunity: sex-based differences predispose immune responses. *Nat Rev Immunol* **8**, 737-744 (2008).
138. Yen, Y.F.*, et al.* Sexual inequality in incident tuberculosis: a cohort study in Taiwan. *BMJ Open* **8**, e020142 (2018).
139. Nhamoyebonde, S. & Leslie, A. Biological differences between the sexes and susceptibility to tuberculosis. *J Infect Dis* **209 Suppl 3**, S100-106 (2014).
140. Gabriel, G. & Arck, P.C. Sex, immunity and influenza. *J Infect Dis* **209 Suppl 3**, S93-99 (2014).
141. Kim, H.I., Lim, H. & Moon, A. Sex Differences in Cancer: Epidemiology, Genetics and Therapy. *Biomol Ther (Seoul)* **26**, 335-342 (2018).
142. Fairweather, D. Sex differences in inflammation during atherosclerosis. *Clin Med Insights Cardiol* **8**, 49-59 (2014).
143. Scully, E.P. Sex Differences in HIV Infection. *Curr HIV/AIDS Rep* **15**, 136-146 (2018).
144. Das, B.*, et al.* Estrogen receptor-1 is a key regulator of HIV-1 latency that imparts gender-specific restrictions on the latent reservoir. *Proc Natl Acad Sci U S A* **115**, E7795-E7804 (2018).
145. Cooper, G.S. & Stroehla, B.C. The epidemiology of autoimmune diseases. *Autoimmun Rev* **2**, 119-125 (2003).
146. Rubtsova, K., Marrack, P. & Rubtsov, A.V. Sexual dimorphism in autoimmunity. *J Clin Invest* **125**, 2187-2193 (2015).
147. Levy, J.A. & Ziegler, J.L. Acquired immunodeficiency syndrome is an opportunistic infection and Kaposi's sarcoma results from secondary immune stimulation. *Lancet* **2**, 78-81 (1983).

148. Sonnabend, J.A., Witkin, S.S. & Purtilo, D.T. A multifactorial model for the development of AIDS in homosexual men. *Ann N Y Acad Sci* **437**, 177-183 (1984).
149. Sonnabend, J., Witkin, S.S. & Purtilo, D.T. Acquired immunodeficiency syndrome, opportunistic infections, and malignancies in male homosexuals. A hypothesis of etiologic factors in pathogenesis. *JAMA* **249**, 2370-2374 (1983).
150. Shearer, G.M. & Levy, R.B. Noninfectious cofactors in susceptibility to AIDS: possible contributions of semen, HLA alloantigens, and lack of natural resistance. *Ann N Y Acad Sci* **437**, 49-57 (1984).
151. Jennes, W.*, et al.* Suppressed cellular alloimmune responses in HIV-exposed seronegative female sex workers. *Clin Exp Immunol* **143**, 435-444 (2006).
152. Root-Bernstein, R.S. & DeWitt, S.H. Semen alloantigens and lymphocytotoxic antibodies in AIDS and ICL. *Genetica* **95**, 133-156 (1995).
153. Root-Bernstein, R.S. Non-HIV immunosuppressive factors in AIDS: a multifactorial, synergistic theory of AIDS aetiology. *Res Immunol* **141**, 815-838 (1990).
154. Ludlam, C.A.*, et al.* Human T-lymphotropic virus type III (HTLV-III) infection in seronegative haemophiliacs after transfusion of factor VIII. *Lancet* **2**, 233-236 (1985).
155. Kuebler, P.J.*, et al.* Persistent HIV Type 1 Seronegative Status Is Associated With Lower CD8+ T-Cell Activation. *The Journal of infectious diseases* **213**, 569-573 (2016).
156. Poudrier, J., Thibodeau, V. & Roger, M. Natural Immunity to HIV: a delicate balance between strength and control. *Clin Dev Immunol* **2012**, 875821 (2012).
157. Riddell, J.t., Amico, K.R. & Mayer, K.H. HIV Preexposure Prophylaxis: A Review. *JAMA* **319**, 1261-1268 (2018).
158. Hodes, R.J.*, et al.* Disease drivers of aging. *Ann N Y Acad Sci* **1386**, 45-68 (2016).
159. Ferrucci, L. & Fabbri, E. Inflammageing: chronic inflammation in ageing, cardiovascular disease, and frailty. *Nat Rev Cardiol* **15**, 505-522 (2018).
160. Derhovanessian, E.*, et al.* Hallmark features of immunosenescence are absent in familial longevity. *J Immunol* **185**, 4618-4624 (2010).
161. Strindhall, J.*, et al.* No Immune Risk Profile among individuals who reach 100 years of age: findings from the Swedish NONA immune longitudinal study. *Exp Gerontol* **42**, 753-761 (2007).
162. Bhat, V., Joshi, A., Sarode, R. & Chavan, P. Cytomegalovirus infection in the bone marrow transplant patient. *World J Transplant* **5**, 287-291 (2015).
163. Fedak, K.M., Bernal, A., Capshaw, Z.A. & Gross, S. Applying the Bradford Hill criteria in the 21st century: how data integration has changed causal inference in molecular epidemiology. *Emerg Themes Epidemiol* **12**, 14 (2015).
164. International HIV Controllers Study*, et al.* The major genetic determinants of HIV-1 control affect HLA class I peptide presentation. *Science* **330**, 1551-1557 (2010).
165. Erkeller-Yuksel, F.M.*, et al.* Age-related changes in human blood lymphocyte subpopulations. *J Pediatr* **120**, 216-222 (1992).
166. Wikby, A., Mansson, I.A., Johansson, B., Strindhall, J. & Nilsson, S.E. The immune risk profile is associated with age and gender: findings from three Swedish population studies of individuals 20-100 years of age. *Biogerontology* **9**, 299-308 (2008).

167. Jentsch-Ullrich, K., Koenigsmann, M., Mohren, M. & Franke, A. Lymphocyte subsets' reference ranges in an age- and gender-balanced population of 100 healthy adults--a monocentric German study. *Clin Immunol* **116**, 192-197 (2005).
168. Bisset, L.R., Lung, T.L., Kaelin, M., Ludwig, E. & Dubs, R.W. Reference values for peripheral blood lymphocyte phenotypes applicable to the healthy adult population in Switzerland. *Eur J Haematol* **72**, 203-212 (2004).
169. Santagostino, A*., et al.* An Italian national multicenter study for the definition of reference ranges for normal values of peripheral blood lymphocyte subsets in healthy adults. *Haematologica* **84**, 499-504 (1999).
170. Bofill, M*., et al.* Laboratory control values for CD4 and CD8 T lymphocytes. Implications for HIV-1 diagnosis. *Clin Exp Immunol* **88**, 243-252 (1992).
171. Reichert, T*., et al.* Lymphocyte subset reference ranges in adult Caucasians. *Clin Immunol Immunopathol* **60**, 190-208 (1991).
172. Maini, M.K*., et al.* Reference ranges and sources of variability of CD4 counts in HIV-seronegative women and men. *Genitourin Med* **72**, 27-31 (1996).
173. Shahabuddin, S. Quantitative differences in CD8+ lymphocytes, CD4/CD8 ratio, NK cells, and HLA-DR(+)-activated T cells of racially different male populations. *Clin Immunol Immunopathol* **75**, 168-170 (1995).
174. Freedman, D.S*., et al.* Black/white differences in leukocyte subpopulations in men. *Int J Epidemiol* **26**, 757-764 (1997).
175. Vuillier, F., Lapresle, C. & Dighiero, G. Comparative analysis of CD4-4B4 and CD4-2H4 lymphocyte subpopulations in HIV negative homosexual, HIV seropositive and healthy subjects. *Clin Exp Immunol* **71**, 8-12 (1988).
176. Lebranchu, Y., Thibault, G., Degenne, D. & Bardos, P. Abnormalities in CD4+ T lymphocyte subsets in patients with common variable immunodeficiency. *Clin Immunol Immunopathol* **61**, 83-92 (1991).
177. Amadori, A*., et al.* Genetic control of the CD4/CD8 T-cell ratio in humans. *Nat Med* **1**, 1279-1283 (1995).
178. Tsegaye, A*., et al.* Immunohematological reference ranges for adult Ethiopians. *Clin Diagn Lab Immunol* **6**, 410-414 (1999).
179. Kassu, A*., et al.* Distribution of lymphocyte subsets in healthy human immunodeficiency virus-negative adult Ethiopians from two geographic locales. *Clin Diagn Lab Immunol* **8**, 1171-1176 (2001).
180. Gratama, J.W*., et al.* Flow cytometric and morphologic studies of HNK1+ (Leu 7+) lymphocytes in relation to cytomegalovirus carrier status. *Clin Exp Immunol* **74**, 190-195 (1988).
181. Giorgi, J.V*., et al.* Quality control in the flow cytometric measurement of T-lymphocyte subsets: the multicenter AIDS cohort study experience. The Multicenter AIDS Cohort Study Group. *Clin Immunol Immunopathol* **55**, 173-186 (1990).
182. Wright, J.J*., et al.* Characterization of common variable immunodeficiency: identification of a subset of patients with distinctive immunophenotypic and clinical features. *Blood* **76**, 2046-2051 (1990).
183. Williams, R.C., Jr., Koster, F.T. & Kilpatrick, K.A. Alterations in lymphocyte cell surface markers during various human infections. *Am J Med* **75**, 807-816 (1983).
184. Yagi, M.J., Joesten, M.E., Wallace, J., Roboz, J.P. & Bekesi, J.G. Human immunodeficiency virus type 1 (HIV-1) genomic sequences and distinct changes in

CD8+ lymphocytes precede detectable levels of HIV-1 antibodies in high-risk homosexuals. *J Infect Dis* **164**, 183-188 (1991).
185. Kalayjian, R.C., *et al.* Age-related immune dysfunction in health and in human immunodeficiency virus (HIV) disease: association of age and HIV infection with naive CD8+ cell depletion, reduced expression of CD28 on CD8+ cells, and reduced thymic volumes. *J Infect Dis* **187**, 1924-1933 (2003).
186. Tollerud, D.J., *et al.* The influence of age, race, and gender on peripheral blood mononuclear-cell subsets in healthy nonsmokers. *J Clin Immunol* **9**, 214-222 (1989).
187. Fahey, J.L., *et al.* Quantitative changes in T helper or T suppressor/cytotoxic lymphocyte subsets that distinguish acquired immune deficiency syndrome from other immune subset disorders. *Am J Med* **76**, 95-100 (1984).
188. Wolfe, W.H., Miner, J.C. & Michalek, J.E. Immunological parameters in current and former US Air Force personnel. *Vaccine* **11**, 545-547 (1993).
189. Nowicki, M.J., *et al.* Correlates of CD4+ and CD8+ lymphocyte counts in high-risk immunodeficiency virus (HIV)-seronegative women enrolled in the women's interagency HIV study (WIHS). *Hum Immunol* **68**, 342-349 (2007).
190. Strindhall, J., *et al.* The inverted CD4/CD8 ratio and associated parameters in 66-year-old individuals: the Swedish HEXA immune study. *Age (Dordr)* **35**, 985-991 (2013).
191. Somma, C., Miller, J.J., 3rd, Silverman, E.D. & Link, M.P. Abnormal helper:suppressor T-cell ratio in the staff of a pediatric hospital. *N Engl J Med* **312**, 1573-1574 (1985).
192. Alam, I., Goldeck, D., Larbi, A. & Pawelec, G. Aging affects the proportions of T and B cells in a group of elderly men in a developing country--a pilot study from Pakistan. *Age (Dordr)* **35**, 1521-1530 (2013).
193. Borkow, G., *et al.* Chronic immune activation associated with intestinal helminth infections results in impaired signal transduction and anergy. *The Journal of clinical investigation* **106**, 1053-1060 (2000).
194. Kalinkovich, A., *et al.* Decreased CD4 and increased CD8 counts with T cell activation is associated with chronic helminth infection. *Clin Exp Immunol* **114**, 414-421 (1998).
195. Beddall, A.C., Al-Rubei, K., Williams, M.D. & Hill, F.G. Lymphocyte subset ratios and factor VIII usage in haemophilia. *Arch Dis Child* **60**, 530-536 (1985).
196. Nicolosi, A., *et al.* Incidence and risk factors of HIV infection: a prospective study of seronegative drug users from Milan and northern Italy, 1987-1989. *Epidemiology* **1**, 453-459 (1990).
197. Donahoe, R.M., *et al.* Mechanistic implications of the findings that opiates and other drugs of abuse moderate T-cell surface receptors and antigenic markers. *Ann N Y Acad Sci* **496**, 711-721 (1987).
198. Verboeket, S.O., *et al.* HIV-negative Men Who Have Sex with Men have higher CD8+ T-cell Counts and Lower CD4+/CD8+ T-cell Ratios compared to HIV-negative Heterosexual Men. *J Infect Dis* (2020).
199. Eibl, M.M., Mannhalter, J.W. & Zlabinger, G. Abnormal T-lymphocyte subpopulations in healthy subjects after tetanus booster immunization. *N Engl J Med* **310**, 198-199 (1984).

200. Sale, G.E., Alavaikko, M., Schaefers, K.M. & Mahan, C.T. Abnormal CD4:CD8 ratios and delayed germinal center reconstitution in lymph nodes of human graft recipients with graft-versus-host disease (GVHD): an immunohistological study. *Exp Hematol* **20**, 1017-1021 (1992).
201. Dutta, A., Uno, H., Lorenz, D.R., Wolinsky, S.M. & Gabuzda, D. Low T-cell subsets prior to development of virus-associated cancer in HIV-seronegative men who have sex with men. *Cancer Causes Control* (2018).
202. Sigel, K.*, et al.* Immunological and infectious risk factors for lung cancer in US veterans with HIV: a longitudinal cohort study. *Lancet HIV* **4**, e67-e73 (2017).
203. Greenwood, E.J.*, et al.* Simian Immunodeficiency Virus Infection of Chimpanzees (Pan troglodytes) Shares Features of Both Pathogenic and Non-pathogenic Lentiviral Infections. *PLoS Pathog* **11**, e1005146 (2015).
