# Supplementary material for: Immune resilience despite inflammatory stress promotes longevity and favorable health outcomes including resistance to infection
Source: Nat Commun. 2023 Jun 13;14:3286. doi: 10.1038/s41467-023-38238-6 (PMC10264401; doi:10.1038/s41467-023-38238-6)
Supplement: Supplementary file 3 — Description of Additional Supplementary Files [file 41467_2023_38238_MOESM3_ESM.pdf]

Description of Additional Supplementary Data Files

Supplementary Data 1. Cohorts/datasets and surveys.
a. Summary of cohorts/datasets evaluated.
b. Summary of literature survey performed.

Supplementary Data 2. Baseline characteristics of study participants from HIV− and HIV+ cohorts

Supplementary Data 3. CD4+ and CD8+ T-cell counts and CD4:CD8 T-cell ratio by IHG status in HIV− cohorts.

Supplementary Data 4. Characteristics of the female sex workers (FSWs)
a. 762 HIV− FSWs.
b. 449 HIV− FSWs.

Supplementary Data 5. Baseline characteristics of renal transplant recipients (RTRs).

Supplementary Data 6. Characteristics of study participants from the HIV+ cohorts

Supplementary Data 7. COVID-19 cohort characteristics by baseline IHG status.

Supplementary Data 8. Associations of behavioral activity score (BAS), total STI score and IHGs with risk of HIV seroconversion in HIV− FSWs.
a. With IHG in 449 HIV− FSWs
b. With IHG subgrades in 762 HIV− FSWs

Supplementary Data 9. Mortality HRs in COVID-19 cohort and FHS offspring cohort
a. Hazard models of the COVID-19 BP-GO term signature scores in the COVID-19 and FHS cohorts
b. SAS and MAS gene signatures: GO terms and overlap of genes
c. Hazard models of SAS, MAS, and IMM-AGE gene signatures in the COVID-19 and FHS cohorts
d. Single hazard model of the MAS and SAS signature scores.
e. Hazard models of SAS-1/MAS-1 profiles in the FHS Cohort

Supplementary Data 10. Correlation of genes in gene signature scores (SAS-1, MAS-1, and IMM-AGE) with three immunological markers in 56 HIV+ patients on ART.

Supplementary Data 11. Immunological associations in non-human primates.

Supplementary Data 12. Seventy-five immune markers examined in the SardiNIA cohort and their association with IHG-III or IHG-IV and/or age or neither (neutral)

Supplementary Data 13. Analysis of gene expression datasets.
a. Characteristics, sources, platform information and normalization performed in all gene expression datasets evaluated in the current study.
b. Gene representation of SAS-1, MAS-1, and IMM-AGE gene signature in all the datasets evaluated in the current study.
c. Sources for data access to various cohorts used in the current study.

Supplementary Data 14.
a. Exact *P* values per figure panel.
b. Exact *P* values for figure panel 5c.
