# Supplementary material for: Immune resilience despite inflammatory stress promotes longevity and favorable health outcomes including resistance to infection
Source: Nat Commun. 2023 Jun 13;14:3286. doi: 10.1038/s41467-023-38238-6 (PMC10264401; doi:10.1038/s41467-023-38238-6)
Supplement: Supplementary file 18 — Reporting Summary [file 41467_2023_38238_MOESM18_ESM.pdf]

nature portfolio

Corresponding author(s): Sunil K. Ahuja, MD

Last updated by author(s): Mar 17, 2023

Reporting Summary

Nature Portfolio wishes to improve the reproducibility of the work that we publish. This form provides structure for consistency and transparency in reporting. For further information on Nature Portfolio policies, see our Editorial Policies and the Editorial Policy Checklist.

Statistics

For all statistical analyses, confirm that the following items are present in the figure legend, table legend, main text, or Methods section.

n/a	Confirmed	
☐	☒	The exact sample size (*n*) for each experimental group/condition, given as a discrete number and unit of measurement
☐	☒	A statement on whether measurements were taken from distinct samples or whether the same sample was measured repeatedly
☐	☒	The statistical test(s) used AND whether they are one- or two-sided<br>*Only common tests should be described solely by name; describe more complex techniques in the Methods section.*
☐	☒	A description of all covariates tested
☐	☒	A description of any assumptions or corrections, such as tests of normality and adjustment for multiple comparisons
☐	☒	A full description of the statistical parameters including central tendency (e.g. means) or other basic estimates (e.g. regression coefficient) AND variation (e.g. standard deviation) or associated estimates of uncertainty (e.g. confidence intervals)
☒	☐	For null hypothesis testing, the test statistic (e.g. *F*, *t*, *r*) with confidence intervals, effect sizes, degrees of freedom and *P* value noted<br>*Give P values as exact values whenever suitable.*
☒	☐	For Bayesian analysis, information on the choice of priors and Markov chain Monte Carlo settings
☒	☐	For hierarchical and complex designs, identification of the appropriate level for tests and full reporting of outcomes
☐	☒	Estimates of effect sizes (e.g. Cohen's *d*, Pearson's *r*), indicating how they were calculated

Our web collection on statistics for biologists contains articles on many of the points above.

Software and code

Policy information about availability of computer code

Data collection	All download was performed in R statistical environment ((v2, v3, v4). Following packages were used: GEOquery_2.56.0, ArrayExpress_1.48.0 etc. GEO2R R script sourced from NCBI GEO [source: https://www.ncbi.nlm.nih.gov/geo/info/geo2r.html] was used for download and analyses of GEO datasets and a script from vignette of ArrayExpress R package was used for download and analyses of ArrayExpress datasets. These scripts were used to download and normalize the data.
Data analysis	All analysis including transcriptomics (normalization and score analysis) was performed in R statistical environment (v2, v3, v4) . Following packages were used: GEOquery_2.56.0, ArrayExpress_1.48.0, Biobase_2.50.0, limma_3.46.0, preprocessCore_1.52.1, stringr_1.4.0, geepack_1.3-1, corrplot_0.84, DESeq_1.39.0, reshape_0.8.8, downloader_0.4, affxparser_1.60.0, RCurl_1.98-1.2, BiocGenerics_0.36.1, survival_3.1-12, lmtest_0.9-38, and others

For manuscripts utilizing custom algorithms or software that are central to the research but not yet described in published literature, software must be made available to editors and reviewers. We strongly encourage code deposition in a community repository (e.g. GitHub). See the Nature Portfolio guidelines for submitting code & software for further information.

Data

Policy information about availability of data

All manuscripts must include a data availability statement. This statement should provide the following information, where applicable:

- Accession codes, unique identifiers, or web links for publicly available datasets
- A description of any restrictions on data availability
- For clinical datasets or third party data, please ensure that the statement adheres to our policy

The phenotype and processed gene expression data generated are available in Figshare database for
COVID-19 cohort (https://figshare.com/projects/Ahuja_Lab_COVID-19_dataset/158732) and
HIV dataset (https://figshare.com/projects/Ahuja_Lab_HIV_dataset/158681).

Individual level raw data files of the VA COVID-19 cohort cannot be shared publicly due to data protection and confidentiality requirements. South Texas Veterans Health Care System (STVHCS) at San Antonio, Texas is the data holder for the COVID-19 data used in this study. Data can be made available to approved researchers for analysis after securing relevant permissions via review by the Institutional Review Board for use of the data collected under this protocol. Inquiries regarding data availability should be directed to the corresponding author. Accession links to all data generated or analyzed during this study are included in Supplementary Data 13a. Source data are provided with this paper. All other patient/individual-level raw data (including HIV dataset from early infection cohort) underlying this article cannot be shared publicly due to data protection and confidentiality requirements. The data holders and contacts for inquiries to data access are in listed in Supplementary Data 13c for the following cohorts: SardiNIA, HIV– UCSD, Schistosomiasis in Kenyan children, renal transplant recipients (RTR), female sex workers (FSWs) from The Kenya Majengo Observational Cohort Study cohort (MOCS), Early infection cohort (EIC), primary infection cohort (PIC), SIV– and SIV+ Sooty mangabeys, SIV– Rhesus macaques, and Collaborative Cross (CC)-RIX mice.

Database and sources of publicly available gene expression datasets analyzed in this study are provided in Supplementary Data 13a, for the following cohorts:
SLE dataset from GEO (GSE49454 - https://www.ncbi.nlm.nih.gov/geo/query/acc.cgi?acc=GSE49454),
HIV/TB Meta analysis with Finnish DILGOM cohort from GEO and Array Express (GSE29429 - https://www.ncbi.nlm.nih.gov/geo/query/acc.cgi?acc=GSE29429;
GSE19439 - https://www.ncbi.nlm.nih.gov/geo/query/acc.cgi?acc=GSE19439;
GSE19442 - https://www.ncbi.nlm.nih.gov/geo/query/acc.cgi?acc=GSE19442;
GSE19444 - https://www.ncbi.nlm.nih.gov/geo/query/acc.cgi?acc=GSE19444;
E-TABM-1036 - https://www.ebi.ac.uk/biostudies/arrayexpress/studies/E-TABM-1036),
San Antonio Family Heart Study from Array Express (E-TABM-305 - https://www.ebi.ac.uk/biostudies/arrayexpress/studies/E-TABM-305),
FHS Offspring cohort from dbGaP (phs000007.v30.p11- https://www.ncbi.nlm.nih.gov/projects/gap/cgi-bin/study.cgi?study_id=phs000007.v30.p11;
phs000363.v17.p11 - https://www.ncbi.nlm.nih.gov/projects/gap/cgi-bin/study.cgi?study_id=phs000363.v17.p11),
Sepsis Meta analysis cohorts from ArrayExpress (E-MATB-4421 - https://www.ebi.ac.uk/biostudies/arrayexpress/studies/E-MTAB-4421;
E-MATB-4451 - https://www.ebi.ac.uk/biostudies/arrayexpress/studies/E-MTAB-4451;
E-MATB-5273 - https://www.ebi.ac.uk/biostudies/arrayexpress/studies/E-MTAB-5273;
E-MATB-5274 - https://www.ebi.ac.uk/biostudies/arrayexpress/studies/E-MTAB-5274),
Sepsis dataset from ArrayExpress (E-MATB-1548 - https://www.ebi.ac.uk/biostudies/arrayexpress/studies/E-MTAB-1548),
Vitality 90+ Study cohort from GEO (GSE65218 - https://www.ncbi.nlm.nih.gov/geo/query/acc.cgi?acc=GSE65218;
GSE65219 - https://www.ncbi.nlm.nih.gov/geo/query/acc.cgi?acc=GSE65219),
HIV-1 Infection dataset from GEO (GSE16363 - https://www.ncbi.nlm.nih.gov/geo/query/acc.cgi?acc=GSE16363),
Influenza and other acute respiratory viral infections dataset from GEO (GSE68310 - https://www.ncbi.nlm.nih.gov/geo/query/acc.cgi?acc=GSE68310),
Influenza A H1N1 and H3N2 virus infection dataset from GEO (GSE52428 - https://www.ncbi.nlm.nih.gov/geo/query/acc.cgi?acc=GSE52428),
symptomatic respiratory viral infection from GEO (GSE17156 - https://www.ncbi.nlm.nih.gov/geo/query/acc.cgi?acc=GSE17156),
Burn injury dataset from GEO (GSE182616 - https://www.ncbi.nlm.nih.gov/geo/query/acc.cgi?acc=GSE182616),
Sepsis cohort from GEO (GSE185263 - https://www.ncbi.nlm.nih.gov/geo/query/acc.cgi?acc=GSE185263),
Severe influenza dataset from GEO (GSE111368 - https://www.ncbi.nlm.nih.gov/geo/query/acc.cgi?acc=GSE111368),
Influenza-infected pre-Collaborative Cross (CC) lines dataset from GEO (GSE30506 - https://www.ncbi.nlm.nih.gov/geo/query/acc.cgi?acc=GSE30506).

Aggregate data presented for these cohorts in the current study are provided in the source data file.

Immunophenotyping data from SardiNIA cohort used in Fig. 10 are derived and sourced from Orrù et al. (doi: 10.1016/j.cell.2013.08.041) and are shared with us by the co-authors. Data from renal transplant recipients (RTR) are derived and sourced from Bottomley et al. (doi: 10.1681/ASN.2015030250) and are shared with us by the co-author.

The sources of the data for literature survey (Fig. 5c) are summarized in Supplementary Table 2.

Human research participants

Policy information about studies involving human research participants and Sex and Gender in Research.

Reporting on sex and gender	Sex was self-reported. No data on gender were collected. Cohort characteristics are described in Methods and Supplementary Information.<br>The reported findings apply to both sexes.<br>Yes, sex and gender were considered in study design.<br>The sex and/or gender was determined based on self-reporting.<br>The source data are provided as Source Data file.

	The overall numbers from multiple cohorts shown in sample size below and details are provided in the Supplementary Information and Supplementary Data 2.<br>Sex based analysis was performed and reported where data was collected and available.
Population characteristics	All covariate (age, sex, and others as appropriate) are described in detail in the Cohort description in Methods and Supplementary Information and Supplementary Data files.
Recruitment	COVID-19 cohort: This is an ongoing, prospective observational cohort study of patients testing positive for SARS-CoV-2 evaluated at the Audie L. Murphy VA Medical Center, South Texas Veterans Health Care System (STVHCS), San Antonio, Texas. In the current study, patients seen from March 20, 2020 through November 15, 2020. were used. Patients were followed during hospitalization and/or a minimum of 30 days from inclusion.<br>All other cohorts are from collaborators and are from previously published works.
Ethics oversight	All studies were approved by the Institutional Review Boards at the University of Texas Health San Antonio (Protocol# HSC20100396HR) and institutions participating in this study. Approved institutional review board protocol numbers for human studies from cohort contributors are as follows:<br><br>SardiNIA cohort : Ethical Committee ASL 1 Sassari (PROT# 2171/CE)<br>HIV- UCSD cohort: UCSD IRB Administration (IRB# 172092, Version 16 in KIRB)<br>Kenyan children with Schistosomiasis: Kenya Medical Research Institute National Ethical Review Committee; Institutional Review Board for Human Studies at University Hospitals of Cleveland Case Medical Center<br>RTR cohort: Oxford University Hospitals Research & Development (Reference# HH/AA/OD/10308); West of Scotland Research Ethics Service (REC reference# 12/WS/0288)<br>FSW cohort: Kenyatta National Hospital - University of Nairobi ERC (Ref# KNH/ERC/R/10; Protocol# P211/09/2006)<br>PIC: USCD IRB Administration (IRB# 191088, Version 16 in KIRB)<br>EIC: USUHS IRB (FWA 00001628; DoD Assurance P60001; Protocol# 357499 (IDCRP-000-05))

Note that full information on the approval of the study protocol must also be provided in the manuscript.

Field-specific reporting

Please select the one below that is the best fit for your research. If you are not sure, read the appropriate sections before making your selection.

☒ Life sciences ☐ Behavioural & social sciences ☐ Ecological, evolutionary & environmental sciences

For a reference copy of the document with all sections, see nature.com/documents/nr-reporting-summary-flat.pdf

Life sciences study design

All studies must disclose on these points even when the disclosure is negative.

Sample size	No priori sample size calculation was performed as this is an epidemiological-mechanistic study and these were secondary analyses. The study involves varied cohorts with 48,936 human samples, 279 non-human primates and 378 mice samples in total. All samples from the cohort were used, unless specifically stated in the statistical section of the specific analysis. Furthermore, given the wide range of cohorts (including several large datasets like SardiNIA [n=3,896]; Framingham Heart Study [n=2,308]), conditions (Aging, HIV, COVID-19, sepsis, influenza etc.), and model systems (humans, non-human primates, and mice) where IHGs were examined under, we believe that the sample size are large enough and sufficient for the results to be highly reproducible in the dataset tested. All data presented are reproducible using the appropriate datasets and criteria used.
Data exclusions	No data were excluded from the analyses that were designed to test the hypothesis following cohort specific inclusion/exclusion criteria, unless specifically stated in the statistical section of the figure panel.
Replication	This study examines metrics of immunological resilience in a wide-range of contexts, including acute/chronic infections, autoimmunity, aging, cancers, and vaccines. An extensive Methods section has been added that includes a Statistics & Reproducibility section. This section provides general information on the study design and how statistical analyses were conducted and detailed in the statistics per panel section in the Supplementary Information. In addition, each figure is linked with a source document for reproducibility. Furthermore, given the wide range of cohorts and conditions that IHGs were examined under, we believe these results to be highly reproducible. All data presented are reproducible using the appropriate datasets and criteria used.
Randomization	All samples were grouped according to predefined criteria and covariates were adjusted as appropriate. This was not an interventional study, or primary analyses, therefore no pre-hoc randomization was used.
Blinding	This was not an interventional study, therefore no blinding was used.

Reporting for specific materials, systems and methods

We require information from authors about some types of materials, experimental systems and methods used in many studies. Here, indicate whether each material, system or method listed is relevant to your study. If you are not sure if a list item applies to your research, read the appropriate section before selecting a response.

Materials & experimental systems

n/a	Involved in the study
☐	☒ Antibodies
☒	☐ Eukaryotic cell lines
☒	☐ Palaeontology and archaeology
☐	☒ Animals and other organisms
☒	☐ Clinical data
☒	☐ Dual use research of concern

Methods

n/a	Involved in the study
☒	☐ ChIP-seq
☐	☒ Flow cytometry
☒	☐ MRI-based neuroimaging

Antibodies

Antibodies used	All data using antibodies are from previously published work by Orru et al., (doi: 10.1016/j.cell.2013.08.041) and reanalyzed based on groupings in the present study.
Validation	Not applicable, as we used derived data from previously published work by Orru et al., (doi: 10.1016/j.cell.2013.08.041) and are shared with us by the co-authors

Animals and other research organisms

Policy information about studies involving animals; ARRIVE guidelines recommended for reporting animal research, and Sex and Gender in Research

Laboratory animals	Sooty mangabeys; rhesus macaques; and CC-RIX mice
Wild animals	N/A
Reporting on sex	Data from both sexes are provided where applicable.
Field-collected samples	N/A
Ethics oversight	All animal studies were approved by institutions participating in this study and the protocol numbers for animal studies from cohort contributors are as follows:<br>Sooty mangabeys - EMORY University: IACUC (Protocol ID: PROTO201700044)<br>Rhesus macaques - Internal Review Board, Kunming Institute of Zoology, Chinese Academy of Sciences (Approval# SYDW-2012091001)<br>CC-RIX mice - UNC-Chapel Hill IACUC (IACUC ID# 15-175.0)

Note that full information on the approval of the study protocol must also be provided in the manuscript.

Flow Cytometry

Plots

Confirm that:

- ☒ The axis labels state the marker and fluorochrome used (e.g. CD4-FITC).
- ☒ The axis scales are clearly visible. Include numbers along axes only for bottom left plot of group (a 'group' is an analysis of identical markers).
- ☒ All plots are contour plots with outliers or pseudocolor plots.
- ☒ A numerical value for number of cells or percentage (with statistics) is provided.

Methodology

Sample preparation	All flow cytometry data are from previously published works by Orru et al., (doi: 10.1016/j.cell.2013.08.041) and are shared with us by the co-authors
Instrument	*Identify the instrument used for data collection, specifying make and model number.*

Software	*Describe the software used to collect and analyze the flow cytometry data. For custom code that has been deposited into a community repository, provide accession details.*
Cell population abundance	*Describe the abundance of the relevant cell populations within post-sort fractions, providing details on the purity of the samples and how it was determined.*
Gating strategy	*Describe the gating strategy used for all relevant experiments, specifying the preliminary FSC/SSC gates of the starting cell population, indicating where boundaries between "positive" and "negative" staining cell populations are defined.*

☒ Tick this box to confirm that a figure exemplifying the gating strategy is provided in the Supplementary Information.
